# The contribution of cyclic imide stereoisomers on cereblon-dependent activity†

**DOI:** 10.1039/d5sc01371b

**Published:** 2025-05-28

**Authors:** Yuka Amako, Saki Ichikawa, Hannah C. Lloyd, N. Connor Payne, Zhi Lin, Andrew S. Boghossian, Matthew G. Rees, Melissa M. Ronan, Jennifer A. Roth, Qian Zhu, Bogdan Budnik, Ralph Mazitschek, Christina M. Woo

**Affiliations:** a Department of Chemistry and Chemical Biology, Harvard University Cambridge MA 02138 USA cwoo@chemistry.harvard.edu; b Center for Systems Biology, Massachusetts General Hospital Boston MA 02114 USA; c Broad Institute of MIT and Harvard Cambridge MA 02142 USA; d Mass Spectrometry and Proteomics Resource Lab (MSPRL), Division of Science, Faculty of Arts and Sciences, Harvard University Cambridge MA 02138 USA; e Harvard T.H. Chan School of Public Health Boston MA 02115 USA

## Abstract

Thalidomide, lenalidomide, and their derivatives mimic glutarimide and aspartimide protein modifications that give rise to a motif recognized by the E3 ligase substrate adapter cereblon (CRBN). These cyclic imides have a chiral center that, given the biological significance of chirality, may influence CRBN's function and therapeutic applications. Here, we systematically examine cyclic imides in small molecules, peptides, and proteins to assess their racemization, CRBN engagement, ternary complex formation *in vitro*, and resulting degradation outcomes in cells. While the thalidomide-binding domain of CRBN consistently favors the (*S*)-stereoisomer across all cyclic imide small molecule ligands and engineered proteins, we find that, in some cases, the (*R*)-stereoisomer can bind to CRBN, either enhancing or hindering the eventual target engagement and degradation. Lenalidomide and its derivatives racemize more rapidly (*t*_50%ee_ = 4–5 h) than the C-terminal cyclic imide under non-enzymatic conditions. These findings highlight that although the (*S*)-stereoisomer of the cyclic imide is the primary ligand for the thalidomide-binding domain of CRBN, the (*R*)-stereoisomer, if present, has the potential to contribute to CRBN-dependent cellular activity.

## Abstract 2

A systematic investigation of cyclic imide stereoisomers in small molecules, peptides, and proteins, examining their racemization, CRBN engagement, ternary complex formation *in vitro*, and resulting degradation outcomes in cells.

## Graphical abstract

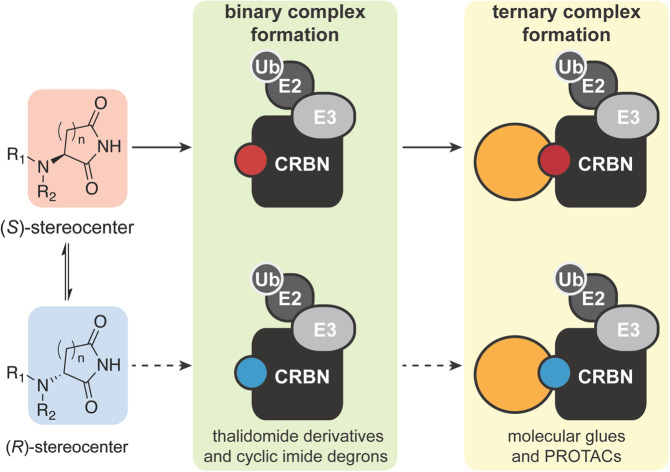

## Introduction

Thalidomide, lenalidomide, and derivatives engage the thalidomide-binding domain of the E3 ligase substrate adapter CRBN^1^ to alter recruitment, ubiquitination, and proteasomal degradation of neosubstrates like IKZF1, IKZF3, CK1α, and GSPT1.^2–5^ Degradation of these neosubstrates partly underlie the clinical outcomes of these ligands in multiple myeloma, del(5q)MDS, and other hematopoietic malignancies.^6^ In other contexts, CRBN-dependent degradation of substrates like SALL4,^7,8^ p63,^9^ and PLZF^10^ by thalidomide drives teratogenic effects observed during development. Thalidomide additionally possesses hypnotic, anti-inflammatory, and anti-angiogenesis effects, although mechanistic targets for these activities have not been fully characterized.^11^

**Fig. 1:**
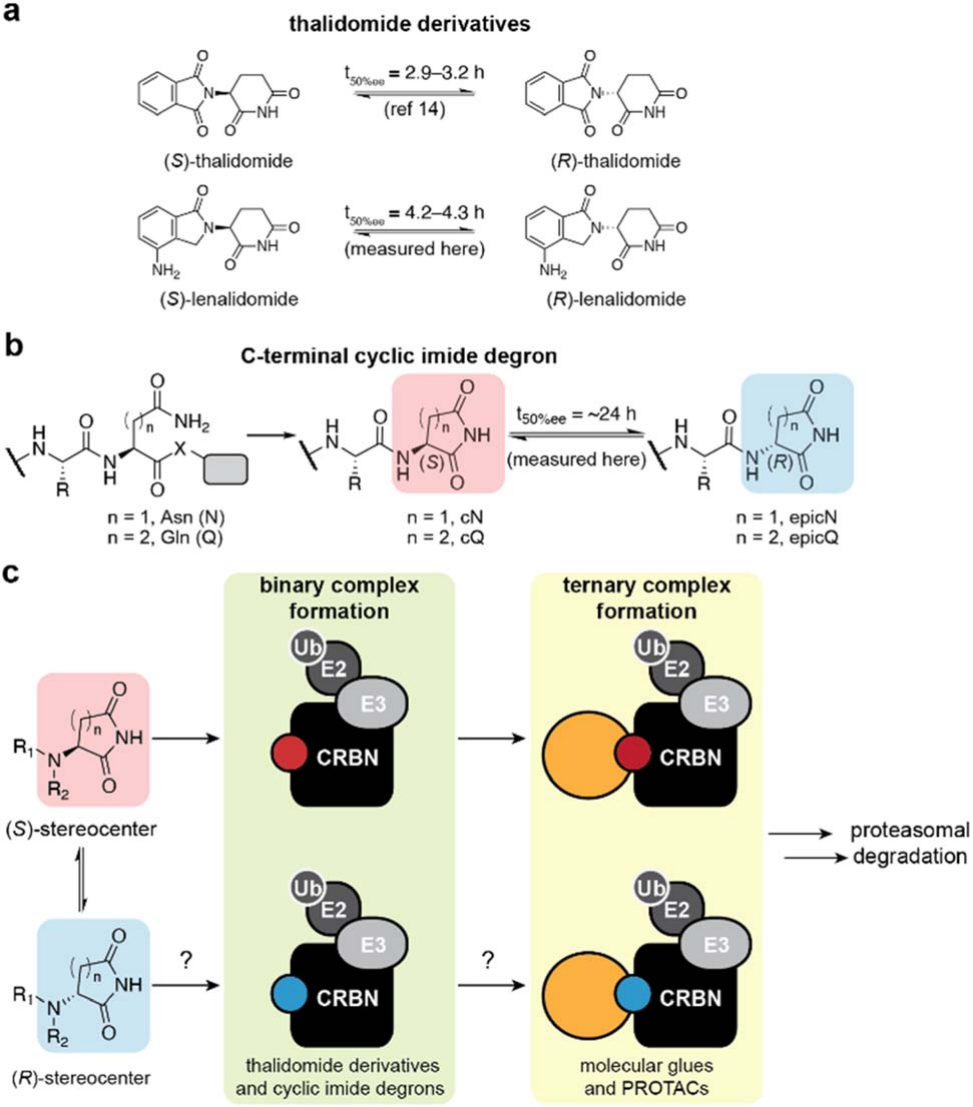
Structures and interaction modes of the cyclic imides with CRBN. (a) Structures of the (*S*)- and (*R*)-enantiomers of thalidomide and lenalidomide. (b) Formation of the (*S*)- and (*R*)-stereoisomers of the C-terminal cyclic imide degron. X = leaving group. (c) Schematic of evaluated binary and ternary complexes with CRBN and stereoisomers of aspartimide or glutarimide ligands.

Thalidomide has a single chiral center, which has driven extensive investigations into the distinct biological effects of each enantiomer that predate more recent studies of these ligands with CRBN (Fig. 1a).^11,12^ These investigations have popularized associations of (*S*)-thalidomide with teratogenicity^13^ and (*R*)-thalidomide with sedation,^14^ although attribution of phenotypic differences to a specific thalidomide enantiomer is challenging due to rapid racemization of thalidomide both *in vivo* (*t*_1/2_ = 2.3 h in human blood and 4–6 h in human subjects)^15^ and *in vitro* (*t*_50%ee_ = 2.9–3.2 h).^16^ With the more recent association of these ligands with CRBN, structural and biochemical studies have revealed that (*S*)-thalidomide has an approximately 10-fold greater binding affinity to CRBN than (*R*)-thalidomide and therefore CRBN-dependent effects are primarily mediated by the (*S*)-enantiomer of thalidomide.^17–20^ These reports indicate that (*R*)-thalidomide weakly engages CRBN^17^ and can recruit SALL4 *in vitro*,^18^ however, whether the stereoisomers of other derivatives engage CRBN and influence the overall degradation outcome is unexamined in the context of monofunctional or bifunctional degraders (Fig. 1a).

Recognition of one or both enantiomers of thalidomide by CRBN may also be related to the physiological function of the thalidomide-binding domain of CRBN. Recently, we discovered that CRBN recognizes the C-terminal cyclic imide degron, which is a motif sufficient to promote ubiquitination and degradation on a protein substrate (Fig. 1b).^21^ C-Terminal cyclic imides are underappreciated modifications from cyclization of asparagine (cN) or glutamine (cQ) residues. C-Terminal cyclic imides resemble internal cyclic imide intermediates that arise from deamidation, a form of protein damage.^22^ For instance, the reported half-life for *in vitro* racemization of the internal cyclic imide is 19.4 h for the hexapeptide VYPcNGA.^23^ If racemization of the C-terminal aspartimide and glutarimide occur on similar timescales, CRBN may recognize complexes with substrates bearing either epimer of the C-terminal cyclic imide degron, thereby promoting the removal of the protein (Fig. 1c).

Here, we characterize the stereoisomers of cyclic imide ligands in small molecules and proteins for their racemization rates, binding affinity against CRBN, and ability to afford productive ternary complexes that result in substrate ubiquitination and degradation *in vitro* and in cells. We perform binding site mapping with the thalidomide-binding domain of CRBN using chemical probes, generate a model for CRBN engagement of both stereoisomers, and report a detailed investigation of the contribution of the cyclic imide stereoisomers in the context of monofunctional degraders, bifunctional degraders, and engineered proteins to overall degradation outcomes. Collectively, we demonstrate across a series of small molecule and protein ligands for CRBN that while the (*S*)-stereoisomer preferentially binds and dictates the majority of the degradation activity, the (*R*)-stereoisomer can also ligand to CRBN and influence substrate degradation.

## Results and discussion

### Binding mode of pLen to the thalidomide-binding domain

**Fig. 2:**
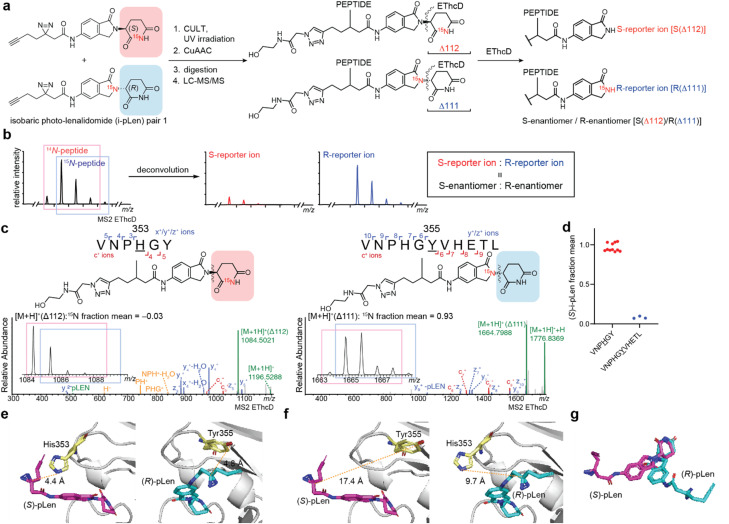
Binding site mapping of the thalidomide-binding domain of CRBN with (*S*)- and (*R*)-i-pLen probes. (a) Experimental workflow for mapping i-pLen probe binding sites within the thalidomide-binding domain of CRBN (CULT). (b) Enantioselective binding of i-pLen measured by LC-MS/MS. The bond between the isoindolinone and the glutarimide is cleaved by EThcD in MS2 and the relative engagement of (*S*)- and (*R*)-i-pLen is quantified by the intensity of the deconvoluted reporter ion. (c) Representative EThcD spectra and quantification of two thalidomide-binding domain peptides conjugated with the (*S*)- and (*R*)-i-pLen probes. (d) Deconvoluted enantiomeric ratio at each binding site based on peptide-spectrum match (PSM) counts. (e and f) Models of (*S*)- and (*R*)-pLen labeling of the thalidomide-binding domain generated from the crystal structure of the lenalidomide-CRBN/DDB1 complex (PDB: 4CI2). (e) Left: (*S*)-pLen (magenta) bound to the thalidomide-binding domain (gray); His353 highlighted in yellow. Right: (*R*)-pLen (blue) bound to the thalidomide-binding domain (gray); Tyr355 highlighted in yellow. (f) The distance between the diazirine carbon of (*S*)-pLen and Tyr355, as well as that of (*R*)-pLen and His353, exceeded the labeling radius of the diazirine. (g) Overlay of (*S*)- and (*R*)-photo-lenalidomide in the complex.

To determine if both enantiomers of thalidomide derivatives like lenalidomide can simultaneously engage CRBN, we envisioned that a chemoproteomics photoaffinity labeling (PAL) approach would reveal the structural configuration of lenalidomide's binding within the thalidomide-binding domain of CRBN in solution. This method facilitates the identification of both targets^24–27^ and binding sites^28^ for small molecule ligands, and can distinguish between the interactions of enantiomeric probes.^29^ PAL is particularly suited for chiral compounds prone to racemization, such as those containing the cyclic imides, since PAL can be initiated shortly after incubation (0.5–1 h). This rapid, time-resolved capture of dynamic interactions effectively circumvents challenges from racemization, which can be significant in other methods. For instance, crystal structures typically reflect the most stable binding conformation of a compound, but within the extended crystallization timeframe, the (*R*)-stereoisomer may have sufficient time to racemize to the (*S*)-stereoisomer, obscuring the stereospecific interactions of the original isomer. We, therefore, surmised that the photolenalidomide (pLen) probe^30^ would enable visualization of the enantiospecific binding mode and site occupancy at the thalidomide-binding domain. Inspired by chemoproteomic methods using enantioprobes^29^ and isobaric labeling strategies for quantitative proteomics,^31,32^ we developed isobaric photolenalidomide (i-pLen) probes designed to produce different reporter ions for quantification after fragmentation (Fig. 2a, S1a and b†). A set of i-pLen probes with inversion of the heavy and light labels were also prepared (Fig. S1c†).

The i-pLen probes reliably captured enantiomer composition across a dynamic range, as fragmentation at the desired carbon–nitrogen bond led to the neutral loss of the glutarimide (Δ111 or Δ112 Da) and quantification of the complementary ion aligned with expected values across various ratios analyzed by tandem mass spectrometry (Fig. S2a†). We also confirmed that pLen and lenalidomide have identical racemization rates in RPMI media at 37 °C [*t*_50%ee_ (*S*)-lenalidomide = 4.2 h, *t*_50%ee_ (*R*)-lenalidomide = 4.3 h, *t*_50%ee_ (*S*)-pLen = 5.8 h, *t*_50%ee_ (*R*)-pLen = 4.9 h] (Fig. S2b†). The racemization half-life (*t*_50%ee_) measured in 0.1 M phosphate buffer (pH 7.4) at 37 °C is previously reported as 3.2 h for (*S*)-thalidomide and 2.9 h for (*R*)-thalidomide.^16^ Although these data were measured in different buffers, they suggest that lenalidomide racemizes more slowly than thalidomide. Indeed, the predicted p*K*_a_ of the stereocenters of thalidomide and lenalidomide was 15.73 and 16.17, respectively, using the AIMNet2-based p*K*_a_ prediction platform.^33–36^ This calculation suggests that the stereocenter of lenalidomide is less acidic than thalidomide, presumably due to the absence of an additional carbonyl group, and is therefore less prone to racemization.

An equimolar mixture of the i-pLen probes was incubated with the thalidomide-binding domain under non-saturating conditions,^37^ and the binding event was covalently captured by photolysis after 30 min at 24 °C to minimize the contribution from racemization (ee > 90% after 30 min at 37 °C, Fig. 2a, S3a and b†). The labeled thalidomide-binding domain was then treated with a cleavable biotin azide probe^25^ by copper-catalyzed azide–alkyne cycloaddition (CuAAC) and digested *in situ* with chymotrypsin (Fig. S3c†). The digested samples were analyzed by tandem mass spectrometry with collision-induced dissociation (CID), higher energy C-trap dissociation (HCD), and electron-transfer/higher-energy collision dissociation (EThcD) fragmentation. The spectra were assigned by database searching to identify the binding site, and the reporter ions produced by fragmentation across the isoindolinone and the glutarimide ring were manually assigned. Quantification of the enantiomeric ratio at each binding site was performed by deconvoluting the ion cluster containing the isoindolinone^38^ with an in-house algorithm adapted from the TMTc^+^ deconvolution method developed by Wühr and co-workers,^39^ where a peptide conjugated with ^14^*N*-isoindolinone represents the (*S*)-enantiomer and the ^15^*N*-isoindolinone represents the (*R*)-enantiomer (Fig. 2b).

The binding sites captured by the i-pLen probes were mapped to two unique peptides within the thalidomide-binding domain, corresponding to CRBN residues 350–355 and residues 350–360, with the modification occurring on His353 and Tyr355, respectively (Fig. 2c, S3d, Tables S2 and3†). These amino acids are located in the conformationally flexible sensor loop of the thalidomide-binding domain of CRBN.^40^ Deconvolution and quantification of the (*S*)- and (*R*)-reporter ions revealed that His353 was primarily labeled by the (*S*)-enantiomer, while Tyr355 was almost exclusively labeled by the (*R*)-enantiomer (Fig. 2d). Inverting the heavy nitrogen in the i-pLen probes preserved the selective labeling of His353 by the (*S*)-enantiomer and Tyr 355 by the (*R*)-enantiomer (Fig. S4, Tables S4 and 5†). Racemic pLen primarily labels CRBN His353,^30^ which is in line with the expected primary engagement of the thalidomide-binding domain with the (*S*)-enantiomer of lenalidomide. Nonetheless, the distinct labeling patterns from i-pLen probes indicate that both enantiomers can engage the thalidomide-binding domain in a racemic mixture.

The labeling of CRBN His353 and Tyr355 by (*S*)- and (*R*)-i-pLen, respectively, is indicative of two structurally distinct binding modes. To gain further structural insight, (*S*)- and (*R*)-pLen were docked to the thalidomide-binding domain using a crystal structure of the lenalidomide-CRBN/DDB1 complex (PDB 4CI2).^20^ The docking results for the top 30 refined poses were clustered and rescored with the GBVI/WSA Δ*G* scoring function. The top five poses with the lowest S scores, representing the predicted binding free energy, were further analyzed by measuring the distance between the diazirine carbon and CRBN His353 or Tyr355 (Fig. S5†). These poses exhibit strong consensus, showing that the diazirine carbon of (*S*)-pLen is positioned closer to His353, while that of (*R*)-pLen is oriented towards Tyr355. The docked structure with the lowest *S* score positioned the diazirine carbon of (*S*)-pLen 4.4 Å from His353 and that of (*R*)-pLen 4.8 Å from Tyr355, which are both within the PAL radius (Fig. 2e).^28^ By contrast, the distance from the diazirine carbon of (*S*)-pLen to Tyr355 and that of (*R*)-pLen to His353 was outside of the labeling radius of the alkyl diazirine (Fig. 2f).^28^ Collectively, these models highlight the conformationally distinct binding modes of the photolenalidomide enantiomers to the thalidomide-binding domain of CRBN (Fig. 2g), as independently and experimentally supported by the proteomics-based binding site analysis.

### Contribution of stereoisomers in monofunctional degraders

**Fig. 3:**
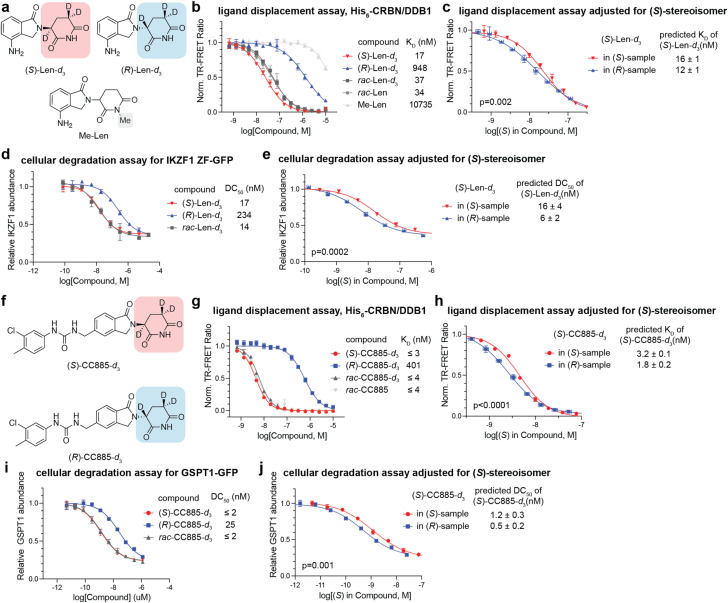
Both enantiomers of lenalidomide and CC885 bind CRBN and promote target degradation in cells. (a) Structures of deuterated lenalidomide enantiomers and a methyl lenalidomide (Me-Len) control. (b) Dose-titration of the indicated compounds in TR-FRET ligand displacement assays and the determined *K*_D_ values of the indicated compounds against His_6_-CRBN/DDB1. Data are presented as mean ± SD (*n* = 3 technical replicates). (c) Adjusted TR-FRET dose-titration curves based on the concentration of the (*S*)-enantiomer formed *in situ* in (*S*)- and (*R*)-Len-*d*_3_ samples. (d) Degradation of IKZF1 ZF2-GFP in HEK293T cells by Len-*d*_3_ enantiomers across a 76 pM–20 μM dose range over 4 h, followed by flow cytometry to assess GFP levels. Data shown are the means ± SD (*n* = 3 biological replicates). (e) Adjusted IKZF1 ZF degradation dose–response curves based on the concentration of the (*S*)-enantiomer formed *in situ* in (*S*)- and (*R*)-Len-*d*_3_ samples. (f) Structures of deuterated CC885 enantiomers. (g) Analysis as in (b) for the indicated compounds. (h) Analysis as in (c) for the indicated compounds. (i) Degradation of GSPT1-GFP in HEK293T cells by CC885-*d*_3_ enantiomers across a 4.8 pM–1.25 μM dose range over 3 h, followed by flow cytometry to assess GFP levels. Data shown are the means ± SD (*n* = 3 biological replicates). (j) Adjusted GSPT1 degradation dose–response curves based on the concentration of the (*S*)-enantiomer formed *in situ* in (*S*)- and (*R*)-CC885-*d*_3_ samples. For (c, e, h and j), the horizontal error bars were calculated based on the 95% confidence intervals of the racemization curves in Fig. S6b.† Statistically meaningful differences between the curves were assessed using an extra sum-of-squares *F*-test. Detailed descriptions of this correction method and its application are provided in Fig. S8.†

As the thalidomide-binding domain of CRBN appears to accommodate both stereoisomers of pLen, we next examined the potential contribution of the stereoisomers on CRBN-dependent activity. To minimize racemization and extend the half-life of each enantiomer for cellular experiments, we generated deuterated lenalidomide (Len-*d*_3_) by hydrogen–deuterium exchange at the three alpha-protons of the glutarimide carbonyl, including the chiral center (Fig. 3a).^41^ Given that deprotonation at the stereocenter is the rate-determining step of keto–enol equilibration, substitution of hydrogen with deuterium at this position should decrease the racemization rate due to the kinetic isotopic effect. Racemization of the deuterated lenalidomide analogs was then monitored in RPMI media over 48 h at 37 °C to measure the racemization rate constant *K*_rac_ and obtain the extrapolated racemization curves (Fig. S6a and b†). As expected, deuteration stabilized the chiral center of deuterated lenalidomide analogs compared to non-deuterated lenalidomide (*t*_50%ee_ Len-*d*_3_ = 24–25 h, *t*_50%ee_ Len = 4.2–4.3 h). To account for the effect of racemization over time in subsequent assays, the experimental data obtained were fitted to the following rate equation for racemization:*Y* = ½(1 − e^−2*K*_rac_*t*^)where *Y* represents the ratio of the other enantiomer formed *via* racemization with constraints of Top = 1 (100% conversion) and Bottom = 0 (0% conversion), *K*_rac_ is the rate constant for the racemization, and *t* is incubation time. Due to symmetry, rate constants are assumed to be equal in both directions. In the analysis of the curve-fitting model, *K*_rac_ was estimated using nonlinear least squares regression, and a 95% confidence interval for the extrapolated racemization curves was determined using the t-distribution (Fig. S6b†).

Based on the obtained racemization curves, we sought to estimate the enantiomeric composition of each sample over time to ascertain the contribution of the (*R*)-enantiomer to the overall binding affinity towards CRBN. The primary motivation for this correction was to account for the contribution of the (*S*)-enantiomer generated *via* racemization in each sample, enabling an accurate assessment of whether the (*R*)-enantiomer independently contributes to CRBN binding. Without this correction, the apparent binding affinity and degradation potency of the (*R*)-enantiomer samples could be confounded by the accumulation of (*S*)-enantiomer, as even a minor fraction of the (*S*)-enantiomer—given its tight binding—can significantly influence the observed readouts.

To quantitatively evaluate binary complex formation, we measured the equilibrium dissociation constant (*K*_D_) of each enantiomer of lenalidomide and deuterated analogs against recombinant His_6_-CRBN/DDB1 by a competitive TR-FRET assay (Fig. 3b, S7a–f and Table S1†). As expected, CRBN exhibited the tightest binding for the (*S*)-enantiomer, followed by racemic mixtures and the (*R*)-enantiomer, across all analogs measured, with minimal impact from deuteration (*K*_D_*rac*-Len = 34 nM; *K*_D_*rac*-Len-*d*_3_ = 37 nM). To correct for the contribution of racemization-derived (*S*)-enantiomer in the (*R*)-enantiomer samples, we first estimated the average fraction of the (*S*)-enantiomer present during the assay window by integrating the area under the racemization curve over time and dividing it by the assay duration. We then adjusted the (*R*)-enantiomer dose–response curves under the null hypothesis that only the (*S*)-enantiomer drives CRBN binding and activity, applying a log transformation based on the estimated (*S*)-enantiomer fraction. This adjustment predicts the expected response if the (*R*)-enantiomer has no independent activity. We also accounted for the *in situ* formation of the (*R*)-enantiomer in the (*S*)-enantiomer sample, although this adjustment fell within the error margins of the original dose–response curve. To assess whether the (*R*)-enantiomer exhibits independent CRBN engagement, we statistically compared the two corrected dose–response curves using an extra sum-of-squares *F*-test. A detailed description of this correction method and its application are provided in the ESI (Fig. S8).†

Following this correction, the adjusted TR-FRET dose-titration curves for (*S*)- and (*R*)-Len-*d*_3_ revealed a statistically meaningful difference (Fig. 3c, *p*-value = 0.002). The curve corresponding to the racemization-derived (*S*)-enantiomer present in the (*R*)-Len-*d*_3_ sample exhibited greater CRBN binding affinity relative to the native (*S*)-Len-*d*_3_ (*K*_D_ (*S*) in (*S*)-Len-*d*_3_ = 16 ± 1 nM; *K*_D_ (*S*) in (*R*)-Len-*d*_3_ = 12 ± 1 nM). Under the null hypothesis that the observed binding affinity of the (*R*)-Len-*d*_3_ sample arises solely from the (*S*)-enantiomer formed *in situ via* racemization, the *K*_D_ values of these two curves would be expected to converge within the error. The observed discrepancy between the two curves led to rejection of the null hypothesis, thereby supporting independent CRBN engagement by the (*R*)-enantiomer. Analysis of the binary complex between lenalidomide and its deuterated derivatives for the thalidomide-binding domain alone revealed a similar result (Fig. S7g–i†). These observations indicate that the CRBN engagement by Len-*d*_3_ was partly augmented by (*R*)-enantiomer.

We next evaluated whether the *in vitro* CRBN engagement by (*R*)-Len-*d*_3_ translated to cellular CRBN engagement and degradation outcomes. HEK293T cells stably expressing IKZF1 zinc finger 2 fused to GFP (IKZF1 ZF2-GFP) were treated with Len-*d*_3_ enantiomers in a full dose–response (Fig. 3d). Adjustment of degradation curves for (*S*)- and (*R*)-Len-*d*_3_, based on the change in concentration of the (*S*)-enantiomer over the incubation period, again suggested the contribution of (*R*)-Len-*d*_3_ to the overall IKZF1 ZF degradation (*p*-value = 0.0002, DC_50_ (*S*) in (*S*)-Len-*d*_3_ = 16 ± 4 nM; DC_50_ (*S*) in (*R*)-Len-*d*_3_ = 6 ± 2 nM, Fig. 3e). A similar analysis for (*S*)- and *rac*-Len-*d*_3_ also demonstrated that (*R*)-enantiomer can still bind to CRBN and enhance cellular target degradation, even when (*S*)-enantiomer is present in equal amounts in a sample (*p*-value = 0.002, DC_50_ (*S*) in *rac*-Len-*d*_3_ = 7 ± 3 nM, Fig. S6j†). Taken together, these results suggest that (*R*)-lenalidomide can augment the degradation of GFP-IKZF1 ZF2 in cells.

To extend these observations, we evaluated the potent degrader of GSPT1, CC885 (*rac*-CC885), and its deuterated isoforms [(*S*)-CC885-*d*_3_, (*R*)-CC885-*d*_3_, and *rac*-CC885-*d*_3_] (Fig. 3f).^42^ As before, (*S*)-CC885-*d*_3_ and *rac*-CC885-*d*_3_ possess low nM *K*_D_ values that were more potent than (*R*)-CC885-*d*_3_ by TR-FRET (Fig. 3g and Table S1†). Considering the structural consistency of the CRBN-binding motif between lenalidomide and CC885, we utilized the extrapolated racemization curve for deuterated lenalidomide to adjust the concentration change of (*S*)-CC885-*d*_3_ in each sample in the following experiments. Adjusted TR-FRET binding curves indicated a partial contribution of (*R*)-CC885-*d*_3_ to the CRBN binding (*p*-value < 0.0001, *K*_D_ (*S*) in (*S*)-CC885-*d*_3_ = 3.2 ± 0.1 nM; *K*_D_ (*S*) in (*R*)-CC885-*d*_3_ = 1.8 ± 0.2 nM, Fig. 3h). Measurement of dose–response of each enantiomer and the racemate of CC885-*d*_3_ in HEK293T cells stably expressing GSPT1-GFP showed a statistically meaningful contribution of (*R*)-CC885-*d*_3_ to deplete GSPT1-GFP levels (*p*-value = 0.001, DC_50_ (*S*) in (*R*)-CC885-*d*_3_ = 0.5 ± 0.2 nM; DC_50_ (*S*) in (*S*)-CC885-*d*_3_ = 1.2 ± 0.3 nM, Fig. 3j), although a statistically significant difference in DC_50_ was not observed between (*S*)- and *rac*-CC885-*d*_3_ (*p*-value = 0.06, DC_50_ (*S*) in *rac*-CC885-*d*_3_ = 0.8 ± 0.2 nM, Fig. S6k†). These results are suggestive that both (*S*)- and (*R*)-CC885-*d*_3_ induce degradation of GSPT1.

To evaluate the biological activity of each enantiomer, we subjected (*S*)-, (*R*)- and *rac*-CC885-*d*_3_ to a PRISM screen against 931 human cancer cell lines, assessing the antiproliferative effects after 24 h (estimated ee = 50%).^43^ Both enantiomers and the racemate of CC885-*d*_3_ displayed similar antiproliferative effects, as indicated by the area under the curve across the cell line panel (Fig. S9a and b†). These results were further validated by MTT assay in a highly sensitive (MOLM13) and a moderately sensitive (BxPC3) cell line (Fig. S9c†). Cell line sensitivity to both enantiomers of CC885 strongly correlated with higher CRBN expression levels but showed no association with GSPT1 expression levels across gene expression, copy numbers, or protein levels (Fig. S9d–f†). However, while these findings suggest contributions from both enantiomers of CC885, they are not conclusive due to the timeframe required to fully capture antiproliferative effects in this cell panel.

### Evaluation of stereoisomers in bifunctional degraders

**Fig. 4:**
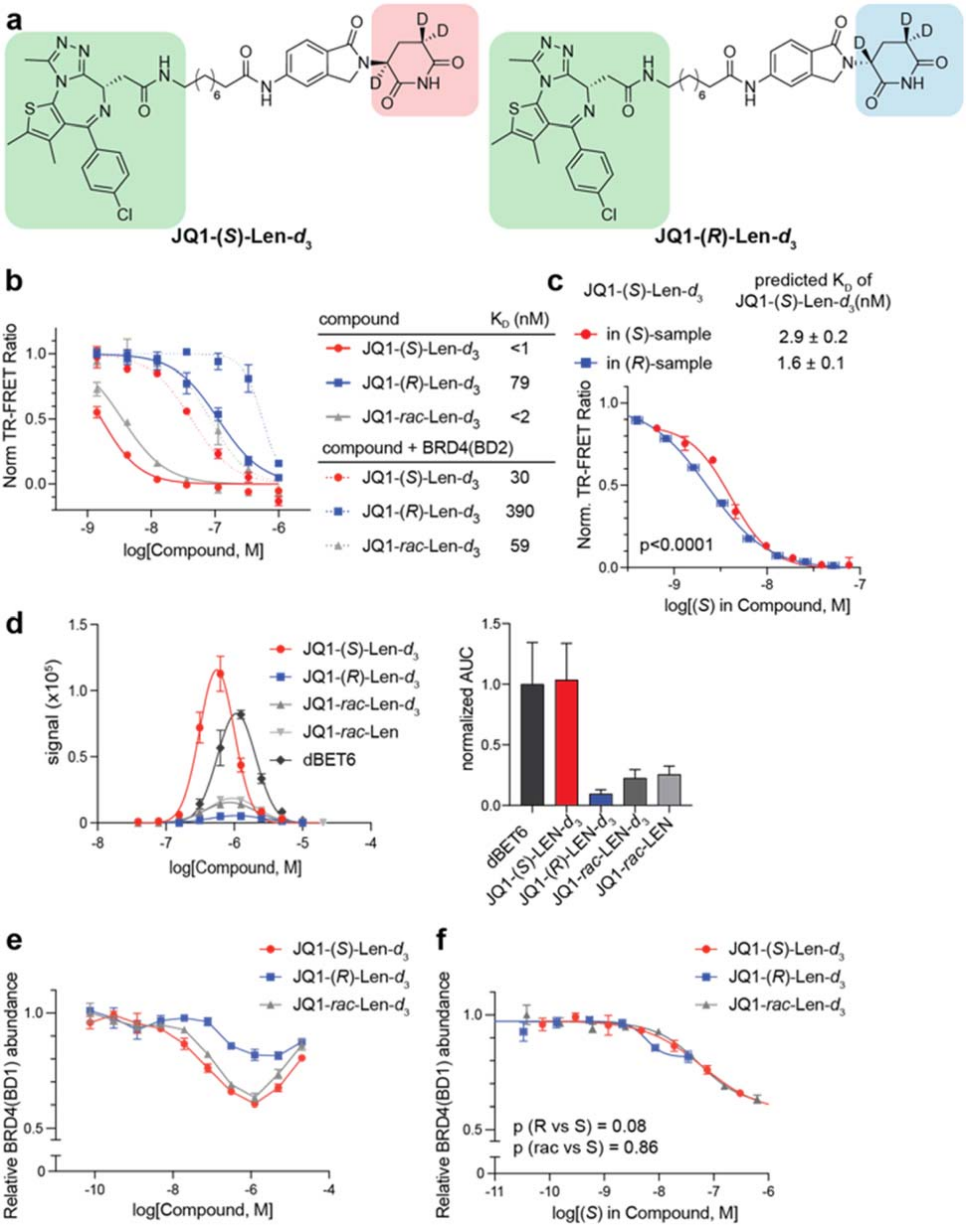
Evaluation of cyclic imide stereoisomers in bifunctional degraders for ternary complex formation with CRBN and BRD4. (a) Structures of JQ1-(*S*)-Len-*d*_3_ and JQ1-(*R*)-Len-*d*_3_. (b) Dose-titration of the indicated compounds in TR-FRET ligand displacement assays with His_6_-CRBN/DDB1 and the determined *K*_D_ values of the indicated compounds against His_6_-CRBN/DDB1 complex with or without BRD4(BD2) measured by TR-FRET assay. (c) Adjusted TR-FRET dose-titration curves based on the concentration of the (*S*)-enantiomer formed *in situ* in JQ1-(*S*)- and (*R*)-Len-d_3_ samples. (d) Ternary complex formation between His_6_-CRBN/DDB1 and BRD4(BD2) mediated by the indicated compound, measured by AlphaScreen assay. (e) Degradation of BRD4(BD1) in cells by JQ1-Len-*d*_3_ enantiomers over a 76 pM–20 μM dose range. HEK293T cells stably expressing BRD4(BD1) fused to GFP were treated with JQ1-Len-*d*_3_ enantiomers for 4 h, followed by flow cytometry to assess BRD4(BD1) degradation. Data shown are the means ± SD (*n* = 3 biological replicates). (f) Adjusted BRD4(BD1) degradation dose–response curves based on the concentration of the (*S*)-enantiomer formed *in situ* in the respective JQ1-(*S*)- and (*R*)-Len-*d*_3_ samples. For (c and f), the horizontal error bars were calculated based on the 95% confidence intervals of the racemization curves in Fig. S6b.† Statistically meaningful differences between the curves were assessed using an extra sum-of-squares *F*-test. Detailed descriptions of this correction method and its application are provided in Fig. S8.†

We additionally investigated if the (*R*)-epimer of a cyclic imide ligand contributes to the activity of a bifunctional degrader by developing a deuterated lenalidomide-based BRD4 degrader conjugated to JQ1 (JQ1-Len-*d*_3_, Fig. 4a). As expected, the (*S*)-epimer and racemate were tighter binders to CRBN than the (*R*)-epimer in the presence or absence of BRD4(BD2) by TR-FRET (Fig. 4b and Table S1†). As bifunctional JQ1-Len-*d*_3_ analogs include deuterated lenalidomide as a CRBN ligand, we employed the extrapolated racemization curve for deuterated lenalidomide to adjust the concentration changes of (*S*)-stereoisomer within each sample in subsequent assays. Analysis accounting for racemization over time supported the engagement of (*R*)-epimer with CRBN in a separate TR-FRET displacement assay against His_6_-CRBN/DDB1 (Fig. 4c). All three compounds showed strong negative cooperativity based on the calculated alpha value, which is in agreement with prior measurements for similar bifunctional ligands (Fig. S10a†).^44–46^ However, only JQ1-(*S*)-Len-*d*_3_ promoted a significant ternary complex formation between His_6_-CRBN/DDB1 and GST-BRD4(BD2) by AlphaScreen (Fig. 4d). The ternary complex formation by JQ1-*rac*-Len-*d*_3_ was additionally much less than JQ1-(*S*)-Len-*d*_3_, presumably due to independent engagement of BRD4(BD2) and competitive inhibition of the ternary complex by JQ1-(*R*)-Len-*d*_3_.

Dose–response degradation assay of BRD4(BD1) in HEK293T cells stably expressing GFP-fused BRD4(BD1) revealed that JQ1-(*S*)-Len-*d*_3_ depleted BRD4 in a dose-dependent manner, while JQ1-(*R*)-Len-*d*_3_ exhibited limited activity (Fig. 4e). Adjustment of degradation curves for each epimer here indicated that the (*R*)-epimer in the bifunctional ligand does not appear to contribute to the overall degradation activity, in contrast to previous observations with the monofunctional (*R*)-Len-*d*_3_ (Fig. 4f). The differential degradation efficiency was further confirmed by global quantitative proteomics, where BRD4 was the most significantly downregulated protein in the JQ1-(*S*)-Len-*d*_3_-treated sample, but BRD4 protein levels were unperturbed in the JQ1-(*R*)-Len-*d*_3_-treated sample after 4 h (Fig. S10b and Tables S6–9†). Taken together, these data suggest that in the context of bifunctional ligands for BRD4, the (*S*)-epimer forms a productive ternary complex while the (*R*)-epimer does not.

### CRBN recognition of the cyclic imide degron stereoisomers

While most CRBN ligands are based on glutarimides, CRBN can recognize both the C-terminal aspartimide and glutarimide as degrons.^21^ Although the relative rate of racemization of the C-terminal cyclic imide is likely slower than thalidomide and lenalidomide, Asn/Asp and Gln/Glu are more prone to racemization due to the formation of these cyclic intermediates.^22^ We, therefore, evaluated the racemization kinetics of the C-terminal cyclic imides and characterized the effect of stereoisomers of the C-terminal cyclic imide degron on CRBN-dependent clearance of protein substrates.

**Fig. 5:**
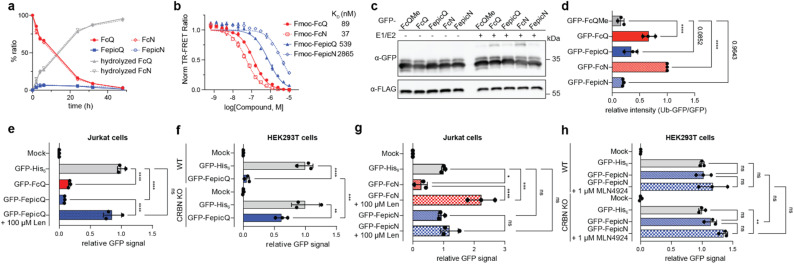
Recognition of the C-terminal cyclic imide degron stereoisomers by CRBN. (a) Racemization study of Fmoc-GGGFcQ/FepicQ/FcN/FepicN in 0.1 M phosphate buffer (pH = 7.4) at 37 °C. (b) *K*_D_ values of Fmoc-GGGFcQ/FepicQ/FcN/FepicN against His_6_-CRBN/DDB1 complex, measured by TR-FRET assay. (c) Western blot analysis of *in vitro* ubiquitination of engineered GFPs carrying the indicated C-terminal modification, mediated by the CRL4^CRBN^ complex. (d) Relative quantification of Ub-GFP/GFP ratio from three biological replicates in (c). (e and f) Levels of engineered GFP with a C-terminal cyclic glutarimide degron in (e) Jurkat cells with or without lenalidomide (100 μM) and (f) HEK293T WT or CRBN KO cells after 6 h. (g and h) Levels of engineered GFP with a C-terminal cyclic aspartimide degron in (g) Jurkat cells with or without lenalidomide (100 μM) and (h) HEK293T WT or CRBN KO cells with or without MLN4924 after 6 h. Statistical significance was analyzed using a one-way ANOVA with Šídák's multiple comparisons test. ns = not significant, * = *p* < 0.05, ** = *p* < 0.01, *** = *p* < 0.001, **** = *p* < 0.0001.

To measure the racemization rates of epimers of the C-terminal cyclic imide degron, we incubated pentapeptides bearing the (*S*)- and (*R*)-epimer of cyclic glutamine (Fmoc-GGGFcQ and Fmoc-GGGFepicQ) or cyclic asparagine (Fmoc-GGGFcN and Fmoc-GGGFepicN) in 0.1 M phosphate buffer (pH 7.4) at 37 °C (Fig. 5a and S11†). Hydrolysis of the C-terminal cyclic imide proceeded faster than racemization, which aligns with the reported kinetics of hydrolysis *versus* racemization for internal cyclic imide intermediates arising from Asn deamidation.^23^ Despite this observation, racemization still occurs across all four peptides at similar rates (77.6–82.5% ee at 6 h, 45.0–49.6% ee at 24 h). Although racemization kinetics may be affected by physiological environments, neighboring amino acid residues, or the protein context, these results suggest that C-terminal cyclic imides undergo only modest racemization under non-enzymatic conditions and at a slower rate than lenalidomide. This observation somewhat aligns with the predicted p*K*_a_ values of the cyclic imide stereocenter (FcQ: 17.21, FcN: 16.15, lenalidomide: 16.17).^33–36^ The lower p*K*_a_ of FcN may result from its smaller ring size (5-membered *vs.* 6-membered in FcQ), which increases strain and could destabilize the conjugate base.

We next measured the dissociation constant of the four pentapeptides against His_6_-CRBN/DDB1 by TR-FRET (Fig. 5b and Table S1†). Fmoc-GGGFcN and Fmoc-GGGFcQ showed strong affinity (*K*_D_ = 37 and 89 nM, respectively), while the epimers are moderate to weak CRBN ligands (*K*_D_ Fmoc-GGGFepicQ = 539 nM, *K*_D_ Fmoc-GGGFepicN = 2.87 μM). Installation of these peptides to GFP using a sortase system to yield GFP-FcQ, GFP-FepicQ, GFP-FcN, and GFP-FepicN revealed similar binding trends, albeit at overall lower absolute affinities (Fig. S12†).^21^ This trend in dissociation constants was maintained in the context of bifunctional degraders, where degrons were linked to JQ1.^21^ The ligand with the highest affinity was JQ1-FcN, followed by JQ1-FcQ, JQ1-FepicQ, and then JQ1-FepicN (Fig. S13a and b†). Consistent with our examination of bifunctional degraders with deuterated lenalidomide stereoisomers, JQ1-FepicQ and JQ1-FepicN minimally promoted the ternary complex with GST-BRD4(BD2) by AlphaScreen (Fig. S13c†), which corresponds to the limited degradation potency of JQ1-FepicQ previously observed in cells.^21^ These data indicate that degron recognition is strongest for the natural isoform of aspartimide, followed by glutarimide, with this trend consistent across peptides, proteins, and small molecule ligands.

The C-terminal cyclic imide epimers were evaluated as substrates for CRBN by measurement of *in vitro* ubiquitination and cellular degradation of the engineered GFPs (Fig. 5c). The monoubiquitination levels of GFP-FcQ, GFP-FepicQ, GFP-FcN and GFP-FepicN with K0-ubiquitin were 4.8, 2.4, 7.1 and 1.3-fold greater than methylated GFP-FcQ (GFP-FcQMe), respectively, although the fold change of GFP-FepicN did not reach statistical significance (Fig. 5d). Strikingly, GFP-FcQ and GFP-FepicQ were degraded in a CRBN-dependent manner after electroporation and incubation for 6 h in Jurkat cells (expected ee = 78–83%, Fig. 5e and f). GFP-FcN was likewise efficiently degraded in a CRBN-dependent manner and competed by lenalidomide in cells,^21^ but GFP-FepicN was not degraded in cells in a manner that was dependent on CRBN or affected by MLN4924, a neddylation inhibitor of Cullin-RING E3 ligases like CRL4^CRBN^ (Fig. 5g and h). The lack of functional engagement of GFP-FepicN by CRBN in cells corresponds with the limited signal measured by TR-FRET and *in vitro* ubiquitination assays. We further examined another set of degron epimers, AcN and AepicN (Fig. S14a†). While GFP-AcN underwent clear degradation, which was rescued by lenalidomide competition, no degradation was observed for GFP-AepicN, aligning with the data with GFP-FcN *versus* GFP-FepicN. TR-FRET assays with pentapeptides bearing either stereoisomer of AcN revealed a >30-fold difference in CRBN binding affinity (Fig. S14b and Table S1†), confirming that GFP-AepicN does not engage CRBN tightly enough for recognition and degradation. Collectively, these data indicate that CRBN can recognize and remove both epimers of the C-terminal cyclic imide on proteins if the epimer engages CRBN within a certain dissociation constant.

## Conclusions

In this study, we systematically characterized how the thalidomide-binding domain of CRBN recognizes multiple classes of cyclic imide ligands and their stereoisomers. Our work provides a comprehensive examination of 12 stereoisomeric pairs (24 stereopure ligands) alongside three racemic samples, spanning molecular glues, PROTACs, and cyclimid peptides and proteins. These compounds were analysed through structural proteomics, *in vitro* racemization and binding assays, and cellular experiments. Structural proteomics and molecular modeling with i-pLen probes revealed that each enantiomer of lenalidomide adopts a more distinct binding mode than previously suggested by structural studies with thalidomide enantiomers.^17^ As expected, CRBN consistently exhibited a pronounced preference for the (*S*)-stereoisomer across monofunctional, bifunctional, and peptide ligands, although the (*R*)-stereoisomer could engage at submicromolar concentrations by *in vitro* TR-FRET displacement assays. Interestingly, our data demonstrate that each class of cyclic imide ligands shows varying degrees of contribution from the (*R*)-stereoisomer to ternary complex formation and target protein degradation in cells, irrespective of CRBN binding affinity measured by TR-FRET displacement assays. This observation further suggests that each stereoisomer adopts a distinct binding mode, which may or may not result in productive ternary complex formation.

Despite substantial interest in studying the impact of cyclic imide ligand stereoisomers for CRBN, their susceptibility to rapid racemization poses a significant challenge to fully control these experiments. The cyclic imide chiral center is relatively stable in peptides, followed by lenalidomide and its derivatives, and most prone to racemization in thalidomide. While deuteration of the chiral center and short experimental timeframes (30 min–1 h) at 24 °C can minimize racemization (<5% racemization in non-deuterated analogs; no detectable racemization in deuterated analogs), controlling racemization in cellular experiments is inherently more challenging. Consequently, the contribution of racemization to cellular contexts remains difficult to fully eliminate, making its precise impact challenging to determine.

Nevertheless, in an effort to assess contributions of individual stereoisomers, we monitored the racemization of deuterated lenalidomide stereoisomers *in vitro* to determine the racemization rate constant *K*_rac_ and obtain the extrapolated racemization curves. These extrapolated racemization curves allowed us to estimate the concentration changes of the (*S*)-stereoisomer present within each sample over the course of each biochemical experiment, enabling a careful evaluation of the (*R*)-stereoisomer's contribution. This analysis revealed that, in the context of monofunctional ligands, the (*R*)-stereoisomer binds to CRBN and contributes to degradation. In contrast, when embedded in bifunctional ligands, the (*R*)-stereoisomer exhibits no statistically meaningful contribution to degradation. Thus, while the predominant biological activity stems from the (*S*)-stereoisomer, the (*R*)-stereoisomer appears to play some role in the overall biological effect. It should be noted that we cannot fully exclude the possibility that each class of ligands exhibits different racemization properties and CC885 and JQ1-Len are assumed to display similar racemization kinetics to lenalidomide based on their structural similarities.

Finally, these data have intriguing implications for the physiological function of CRBN in recognizing the C-terminal aspartimide or glutarimide modification^21^ that may be relevant to the evolutionary development of the thalidomide-binding domain.^19,20,47^ While the sources of C-terminal cyclic imide in mammalian cells are still under investigation, their non-enzymatic formation is analogous to spontaneous deamidation observed in protein aging, where an internal cyclic imide intermediate forms prior to hydrolysis.^23,48–50^ Our studies with Fmoc-GGGFcQ and Fmoc-GGGFcN show that C-terminal cyclic imides undergo hydrolytic decomposition more rapidly than racemization, suggesting that the contribution of racemization during the typical timeframe of cellular degradation experiments may be minimal. Computational p*K*_a_ predictions further supported these observations, indicating that the cyclic imide chiral center of FcQ is less acidic than that of thalidomide, lenalidomide, and FcN, while FcN and lenalidomide exhibit comparable acidity. This difference may contribute to the slower racemization of C-terminal cyclic imides (FcQ and FcN) relative to lenalidomide and thalidomide, although overall epimerization rates and subsequent CRBN binding are influenced by both stereocenter acidity and the local chemical environment of the specific peptide or protein sequence.

Collectively, these findings robustly demonstrates that CRBN can recognize and mediate degradation of engineered GFPs with C-terminal cyclic imide epimers if they bind within a certain dissociation constant range. Our data further indicate the highest recognition of the (*S*)-stereoisomer of aspartimide in line with prior data comparing aspartimide to glutarimide cyclimids,^46^ yet reveal relatively flexible recognition of both stereoisomers of the glutarimide. These data are in line with previous observations with other ligands for CRBN, such as phenyl glutarimide ligands^51^ or substitution with dihydrouracil,^52,53^ whereas aspartimide-based thalidomide derivatives have reduced binding and degradation activity.^54^ Future studies on endogenous CRBN substrates may strengthen the trends observed here. In sum, these data confirm that the (*S*)-stereoisomer of the cyclic imide is the primary contributor to CRBN-dependent activity, while illustrating instances where the (*R*)-stereoisomer can exert an influence.

## Data availability

The datasets supporting this article have been uploaded as part of the ESI.†

## Author contributions

C. M. W. and Y. A. conceived of the project. Y. A., S. I., N. C. P., and Q. Z. synthesized compounds. Y. A. and Z. L. performed binding site mapping and modeling. B. B. supervised proteomics data acquisition. S. I. and N. C. P. designed and performed the evaluation of the binary and ternary complex engagement with compounds by TR-FRET assay and associated assay validation. R. M. supervised TR-FRET assay validation and development. H. C. L. prepared semi-synthetic GFPs. S. I., Y. A., and H. C. L. performed degradation assays. S. I. and Y. A. performed racemization studies and analyzed biochemical assay data to reflect the effect of the racemization over time. A. S. B., M. G. R., M. M. R., and J. A. R. performed PRISM screens. Y. A. and S. I. drafted the manuscript, and all authors jointly discussed the results and edited the manuscript.

## Conflicts of interest

The authors declare the following competing interests: Harvard University has filed a PCT patent application on April 13, 2022 covering the chemical structures and their use. C. M. W. and S. I. are inventors of this patent. R. M. and N. C. P. are inventors on patent applications related to the CoraFluor TR-FRET probes used in this work. B. B. is on the scientific advisory board of Preverna. All other authors declare no competing interests.

## Supplementary Material

SC-OLF-D5SC01371B-s001

SC-OLF-D5SC01371B-s002

SC-OLF-D5SC01371B-s003

SC-OLF-D5SC01371B-s004
