# Supplementary material for: The contribution of cyclic imide stereoisomers on cereblon-dependent activity
Source: Chem Sci. 2025 May 28;16(25):11519–29. doi: 10.1039/d5sc01371b (PMC12117711; doi:10.1039/d5sc01371b)
Supplement: SC-016-D5SC01371B-s001 [file SC-016-D5SC01371B-s001.pdf]

Supplementary Information (SI) for Chemical Science.
This journal is © The Royal Society of Chemistry 2025

Supporting Information

The contribution of cyclic imide stereoisomers on cereblon-dependent activity

Yuka Amako,[1,†] Saki Ichikawa,[1,†] Hannah C. Lloyd,[1] N Connor Payne,[1,2] Zhi Lin,[1] Andrew S. Boghossian,[3] Matthew G. Rees,[3] Melissa M. Ronan,[3] Jennifer A. Roth,[3] Qian Zhu,[1] Bogdan Budnik,[4] Ralph Mazitschek,[2,3,5] Christina M. Woo[1,3,*]

[1] Department of Chemistry and Chemical Biology, Harvard University, Cambridge, MA 02138 USA

[2] Center for Systems Biology, Massachusetts General Hospital, Boston, MA 02114 USA

[3] Broad Institute of MIT and Harvard, Cambridge, MA 02142, USA

[4] Mass Spectrometry and Proteomics Resource Lab (MSPRL), Division of Science, Faculty of Arts and Sciences, Harvard University, Cambridge, MA 02138, USA

[5] Harvard T.H. Chan School of Public Health, Boston, MA, 02115 USA

* Email: cwoo@chemistry.harvard.edu

Index

Supplemental Figures	S2
Materials and Methods	S19
General Procedures	S22
Synthetic Procedures	S32
References	S48
NMR and IR Spectra	S49

Other Supplementary Materials for this manuscript include the following:

Supplementary Table for TR-FRET assay (Table S1, Excel)
Supplementary Table for Structural Proteomics (Table S2–S5, Excel)
Supplementary Table for Global Quantitative Proteomics (Table S6–S9, Excel)

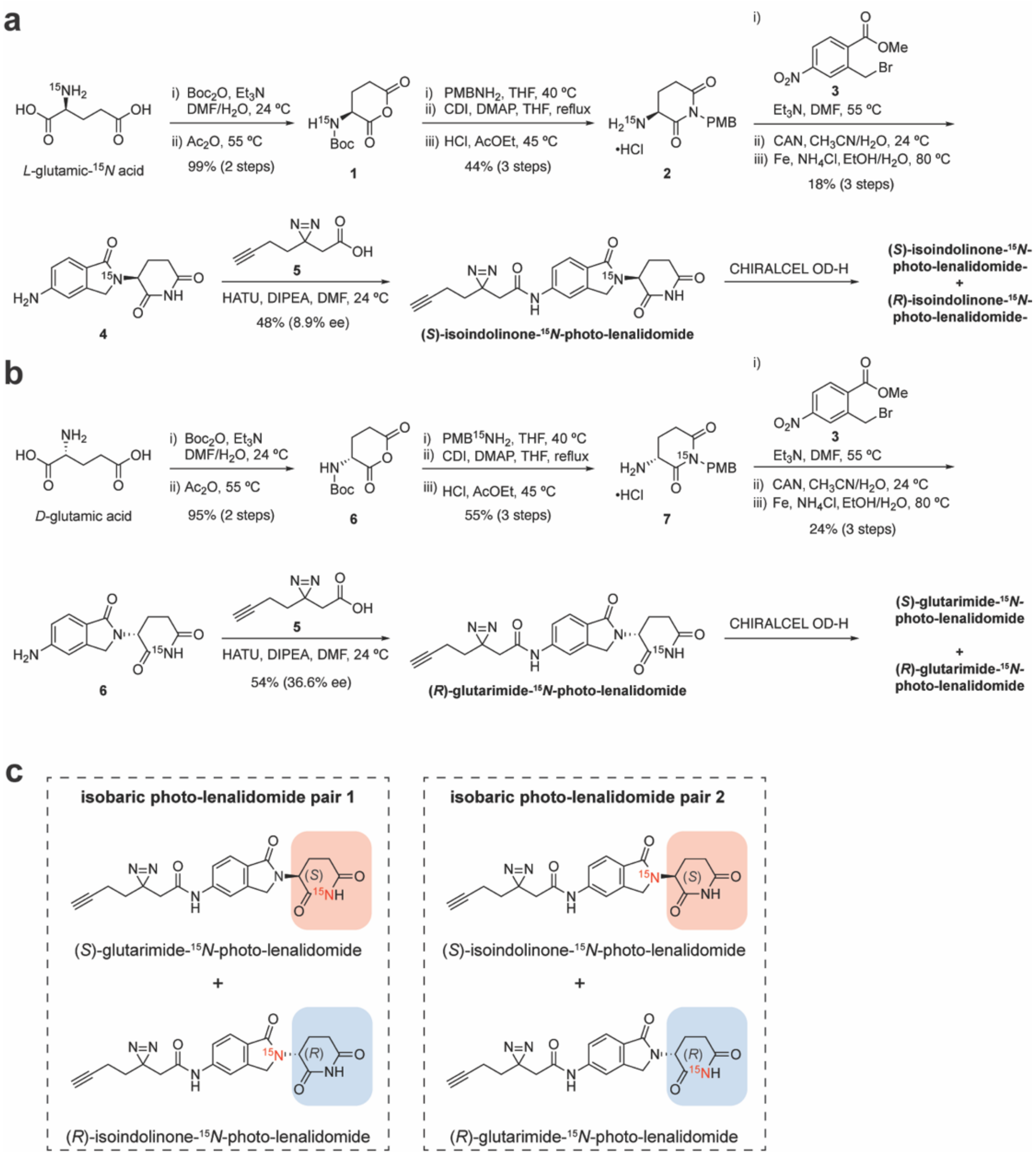

Fig. S1. Synthesis of isobaric photo-lenalidomide. (a, b) Synthetic scheme of isoindolinone-^{15}N-photo-lenalidomide **(a)** glutarimide-^{15}N-photo-lenalidomide **(b)**. **(c)** Structures of each set of isobaric photo-lenalidomide probes.

a

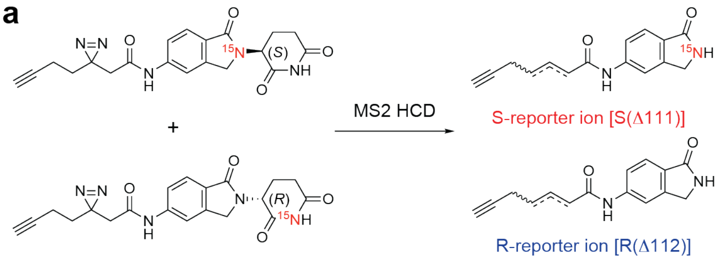

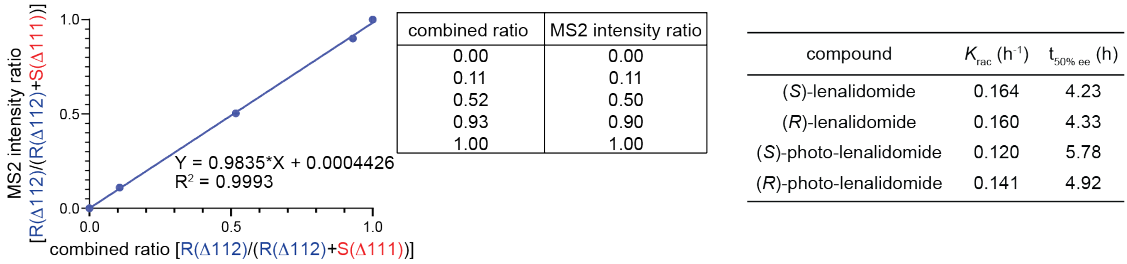

combined ratio	MS2 intensity ratio
0.00	0.00
0.11	0.11
0.52	0.50
0.93	0.90
1.00	1.00

b

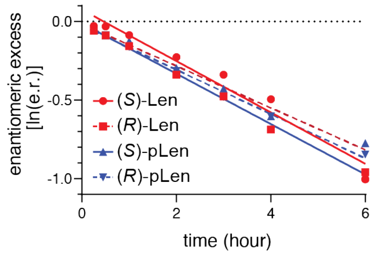

compound	K_{rac} (h^{-1})	$t_{50\% ee}$ (h)
(*S*)-lenalidomide	0.164	4.23
(*R*)-lenalidomide	0.160	4.33
(*S*)-photo-lenalidomide	0.120	5.78
(*R*)-photo-lenalidomide	0.141	4.92

Fig. S2. Validation of pLen and i-pLen probes with the thalidomide-binding domain of CRBN. (a) Validation of quantification with i-pLen pair 2 (Fig. S1c) by tandem mass spectrometry. Combined ratio determined by integration of a proton on the glutarimide ring nitrogen from ^{1}H-NMR spectra was compared with MS2 intensity ratio of S- and R-reporter ions. **(b)** Racemization rate of lenalidomide and photo-lenalidomide in RPMI medium at 37 °C with 5% CO_2.

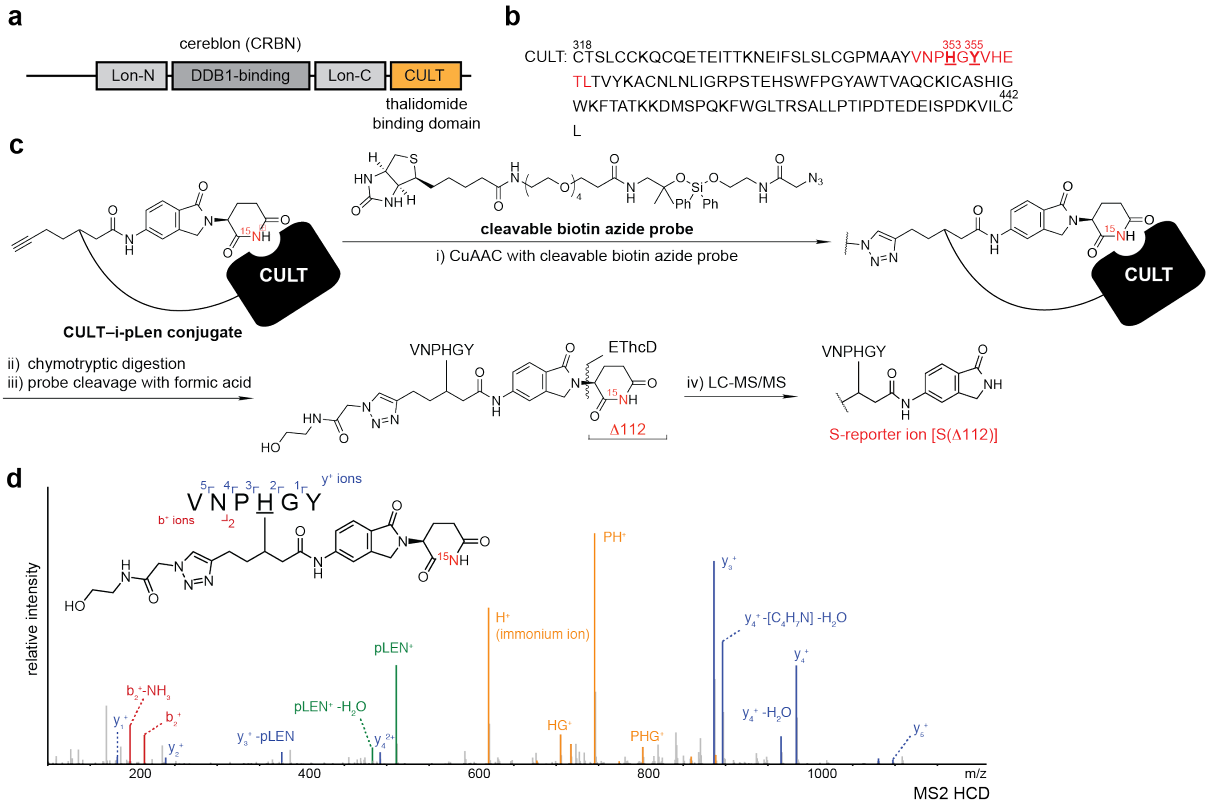

Fig. S3. Supplemental Figures for thalidomide-binding domain labeling by isobaric photo-lenalidomide. (a) Schematic of CRBN. **(b)** Amino acid sequence of the thalidomide-binding domain. The modified peptide characterized by mass spectrometry is colored in red and amino acid residues labeled by (*S*)-photo-lenalidomide (His353) and (*R*)-photo-lenalidomide (Tyr355) are underlined. The residue numbers correspond to those of CRBN**. (c)** Schematic of the PAL labeling approach for thalidomide-binding domain. **(d)** Representative MS2 HCD spectrum assignment of thalidomide-binding domain peptide conjugated to isobaric photo-lenalidomide**.**

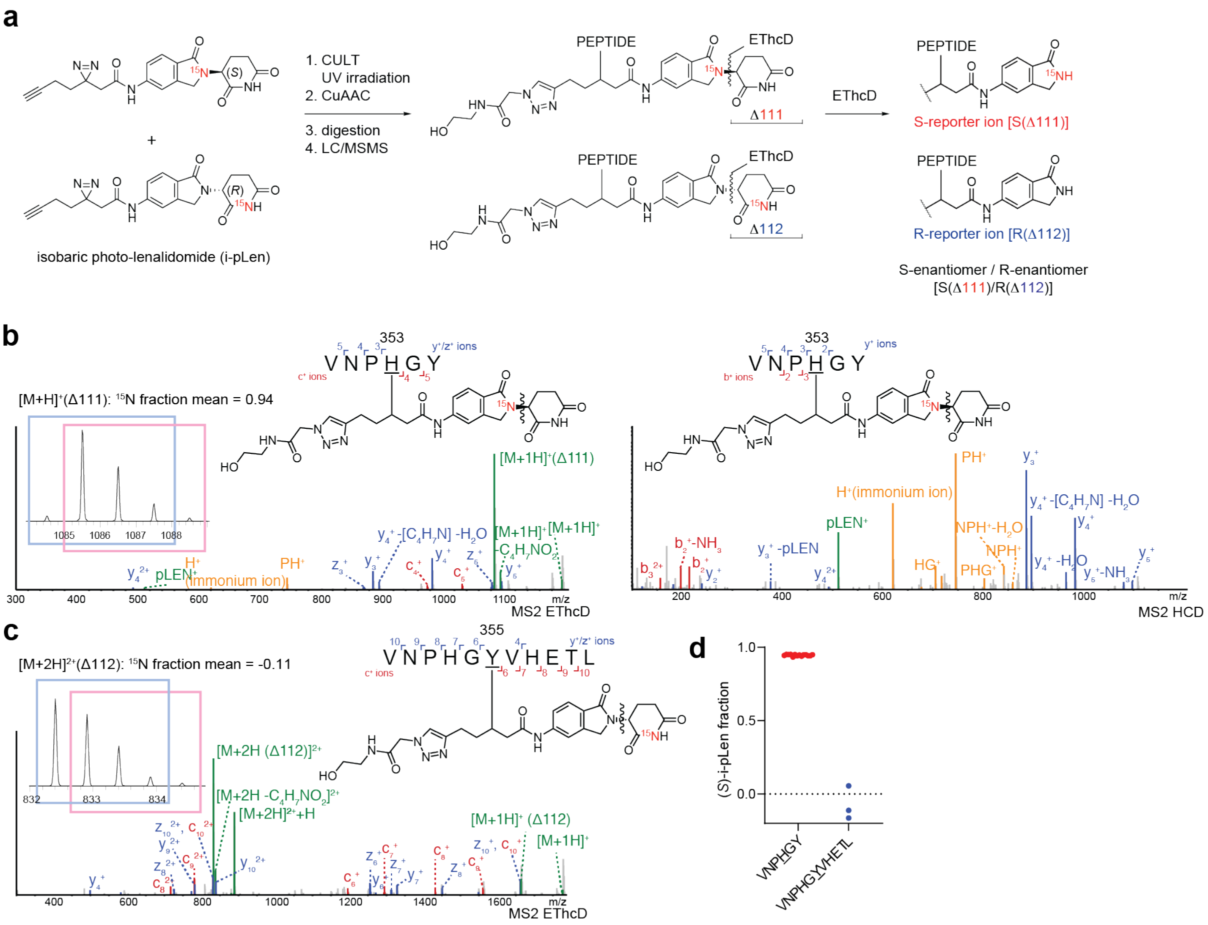

Fig. S4. The thalidomide-binding domain labeling with an analogous set of isobaric photo-lenalidomide. (a) Schematic of the thalidomide-binding domain labeling with an analogous set of isobaric photo-lenalidomide. **(b–c)** Representative assignments of thalidomide-binding domain peptides conjugated to isobaric photo-lenalidomide. **(d)** Enantiomeric ratio at binding sites by spectral counts.

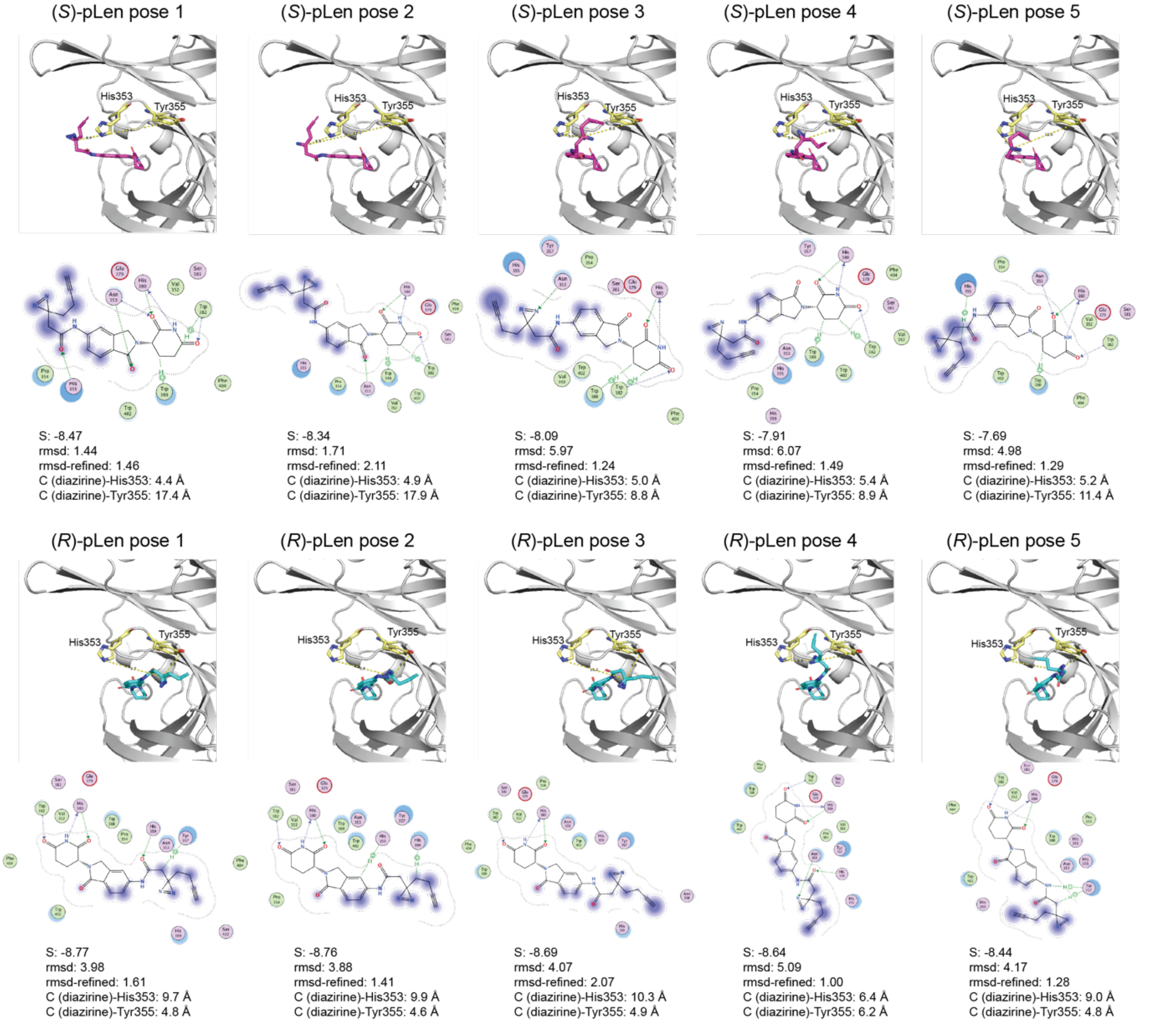

Fig. S5. Comparative analysis of docking poses and key interactions for (*S*)- and (*R*)-pLen. The top five docking poses with the lowest S scores of (*S*)- and (*R*)-pLen, respectively, were generated from the crystal structure of the lenalidomide–CRBN/DDB1 complex (PDB: 4CI2), along with two-dimensional interaction maps highlighting key contacts. Residue number of the three-dimensional structure corresponds to homo sapience CRBN and residue number of the two-dimensional interaction corresponds to gallus gallus CRBN.

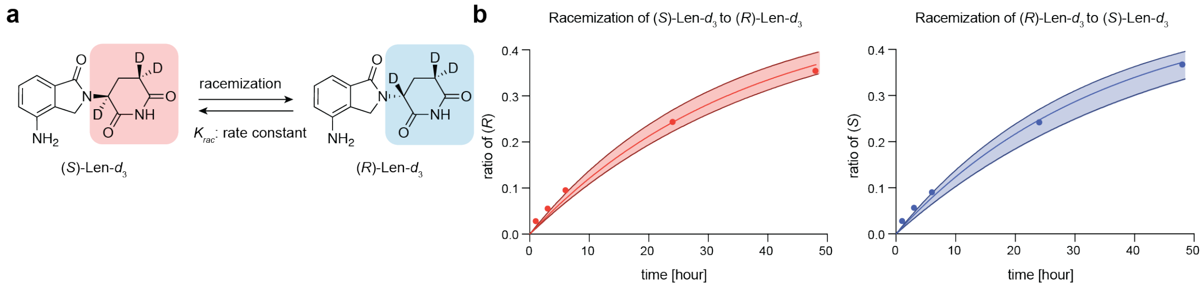

compound	K_{rac} (h^{-1})			$t_{50\% ee}$ (h)		
	fitted	upper	lower	fitted	upper	lower
(*S*)-lenalidomide-d_3	0.0138	0.0162	0.0115	25.0	30.1	21.4
(*R*)-lenalidomide-d_3	0.0142	0.0161	0.0123	24.4	28.2	21.5

Fig. S6. Racemization of deuterated lenalidomide. (a) Schematic of racemization between the deuterated lenalidomide enantiomers (*S*)-Len-d_3 and (*R*)-Len-d_3. **(b)** Racemization of deuterated lenalidomide in RPMI medium at 37 °C with 5% CO_2. K_{rac}: rate constant for the racemization. The ratio of the other enantiomer formed via racemization is shown with constraints of Top = 1 (100% conversion) and Bottom = 0 (0% conversion). A 95% confidence interval for the extrapolated racemization curves is visualized with the shaded area, offering a visual representation of the uncertainty inherent in the model predictions.

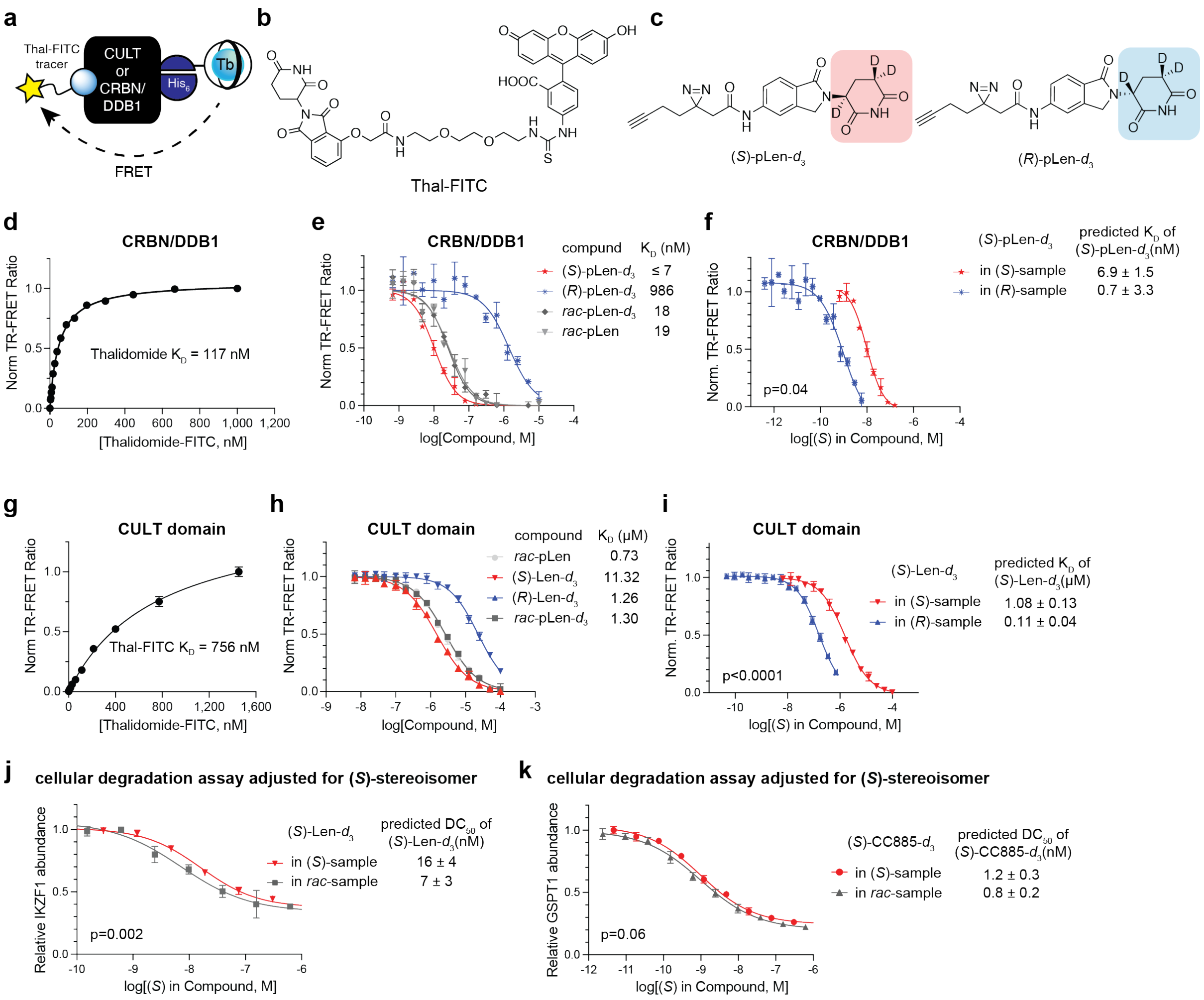

Fig. S7. Supplementary data for Fig. 3. (a) Schematic of TR-FRET assay. TBD, thalidomide-binding domain. **(b)** Structure of the Thal-FITC tracer. **(c)** Structures of deuterated photo-lenalidomide (pLen) enantiomers. **(d)** Saturation binding of Thal-FITC to Tb-labeled His_6-CRBN/DDB1 complex. **(e)** Dose-titration of the indicated compounds in TR-FRET ligand displacement assays with His_6-CRBN/DDB1, with determined K_D values. Data are presented as mean ± SD (n = 3 technical replicates). **(f)** Adjusted TR-FRET dose-titration curves with His_6-CRBN/DDB1, incorporating changes in (*S*)-enantiomer concentration over time in (*S*)- and (*R*)-pLen-d_3 samples. **(g)** Saturation binding of Thal-FITC to Tb-labeled thalidomide-binding domain. **(h)** Dose-titration of the indicated compounds in TR-FRET ligand displacement assays with the thalidomide-binding domain, with determined K_D values. Data are presented as mean ± SD (n = 3 technical replicates). The differential dissociation constant between CRBN and the thalidomide-binding domain is presumably due to the absence of rest of the protein that promotes the stabilization of the thalidomide-binding domain.[1] **(i)** Adjusted TR-FRET dose-titration curves with the thalidomide-binding domain, incorporating (*S*)-enantiomer concentration changes over time in (*S*)- and (*R*)-pLen-d_3 samples. Adjusted **(j)** IKZF1 or **(k)** GSPT1 degradation dose-response curves, accounting for in situ formation of (*S*)-enantiomer in (*S*)- and racemic samples of the indicated compounds. For **(f, i, j, k)**, the horizontal error bars were calculated based on the 95% confidence intervals of the racemization curves in Fig. S6b. Statistically meaningful differences between the curves were assessed using an extra sum-of-squares F-test. Detailed description of this correction method and its application are provided in Fig. S8.

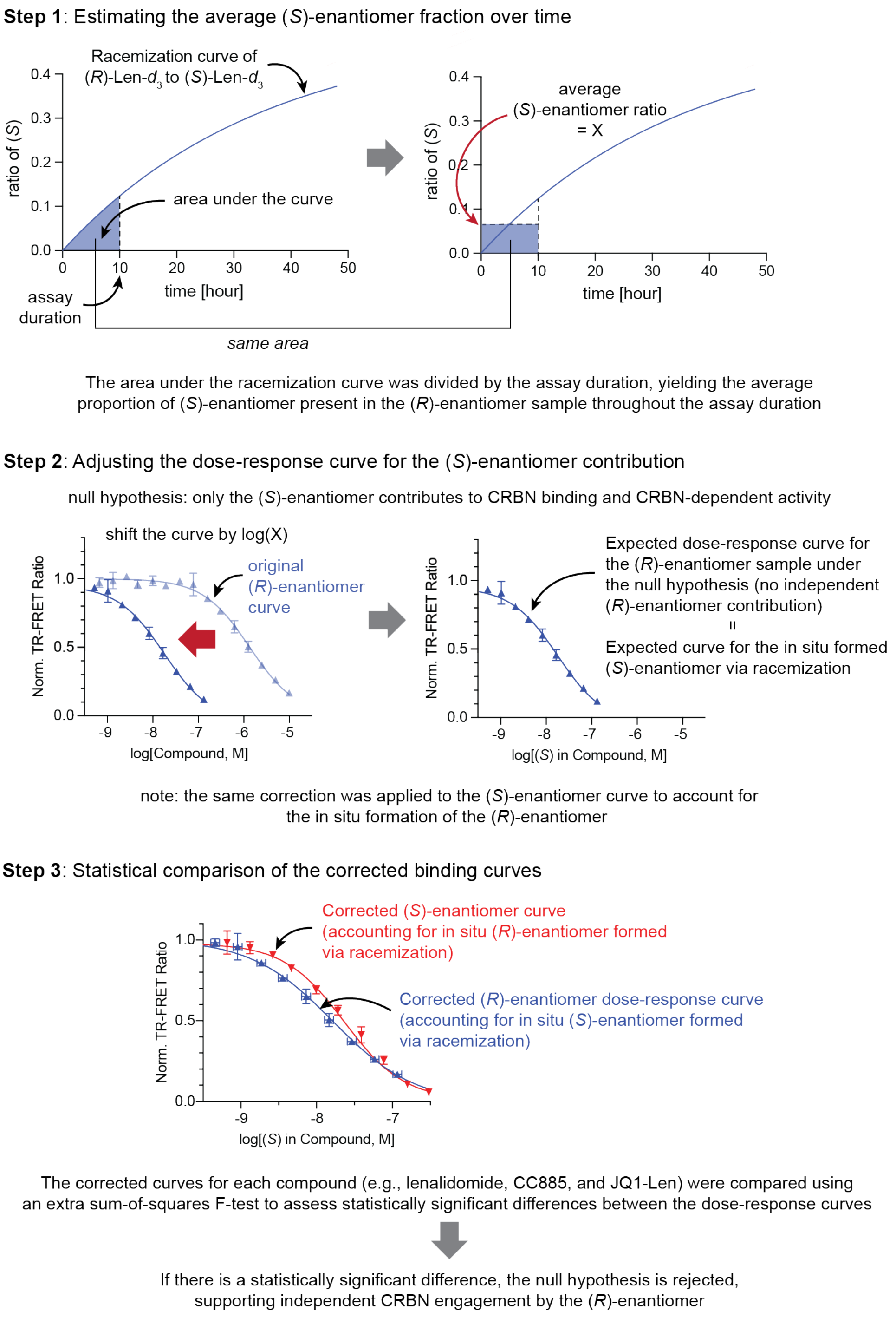

Fig. S8. Schematic of the correction method used in Figures 3 and 4 to account for racemization during the assay time window.

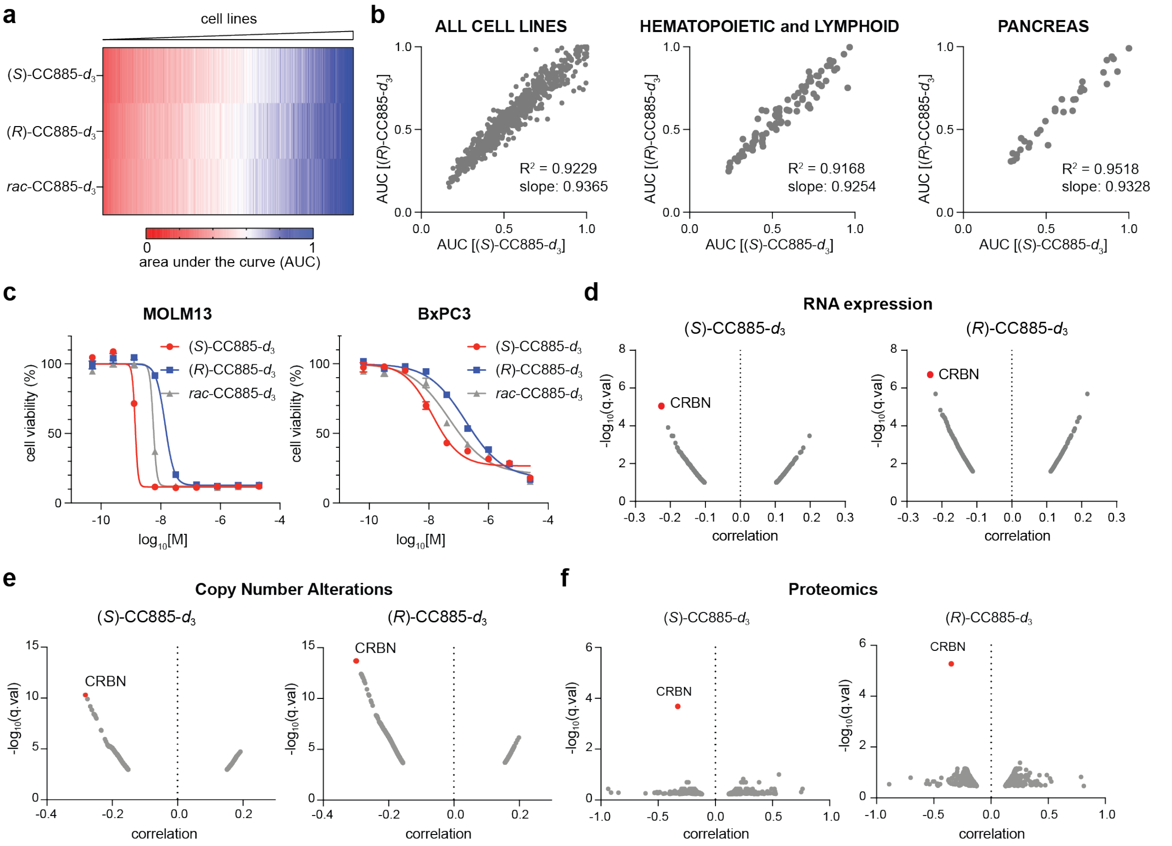

Fig. S9. Supplementary analyses from the PRISM screen of deuterated CC885 enantiomers. **(a)** Heat map of AUC values of dose-response curves of the 931 cell lines treated with the indicated compound for 24 h. **(b)** Linear regression analysis of AUC of (*S*)- and (*R*)-CC885-d_3 for all cell lines (left), hematopoietic and lymphoid cell lines (middle), and pancreas cell lines (right). **(c)** 24-h MTT assay of MOLM13 and BxPC3 cells with the indicated compounds. **(d–f)** Correlation analysis of mRNA expression **(d)**, copy number alteration **(e)**, and protein expression **(f)** with sensitivity to the compound based on an AUC value of a dose-response curve. Negative correlations correspond to increasing protein levels corresponding to increased sensitivity. CRBN showed negative correlation and was ranked first for both (*S*)- and (*R*)-CC885-d_3.

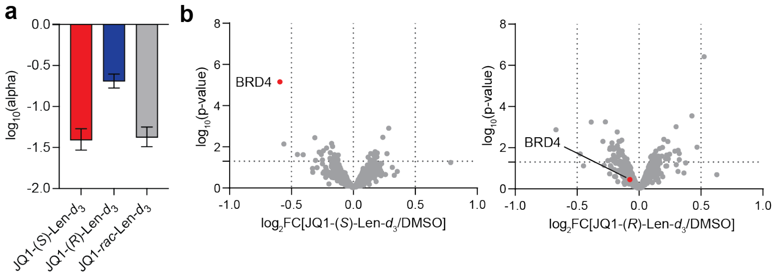

Fig. S10. Supplementary of Fig. 4. (a) Alpha value of the indicated compounds calculated based on K_D values in Fig. 4b. **(b)** Quantitative proteomics of protein levels following treatment of MOLM13 cells with the indicated compound at 100 nM for 4 h (n = 4).

Fmoc-GGG-FcQ

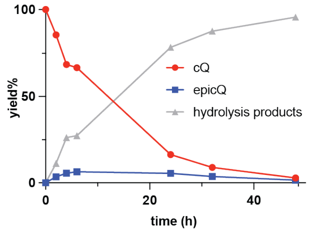

time (h)	yield (%)			de (%)
	cQ	epicQ	hydrolysis products	
0	100.0	0.0	0.0	100.0
2	85.3	3.4	11.2	92.3
4	68.3	5.6	26.1	84.9
6	66.5	6.4	27.2	82.5
24	16.3	5.5	78.2	49.6
32	8.8	3.6	87.5	41.8
48	2.8	1.6	95.6	28.8

Fmoc-GGG-FepicQ

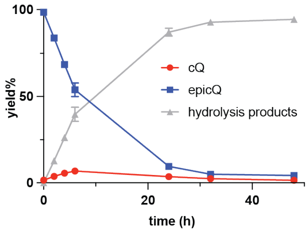

time (h)	yield (%)			de (%)
	cQ	epicQ	hydrolysis products	
0	1.5	98.5	0.0	96.9
2	3.7	83.6	12.7	91.5
4	5.7	68.3	26.1	84.9
6	6.9	53.7	39.5	77.6
24	4.0	9.4	87.0	45.0
32	2.4	4.9	92.7	35.2
48	1.4	4.2	94.4	49.0

Fmoc-GGG-FcN

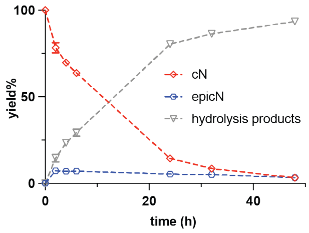

time (h)	yield (%)			de (%)
	cN	epicN	hydrolysis products	
0	100.0	0.0	0.0	100.0
2	78.3	7.2	14.5	83.1
4	69.7	6.9	23.4	81.9
6	63.7	7.0	29.3	80.1
24	14.4	5.3	80.4	46.4
32	8.6	5.1	86.4	25.7
48	3.2	3.8	93.5	-7.8

Fmoc-GGG-FepicN

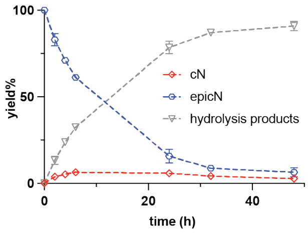

time (h)	yield (%)			de (%)
	cN	epicN	hydrolysis products	
0	0.0	100.0	0.0	100.0
2	3.8	82.9	13.3	91.3
4	5.2	71.0	23.8	86.4
6	6.4	61.2	32.4	81.1
24	5.8	15.7	78.5	45.9
32	4.2	8.7	87.1	34.6
48	2.7	6.4	90.9	40.4

Fig. S11. Racemization of the indicated synthetic peptides in 0.1 M phosphate buffer pH 7.4 at 37 °C.

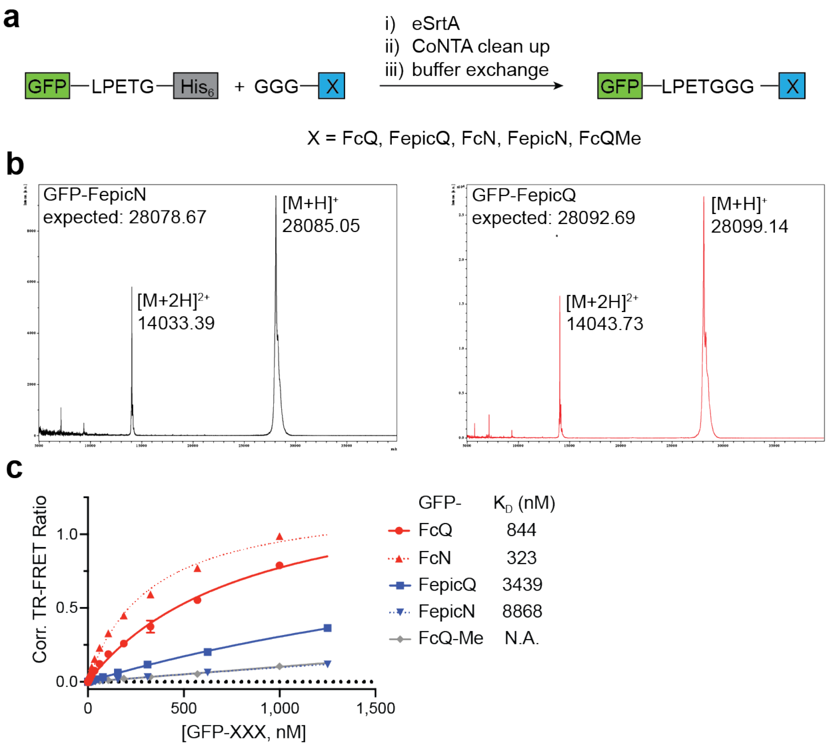

Fig. S12. Supplementary Fig.ures of semi-synthetic GFPs. (a) Schematic of GFP-FcQ, GFP-FcN, GFP-FepicQ and GFP-FepicN formation by sortase reaction. **(b)** Intact mass spectrometry of GFP-FepicN and GFP-FepicQ by MALDI-TOF. **(c)** Binding affinity of the semisynthetic GFPs bearing C-terminal cyclic imides against His-DDB1/CRBN measured by TR-FRET assay.

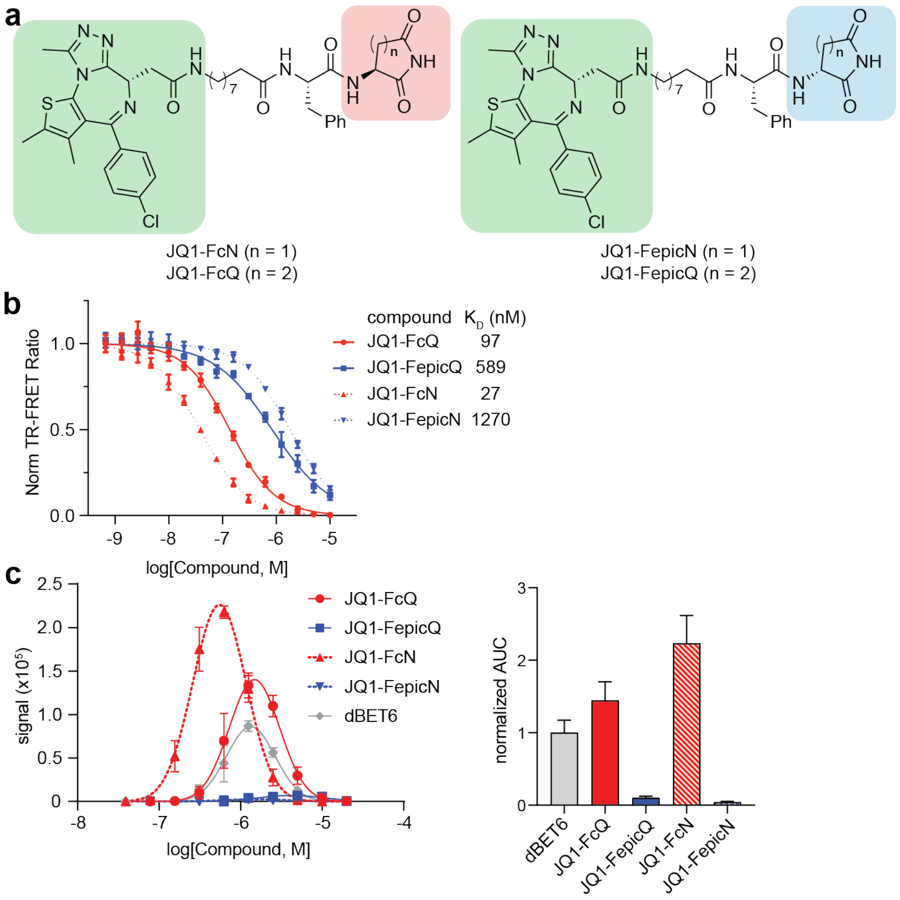

Fig. S13. CRBN engagement and ternary complex formation with JQ1-dipeptide degraders. (a) Structures of JQ1-FcN, JQ1-FcQ, JQ1-FepicN and JQ1-FepicQ. **(b)** K_D values of the indicated compounds against His_6-CRBN/DDB1 complex measured by TR-FRET assay. **(c)** Ternary complex formation with His_6-CRBN/DDB1 complex and GST-BRD4 BD2 domain measured by AlphaScreen.

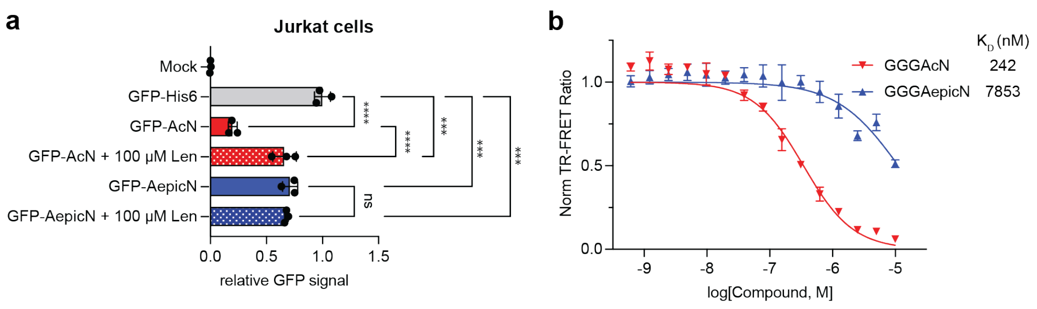

Fig. S14. Recognition of the C-terminal aspartimide degron stereoisomers by CRBN. (a) Levels of engineered GFP with a C-terminal aspartimide degron in Jurkat cells with or without lenalidomide (100 μM) after 6 h. **(b)** K_D values of GGGAcN/AepicN against His_6-CRBN/DDB1 complex, measured by TR-FRET assay.

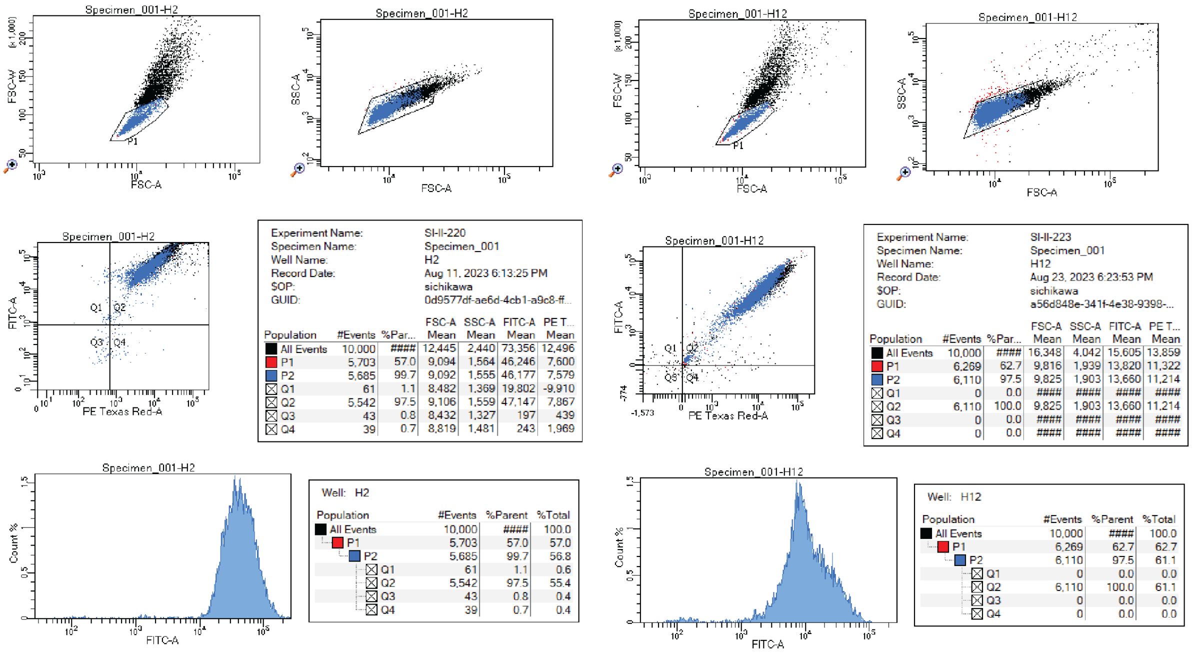

Experiment Name: SI-II-220
Specimen Name: Specimen_001
Well Name: H2
Record Date: Aug 11, 2023 6:13:25 PM
$OP: sichikawa
GUID: 0d9577df-ae6d-4cb1-a9c8-ff...

Population	#Events	%Par...	FSC-A Mean	SSC-A Mean	FITC-A Mean	PE T... Mean
All Events	10,000	####	12,445	2,440	73,356	12,496
P1	5,703	57.0	9,094	1,564	46,246	7,600
P2	5,685	99.7	9,092	1,555	46,177	7,579
Q1	61	1.1	8,482	1,369	19,802	-9,910
Q2	5,542	97.5	9,106	1,559	47,147	7,867
Q3	43	0.8	8,432	1,327	197	439
Q4	39	0.7	8,819	1,481	243	1,969

Experiment Name: SI-II-223
Specimen Name: Specimen_001
Well Name: H12
Record Date: Aug 23, 2023 6:23:53 PM
$OP: sichikawa
GUID: a56d848e-341f-4e38-9398-...

Population	#Events	%Par...	FSC-A Mean	SSC-A Mean	FITC-A Mean	PE T... Mean
All Events	10,000	####	16,348	4,042	15,605	13,859
P1	6,269	62.7	9,816	1,939	13,820	11,322
P2	6,110	97.5	9,825	1,903	13,660	11,214
Q1	0	0.0	####	####	####	####
Q2	6,110	100.0	9,825	1,903	13,660	11,214
Q3	0	0.0	####	####	####	####
Q4	0	0.0	####	####	####	####

Well: H2

Population	#Events	%Parent	%Total
All Events	10,000	####	100.0
P1	5,703	57.0	57.0
P2	5,685	99.7	56.8
Q1	61	1.1	0.6
Q2	5,542	97.5	55.4
Q3	43	0.8	0.4
Q4	39	0.7	0.4

Well: H12

Population	#Events	%Parent	%Total
All Events	10,000	####	100.0
P1	6,269	62.7	62.7
P2	6,110	97.5	61.1
Q1	0	0.0	0.0
Q2	6,110	100.0	61.1
Q3	0	0.0	0.0
Q4	0	0.0	0.0

Fig. S15. Representative FACS gating for Fig. 3d–e, 3i–j, and 4e–f.

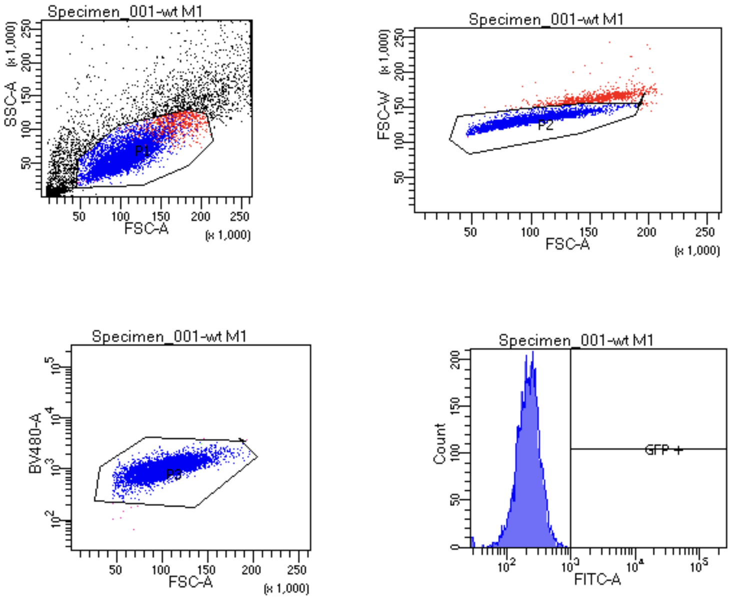

Tube: wt M1

Population	#Events	%Parent	%Total
All Events	10,000	####	100.0
P1	6,234	62.3	62.3
P2	5,301	85.0	53.0
P3	5,290	99.8	52.9
GFP +	1	0.0	0.0

Experiment Name:	HCL 252
Specimen Name:	Specimen_001
Tube Name:	wt M1
Record Date:	Sep 18, 2022 2:36:28 PM
$OP:	hlloyd
GUID:	49bcbb42-c1e7-489b-8aa1-7c0...

Population	#Events	%Parent	FITC-A Mean
P3	5,290	99.8	214
GFP +	1	0.0	1,363

Fig. S16. Representative FACS gating for Fig. 5e–h.

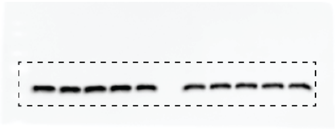

Figure 5c: α-FLAG

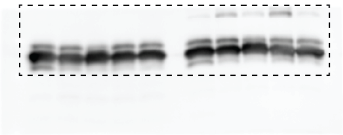

Figure 5c: α-GFP

Fig. S17. Full blot image of Fig. 5c.

Materials and Methods

General Supplies

- (*R*, *S*)-Lenalidomide (BioVision 1862-25)
- CC885 (Axon Medchem 2645)
- MLN4924 (Selleck Chemicals S7109)
- MG132 (Selleck Chemicals S2619)
- 3-(4,5-Dimethylthiazol-2-yl)-2,5-diphenyltetrazolium bromide (MTT) (Sigma-Aldrich 475989)
- Mini bio-spin chromatography columns (Bio-Rad 732-6207)
- Dulbecco's Modified Eagle's Medium (DMEM) (Genesee Scientific 25-500)
- RPMI 1640 (Gibco 10-040-CV)
- Trypsin-EDTA (Fisher Scientific 25200114)
- Fetal bovine serum (FBS) (Peak Serum PS-FB2)
- Penicillin-streptomycin (100×) (Lonza 17-602E)
- NuPAGE 3–8% Tris-Acetate precast gels (ThermoFisher EA0375BOX)
- Criterion XT Tris-Acetate precast gels (Bio-Rad 3450131)
- 4–15% Criterion TGX precast gels (Bio-Rad 5671085)
- iBlot 2 nitrocellulose transfer stack (Invitrogen IB23001; IB23002).
- BCA solution (BCA Reagent A) (VWR 786-847)
- Copper Solution (BCA Reagent B) (VWR 76825-860)
- Tris(3-hydroxypropyltriazolylmethyl)amine (THPTA) (Sigma-Aldrich #762342)
- Azide-Fluor 488 (Sigma-Aldrich #760765).
- Biotin-PEG3-azide (Sigma-Aldrich #762024)
- GelCode Blue Stain Reagent (Thermo Scientific, #24590)
- Protease inhibitor tablets (Roche EDTA-free cOmplete tablets, Sigma-Aldrich, #11836170001)

*All CRBN modulators have the potential for teratogenic properties.

Immunoprecipitation

- Non-denaturing cell lysis buffer (10×) (Cell Signaling Technology 9803S)
- Protein G magnetic beads (Cell Signaling Technology 70024)
- Anti-FLAG M2 beads (Sigma-Aldrich M8823-1ML)
- 2× SDS-PAGE loading buffer (concentration for 1×: 50 mM Tris–HCl, 2% SDS, 10% glycerol, 1% β-mercaptoethanol, 0.02% bromophenol blue)
- 3× FLAG peptide (Sigma-Aldrich F4799-4MG)

Structural/Global Quantitative Proteomics

- S-Trap micro (Protifi)
- Triethylammonium bicarbonate buffer (Sigma-Aldrich T7408-100ML)
- Trypsin/Lys-C mix, mass spectrometry grade (Promega V5073)
- Trypsin, sequencing grade modified (Promega V5111)
- Chymotrypsin, sequencing grade (Promega V1061)
- TMT10plex isobaric label reagent set (ThermoFisher 90406)
- TMTpro 16plex label reagent set (ThermoFisher A44520)
- Pierce high pH reversed-phase peptide fractionation kit (ThermoFisher 84868)

TR-FRET assay

- 384-well plates (Corning 3572 or Greiner 781207)
- Anti-6xHis antibody (Abcam 18184)
- CoraFluor-1-Pfp[2]
- AF488-Tfp (ThermoFisher A37570)
- His_6-CRBN/DDB1 (a generous gift from Bristol Myers Squibb)
- His_6-Thalidomide-binding domain (TBD)

- Thalidomide-FITC

Flow Cytometry Reagents
- DMEM with 4.5 g/L Glucose, without phenol red (Lonza, 12-917F)
- Trypsin-EDTA (0.5%), no phenol red (ThermoFisher, 15400054)
- Propidium iodide (Sigma-Aldrich, Cat# P4170; CAS 25535-16-4)

Alphascreen
- Optiplate 384 (Perkin Elmer 6007290)
- TopSeal-A PLUS (Perkin Elmer 6050185)
- GST-BRD4(BD2) (Epicypher 15-0013)
- GST-IKZF1 (Novus Biologics H00010320-P01-2ug)
- His_6-CRBN/DDB1 (generous gift from Bristol Myers Squibb)
- AlphaScreen Glutathione Donor beads (Perkin Elmer 6765301)
- Nickel chelate AlphaLISA acceptor beads (Perkin Elmer AL108M)
- Alphascreen Omnibeads (Perkin Elmer 6760626D)
- GST-His_6 (EMD Millipore 12-523)

In Vitro Ubiquitination
- Pierce IP lysis buffer (ThermoFisher 87788)
- Protease inhibitor cocktail (Sigma-Aldrich 11873580001, 1 tablet dissolved in 2 mL water for a 25× stock solution)
- Anti-FLAG M2 beads (Sigma-Aldrich M8823-1ML)
- 3× FLAG peptide (Sigma-Aldrich F4799-4MG)
- UBE1 (E1, Boston Biochem E-305-025)
- UbcH5a (E2, aka UBE2D1, Boston Biochem E2-616-100)
- UbcH5c (E2, aka UBE2D3, Boston Biochem E2-627-100)
- K0 ubiquitin (Boston Biochem UM-NOK-01M)
- Ubiquitin aldehyde (Boston Biochem U-201-050)
- Mg-ATP (Boston Biochem B-20)
- E3 ligase buffer (Boston Biochem B-71)
- MG132 (Boston Biochem I13005M)
- MG101 (Tocris 3358)

Electroporation
- Neon Transfection System 100 µL Kit (ThermoFisher MPK10025)
- Neon Transfection System 10 µL Kit (ThermoFisher MPK1025)
- Neon Transfection Tubes (ThermoFisher MPT100)

Mammalian Cell Lines and Tissues
HEK293T, MM.1S and Jurkat cells were obtained from American Type Culture Collection (ATCC). HEK293T cells stably expressing FLAG-CRBN (HEK-CRBN cells) were kindly provided by the Deshaies Lab (California Institute of Technology).[3] HEK293T cells stably expressing IKZF1 ZF-GFP or GSPT1-GFP were generated in our lab.[4,5] HEK293T cells stably expressing BRD4(BD1)-GFP were kindly provided by the Fischer Lab (Dana-Farber Cancer Institute).[6] MOLM13 cells were kindly provided by the Shair lab (Harvard University). BxPC3 cells were kindly provided by the Bertozzi lab (Stanford University).

Antibodies

No.	***Antibody Name***	***Host Species***	***Dilution***	***Supplier***	***Catalog#***
1	BRD4 (E2A7X)	Rabbit mAb	1:1000	Cell Signaling Technology	13440S

2	Vinculin (V284)	Mouse mAb	1:1000	Bio-Rad	MCA465 GA
3	FLAG (M2)	Mouse mAb	1:1000	Sigma-Aldrich	F1804
4	FLAG	Rabbit mAb	1:1000	Cell Signaling Technology	14793S
5	Ikaros/IKZF1 (D6N9Y)	Rabbit mAb	1:1000	Cell Signaling Technology	14859
6	GSPT1	Rabbit pAb	1:1000	Proteintech	10763-1-AP
7	β-actin (C4)	Mouse mAb	1:1000	Santa Cruz Biotechnology	sc-47778
8	GAPDH	Rabbit pAb	1:1000	Cell Signaling Technology	5174S
9	GFP (D5.1)	Rabbit mAb	1:1000	Cell Signaling Technology	2956S
10	Anti-6xHis	Mouse	ref 1	Abcam	Ab18184
11	Anti-mouse-HRP	Goat	1:10,000	Rockland Immunochemicals	610-1302
12	Anti-rabbit-HRP	Goat	1:10,000	Rockland Immunochemicals	611-1302
13	Anti-mouse-IRDye® 800CW	Goat	1:10,000	LI-COR Biosciences	925-32210

Instrumentation
Protein quantification by bicinchoninic acid assay (BCA) was measured on a multi-mode microplate reader FilterMax F3 (Molecular Devices LLC, Sunnyvale, CA, 570 nm filter). AlphaScreen readings were performed on a SpectraMax i3x plate reader equipped with an AlphaScreen Detection Cartridge (384 STD) (Molecular Devices LLC, Sunnyvale, CA). Protein concentration and OD600 measurements were measured by Nanodrop One^{C} Microvolume UV-Vis Spectrophotometer (ThermoFisher). TR-FRET measurements were acquired on a Tecan SPARK plate reader with SPARKCONTROL software version V2.1 (Tecan Group Ltd.). Cell lysis was performed using a Branson Ultrasonic Probe Sonicator (model 250). Fluorescence and chemiluminescence imaging were performed using an Azure Imager c600 or 600 (Azure Biosystems, Inc., Dublin, CA). Protein purification and analytical SEC was performed using an ÄKTA pure 25 equipped with a F9-R fraction collector, a C9n conductivity monitor, and computer running UNICORN v6.3.2.89 (GE Healthcare). All proteomics data were obtained on a Waters ACQUITY UPLC system connected in line to an Orbitrap Fusion Lumos Tribrid Mass Spectrometer or LTQ Orbitrap Velos (both ThermoFisher) within the Mass Spectrometry and Proteomics Resource Laboratory at Harvard University. Intact protein mass spectra were collected using a Bruker Ultraflextreme MALDI-TOF/TOF within the Mass Spectrometry and Proteomics Resource Laboratory at Harvard University. Western blotting transfer was performed using an Invitrogen iBlot 2 dry blotting system. Electroporation was performed using a Neon electroporation system (ThermoFisher). Flow cytometry was conducted using Fortessa and FACSymphony A3 Lite flow cytometers (both BD). Cell sorting was conducted using MoFlo XDP Cell Sorter (Beckman Coulter). Cell numbers and viability were measured using TC20 automated cell counter (Bio-Rad). Samples were dried using a Vacufuge Plus (Eppendorf). Photoirradiation was performed using a Dymax ECE 5000 UV Light-Curing Flood Lamp system (Cat # 41060) in a 4 ºC cold room. The lamp was turned on for at least 15 minutes prior to use.

Software
Data was analyzed and visualized using Microsoft Excel (v16.44), GraphPad Prism (v8.4.3), and Python (v3.8). MOE (Chemical Computing Group, v2020.09) was used to model the protein complexes and the obtained structures were analyzed with PyMOL (v2.3.1). Proteomics data was analyzed using Xcalibur Qual Browser (v3.0.63) and Proteome

Discoverer (v2.4.1.15). Images were made using ImageJ (NIH, v1.52q), Adobe Photoshop (v21.1.1) and Adobe Illustrator (v24.1).

General Procedures

Cell culture

HEK293T cells and HEK-CRBN cells were cultured in DMEM supplemented with penicillin (50 µg/mL), streptomycin (50 µg/mL), and 10% (v/v) FBS. MM.1S, MOLM13, Jurkat, and BxPC3 cells were cultured in RPMI 1640 supplemented with penicillin (50 µg/mL), streptomycin (50 µg/mL), and 10% (v/v) FBS. For collection of the cells, cells were washed twice with PBS and pelleted by centrifugation at 500 × g, 24 °C, 3 min.

Western Blotting Procedures

Unless otherwise noted, cells were lysed by probe sonication (5 sec on, 3 sec off, 15 sec in total, 11% amplitude) in 1% SDS in 1× PBS and cleared by centrifugation at 21,000 × g, 4 °C, 10 min. As needed, a BCA assay was performed to determine the protein concentration of lysates and the concentration was adjusted using lysis buffer. 5× SDS-PAGE loading buffer (5% (v/v) β-mercaptoethanol, 0.02% (w/v) bromophenol blue, 30% (v/v) glycerol, 10% (w/v) SDS/ 250 mM Tris pH 6.8) was added to the protein samples to a final concentration of 1× and the samples were heated at 95 °C for 5 min. Protein samples (8–15 µL per lane) were loaded on NuPAGE 3–8% Tris-Acetate precast gels or Criterion XT Tris-Acetate precast gels for high molecular weight proteins, 6/12% Tris-Glycine gels or 4–15% Criterion™ TGX™ precast gels for medium molecular weight proteins. Gels were transferred to membranes using the Invitrogen iBlot 2 dry blotting system and iBlot 2 nitrocellulose transfer stacks, using program P0 (1 min at 20 V, 4 min at 23 V, 2 min at 25 V) for most proteins and 8 min at 25 V for high molecular weight proteins. Membranes were stained with Ponceau S solution to visualize transfer and protein loading. After being blocked with 5% milk or 5% BSA in TBST at 24 °C for 1 h, the membranes were incubated with primary antibodies at 4 °C for overnight. Membranes were washed (3 × 5 min) with TBST and incubated with secondary antibodies at 24 °C for 1 h. Membranes were washed (3 × 5 min) with TBST and the results were obtained by chemiluminescence and/or IR imaging using Azure 600 or c600.

Degradation assay by flow cytometry

Cells were seeded in 96-well plates ($4×10^4$ cells per well) 1 h before compound treatment. DMSO or each compound at each concentration was added into three separate wells (final DMSO concentration: 0.2%) and incubated at 37 °C, 5% CO_2 for 24 h. The cells were detached by trypsinization in 0.25% trypsin without phenol red (diluted 2× by PBS), followed by resuspension in DMEM +/– without phenol red. For IKZF1 degradation assay, propidium iodide/PBS (0.5 mg/mL, 4 µL) was added to each well at this point.

All plates were subsequently analyzed using the FACSymphony system (BD Biosciences) equipped with a high throughput sampler. A minimum of 10,000 events per well were recorded, and the fluorescence signals from FITC and PE Texas Red were measured. The abundance of specific target proteins (namely IKZF1 zinc-finger, GSPT1, or BRD4(BD1)) was determined by calculating the ratio of the geometric mean of the FITC signal to the PE Texas Red signal using the BD FACSDiva software v9.1. For each experimental condition, the arithmetic mean of the ratios from the triplicate wells was determined and then normalized to the corresponding DMSO ratios. This normalization provided the relative abundance of the target protein when comparing compound treatment to DMSO control.

Structural proteomics mass spectrometry sample preparation

Samples were prepared according to the previous report.[7] Briefly, 20 µM thalidomide-binding domain and 22 µM of the indicated compound in 0.1% Triton-X 100 in PBS were incubated for 30 min at 25 °C, followed by photoirradiation for 30 sec at 4 °C. A stock solution of 10% RapiGest/PBS was added to the photo-irradiated sample to a final concentration of 1 %. The denatured proteins were tagged with a cleavable biotin picolyl-azide probe[8] via CuAAC by

addition of a pre-mixed cocktail (final concentrations: 100 µM cleavable biotin picolyl-azide probe, 250 µM THPTA, 250 µM $CuSO_4$, 2.5 mM freshly dissolved sodium ascorbate) and incubated for 1.5 h at 25 °C with inversion. After excess reagent was removed by acetone precipitation, the protein pellet was resuspended in 100 mM TEAB (50 µL, pH=8.5) and 100 mM DTT/PBS (1.5 µL, final concentration 3 mM) was added before incubating for 30 min at 25 °C with inversion. 500 mM iodoacetamide/PBS (1.0 µL, final concentration 10 mM) was then added to the sample and incubated for 30 min at 25 °C in the dark with inversion. The protein was then digested by adding sequence-grade chymotrypsin (0.5 µg) in 1 mM HCl and incubating for 15–18 h at 25 °C with inversion. The digested protein was acidified by formic acid (1.0 µL) and incubated for 30 min at 25 °C with inversion to cleave the cleavable biotin picolyl-azide probe. The sample was desalted using a C18 Ziptip according to the manufacture's protocol with elution in 0.1% formic acid/70% acetonitrile/water and the desalted peptides were dried on a Speed-Vac. The dried sample was re-suspended in 20 µL 0.1% formic acid/water for analysis.

<u>Structural proteomics mass spectrometry procedures.</u>
The sample was loaded onto a microcapillary trapping column (C18 Reprosil resin, Dr. Maisch, 5 µm particle size, 100 Å pore size, 30 mm length, 100 µm internal diameter) and then separated on an analytical column (50 cm µPAC™ column, PharmaFluidics) at 200 nL/min. The column temperature was maintained at 30℃. Peptides were eluted with a water/acetonitrile gradient (buffer A = 0.1% formic acid/water, buffer B = 0.1% formic acid/acetonitrile; flow rate 200 nL/min; gradient: hold at 2% B for 5 min, increase to 6% B over 5 min, increase to 35% B over 90 min, increase to 95% B over 10 min, hold at 95% B for 10 min). Electrospray ionization was enabled through applying a voltage of 2.2 kV. The Lumos Orbitrap was operated in data-dependent mode and survey scans of peptide precursors were performed at 120K FWHM resolution over a *m/z* range of 410-1800. Tandem MS was performed on the 10 most abundant precursors exhibiting a charge state from 2 to 6 with collision energy of 35% and 35 ms maximum injection time for CID, collision energy of 37% and 200 ms maximum injection time for HCD, and supplemental activation collision energy of 37% and 250 ms maximum injection time for EThcD, all at 15K FWHM resolution and 3 m/z isolation window. With a mass tolerance of 10 ppm, selected precursors were excluded from further fragmentation for 30 s after 2 occurrences within 30 s.

<u>Structural proteomics data analysis procedures</u>
The raw data were analyzed using Proteome Discoverer 2.4.1.15. Assignment of MS/MS spectra was performed using the Sequest HT algorithm by searching the data against a protein sequence database of thalidomide-binding domain and common contaminant proteins. Search parameters included: mass tolerance of 10 ppm for the precursor, 0.02 Da for HCD and EThcD fragment ions, 0.6 Da for CID fragment ions, semi-specific digestion, 2 missed cleavages, static modification of carbamidomethylation on cysteine residues (+57.0214 Da), a dynamic oxidation on methionine residues (+15.9949 Da), a dynamic deamidation on asparagine and glutamine residues (+0.9847 Da) and a dynamic modification of the compounds on any amino acid residues (isobaric photo-lenalidomide: +510.1993 Da). Peptide spectral matches (PSMs) were validated with the Target Decoy PSM Validator. For spectra assigned as isobaric photo-lenalidomide-conjugated peptides, complementary ions derived from neutral loss of glutarimide were deconvoluted and quantified by in-house algorithm (https://github.com/christinawoo/i-pLen_TMTc) adapted from TMTc+ deconvolution method[9] and further were manually validated.

<u>Structural modeling details</u>
Docking study was performed with MOE version 2020.09 as previously described.[10] Briefly, A ligand molecule in the indicated crystal structure was adapted to the indicated compounds and the adapted complex was subjected to energy minimization with Amber10:EHT force field followed by preparation with Protonate 3D. Ligand conformations were generated with the bond rotation method and the docking was performed with the Triangle Matcher placement

method and scored with the London dG scoring function. The lowest energy conformations were rendered and 30 of them were submitted to a refinement step with the Induced Fit method and rescored with the GBVI/WSA dG scoring function. A conformation of each complex with the lowest S score given by the GBVI/WSA dG scoring function was analyzed for the ligand interactions.

In-gel fluorescence imaging of thalidomide-binding domain–photo-lenalidomide conjugates

10 µM Thalidomide-binding domain and (*S*)-, (*R*)-, rac-photo-lenalidomide or DMSO at the indicated concentration in 20 µL 0.1% Triton-X 100 in PBS were incubated for 30 min at 25 °C in the dark, followed by photoirradiation for 30 sec at 4 °C. Protein was precipitated to remove unreacted reagents by adding 80 µL cold acetone and incubating at -80 °C for 30 min, followed by centrifugation at 21,000 × g, 4 °C, 10 min. The air-dried protein pellet was resuspended in 20 µL 1% SDS/PBS and reacted with a mixture of Azide-Fluor 488 (AF488, 25 µM), THPTA (100 µM), $CuSO_4$ (1 mM) and sodium ascorbate (2 mM) for 1 h at 25 °C with inversion in the dark. The reaction was quenched by addition of 5 µL 5x SDS-PAGE loading buffer (5% (v/v) β-mercaptoethanol, 0.02% (w/v) bromophenol blue, 30% (v/v) glycerol, 10% (w/v) SDS/ 250 mM Tris pH 6.8) and heated at 95 °C for 5 min. 10 µL of each sample was loaded on 15% Tris-glycine gel and probe labeling was visualized by analyzing fluorescence signal and protein loading visualized with gel-code blue staining.

Time-course Study of Racemization of Lenalidomide and Photo-lenalidomide in RPMI Medium

To a 12-well plate, 25 µL of the indicated compound (4 mM stock solution in methanol) was added and methanol was air-dried. 1 mL of RPMI medium was added to each well containing the compound and the plate was incubated at 37 °C, 5% CO_2. At the indicated time point, RPMI medium containing the compound was collected in a 5-mL Eppendorf tube, and 4 mL of HPLC-grade ethyl acetate was added to each tube. The tube was shaken for 2 min to extract the compounds into ethyl acetate. After two layers were separated, 3 mL was taken from the organic layer and filtered through sodium sulfate to remove residual water. To the same 5-mL Eppendorf tube, 3 mL of HPLC-grade ethyl acetate was added to repeat the extraction step. The taken organic layers were combined and concentrated in vacuo to dryness. The sample was resuspended in HPLC grade isopropanol and analyzed with Waters Prep 150 LC System equipped with a chiral column with conditions indicated in the synthetic procedure.
The experimental data obtained were fitted to the following rate equation for racemization:

$$Y = \frac{1}{2}(1 - e^{-2K_{rac}t})$$

Where Y represents the ratio of the other enantiomer formed via racemization with constraints of Top = 1 (100% conversion) and Bottom = 0 (0% conversion), K_{rac} is the rate constant for the racemization, and t is incubation time. For the sake of symmetry, rate constants are the same in both directions.
In the analysis of the above curve fitting model, parameter estimation for K_{rac} was carried out using nonlinear least squares regression, employing the “curve_fit” function from the SciPy library in Python. This approach yielded an estimated value for K_{rac}. To ascertain the precision of this estimation and to quantify the associated uncertainty, the standard error of K_{rac} was calculated based on the diagonal elements of the covariance matrix returned by the curve fitting procedure. Subsequently, to construct a 95% confidence interval for the extrapolated racemization curves, the t-distribution was employed, considering the degrees of freedom (the number of data points minus the number of fitted parameters). The critical t-value for a 95% confidence level was determined by using the “t.ppf” function from the SciPy library in Python, and the confidence interval for K_{rac} was then derived as the following equation:

$$K_{rac} \pm t_{critical} \times SE(K_{rac})$$

Where $t_{critical}$ is the critical t-value for a 95% confidence level and $SE(K_{rac})$ is the standard error of K_{rac}.
The upper and lower bounds of this interval were further utilized to generate the corresponding confidence bounds for the curve, offering a visual representation of the uncertainty inherent in the model predictions.

Correction Method to Account for Racemization During the Assay Time Window

The primary motivation for this correction was to account for the contribution of (*S*)-enantiomer generated via racemization in each sample, enabling an accurate assessment of whether the (*R*)-enantiomer independently contributes to CRBN binding and CRBN-dependent activities. Without this correction, the apparent binding affinity and degradation potency of the (*R*)-enantiomer or racemate samples could be confounded by the accumulation of (*S*)-enantiomer, as even a minor fraction of the (*S*)-enantiomer—given its tight binding and potent activity—can significantly influence the observed readouts.

To achieve the correction described above, we implemented the following steps:

1. Estimating the average (*S*)-enantiomer fraction over time: The area under the racemization curve was integrated and divided by the assay duration, yielding the average proportion of (*S*)-enantiomer present in the (*R*)-enantiomer or racemate sample throughout the assay duration. The same analysis was also applied to the (*S*)-enantiomer sample to estimate the average proportion of (*R*)-enantiomer present within it.
2. Adjusting the dose-response curve for the (*S*)-enantiomer contribution: Under the null hypothesis that only the (*S*)-enantiomer contributes to CRBN binding and CRBN-dependent activity, we adjusted the dose-response curves accordingly. Because dose-response data are plotted on a logarithmic scale, a log transformation was applied: each log(concentration) value on the (*R*)-enantiomer dose-response curve was shifted by log(X), where X represents the average ratio of (*S*)-enantiomer present in the (*R*)-enantiomer or racemate sample (e.g., 10% corresponds to ratio X = 0.1). This transformation predicts the expected dose-response curve for the (*R*)-enantiomer or racemate sample if there is no independent contribution from the (*R*)-enantiomer. For completeness, the same correction was also applied to the (*S*)-enantiomer curve to account for the in situ formation of the (*R*)-enantiomer, although this adjustment fell within the error margins of the original curve.
3. Statistical comparison of the corrected binding curves: Under the null hypothesis—assuming it holds true—the dose-response curve for the (*R*)-enantiomer or racemate sample (corrected to account solely for the contribution of the (*S*)-enantiomer) and the curve for the (*S*)-enantiomer sample (adjusted to account for the small proportion of (*R*)-enantiomer formed via racemization) should not display any statistically significant differences. To test this, the corrected two curves mentioned above were compared for each compound (e.g., lenalidomide, CC885, and JQ1-Len) using an extra sum-of-squares F-test. This analysis determined whether there was a statistically significant difference between the two dose-response curves, thereby assessing whether the (*R*)-enantiomer exhibits independent activity beyond the contribution from racemization-derived (*S*)-enantiomer. If there is a statistically significant difference, the null hypothesis is rejected, supporting independent CRBN engagement by the (*R*)-enantiomer.

Immunoprecipitation

For immunoprecipitation of proteins with the FLAG tag, 2.5–5.0x10^6 HEK-CRBN cells were seeded and incubated at 37 °C, 5% CO_2 for 18–24 h. Cells were treated with 1 μM MLN4924 for 1 h and followed by the indicated compound for 2 h. The cells were collected and lysed in 1× protease/phosphatase inhibitor/1× non-denaturing lysis buffer (150 μL) and clarified by centrifugation (21,000 × g, 4 °C, 10 min). Protein concentration of the soluble portion of the lysate was measured by BCA assay and diluted to 2.5 mg/mL. 200 μL of lysate was incubated with of protein G magnetic beads for 20 min (20 μL) to minimize the non-specific binding. The solution was collected and was then incubated with anti-FLAG M2 magnetic beads (30 μL) on a tube rotator at 4 °C for 1 h. The magnetic beads were washed with 1× non-denaturing lysis buffer (5× 500 μL). Then, the enriched proteins were eluted by addition of 30 μL of 2× SDS-PAGE loading buffer and heated at 95 °C for 5 min prior to Western blot analysis.

TR-FRET

TR-FRET measurements

Unless otherwise noted, experiments were performed in white, 384-well microtiter plates (Corning 3572 or Greiner 781207) in 30 µL assay volume. TR-FRET measurements were acquired on a Tecan SPARK plate reader with SPARKCONTROL software version V2.1 (Tecan Group Ltd.), with the following settings: 340/50 nm excitation, 490/10 nm (Tb), and 520/10 nm (FITC) emission, 100 µs delay, 400 µs integration. The 490/10 nm and 520/10 nm emission channels were acquired with a 50% mirror and a dichroic 510 mirror, respectively, using independently optimized detector gain settings unless specified otherwise. The TR-FRET ratio was taken as the 520/490 nm intensity ratio on a per-well basis.

Protein labeling

Anti-6xHis antibody (Abcam 18184) and His_6-thalidomide-binding domain were labeled with CoraFluor-1-Pfp ester as previously described [34675420]. The following extinction coefficients were used to calculate protein concentration and degree-of-labeling (DOL): IgG E_{280} = 210,000 $M^{-1}cm^{-1}$, His-thalidomide-binding domain E_{280} = 28,460 $M^{-1}cm^{-1}$, CoraFluor-1-Pfp E_{340} = 22,000 $M^{-1}cm^{-1}$. Protein conjugates were snap-frozen in liquid nitrogen, and stored at -80°C.

Determination of equilibrium dissociation constant (K_D) for Thal-FITC by TR-FRET

The following conditions have been employed: (i) 1 nM CoraFluor-1-labeled anti-6xHis antibody, 2.5 nM 6xHis-CRBN-DDB1 complex in assay buffer (25 mM HEPES, 150 mM NaCl, 0.5 mg/mL BSA, 0.005% TWEEN-20, pH 7.5), or (ii) 50 nM CoraFluor-1-labeled His-thalidomide-binding domain in assay buffer. Thal-FITC was added in serial dilution using a HP D300 digital dispenser (Hewlett-Packard) and allowed to equilibrate for 1 h at room temperature before TR-FRET measurements were taken. Nonspecific signal was determined from (i) wells containing the CoraFluor-1-labeled anti-6xHis antibody conjugate alone in the absence of the target protein, or (ii) wells containing 100 µM 5-NH_2-EM12.[11] Data were fitted to a One Site – Specific Binding model in Prism 9.

For system (i), employing anti-6xHis antibody and His_6-CRBN/DDB1 complex, The K_D determined from the one-site model was then used in Equation 1 to adjust for a two-site model due to the presence of bivalent anti-6xHis antibody:

$$K_{D,2-site} = K_{D,1-site} \times \left(1+\sqrt{2}\right) \qquad (1)$$

In subsequent TR-FRET ligand displacement assays, inhibitor K_D values have been calculated using Cheng-Prusoff principles, outlined in Equation 2 below:

$$K_D = \frac{IC_{50}}{1+\frac{[S]}{K_X}} \qquad (2)$$

Where IC_{50} is the measured IC_{50} value, [S] is the concentration of Thal-FITC, and K_X is the K_D of the fluorescent tracer for a given condition [4202581].

TR-FRET ligand displacement assays

The following conditions have been employed: (i) 1 nM CoraFluor-1-labeled anti-6xHis antibody, 2.5 nM His_6-CRBN-DDB1 complex, and 50 nM Thal-FITC in assay buffer, (ii) 50 nM CoraFluor-1-labeled His-thalidomide-binding domain, 775 nM Thal-FITC in assay buffer, or (iii) 4 nM GST-BRD4(BD2), 4 nM CoraFluor-1-labeled anti-GST VHH, and 20 nM JQ1-FITC in assay buffer. Test compounds were added in serial dilution (1:2 titration, 15-point, c_{max} = 10 or 100 µM) using a HP D300 digital dispenser and allowed to equilibrate for 1 h at room temperature before TR-FRET measurements were taken. The assay floor (bottom) was determined from wells treated with 10 or 100 µM dBET6, and the assay ceiling (top) was defined via a no-inhibitor control. Data were background-corrected, normalized and fitted to a four-parameter dose-response model [log(inhibitor vs. response – Variable slope (four parameters)] in Prism 9, with constraints of Top = 1 and Bottom = 0.

AlphaScreen

AlphaScreen was performed according to the previously reported method.[7] Briefly, for AlphaScreen of His_6-CRBN/DDB1, GST-IKZF1 and (*S*)-, (*R*)- or *rac*-lenalidomide-d_3, a solution of 100 nM His_6-CRBN/DDB1 and 200 nM GST-IKZF1 in 1x AlphaScreen buffer (5 µL) and a solution of the indicated compound in 1x AlphaScreen buffer (5 µL) were added to a each well of a 384-well Optiplate and incubated at 24 °C for 1 h. To each well, a solution of 5 µL of 60 µg/mL AlphaScreen Glutathione Donor beads, 60 µg/mL AlphaLISA Nickel Chelate Donor beads in 1× AlphaScreen buffer was added to each well and incubated at 24 °C for 1 h in the dark and the plate was analyzed. For AlphaScreen of His_6-CRBN/DDB1, GST-BRD4(BD2) and bivalent degraders, a solution of 750 nM His-CRBN/DDB1 and 375 nM GST-BRD4(BD2) in 1x AlphaScreen buffer (5 µL) and a solution of the indicated compound in 1x AlphaScreen buffer (5 µL) were added to a each well of a 384-well Optiplate and incubated at 24 °C for 1 h. To each well, a solution of 5 µL of 60 µg/mL AlphaScreen Glutathione Donor beads, 60 µg/mL AlphaLISA Nickel Chelate Donor beads in 1× AlphaScreen buffer was added to each well and incubated at 24 °C for 1 h in the dark and the plate was analyzed.

Photoaffinity labeling experiment in cells and in-gel fluorescent imaging

$5.0x10^6$ HEK-CRBN cells were seeded in basal DMEM and indicated at 37 °C, 5% CO_2 for 3 h. The cells were treated with 10 µM MG132 for 30 min and followed by the indicated compound for 1 h. The cells were photo-irradiated at 4 °C (60 sec on, 30 sec off, 120 sec in total). The cells were collected and washed with PBS twice and lysed in 1× protease/phosphatase inhibitor/1× non-denaturing lysis buffer (200 µL) and clarified by centrifugation (21,000 × g, 4 °C, 10 min). Protein concentration of the soluble portion of the lysate was measured by BCA assay and diluted to 1.5 mg/mL. 500 µL of lysate was incubated with anti-FLAG M2 magnetic beads (30 µL) on a tube rotator at 4 °C for 1.0 h. Supernatant was saved as the "input" sample for the following Western blotting analysis. The magnetic beads were washed with TBST (3× 500 µL) and the washed beads were incubated with 20 µL of 3x FLAG peptide (100 ng/µL) at 4 °C for 30 min to elute the bound proteins. The solution was collected in a tube and the elution was repeated again. The combined elution (40 µL) was reacted with a mixture of Azide-Fluor 488 (AF488, 25 µM), THPTA (250 µM), $CuSO_4$ (250 µM) and sodium ascorbate (2.5 mM) for 1 h at 25 °C with inversion in the dark. The reaction was quenched by adding 10 µL of 5x SDS-PAGE loading buffer (5% (v/v) β-mercaptoethanol, 0.02% (w/v) bromophenol blue, 30% (v/v) glycerol, 10% (w/v) SDS/ 250 mM Tris pH 6.8) and heated at 95 °C for 5 min. 10 µL of the immunoprecipitated sample was load on a 12% Tris-glycine gel and probe-labeling was visualized by fluorescence signal. Separately, 10 µL of the immunoprecipitated sample and the "input" sample were load on a 12% Tris-glycine gel and analyzed by Western blotting.

PRISM Screen

Cell Lines

The current PRISM cell set consists of 931 cell lines representing more than 45 lineages including both adherent and suspension/hematopoietic cell lines. These cell lines largely overlap with and reflect the diversity of the Cancer Cell Line Encyclopedia (CCLE) cell lines (see https://portals.broadinstitute.org/ccle). Cell lines were grown in RPMI 10% FBS without phenol red for adherent lines and RPMI 20% FBS without phenol red for suspension lines. Parental cell lines were stably infected with a unique 24-nucleotide DNA barcode via lentiviral transduction and blasticidin selection. After selection, barcoded cell lines were expanded and QCed (mycoplasma contamination test, a SNP test for confirming cell line identity, and barcode ID confirmation). Passing barcoded lines were then pooled (20-25 cell lines per pool) based on doubling time and frozen in assay-ready vials.

PRISM Screening

Test compounds were added to 384-well plates and run at 8 pt. dose with 3-fold dilutions, the highest concentration of 25 µM in triplicate. These assay ready plates were then seeded with

the thawed cell line pools. Adherent cell pools were plated at 1250 cells per well, while suspension and mixed adherent/suspension pools were plated at 2000 cells per well. Treated cells were incubated for 24 hours then lysed. Lysate plates were collapsed together prior to barcode amplification and detection.

Barcode Amplification and Detection
Each cell line's unique barcode is located at the end of the blasticidin resistance gene and gets expressed as mRNA. These mRNAs were then captured by using magnetic particles that recognize polyA sequences. mRNA was then reverse-transcribed into cDNA and then the sequence containing the unique PRISM barcode was amplified using PCR. Finally, Luminex beads that recognize the specific barcode sequences in the cell set were hybridized to the PCR products and then detected using a Luminex scanner which reports signal as a median fluorescent intensity (MFI).

Data Processing

I. For each plate, we first normalize the logMFI (log2 mean fluorescence intensity) of the DMSO wells to their median logMFI.
II. Each detection well contains 10 control barcodes in increasing abundances as spike-in controls. A monotonic smooth p-spline is fit for each control barcode detection well to normalize the abundance of each barcode to the corresponding value in the plate-wise median DMSO profiles. Next, all the logMFI values in the well are transformed through the inferred spline function to correct for amplification and detection artifacts.
III. Next, the separability between negative and positive control treatments is assessed. In particular, we use the error rate of the optimum simple threshold classifier between the control samples for each cell line and plate combination. Error rate is a measure of overlap of the two control sets and is defined as

$$\text{Error}=\frac{FP+FN}{n}$$

where FP is false positives, FN is false negatives, and n is the total number of controls. A threshold is set between the distributions of positive and negative control logMFI values (with everything below the threshold said to be positive and above said to be negative) such that this value is minimized. Additionally, we also compute the strictly standardized mean difference (SSMD)[12], for each cell line. SSMD is defined as

$$\text{SSMD}=\frac{\mu_- - \mu_+}{\sqrt{\sigma_-^2 + \sigma_+^2}}$$

where μ+/− and σ+/− stand for the median and standard deviation of the normalized logMFI values in positive/negative control samples.

IV. We filter out cell lines with error rate above 0.05 from the downstream analysis. Additionally, any cell line that has less than 2 passing replicates is also omitted for the sake of reproducibility. Finally, we compute viability by normalizing with respect to the median negative control for each plate. Log-fold-change viabilities are computed as

$$\text{log-viability} = \log_2(x) - \log_2(\mu_-)$$

where log2(x) is the corrected logMFI value in the treatment and log2(μ−) is the median corrected logMFI in the negative control wells in the same plate.

V. Log-viability scores are corrected for batch effects coming from pools and culture conditions using the ComBat algorithm.[13]

VI. We fit a robust four-parameter logistic curve to the response of each cell line to the compound:

$$f(x) = b + \frac{a-b}{1+e^{s \log \frac{x}{EC50}}}$$

and compute AUC and IC50 values for each dose-response curve.

VII. Finally, the replicates are collapsed to a treatment level profile by computing the median score for each cell line.

Biomarker Identification
After data processing, we explored the univariate associations between the PRISM sensitivity profiles and the genomic features or genetic dependencies. In particular, we computed the Pearson correlations and associated p-values.
Correlations and p-values for log-viability values at each dose, AUC scores and logIC50 values were tabulated. For each dataset, the q-values were computed from p-values using the Benjamini-Hochberg algorithm. Associations with q-values above 0.1 were filtered out and q-values below 1e-20 plotted at 1e-20 for plot readability.

Univariate models were run on available feature sets including CCLE genomic characterization data such as gene expression, cell lineage, mutation, copy number, metabolomics, and proteomics, as well as loss-of-function genetic perturbation (both RNAi and CRISPR) data from the Dependency Map. In addition to these datasets, viability data from the PRISM drug repurposing project was used as a feature set for univariate analysis. For discrete data, such as mutation and lineage, a t-test was done to determine differential sensitivities. For continuous data, such as gene expression, correlations between sensitivity and the characteristic of interest were calculated to determine any association.

Cell viability assay
50,000 cells (MOLM13)/20,000 cells (BxPC3) were seeded in a 96-well plate containing 100 µL RPMI supplemented with 10% FBS and 1× penicillin-streptomycin and incubated at 37 °C, 5% CO_2, 24 h. The compound of interest was added to each well to the indicated concentration of from 200× stock solutions in DMSO (0.5 µL) and incubated at 37 °C, 5% CO_2, 24 h. To each well, 10 µL of 3-(4,5-Dimethylthiazol-2-yl)-2,5-diphenyltetrazolium bromide (MTT) in PBS (4 mg/mL) was added and the cells were incubated for 4 h at 37 °C. The formazan crystals were solubilized by the addition of 100 µL of 10% SDS/0.01 M HCl per well, and the plate was allowed to incubate at 37 °C overnight. The absorbance at 570 nm was measured to quantify the formazan generated in each well. The blank was defined by wells containing media and MTT reagent without any cells. For each treatment well, the cell viability was calculated by subtracting the blank value and normalizing to the average absorbance of the vehicle control wells (cells treated with DMSO).

Global Quantitative Proteomics Sample Preparation and Mass Spectrometry Acquisition
Global Quantitative Proteomics was performed according to the previously reported method.[7] Briefly, 1.0×10^6 MOLM13 cells were seeded in 12-well plates and small molecules of interest were added, and cells were incubated at 37 °C, 5% CO_2 for 4 hours. Cells were collected according to the described general procedure, lysed by probe sonication (5 sec on, 3 sec off, 15 sec in total, 11% amplitude) in lysis buffer (5% SDS in 50 mM triethylammonium bicarbonate (TEAB), pH 7.55), and cleared by centrifugation (21,000 × g, 4 °C, 10 min). After protein quantification by BCA protein assay, the lysates were diluted to 1.5 mg/mL with the lysis buffer. The diluted lysates (60 µL) were reduced by addition of dithiothreitol (20 mM) at 24 °C for 30 min then alkylated by addition of iodoacetamide (40 mM) and incubation in the dark at 24 °C for 30 min.

The samples were desalted and digested with trypsin/Lys-C mix with using a S-Trap micro according to the manufacture's protocol. The digested samples were concentrated to dryness in a vacufuge and resuspended in 25 µL ddH_2O. For each resuspended sample, 12.5 µL was taken for labeling with TMT reagent (5 µL) at 24 °C for 1 h. Hydroxylamine (50%, 3 µL) was added to each sample to quench the TMT reagent, and the samples were incubated at 24 °C for 15 min. The TMT-labeled samples were combined and dried in a vacufuge. The dried sample was resuspended in 720 µL 0.1% trifluoroacetic acid (TFA) and 100 µL was taken and diluted with 200 µL 0.1% TFA to a final volume of 300 µL. The sample was fractionated to 20 fractions using a Pierce high pH reversed-phase peptide fractionation kit. The peptides were eluted sequentially by 4% acetonitrile/0.1% triethylamine (TEA) through 20% acetonitrile/0.1% TEA in 1% acetonitrile increments (17 fractions), followed by 25%, 30% and 50% acetonitrile/0.1% TEA. The first fraction (4% acetonitrile/0.1% TEA) was excluded from LC-MS/MS analysis. The other fractions were concentrated to dryness and each sample was resuspended in 20 µL of 0.1% formic acid prior to LC-MS/MS analysis. Mass spectrometry acquisition was performed according to the reported procedure.[7]

<u>Global Quantitative Proteomics Data Analysis</u>

Data analysis was performed in Thermo Scientific Proteome Discoverer version 2.4.1.15. according to the previously reported method.[7] Briefly, the raw data were searched against SwissProt human (*Homo sapiens*) protein database (19 August 2016; 20,156 total entries) and contaminant proteins using the Sequest HT algorithm. Searches were performed with the following guidelines: spectra with a signal-to-noise ratio greater than 1.5; mass tolerance of 10−20 ppm for the precursor ions and 0.02 Da (HCD) and 0.6 Da (CID) for fragment ions; full trypsin digestion; 2 missed cleavages; variable oxidation on methionine residues (+15.995 Da); static carboxyamidomethylation of cysteine residues (+57.021 Da); static TMT labeling (+304.207 Da for TMTpro-16plex) at lysine residues and N-termini. The TMT reporter ions were quantified using the Reporter Ions Quantifier node and normalized such that the summed peptide intensity per channel was equal. Peptide spectral matches (PSMs) were filtered using a 1% false discovery rate (FDR) using Percolator. PSMs were filtered to PSMs in only one protein group with an isolation interference under 70%. For the obtained proteome, the data were further filtered to include only master proteins with high protein FDR confidence and exclude all contaminant proteins. The data were additionally filtered to proteins with greater than or equal to 3 unique peptides and greater or equal to 4 PSMs. For the p-value and fold change calculations, the data were further processed according to the methods of Huber and coworkers.[14] The model incorporates dependence of the variance on the mean intensity and a variance-stabilizing data transformation. In brief, missing abundances were filled in with minimum noise level computed by taking the minimum for each channel in Control and minimum for each channel in Treatment. A set of 2000 centroids were generated at random from the absolute maximum in the Control and Treatment and the absolute minimum in Control and Treatment, and a minimum noise level was generated using a K-means clustering method. If one abundance was missing, then the instance was filled with the geometric mean of the PSM for Control or Treatment. If all abundances were missing for Control and Treatment or the variance between existing abundances was above 30%, the PSM was removed. P-values for the abundance ratios were calculated using the t-test (background) method. The code used for data analysis is available on GitHub:
https://github.com/harvardinformatics/quantproteomics/tree/master/PEA

<u>Time-Course Study of Racemization of C-Terminal Cyclic Imides</u>

To a 1.7 mL microcentrifuge tube with 500 µL of 0.1 M phosphate buffer pH 7.4, 0.5 µL of 50 mM Fmoc-GGG-FcQ, Fmoc-GGG-FepicQ, Fmoc-GGG-FcN or Fmoc-GGG-FepicN in DMSO was added to a final concentration of 50 µM. Each sample was prepared in triplicate and the resulting solution were placed in a sand bath pre-heated at 37 °C. From each tube, 40 µL was taken at the indicated time to monitor reaction progress and the taken samples were frozen with dry ice and stored at –20 °C until analysis. For Fmoc-GGG-FcQ and Fmoc-GGG-FepicQ samples, 3 µL of each sample was injected to a Waters ACQUITY UPLC system with SQ

Detector 2 mass spectrometer and the epimers and the hydrolyzed products were separated on a ACQUITY UPLC BEF C18, 1.7 µm, 1.2 x 50 mm column with following conditions; buffer A = 0.1% formic acid/water, buffer B = 0.01% formic acid/acetonitrile; flow rate 0.3 mL/min; gradient: increase from 5 to 30% B over 1 min, increase to 40% B over 10 min, increase to 90% B over 1 min, hold at 90% B for 0.9 min, decrease to 5% B over 0.1 min, hold at 5% B for 0.9 min, retention time of Fmoc-GGG-FcQ; 5.6 min, Fmoc-GGG-FepicQ; 5.8 min). For Fmoc-GGG-FcN and Fmoc-GGG-FepicN samples, 25 µL of each sample was injected to Agilent 1260 Infinity II Preparative HPLC System and the epimers and the hydrolyzed products were separated on DAICEL CHIRALPAK ID, 5.0 µm, 4.6 x 150 mm with following conditions; buffer A = water, buffer B = acetonitrile, flow rate 1.5 mL/min, increase from 10 to 90% B over 10 min, hold at 90% B for 2 min, decrease to 10% B over 0.1 min, hold at 10% B for 1.9 min, retention time of Fmoc-GGG-FcN; 7.4 min, Fmoc-GGG-FepicQ; 8.0 min). The conversion of each synthetic peptides to its cyclic imide epimer and hydrolyzed form was calculated based on the integration of the corresponding peaks relative to the integration of the corresponding starting material.

In Vitro Ubiquitylation of C-Terminally-Tagged GFPs

C-terminally-tagged GFPs were synthesized and in vitro ubiquitylation was performed according to the reported procedure.[7] Briefly, HEK-CRBN cells were grown to confluency in a 150-mm plate and the cells were collected and flash frozen with liquid nitrogen, and stored at –80 °C until use. The pellet from a confluent 150-mm plate was thawed on ice, and was resuspended in 1x protease inhibitor cocktail/Pierce IP lysis buffer (1 mL). Lysates were incubated on ice for 10 min, then were clarified by centrifugation (21,000 × g, 4 °C, 10 min). The soluble portions of the lysates were collected, and 150 µL of TBS-washed anti-FLAG M2 beads were added. Samples were incubated at 4 °C on a roller for 1 h. Using a magnetic tube rack, the beads were collected and washed 3× with 1 mL TBS. Each sample was then eluted with by adding 100 µL of 100 ng/µL 3× FLAG peptide/TBS and incubating at 4 °C on a roller for 1 h. The eluent was collected. Ubiquitylation master mixes were prepared at 2× with and without E1 and E2 enzymes. Final concentrations (1×) of the master mix components were 0.2 µM UBE1, 2 µM UbcH5a, 1 mM UbcH5c, 400 µg/mL K0 ubiquitin, 1 µM ubiquitin aldehyde, 1× Mg-ATP, 1× E3 ligase buffer, 10 µM MG132, and 100 nM MG101. Reactions were prepared by combining 6.25 µL FLAG eluent, 6.25 µL of 6.25 µM C-terminally-tagged GFPs in PBS, and 12.5 µL of ubiquitylation master mix in PCR tubes. Reactions were incubated at 30 °C for 90 min then were stopped by the addition of 6.25 µL of 5× SDS-PAGE loading buffer. Samples were heated at 95 °C for 5 min prior to analysis by SDS-PAGE and Western blotting.

Cellular Degradation of C-Terminally-Tagged GFPs

Cellular degradation of C-terminally-tagged GFPs was performed according to the reported procedure.[7] Briefly, cells were grown to 80-90% confluency in FBS-supplemented media without antibiotics (+/-) prior to electroporation. Cells were washed with PBS, then resuspended in PBS and counted. For each sample, an equal number of cells ($7.0\text{-}8.7 \times 10^5$) were aliquoted into a 1.7 mL tube and pelleted. For experiments with competition, Neon buffer R was combined with 100x compound (final concentration 100 µL lenalidomide or 1 µL MLN4924) or an equivalent volume of DMSO. Electroporation mixes were prepared for each sample type by combining 43.75 or 87.5 µL Neon buffer R with compound or DMSO if applicable with 6.1 or 12.5 µL of 50 µM protein in PBS, or PBS for control samples. Immediately prior to each electroporation, PBS was removed from the pelleted cells and the pellet was resuspended in 12 µL of electroporation mix. The sample was taken up into a tip attached to a Neon pipette, and the pipette tip was submerged in a Neon cuvette containing 3 mL Neon buffer E. The sample was then electroporated (1325 V, 10 msec, 3 pulses for Jurkats or 800 V, 25 ms, 2 pulses for HEK293T cells). The cells were then dispensed into 100 µL of warmed PBS. This process was repeated for each sample, with each tip used for 3 electroporation cycles. Cells were then pelleted by centrifugation and the supernatant was removed. Cells were resuspended in 50 µL trypsin-EDTA solution and incubated at 37 °C for 5 min. Trypsinization was quenched by the addition of 0.5 mL +/− media with 1000x compound

stock or DMSO as appropriate, and cells were incubated at 37 °C for 6 hours from the start of electroporation. Cells were collected by centrifugation and washed 1x with PBS.

For analysis of GFP levels, each sample was resuspended in 500 µL PBS with 10 µL 0.5 mg/mL propidium iodide (Jurkats) or 50 nM SYTOX Blue (HEK293T) added to allow for exclusion of dead cells. Cells were analyzed by flow cytometry (mCherry and FITC for Jurkats and BV480-A and FITC for HEK293T on BD FACSyphony A3 Lite). 10,000 events were analyzed for each sample. Relative GFP level was determined by subtracting the arithmetic mean GFP signal among live cells in the control sample from the arithmetic mean GFP signal among live cells for each sample, then normalizing the resulting values to the arithmetic mean for GFP-His_6.

Comparisons were performed using a one-way ANOVA with Šídák's multiple comparisons test. ns = not significant, * = $p < 0.05$, ** = $p < 0.01$, *** = $p < 0.001$, **** = $p < 0.0001$.

Synthetic Procedure

General Reagent Information

All reactions were performed under a positive pressure of argon using the indicated method in general procedures unless otherwise noted. *N,N*-Dimethylformamide (DMF) (Beantown Chemical, catalog no. BT138690) and tetrahydrofuran (THF) (Fisher Chemical, catalog no. T427-4) were vigorously purged with argon for 1 h, followed by passage under argon pressure through two packed columns of neutral alumina (Pure Process Technology). Milli-Q (MQ) water was prepared using a Barnstead™ GenPure™ xCAD Plus Ultrapure Water Purification System (Thermo Fisher Scientific). CD_3OD, DMSO-d_6, $CDCl_3$ and acetone-d_6 were purchased from Cambridge Isotope Laboratories. Minimalist tag was prepared according to the method of Yao and co-workers.[15] The cleavable biotin azide probe was synthesized according to the method of Woo and co-workers.[16] All other commercial solvents and reagents were used as received. Flash Column Chromatography was performed using silica gel purchased from Silicycle (SilicaFlash® F60, 40–63 µm) with the aid of a CombiFlash® NextGen 300+ Automated Flash Chromatography System (Teledyne ISCO). Preparative and analytical HPLC was performed on Waters Prep 150 LC System or Agilent 1260 Infinity II Preparative HPLC System. Reverse phase preparative separation was performed with XBridge BEH C_{18} OBD Prep Column (130 Å, 5 µm, 19 mm x 100 mm) and chiral separation was performed with an indicated column. Organic solutions were concentrated in vacuo using a Buchi rotary evaporator at 30–33 °C. Analytical thin-layer chromatography (TLC) was performed using glass plates pre-coated with silica gel (0.25 mm, 60 Å pore size) containing a fluorescent indicator (254 nm). TLC plates were visualized by exposure to ultraviolet light (UV), iodine (I_2), and/or submersion in *p*-anisaldehyde followed by brief heating with a heat gun (10–15 sec).

General Analytical Information

NMR experiments were performed on a Bruker 400 MHz, Varian 500 MHz, or Agilent 600 MHz instrument at 25 °C. Chemical shifts are reported in parts per million (ppm) relative to residual solvent as an internal reference (CD_2HOD: δ 3.31 ppm for ^1H and δ 49.00 ppm for ^{13}C; DMSO: 2.50 ppm for ^1H and 39.52 ppm for ^{13}C; acetone: δ 2.05 ppm for ^1H and 29.84 ppm for ^{13}C). The following abbreviations were used to explain multiplicities: s = singlet, bs = broad singlet, d = doublet, t = triplet, q = quartet, dd = doublet of doublets, m = multiplet. All ^{13}C NMR spectra were obtained with ^1H decoupling. All IR spectra were recorded on a Bruker ALPHA FT-IR and are reported in terms of frequency of absorption (cm^{-1}) and intensity of absorption (s = strong, m = medium, w = weak, br = broad). High-resolution mass spectra were recorded on a Bruker micro TOF-Q II hybrid quadrupole-time of flight system and on an Agilent 1260 UPLC-MS system. Low-resolution mass spectra were obtained on a Waters ACQUITY UPLC system equipped with SQ Detector 2 mass spectrometer.

General procedure A: Synthesis of the compound **9**

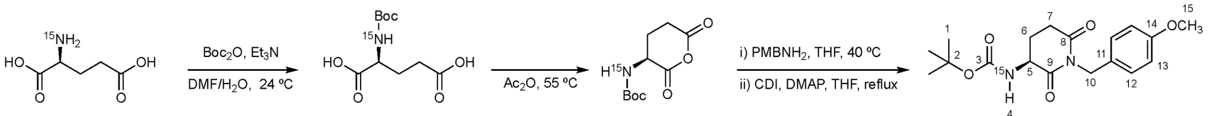

L-glutamic-15*N* acid Boc-*L*-glutamic-15*N* acid **1** **9**

***L*-glutamic-15*N*-acid** (500 mg, 3.38 mmol, 1.00 equiv) was dissolved in *N,N*-dimethylformamide/water (5 mL, 3:2) at 24 °C and triethylamine (936 µL, 4.05 mmol, 2.00 equiv) was added to the stirred solution at 24 °C. To the mixture, Boc_2O (884 mg, 1.20 equiv) was added at 0 °C. The resulting mixture was stirred at 24 °C for 24 h. The reaction was quenched by adding 1*N* hydrochloric acid (20 mL), saturated with sodium chloride and extracted by ethyl acetate (20 mL x 3). Combined organic layer was dried over sodium sulfate and concentrated under vacuum to obtain **Boc-*L*-glutamic-15*N*-acid** as colorless oil (838 mg, 3.38 mmol, quant.) The product was used in the following step without further purification.

Boc-*L*-glutamic-15*N*-acid (839.1 mg, 3.38 mmol, 1.00 equiv) obtained from the last step was dissolved in acetic anhydride (11 mL) and stirred at 55 °C for 15 min. The reaction mixture was concentrated under vacuum to obtain the compound **1** as white solid (720 mg, 3.13 mmol, 95%). The product was used in the following step without further purification.

The compound **1** (500 mg, 2.17 mmol, 1.00 equiv) obtained from the last step was dissolved in tetrahydrofuran (3.6 mL) and added *p*-methoxybenzyl amine (313 mg, 2.28 mmol, 1.05 equiv) at 24 °C. The resulting mixture was stirred at 40 °C for 24 h. The product mixture was cooled down to 24 °C, 1*N* hydrochloric acid was added (20 mL) and extracted by ethyl acetate (20 mL x 3). Combined organic layer was dried over sodium sulfate and concentrated under vacuum. The product mixture was directly used in the following step.

The reaction mixture obtained from the last step was dissolved in tetrahydrofuran (22 mL) and added carbonyldiimidazole (1.06 g, 6.52 mmol, 3.00 equiv) and 4-dimethylaminopyridine (53.1 mg, 0.43 mmol, 0.20 equiv). The resulting mixture was refluxed for 24 h. The product mixture was cooled down to 24 °C, 1*N* hydrochloric acid was added and extracted by ethyl acetate (20 mL x 3). Combined organic layer was dried over sodium sulfate and concentrated under vacuum. The crude mixture was purified by flash-column chromatography (eluting with 20% ethyl acetate–hexanes, grading to 40% ethyl acetate–hexanes, two steps) to afford the compound **9** as white solid (436 mg, 1.25 mmol, 55% in 2 steps).

R_f = 0.25 (40% ethylacetate-hexane; ninhydrin). ^{1}H NMR (500 Hz, chloroform-*d*): δ 7.30 (d, 2H, *J* = 9.0 Hz, H_{12}/H_{13}), 6.80 (d, 2H, *J* = 9.0 Hz, H_{12}/H_{13}), 5.42 (d, 1H, *J* = 90.5 Hz, H_4), 4.89 (d, 1H, *J* = 14.0 Hz, H_{10}), 4.85 (d, 1H, *J* = 14.0 Hz, H_{10}), 4.26 (bs, 1H, H_{11}), 3.77 (s, 3H, H_{15}), 2.85 (ddd, 1H, *J* = 18.5, 5.0, 2.5 Hz, H_7), 2.70 (ddd, 1H, *J* = 18.5, 13.5, 5.0 Hz, H_7), 2.45-2.47 (m, 1H, H_6), 1.77 (ddd, 1H, *J* = 26.5, 13.5, 5.0 Hz, H_6), 1.45 (s, 9H, H_1). ^{13}C NMR (125 Hz, $CDCl_3$): δ ^{13}C NMR (126 MHz, chloroform-*d*) δ 172.0 (C), 171.3 (C), 159.2 (C), 130.6 (CH), 129.2 (C), 113.9 (CH, C), 80.5 (C), 55.4 (O<u>C</u>H_3), 52.7 (CH, d, *J* = 13.25 Hz), 43.3 (CH_2), 32.0 (CH_2, d, *J* = 2.25 Hz), 28.4 (CH_3), 24.9 (CH_2). IR (ATR-FTIR) cm^{-1}: 3350 (br), 2975 (m), 1675 (s), 1513 (s), 1247 (s), 1158 (s). HRMS-ESI (*m/z*): $[M+H]^+$ calculated for $C_{18}H_{25}N^{15}NNaO_5$, 372.1548; found, 372.1571.

<u>General procedure B: Synthesis of the compound **10**</u>

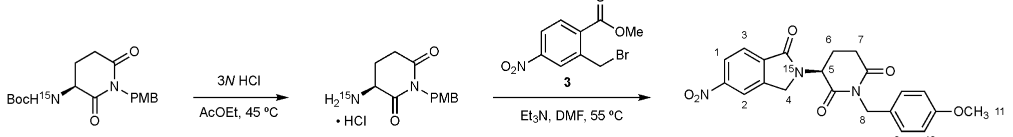

9 **7** **10**

3*N* Hydrochloric acid (2.9 mL, 8.65 mmol, 6 equiv) was added to a stirred solution of the compound **9** (504 mg, 1.44 mmol, 1 equiv) in ethyl acetate (7.2 mL). The reaction mixture was stirred at 45 °C for 24 h. The reaction mixture was concentrated to dryness and the obtained mixture was dissolved in benzene (5.0 mL) and concentrated to dryness to afford **7** as a white solid (412 mg, 1.44 mmol, quant.). The product was used in the following step without further purification.

The compound **3** was prepared according to the method reported by Man and co-workers[17]. The compound **7** (150 mg, 525 μmol, 1.00 equiv) and triethylamine (219 μL, 1.57 mmol, 3.00 equiv) were added in a single portion to a stirred solution of the compound **3** (144 mg, 524 μmol, 1.00 equiv) in *N,N*-dimethylformamide (2.6 mL) at 24 °C. The reaction mixture was stirred for 6 h at 55 °C. The compound **3** (72.0 mg, 262 μmol, 0.50 equiv) was added in a single portion at 24 °C and the reaction mixture was stirred for additional 24 h at 55 °C. The product mixture was diluted with ethyl acetate (10.0 mL) and transferred to a separatory funnel. The diluted product mixture was washed with 1*N* hydrochloric acid (5.0 mL) and the aqueous layer was extracted by ethyl acetate (3 x 5 mL). The combined organic layers were dried over sodium sulfate and concentrated to dryness. The crude product was purified by flash-column chromatography (eluting with 40% ethyl acetate/hexane, grading to 70% ethyl acetate/hexane) to afford the compound **10** as a white solid (146 mg, 335 μmol, 68%).

Rf = 0.20 (50% ethyl acetate/hexane). ^{1}H NMR (500 Hz, chloroform-*d*): 8.36 (d, *J* = 8.3 Hz, 1H, H_1/H_3), 8.32 (s, 1H, H_2), 8.02 (d, *J* = 8.3 Hz, 1H, H_1/H_3), 7.31 (d, *J* = 8.6 Hz, 2H, H_9), 6.79 (d, *J* = 8.6 Hz, 2H, H_{10}), 5.20 (dd, *J* = 13.6, 5.2 Hz, 1H, H_5), 4.88 (s, 2H, H_8), 4.54 (d, *J* = 16.6 Hz, 1H, H_4), 4.42 (d, *J* = 16.6 Hz, 1H, H_4), 3.76 (s, 3H, H_{11}), 3.06 – 2.96 (m, 1H, H_7), 2.87 (ddd, *J* = 17.6, 13.8, 5.5 Hz, 1H, H_7), 2.38 – 2.26 (m, 1H, H_6), 2.23 – 2.14 (m, 1H, H_6).^{13}C NMR (125 Hz, $CDCl_3$): δ ^{13}C NMR (126 MHz, chloroform-*d*) δ 170.8 (C), 169.6 (C), 167.2 (C, d, *J* = 14.9 Hz), 159.2, 150.5, 142.4, 137.0 (d, *J* = 12.1 Hz), 130.7 (CH), 129.0, 125.3, 124.1, 118.8, 113.9 (CH), 55.3 (CH_3), 53.0 (CH, d, *J* = 13.0 Hz), 47.3 (CH_2, d, *J* = 11.3 Hz), 43.4 (CH_2), 32.2 (CH_2, *J* = 2.3 Hz), 22.7. IR (ATR-FTIR) cm^{-1}: 2923 (br), 1676 (s), 1528 (m), 1513 (m), 1344 (s), 1246 (m), 1163 (s), 819 (m), 728 (s). HRMS-ESI (*m/z*): [M+H]$^+$ calculated for $C_{21}H_{20}N_2{}^{15}NO_6$, 411.1317; found, 411.1305.

General procedure C: Synthesis of the compound **11**

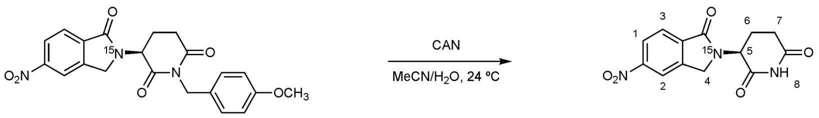

Ammonium cerium(IV) nitrate (975 mg, 1.78 mmol, 5.00 equiv) was added in a single portion to a solution of the compound **10** (146 mg, 356 μmol, 1.00 equiv) in acetonitrile (3.95 mL) and water (790 μL) at 24 °C. The reaction mixture was stirred for 4 h at 24 °C. To the stirred reaction mixture, ammonium cerium(IV) nitrate (195 mg, 356 μmol, 1.00 equiv) was added in a single portion at 24 °C and the reaction mixture was stirred for 4 h at 24 °C. The product mixture was filtrated through celite and the filter cake was washed with methanol (5 mL) and *N,N*-dimethylformamide (5 mL). The filtrate was concentrated to dryness. The crude product was purified by flash-column chromatography (eluting with 5% methanol/dichloromethane, grading to 10% methanol/dichloromethane) to afford the compound **11** as a colorless solid (82.9 mg, 286 μmol, 80%).

^{1}H NMR (400 MHz, dimethylsulfoxide-*d*$_6$) δ 11.04 (s, 1H, H_8), 8.53 (d, *J* = 2.0 Hz, 1H, H_2), 8.36 (dd, *J* = 8.3, 2.0 Hz, 1H, H_1), 7.98 (d, *J* = 8.3 Hz, 1H, H_3), 5.17 (dd, *J* = 13.2, 5.2 Hz, 1H, H_5), 4.61 (d, *J* = 18.1 Hz, 1H, H_4), 4.49 (d, *J* = 18.1 Hz, 1H, H_4), 2.92 (ddd, *J* = 17.2, 13.6, 5.4 Hz, 1H, H_7), 2.64-2.59 (m, 1H, H_7), 2.41 (dddd, *J* = 13.2, 13.2, 13.2, 4.4 Hz, 1H, H_6), 2.05 (dddd, *J* = 13.2, 5.4, 5.4, 2.2 Hz, 1H, H_6). ^{13}C NMR (101 MHz, dimethylsulfoxide -*d*$_6$) δ 172.8 (C),

170.7 (C), 166.2 (d, J = 15.5 Hz, C), 149.8 (C), 143.3 (C), 136.9 (d, J = 11.6 Hz, C), 124.3 (CH), 123.7 (CH), 119.4 (CH), 52.0 (d, J = 12.8 Hz, CH), 47.5 (d, J = 11.1Hz, CH_2), 31.1 (CH_2), 22.3 (CH_2). IR (ATR-FTIR) cm^{-1}: 3080 (br), 1705 (m), 1679 (s), 1664 (s), 1530 (s), 1354 (s), 1335 (s), 1233 (m), 1203 (m), 1183 (m), 822 (m), 731 (s). HRMS-ESI (*m/z*): $[M+Na]^+$ calculated for $C_{13}H_{12}N_2{}^{15}NNaO_5$, 313.0561; found, 313.0551.

General procedure D: Synthesis of the compound **4**

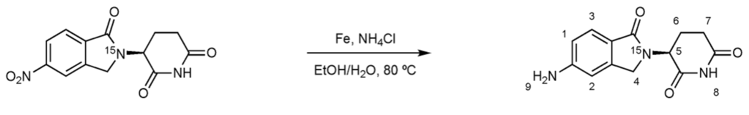

11 **4**

Ammonium chloride (114 mg, 2.12 mmol, 8.00 equiv) was added in a single portion to a solution of the compound **11** (77.0 mg, 265 µmol, 1.00 equiv) in ethanol (3.50 mL) and water (700 µL) at 24 °C. The reaction mixture was stirred for 5 min at 60 °C and iron powder (59.3 mg, 1.06 mmol, 4.00 equiv) was added in a single portion to a stirred reaction mixture at 60 °C. The reaction mixture was stirred for 4 h at 80 °C. The product mixture was cooled down to 24 °C and filtrated through celite. The filter cake was washed with *N,N*-dimethylformamide (5 mL) and the filtrate was concentrated to dryness. The crude product was dissolved in *N,N*-dimethylformamide (5 mL) and filtrated through cotton to remove ammonium chloride. The crude product was concentrated to dryness and purified by flash-column chromatography (eluting with 3% methanol/dichloromethane, grading to 5% methanol/dichloromethane) to afford the compound **4** as a colorless solid (23.0 mg, 88.7 µmol, 33%).

^1H NMR (600 MHz, dimethylsulfoxide -d_6) δ 10.92 (s, 1H, H_8), 7.34 (d, J = 8.7 Hz, 1H, H_1/H_3), 6.64 – 6.58 (m, 2H, H_1/H_3, H_2), 5.81 (s, 2H, H_9), 5.00 (dd, J = 13.2, 4.7 Hz, 1H, H_5), 4.24 (d, J = 16.5 Hz, 1H, H_4), 4.11 (d, J = 16.5 Hz, 1H, H_4), 2.89 (ddd, J = 16.8, 13.2, 5.4 Hz, 1H, H_7), 2.57 (ddd, J = 16.8, 4.4, 2.4 Hz, 1H, H_7), 2.32 (dddd, J = 13.2, 13.2, 13.2, 4.4 Hz, 1H, H_6), 1.93 (dddd, J = 13.2, 5.4, 5.4, 2.4 Hz, 1H, H_6). ^{13}C NMR (101 MHz, dimethylsulfoxide -d_6) δ 172.9 (C), 171.4 (C), 168.7 (d, J = 14.4 Hz, C), 152.6 (C), 144.3 (C), 124.1 (CH), 118.9 (d, J = 12.0 Hz, C), 113.6 (CH), 106.5 (CH), 51.2 (d, J = 13.1 Hz, CH), 46.6 (d, J = 11.6 Hz, CH_2), 31.3 (CH_2), 22.63 (CH_2). IR (ATR-FTIR) cm^{-1}: 3343 (br), 3230 (br), 1655 (s), 1609 (s), 1284 (m), 1230 (m), 1199 (m), 421 (s). HRMS-ESI (*m/z*): $[M+H]^+$ calculated for $C_{13}H_{14}N_2{}^{15}NO_3$, 261.1000; found, 261.0990.

Synthesis of (*S*)-isoindolinone-^{15}*N*-photolenalidomide

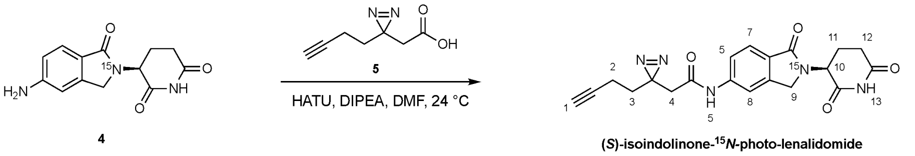

4 **(*S*)-isoindolinone-^{15}*N*-photo-lenalidomide**

(*S*)-isoindolinone-^{15}*N*-photolenalidomide was prepared as previously reported[10] from the compound **4** (17.1 mg, 65.7 µmol, 1.00 equiv), the compound **5** (15.0 mg, 98.6 µmol, 1.50 equiv), HATU (75.0 mg, 197 µmol, 3.00 equiv), diisopropylethylamine (34.3 µL, 197 µmol, 3.00 equiv) in *N,N*-dimethylformamide (82 µL). After purification, the titled compound was obtained as white solid (12.5 mg, 31.7 µmol, 48%). The purified compound was separated in enantiomers with HPLC (CHIRALCEL OD-H, 10x250 mm, 5 µm, isopropanol 100%, flow rate 0.7 mL/min, retention time 14.8 min (enantiomer 1), 28.4 min (enantiomer 2)). Enantiomer 1 was determined as S-enantiomer; $[\alpha]^D{}_{23}$ = -11.2 (c = 0.0025, 98.8% ee, MeCN). Enantiomer 2 was determined as R-enantiomer; $[\alpha]^D{}_{23}$ = +27.5 (c = 0.0018, 99.5% ee, MeCN)

^{1}H NMR (400 MHz, dimethylsulfoxide -d_6) δ 10.97 (s, 1H, H_{13}), 10.28 (s, 1H, H_5), 7.95 (s, 1H, H_8), 7.66 (d, J = 8.2 Hz, 1H, H_7), 7.54 (dd, J = 8.2, 1.8 Hz, 1H, H_5), 5.08 (dd, J = 13.6, 5.0, Hz, 1H, H_{10}), 4.43 (d, J = 17.2 Hz, 1H, H_9), 4.29 (d, J = 17.2 Hz, 1H, H_9), 2.91 (ddd, J = 17.2, 13.6, 5.4 Hz, 1H, H_{12}), 2.84 (t, J = 2.6 Hz, 1H, H_1), 2.63-2.57 (m, 1H, H_{12}), 2.54 (s, 2H, H_4), 2.37 (dddd, J = 13.0, 13.0, 13.0, 4.2 Hz, 1H, H_{11}), 2.07 (td, J = 7.4, 2.6 Hz, 2H, H_2), 2.04 – 1.95 (m, 1H, H_{11}), 1.73 (t, J = 7.4 Hz, 2H, H_3). ^{13}C NMR (101 MHz, dimethylsulfoxide -d_6) δ 172.9 (C), 171.1 (C), 167.7 (d, J = 14.3 Hz, C), 166.9 (C), 143.3 (C), 141.9 (C), 126.6 (d, J = 12.0 Hz, C), 123.7 (CH), 118.8 (CH), 113.4 (CH), 83.1 (C), 71.9 (CH), 51.5 (d, J = 13.3, CH), 47.1 (d, J = 10.7 Hz, CH_2), 40.8 (CH_2), 31.8 (CH_2), 31.2 (CH_2), 26.6 (C), 22.5 (CH_2), 12.6 (CH_2). HRMS-ESI (m/z): $[M+H]^+$ calculated for $C_{20}H_{20}N_4{}^{15}NO_4$, 395.1480; found, 395.1448.

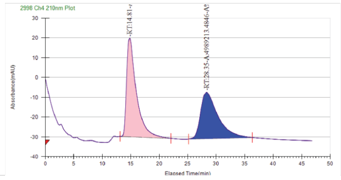

Peak Information

Peak No	% Area	Area	Ret. Time	Height	Cap. Factor
1	54.472	5969347.3761	14.81 min	49.822	14807.3333
2	45.528	4989213.4846	28.35 min	23.5114	28349

enantiomeric mixture

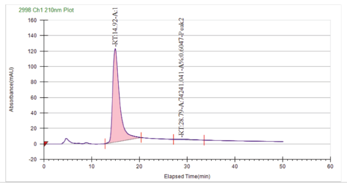

Peak Information

Peak No	% Area	Area	Ret. Time	Height	Cap. Factor
1	99.3953	12203100.9766	14.92 min	120.5467	14915.6667
2	0.6047	74241.041	28.79 min	0.3679	28790.6667

enantiomer 1 (S)

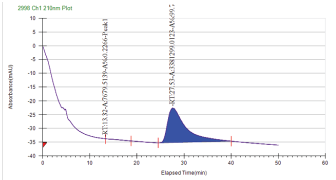

Peak Information

Peak No	% Area	Area	Ret. Time	Height	Cap. Factor
1	0.2266	7679.5139	13.32 min	0	0
2	99.7734	3381299.0123	27.53 min	12.5796	0

enantiomer 2 (R)

Synthesis of the compound **12**

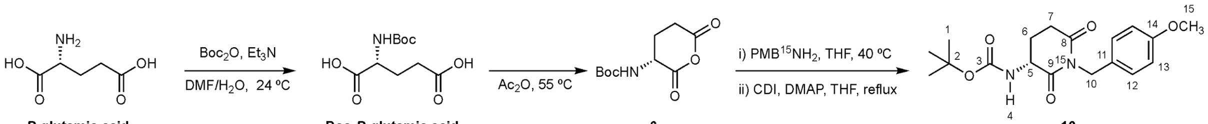

12 was prepared according to the General procedure A *Step 1* from *D*-glutamic acid (1.00 g, 6.75 mmol, 1.0 equiv), di-*tert*-butyl decarbonate (1.77 g, 8.20 mmol, 1.2 equiv) and triethylamine (1.87 mL, 13.5 mmol, 2.0 equiv) in *N,N*-dimethylformamide/water (10 mL, 3:2). After aqueous work up, Boc-*D*-glutamic acid was obtained as colorless oil (1.67 g, 6.75 mmol, quant.). The product was used in the following step without further purification. The compound **6** was prepared according to the General procedure A *Step 2* from Boc-*D*-glutamic acid (1.67 g, 6.75 mmol, 1 equiv) in acetic anhydride (17.0 mL). The compound **6** was obtained as white solid (1.53 g, 6.67 mmol, 99%). The product was used in the following step without further purification. The compound **12** was prepared according to the General procedure A *Step 3* and *4* from the compound **6** (527 mg, 2.30 mmol, 1 equiv), *p*-methoxybenzyl amine-^{15}N (334 mg, 2.42 mmol, 1.05 equiv) in tetrahydrofuran (3.8 mL). After aqueous work up, the obtained product mixture was directly used in the next step. The product mixture obtained from the last step, carbonyldiimidazole (1.12 g, 6.90 mmol, 3 equiv) and 4-dimethylaminopyridine (56.2 mg, 0.46 mmol, 0.2 equiv) in tetrahydrofuran (23 mL). After purification, the compound **12** was obtained as white solid (358 mg, 1.02 mmol, 44% in 2 steps.)

R_f = 0.25 (40% ethylacetate-hexane; ninhydrin). ^{1}H NMR (500 Hz, chloroform-d): δ 7.30 (d, 2H, J = 9.0 Hz, H_{12}/H_{13}), 6.81 (d, 2H, J = 9.0 Hz, H_{12}/H_{13}), 5.42 (bs, 1H, H_4), 4.89 (d, 1H, J = 14.0 Hz, H_{10}), 4.85 (d, 1H, J = 14.0 Hz, H_{10}), 4.26 (bs, 1H, H_{11}), 3.77 (s, 3H, H_{15}), 2.83-2.88 (m, 1H, H_7), 2.70 (ddd, 1H, J = 18.5, 13.5, 5.0 Hz, H_7), 2.45-2.47 (m, 1H, H_6), 1.77 (ddd, 1H, J = 26.5, 13.5, 5.0 Hz, H_6), 1.45 (s, 9H, H_1). δ ^{13}C NMR (126 MHz, chloroform-d) δ 172.0 (C, d, J = 12 Hz), 171.3 (C, d, J = 7.75 Hz), 159.2 (C), 130.6 (CH), 129.1 (C), 113.9 (CH, C), 80.5 (C), 55.4 (O<u>C</u>H_3), 52.7 (CH), 43.3 (CH_2, d, J = 8.5 Hz), 32.0 (CH_2, d, J = 6.13 Hz), 28.4 (CH_3), 24.9 (CH_2). IR (ATR-FTIR) cm^{-1}: 3369 (br), 2975 (m), 1672 (s), 1512 (s), 1246 (s), 1150 (s). HRMS-ESI (m/z): $[M+Na]^+$ calculated for $C_{18}H_{24}N^{15}NNaO_5$, 372.1548; found, 372.1565

Synthesis of the compound **13**

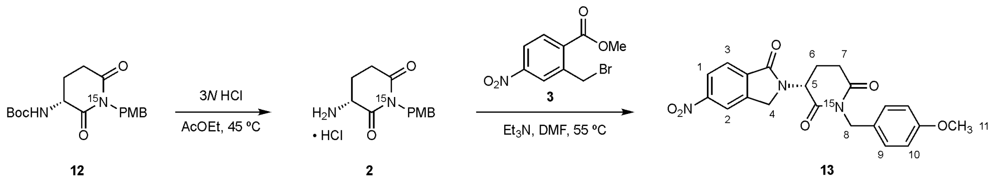

The compound **13** was prepared according to the General procedure B *Step 1* from the compound **12** (358 mg, 1.02 mmol, 1.00 equiv), 3*N* hydrochloric acid (2.0 mL, 6.14 mmol, 6.00 equiv) in ethyl acetate (5.1 mL). After concentration, the compound **2** was obtained as a white solid (293 mg, 1.02 mmol, quant.). The product was used in the following step without further purification. The compound **13** was prepared according to the General procedure *Step 2* from the compound **2** (151 mg, 528 µmol, 1.00 equiv), the compound **3** (218 mg, 792 µmol, 1.50 equiv) and triethylamine (221 µL, 1.58 mmol, 3.00 equiv) in *N,N*-dimethylformamide (2.6 mL). After purification, the compound **13** was obtained as a white solid (137 mg, 332 µmol, 63%).

Rf = 0.20 (50% ethyl acetate/hexane). ^{1}H NMR (399 MHz, chloroform-*d*) δ: 8.36 (d, *J* = 8.3 Hz, 1H, H_1/H_3), 8.32 (s, 1H, H_2), 8.02 (d, *J* = 8.3 Hz, 1H, H_1/H_3), 7.31 (d, *J* = 8.8 Hz, 2H, H_9), 6.79 (d, *J* = 8.8 Hz, 2H, H_{10}), 5.20 (dd, *J* = 13.2, 5.2 Hz, 1H, H_5), 4.88 (s, 2H, H_8), 4.54 (d, *J* = 16.6 Hz, 1H, H_4), 4.42 (d, *J* = 16.6 Hz, 1H, H_4), 3.76 (s, 3H, H_{11}), 3.01 (dddd, *J* = 18.0, 5.2, 5.2, 2.1 Hz, 1H, H_7), 2.88 (ddd, *J* = 18.0, 13.2, 5.2 Hz, 1H, H_7), 2.32 (qd, *J* = 13.2, 13.2, 13.2, 5.2 Hz, 1H, H_6), 2.19 (dddd, *J* = 13.2, 5.2, 5.2, 2.4 Hz, 1H, H_6). ^{13}C NMR (100 MHz, chloroform-*d*) δ 170.8 (C, d, *J* = 7.9 Hz), 169.5 (C, d, *J* = 8.4 Hz), 167.2 (C), 159.2 (C), 150.4 (C), 142.4 (C), 137.0 (C), 130.7 (CH), 128.9 (C), 125.3 (CH), 124.1 (CH), 118.8 (CH), 113.9 (CH), 55.3 (CH_3), 53.0 (CH, d, *J* = 6.4 Hz), 47.3 (CH_2), 43.4 (CH_2, d, *J* = 8.3 Hz), 32.2 (CH_2, d, *J* = 5.8 Hz), 22.7 (CH_2). IR (ATR-FTIR) cm^{-1}: 2919 (br), 1672 (s), 1528 (m), 1512 (m), 1341 (s), 1244 (s), 1156 (s), 818 (s), 729 (s). HRMS-ESI (*m/z*): $[M+Na]^+$ calculated for $C_{21}H_{19}N_2{}^{15}NNaO_6$, 433.1136; found, 433.1120.

Synthesis of the compound **14**

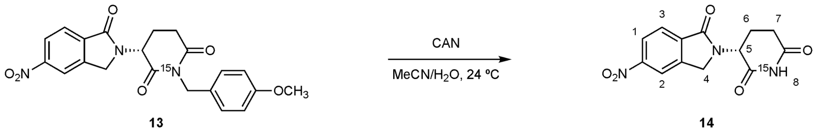

The compound **14** was prepared according to the General procedure C from the compound **13** (137 mg, 334 µmol, 1.00 equiv), ammonium cerium(IV) nitrate (915 mg, 1.67 mmol, 5.00 equiv) acetonitrile (3.7 mL) and water (742 µL). After purification, the compound **14** was obtained as colorless solid (58.0 mg, 200 µmol, 60%).

^{1}H NMR (400 MHz, dimethylsulfoxide-*d*$_6$) δ 11.03 (d, *J* = 87.8 Hz, 1H, H_8), 8.53 (d, *J* = 2.0 Hz, 1H, H_2), 8.36 (dd, *J* = 8.4, 2.0 Hz, 1H, H_1), 7.98 (d, *J* = 8.4 Hz, 1H, H_3), 5.17 (dd, *J* = 13.2, 5.2 Hz, 1H, H_5), 4.61 (d, *J* = 18.1 Hz, 1H, H_4), 4.49 (d, *J* = 18.1 Hz, 1H, H_4), 2.92 (ddd, *J* = 17.2, 13.2, 5.3 Hz, 1H, H_7), 2.63-2.59 (m, 1H, H_7), 2.43 (dddd, *J* = 13.2, 13.2, 13.2, 4.4 Hz, 1H, H_6), 2.05 (dddd, *J* = 13.2, 13.2, 5.2, 2.0 Hz, 1H, H_6). ^{13}C NMR (101 MHz, dimethylsulfoxide -*d*$_6$) δ 172.8 (d, *J* = 9.1 Hz, C), 170.7 (d, *J* = 10.2 Hz, C), 166.2 (C), 149.8 (C), 143.3 (C), 136.9 (C), 124.3 (CH), 123.7 (CH), 119.4 (CH), 52.0 (d, *J* = 6.6 Hz, CH), 47.6 (CH_2), 31.1 (d, *J* = 6.0 Hz, CH_2), 22.3 (CH_2). IR (ATR-FTIR) cm^{-1}: 3110 (br), 1699 (s), 1682 (s), 1530 (m), 1348 (s), 1235 (m), 1181 (s), 821 (m), 730 (m). HRMS-ESI (*m/z*): $[M+Na]^+$ calculated for $C_{13}H_{12}N_2{}^{15}NNaO_5$, 313.0561; found, 313.0548

Synthesis of the compound **8**

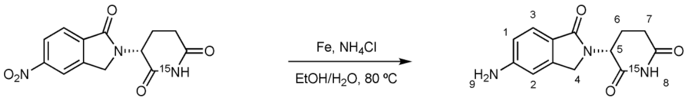

14 **8**

The compound **8** was prepared according to the General procedure D from the compound **14** (53 mg, 183 µmol, 1.00 equiv), ammonium chloride (78.1 mg, 1.46 mmol, 8.00 equiv), iron powder (40.8 mg, 730 µmol, 4.00 equiv) in ethanol (2.4 mL) and water (480 µL). After purification, the compound **8** was obtained as a colorless solid (30.3 mg, 116 µmol, 64%).

^{1}H NMR (400 MHz, dimethylsulfoxide -*d*$_6$) δ 10.91 (d, *J* = 87.7 Hz, 1H, H$_8$), 7.34 (d, *J* = 8.7 Hz, 1H, H$_1$/H$_3$), 6.63-6.61 (m, 2H, H$_2$, H$_1$/H$_3$), 5.80 (bs, 2H, H$_9$), 5.00 (dd, *J* = 13.2, 5.2 Hz, 1H, H$_5$), 4.24 (d, *J* = 16.6 Hz, 1H, H$_4$), 4.11 (d, *J* = 16.6 Hz, 1H, H$_4$), 2.89 (ddd, *J* = 17.2, 13.6, 5.2 Hz, 1H, H$_7$), 2.60-2.55 (m, 1H, H$_7$), 2.32 (dddd, *J* = 13.2, 13.2, 13.2, 4.4 Hz, 1H, H$_6$), 1.93 (dddd, *J* = 13.2, 5.2, 5.2, 2.4 Hz, 1H, H$_6$). ^{13}C NMR (101 MHz, dimethylsulfoxide -*d*$_6$) δ 172. 9 (d, *J* = 9.0 Hz, C), 171.4 (d, *J* = 9.7 Hz, C), 168.7 (C), 152.6 (C), 144.3 (C), 124.1 (CH), 118.9 (C), 113.6 (CH), 106.5 (CH), 51.2 (d, *J* = 6.5 Hz, CH), 46.6 (CH$_2$), 31.2 (d, *J* = 6.0 Hz, CH$_2$), 22.6 (CH$_2$). HRMS-ESI (*m/z*): [M+H]$^+$ calculated for $C_{13}H_{14}N_2{}^{15}NO_3$, 261.1000; found, 261.0984.

Synthesis of (*R*)-glutarimide-^{15}*N*-photolenalidomide

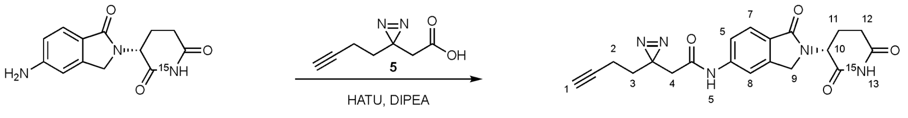

8 **(*R*)-glutarimide-^{15}*N*-photo-lenalidomide**

(*R*)-glutarimide-^{15}*N*-photolenalidomide was prepared as previously reported[10] from the compound **8** (17.1 mg, 65.7 µmol, 1.00 equiv), the compound **5** (15.0 mg, 98.6 µmol, 1.50 equiv), HATU (75.0 mg, 197 µmol, 3.00 equiv), diisopropylethylamine (34.3 µL, 197 µmol, 3.00 equiv) in *N,N*-dimethylformamide (82 µL). After purification, the titled compound was obtained as white solid (14.0 mg, 35.5 µmol, 54%). The purified compound was separated in enantiomers with HPLC (CHIRALCEL OD-H, 10x250 mm, 5 µm, isopropanol 100%, flow rate 0.7 mL/min, retention time 15.4 min (enantiomer 1), 28.5 min (enantiomer 2)).

^{1}H NMR (400 MHz, dimethylsulfoxide -*d*$_6$) δ 11.0 (d, *J* = 87.8 Hz, 1H, H$_{13}$), 10.3 (s, 1H, H$_5$), 7.95 (s, 1H, H$_8$), 7.66 (d, *J* = 8.3 Hz, 1H, H$_7$), 7.54 (dd, *J* = 8.3, 1.8 Hz, 1H, H$_5$), 5.08 (dd, *J* = 13.0, 5.1 Hz, 1H, H$_{10}$), 4.43 (d, *J* = 17.2 Hz, 1H, H$_9$), 4.29 (d, *J* = 17.2 Hz, 1H, H$_9$), 2.91 (ddd, *J* = 17.5, 13.0, 5.4 Hz, 1H, H$_{12}$), 2.84 (t, *J* = 2.7 Hz, 1H, H$_1$), 2.62-2.57 (m, 1H, H$_{12}$), 2.54 (s, 2H, H$_{14}$), 2.37 (dddd, *J* = 13.0, 13.0, 13.0, 4.2 Hz, 1H, H$_{11}$), 2.07 (td, *J* = 7.4, 2.7 Hz, 2H, H$_2$), 2.04 – 1.95 (m, 1H, H$_{11}$), 1.73 (t, *J* = 7.4 Hz, 2H, H$_3$). ^{13}C NMR (101 MHz, dimethylsulfoxide -*d*$_6$) δ 172.9 (d, *J* = 8.9 Hz, C), 171.1 (d, *J* = 9.9 Hz, C), 167.8 (C), 166.9 (C), 143.3 (C), 141.9 (C), 126.6 (C), 123.7 (CH), 118.8 (CH), 113.4 (CH), 83.1 (C), 71.9 (CH), 51.5 (d, *J* = 5.7 Hz, CH), 47.2 (CH$_2$), 40.8 (CH$_2$), 31.9 (CH$_2$), 31.2 (d, *J* = 6.1 Hz, CH$_2$), 26.6 (C), 22.5 (CH$_2$), 12.6 (CH$_2$). HRMS-ESI (*m/z*): [M+H]$^+$ calculated for $C_{20}H_{20}N_4{}^{15}NO_4$, 395.1480; found, 395.1448.

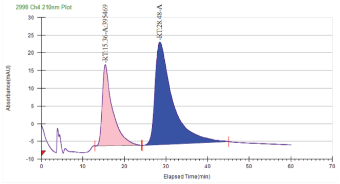

Peak Information

Peak No	% Area	Area	Ret. Time	Height	Cap. Factor
1	31.7185	3954690.7527	15.36 min	22.9215	15357.3333
2	68.2815	8513412.5257	28.48 min	28.9656	28474

enantiomeric mixture

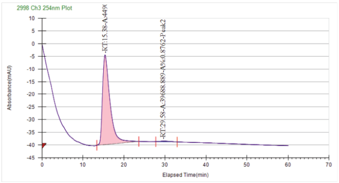

Peak Information

Peak No	% Area	Area	Ret. Time	Height	Cap. Factor
1	99.1238	4490147.0013	15.38 min	35.3314	0
2	0.8762	39688.889	29.58 min	0.233	0

enantiomer 1 (S)

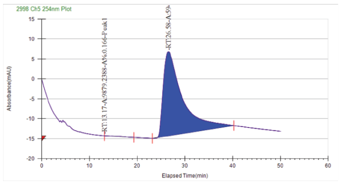

Peak Information

Peak No	% Area	Area	Ret. Time	Height	Cap. Factor
1	0.166	9879.2388	13.17 min	0.0001	13165.6667
2	99.834	5940853.4288	26.58 min	21.0947	26574

enantiomer 2 (R)

General procedure E: Deuteration of lenalidomide

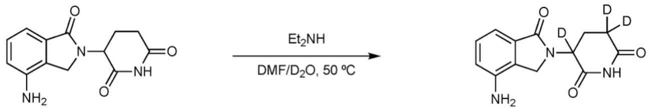

lenalidomiide **lenalidomide-*d*3**

Lenalidomide (10.4 mg, 40.0 µmol, 1.0 equiv) was dissolved in *N'N*-dimethylformamide/deuterium oxide (3.0 mL, 2:1) at 24 °C and diethylamine (42 µL, 400 µmol. 10 equiv) was added in a single portion. The resulting mixture was stirred at 50 °C for 6 hours. The reaction was quenched by cooling down to 24 °C. The reaction mixture was concentrated in vacuo and purified by column chromatography chromatography (ISCO, 4 g column, 0–4% $MeOH/CH_2Cl_2$, 20 min gradient) to yield **lenalidomide-*d*₃** as a white solid (2.0 mg, 7.63 µmol, 19% yield).

^1H NMR (400 MHz, dimethylsulfoxide-d_6) δ 10.99 (s, 1H), 7.19 (dd, *J* = 7.6, 7.6 Hz, 1H), 6.91 (dd, *J* = 7.6, 0.9 Hz, 1H), 6.79 (dd, *J* = 7.6, 0.9 Hz, 1H), 5.41 (s, 2H), 4.20 (d, *J* = 16.9 Hz, 1H), 4.10 (d, *J* = 16.9 Hz, 1H), 2.28 (d, *J* = 12.6 Hz, 1H), 2.01 (d, *J* = 12.6 Hz, 1H). HRMS (ESI) (*m/z*) $[M+H]^+$ calculated for $C_{13}H_{11}D_3N_3O_3$, 263.1218; found, 263.1220.

The purified compound was separated in enantiomers with HPLC (CHIRALPAK IC, 20 mm x 250 mm, 5 µm, ethanol 100%, flow rate 15.0 mL/min, retention time 9.3 min (enantiomer 1, 98.7 %ee), 14.4 min (enantiomer 2, 98.8% ee). With using (*R*)-lenalidomide which was purified with known HPLC conditions,[18] enantiomer 1 was determined as (*S*)-lenalidomide-d_3 and enantiomer 2 was determined as (*R*)-lenalidomide-d_3.

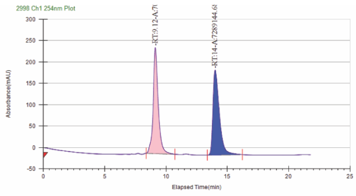

Peak Information

Peak No	% Area	Area	Ret. Time	Height	Cap. Factor
1	49.2545	7074973.4397	9.12 min	248.3876	9124
2	50.7455	7289144.6826	14 min	199.1712	13999

enantiomeric mixture

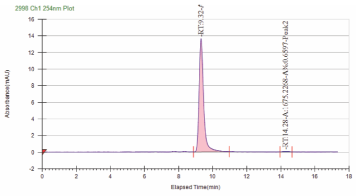

Peak Information

Peak No	% Area	Area	Ret. Time	Height	Cap. Factor
1	99.3403	252256.4555	9.32 min	13.6291	9315.6667
2	0.6597	1675.2268	14.28 min	0.0686	14274

enantiomer 1 (S)

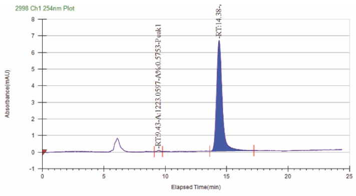

Peak Information

Peak No	% Area	Area	Ret. Time	Height	Cap. Factor
1	0.5753	1223.0597	9.43 min	0.0758	9432.3333
2	99.4247	211361.8918	14.38 min	6.6659	14374

enantiomer 2 (R)

Synthesis of 5-NH_2-EM12-d_3

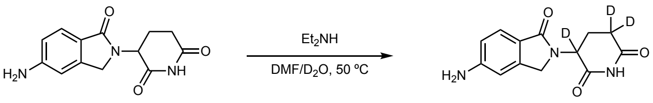

5-NH2-EM12 **5-NH2-EM12-*d*3**

The titled compound was prepared according to General procedure E from 5-NH_2-EM12 (102.1 mg, 393 µmol, 1.0 equiv), diethylamine (204 µL, 1.97 mmol, 5.0 equiv) in a mixture of *N'N*-dimethylformamide/deuterium oxide (6.0 mL, 2:1). After purification by column chromatography (ISCO, 4 g column, 0–4% $MeOH/CH_2Cl_2$, 20 min gradient), the title compound, **5-NH_2-EM12-d_3**, was obtained as a white solid (49.9 mg, 190 µmol, 48% yield).

^1H NMR (600 MHz, dimethylsulfoxide-d_6) δ 10.93 (s, 1H), 7.34 (d, *J* = 9.0 Hz, 1H), 6.61-6.62 (m, 2H), 5.82 (s, 2H), 4.24 (d, *J* = 16.4 Hz, 1H), 4.10 (d, *J* = 16.4 Hz, 1H), 2.30 (d, *J* = 12.6 Hz, 1H), 1.91 (d, *J* = 12.6 Hz, 1H). HRMS (ESI) (*m/z*) $[M+H]^+$ calculated for $C_{13}H_{11}D_3N_3O_3$, 263.1218; found, 263.1220.

The purified compound was separated in enantiomers with HPLC (CHIRALPAK IC, 20 mm x 250 mm, 5 µm, ethanol 100%, flow rate 15.0 mL/min, retention time 10.2 min (enantiomer 1, 98.4 %ee), 19.1 min (enantiomer 2, 99.2% ee). Enantiomer 1 was determined as S-enantiomer; $[\alpha]^{D}_{23}$ = -28.0 (c = 0.0015, 98.0% ee, *N'N*-dimethylformamide). Enantiomer 2 was determined as R-enantiomer; $[\alpha]^{D}_{23}$ = +26.3 (c = 0.0016, 95.0% ee, *N'N*-dimethylformamide).

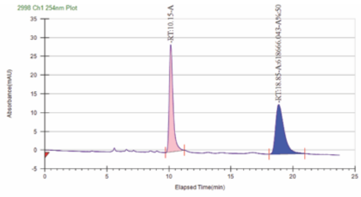

Peak Information

Peak No	% Area	Area	Ret. Time	Height	Cap. Factor
1	49.6817	610838.4215	10.15 min	28.5451	10149
2	50.3183	618666.043	18.85 min	13.1969	18849

enantiomeric mixture

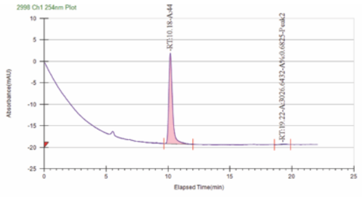

Peak Information

Peak No	% Area	Area	Ret. Time	Height	Cap. Factor
1	99.3175	440414.7351	10.18 min	21.106	10182.3333
2	0.6825	3026.6432	19.22 min	0.0921	19215.6667

enantiomer 1 (S)

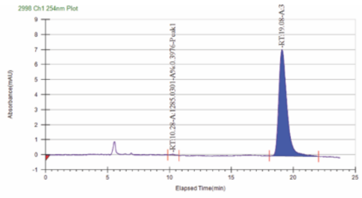

Peak Information

Peak No	% Area	Area	Ret. Time	Height	Cap. Factor
1	0.3976	1285.0301	10.28 min	0.0677	10282.3333
2	99.6024	321905.8612	19.08 min	7.0849	19074

enantiomer 2 (R)

Synthesis of photo-lenalidomide-*d*$_3$

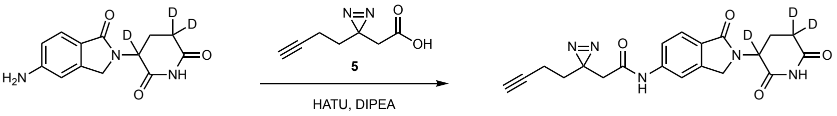

5-NH$_2$-EM12-*d*$_3$ **photo-lenalidomide-*d*$_3$**

Photo-lenalidomide-*d*$_3$ was prepared as previously reported[9] from **5-NH$_2$-EM12-*d*$_3$** (47.0 mg, 179 µmol, 1.00 equiv), the compound **5** (32.7 mg, 215 µmol, 1.20 equiv), HATU (204 mg, 537 µmol, 3.00 equiv), diisopropylethylamine (94 µL, 537 µmol, 3.00 equiv) in *N,N*-dimethylformamide (1.70 mL). After purification by column chromatography (ISCO, 4 g column, 0–6% MeOH/CH_2Cl_2, 30 min gradient) and reverse phase HPLC (flow rate 10 min/mL, 95–5% water/acetonitrile, 27 min gradient, retention time 12.0 min), the titled compound was obtained as a white solid (20.5 mg, 51.7 µmol, 29% yield). The purified compound was separated in enantiomers with HPLC equipped with CHIRALPAK IC (20 mm x 250 mm, 5 µm, ethanol 100%, flow rate 15.0 mL/min, retention time 11.7 min (enantiomer 1), 16.3 min (enantiomer 2)). Each enantiomer was analyzed by CHIRALCEL OD-H (10 mm x 250 mm, 5 µm, isopropanol 100%, flow rate 0.7 mL/min) and enantiomer 1 was determined as (*S*)-photo-lenalidomide-*d*$_3$ (>99.9% ee) and enantiomer 2 was determined as (*R*)-photo-lenalidomide-*d*$_3$ (99.6% ee).

^{1}H NMR (600 MHz, methanol-*d*$_4$) δ 8.03 (s, 1H), 7.74 (d, *J* = 8.3 Hz, 1H), 7.52 (dd, *J* = 8.3, 1.8 Hz, 1H), 4.51 (d, *J* = 17.0 Hz, 1H), 4.45 (d, *J* = 17.0 Hz, 1H), 2.52 (s, 2H), 2.48 (d, *J* = 12.8 Hz, 1H), 2.29 (t, *J* = 2.7 Hz, 1H), 2.15 (d, *J* = 12.8 Hz, 1H), 2.11 (td, *J* = 7.5, 2.7 Hz, 2H), 1.79 (t, *J* = 7.5 Hz, 2H). HRMS (ESI) (*m/z*) $[M+H]^+$ calculated for $C_{20}H_{15}D_3N_5O_4$, 397.1698; found, 397.1702.

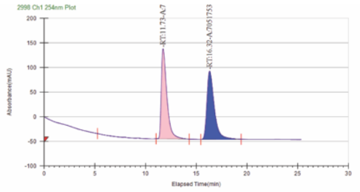

Peak Information

Peak No	% Area	Area	Ret. Time	Height	Cap. Factor
1	49.9548	7039026.1756	11.73 min	183.0133	11732.3333
2	50.0452	7051753.355	16.32 min	138.8942	16315.6667

enantiomeric mixture

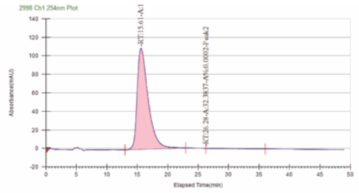

Peak Information

Peak No	% Area	Area	Ret. Time	Height	Cap. Factor
1	99.9998	13842680.7137	15.61 min	109.2106	15607.3333
2	0.0002	32.3837	26.28 min	0.0071	26282.3333

enantiomer 1 (S)

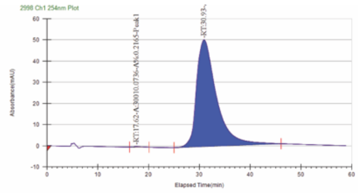

Peak Information

Peak No	% Area	Area	Ret. Time	Height	Cap. Factor
1	0.2165	30010.0736	17.62 min	0.2383	17615.6667
2	99.7835	13833950.018	30.93 min	50.4119	30932.3333

enantiomer 2 (R)

Synthesis of CC885-d_3

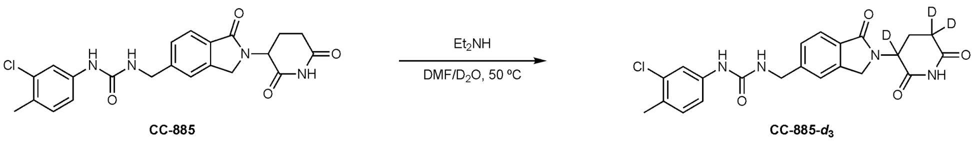

The titled compound was prepared according to General procedure E from CC885 (15.2 mg, 34.5 μmol, 1.0 equiv), diethylamine (35.7 μL, 345 μmol, 10.0 equiv) in a mixture of *N'N*-dimethylformamide/deuterium oxide (2.1 mL, 2:1). After purification by column chromatography (ISCO, 4 g column, 0–4% MeOH/CH_2Cl_2, 20 min gradient), the title compound, **CC885-*d*$_3$**, was obtained as a white solid (2.8 mg, 6.31 μmol, 18% yield).

^{1}H NMR (400 MHz, dimethylsulfoxide-*d*$_6$) δ 10.97 (s, 1H), 8.73 (s, 1H), 7.69 (d, *J* = 7.6 Hz, 1H), 7.66 (d, *J* = 2.0 Hz, 1H), 7.51 (s, 1H), 7.44 (d, *J* = 7.6 Hz, 1H), 7.18 (d, *J* = 8.5 Hz, 1H), 7.13 (dd, *J* = 8.5, 2.0 Hz, 1H), 6.78 (t, *J* = 5.9 Hz, 1H), 4.48 – 4.38 (m, 3H), 4.31 (d, *J* = 17.1 Hz, 1H), 2.36 (d, *J* = 12.6 Hz, 1H), 2.23 (s, 3H), 1.98 (d, *J* = 12.6 Hz, 1H). HRMS (ESI) (*m/z*) $[M+H]^+$ calculated for $C_{22}H_{19}D_3Cl_1N_4O_4$, 444.1512; found, 444.1512.

The purified compound was separated in enantiomers with HPLC (CHIRALPAK IC, 20 mm x 250 mm, 5 μm, ethanol 100%, flow rate 15.0 mL/min, retention time 11.7 min (enantiomer 1, >99.9 %ee), 21.2 min (enantiomer 2, 99.9% ee). Enantiomer 1 was determined as S-enantiomer; $[\alpha]^D{}_{23}$ = -11.0 (c = 0.0025, >99.9% ee, *N'N*-dimethylformamide). Enantiomer 2 was determined as R-enantiomer; $[\alpha]^D{}_{23}$ = +5.56 (c = 0.0027, >99.9% ee, *N'N*-dimethylformamide).

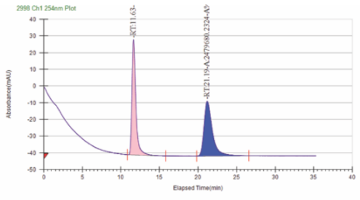

Peak Information

Peak No	% Area	Area	Ret. Time	Height	Cap. Factor
1	49.8165	2461550.2994	11.63 min	69.0893	11632.3333
2	50.1835	2479680.2324	21.19 min	32.8131	21190.6667

enantiomeric mixture

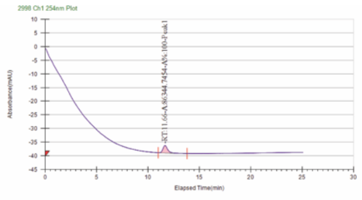

Peak Information

Peak No	% Area	Area	Ret. Time	Height	Cap. Factor
1	100	86344.7454	11.66 min	2.6996	0

enantiomer 1 (S)

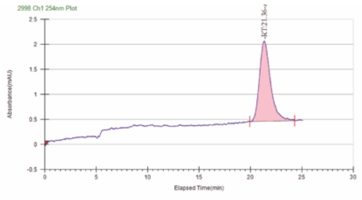

Peak Information

Peak No	% Area	Area	Ret. Time	Height	Cap. Factor
1	100	119570.2584	21.36 min	1.6002	0

enantiomer 2 (R)

General procedure F: Synthesis of JQ1-*rac*-Len

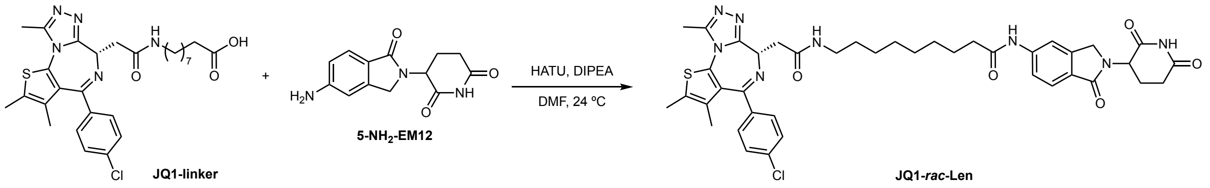

JQ1-linker (10.4 mg, 18.7 μmol, 1.00 equiv) and **5-NH$_2$-EM12** (9.7 mg, 34.7 μmol, 2.00 equiv) were dissolved in dry *N,N*-dimethylformamide (0.098 M). *N,N*-Diisopropylethyl amine (5.00 equiv) and HATU (1.50 equiv) were added in sequence to the stirred reaction mixture. After stirring at 24 °C for 24 h, the reaction mixture was diluted with ethyl acetate and washed sequentially with brine, 10% aqueous citric acid, and saturated aqueous $NaHCO_3$. The organic layer was dried over Na_2SO_4, filtered, and concentrated with the aid of a rotary evaporator. The residue obtained was purified by column chromatography (ISCO, 4 g column, 3–10% MeOH/CH_2Cl_2, 30 min gradient). The fraction obtained was further purified by reverse phase HPLC (flow rate 10 min/mL, 95–5% water/acetonitrile, 27 min gradient, retention time 15.8

mim) to afford **JQ1-*rac*-Len** (3.3 mg, 4.14 µmol, 22% yield).

^{1}H NMR (500 MHz, methanol-d_4) δ 8.05 (s, 1H, H_{18}), 7.72 (d, *J* = 8.0 Hz, 1H), 7.55 (d, *J* = 8.0 Hz, 1H), 7.46 (d, *J* = 8.5 Hz, 2H), 7.41 (d, *J* = 9.0 Hz, 2H), 5.13 (dd, *J* = 13.0, 4.5 Hz, 1H), 4.64 (dd, *J* = 9.0, 5.3 Hz, 1H), 4.50 (d, *J* = 17.0 Hz, 1H), 4.44 (d, *J* = 17.0 Hz, 1H), 3.42 (dd, *J* = 15.0, 9.0 Hz, 1H), 3.39 – 3.21 (m, 3H), 2.91 (ddd, *J* = 16.0, 13.5, 5.5 Hz, 1H), 2.81-2.77 (m, 1H), 2.70 (s, 3H), 2.56 – 2.36 (m, 2H), 2.45 (s, 3H), 2.41 (t, J = 7.5 Hz, 2H), 2.17-2.15 (m, 1H), 1.74-1.71 (m, 2H), 1.70 (s, 3H), 1.64 – 1.52 (m, 2H), 1.40 (bs, 8H). ^{13}C NMR (101 MHz, dimethylsulfoxide-d_6) δ 172.92 (C), 171.79 (C), 171.14 (C), 169.32 (C), 167.86 (C), 162.97 (C), 155.13 (C), 149.80 (C), 143.28 (C), 142.57 (C), 136.73 (C), 135.24 (C), 132.26 (C), 130.71 (C), 130.12 (CH), 129.80 (C), 129.57 (C), 128.44 (CH), 126.09 (C), 123.60 (CH), 118.62 (CH), 113.06 (CH), 53.93 (CH), 51.52 (CH), 47.13 (CH_2), 38.43 (CH_2), 37.67 (CH_2), 36.51 (CH_2), 31.23 (CH_2), 29.23 (CH_2), 28.80 (CH_2), 28.66 (CH_2), 28.63 (CH_2), 26.37 (CH_2), 25.02 (CH_2), 22.54 (CH_2), 14.05 (CH_3), 12.68 (CH_3), 11.29 (CH_3). IR (ATR-FTIR) cm^{-1}; 2925 (bs), 1665 (s), 1543 (s), 1416 (s), 1231 (s), 1193 (s), 731 (s). HRMS (ESI) (*m/z*) [M+H]$^+$ calculated for $C_{41}H_{46}ClN_8O_5S$, 797.2995; found, 797.2990.

Synthesis of JQ1-*rac*-Len-d_3

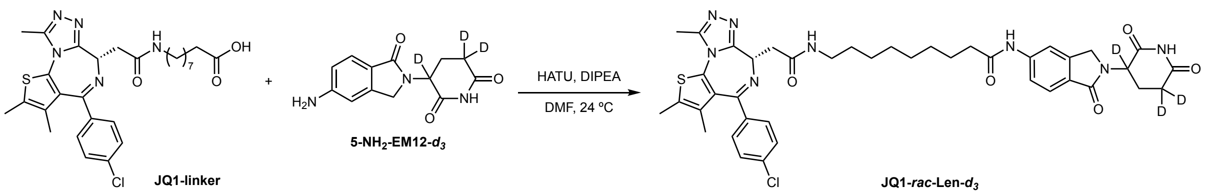

JQ1-*rac*-Len-*d*3 was synthesized according to the General Procedure F from **JQ1-linker** (10.6 mg, 19.1 µmol, 1.00 equiv) and **5-NH2-EM12-*d*3** (5.0 mg, 19.1 µmol, 1.00 equiv), *N,N*-diisopropylethyl amine (17 µL, 95.3 µmol, 5.00 equiv) and HATU (10.9 mg, 28.6 µmol, 1.50 equiv) in dry *N,N*-dimethylformamide (190 µL, 0.10 M). After purification by HPLC (flow rate 10 min/mL, 95–5% water/acetonitrile, 27 min gradient, retention time 15.8 min) the titled compound was obtained as a white solid (4.7 mg, 5.87 µmol, 31% yield).

^{1}H NMR (600 MHz, methanol-d_4) δ 8.04 (s, 1H), 7.71 (d, *J* = 8.4 Hz, 1H), 7.54 (d, *J* = 8.4 Hz, 1H), 7.45 (d, *J* = 8.4 Hz, 2H), 7.40 (d, *J* = 8.4 Hz, 2H), 4.62 (dd, *J* = 8.9, 5.3 Hz, 1H), 4.49 (d, *J* = 17.0 Hz, 1H), 4.43 (d, *J* = 17.0 Hz, 1H), 3.45 – 3.37 (m, 1H), 3.30 – 3.21 (m, 3H), 2.69 (s, 3H), 2.51 – 2.45 (m, 1H), 2.43 (s, 3H), 2.40 (t, *J* = 7.5 Hz, 2H), 2.14 (dd, *J* = 13.0, 4.8 Hz, 1H), 1.72-1.69 (m, 2H), 1.69 (s, 3H), 1.59-1.57 (m, 2H), 1.38 (bs, 8H). HRMS (ESI) (*m/z*) [M+H]$^+$ calculated for $C_{41}H_{43}D_3ClN_8O_5S$, 800.3183; found, 800.3170.

Synthesis of JQ1-(*S*)-Len-d_3

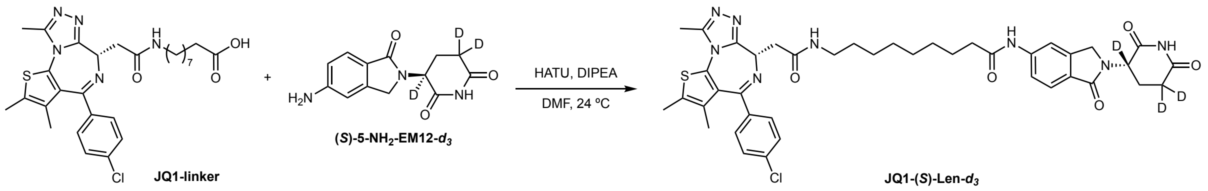

JQ1-(*S*)-Len-*d*3 was synthesized according to the General Procedure F from **JQ1-linker** (10.4 mg, 18.7 µmol, 1.00 equiv) and **(*S*)-5-NH2-EM12-*d*3** (4.9 mg, 18.7 µmol, 1.00 equiv), *N,N*-diisopropylethyl amine (16 µL, 93.4 µmol, 5.00 equiv) and HATU (10.7 mg, 28.0 µmol, 1.50 equiv) in dry *N,N*-dimethylformamide (190 µL, 0.10 M). After purification by HPLC (flow rate 10 min/mL, 95–5% water/acetonitrile, 27 min gradient, retention time 15.8 min) the titled compound was obtained as a white solid (3.4 mg, 4.25 µmol, 23% yield).

^{1}H NMR (600 MHz, methanol-d_4) δ 8.04 (s, 1H), 7.71 (d, *J* = 8.4 Hz, 1H), 7.54 (d, *J* = 8.4 Hz, 1H), 7.45 (d, *J* = 9.0 Hz, 2H), 7.40 (d, *J* = 9.0 Hz, 2H), 4.63 (dd, *J* = 9.0, 5.4 Hz, 1H), 4.48 (d, *J* = 16.8 Hz, 1H), 4.43 (d, *J* = 16.8 Hz, 1H), 3.41 (dd, *J* = 14.8, 8.9 Hz, 1H), 3.30 – 3.20 (m, 3H), 2.69 (s, 3H), 2.44 (d, *J* = 12.6 Hz, 1H), 2.43 (s, 3H), 2.40 (t, *J* = 7.5 Hz, 2H), 2.14 (d, *J* = 12.6 Hz, 1H), 1.72-1.69 (m, 5H), 1.57 (t, *J* = 7.0 Hz, 2H), 1.38 (bs, 8H).

Synthesis of JQ1-(*R*)-Len-d_3

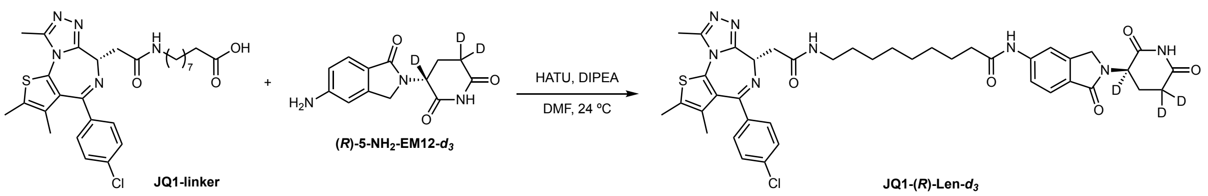

JQ1-(*R*)-Len-d_3 was synthesized according to the General Procedure F from **JQ1-linker** (10.2 mg, 18.3 µmol, 1.00 equiv) and **(*R*)-5-NH$_2$-EM12-d_3** (4.3 mg, 18.3 µmol, 1.00 equiv), *N,N*-diisopropylethyl amine (16 µL, 91.5 µmol, 5.00 equiv) and HATU (10.4 mg, 27.5 µmol, 1.50 equiv) in dry *N,N*-dimethylformamide (183 µL, 0.10 M). After purification by HPLC (flow rate 10 min/mL, 95–5% water/acetonitrile, 27 min gradient, retention time 15.8 min) the titled compound was obtained as a white solid (7.0 mg, 8.75 µmol, 48% yield).

^{1}H NMR (600 MHz, methanol-d_4) δ 8.04 (s, 1H, H20), 7.71 (d, *J* = 8.6 Hz, 1H, H19/H20), 7.54 (d, *J* = 8.6 Hz, 1H, H19/H20), 7.45 (d, *J* = 8.6 Hz, 2H, H2/H3), 7.40 (d, *J* = 8.8 Hz, 2H, H2/H3), 4.62 (dd, *J* = 9.0, 5.4 Hz, 1H, H1), 4.49 (d, *J* = 16.8 Hz, 1H, H21), 4.43 (d, *J* = 16.8 Hz, 1H, H21), 3.41 (dd, *J* = 15.0, 9.0 Hz, 1H, H7), 3.30 – 3.18 (m, 3H, H7, H9), 2.69 (s, 3H), 2.47 (d, *J* = 13.2 Hz, 1H, H23), 2.43 (s, 3H), 2.40 (t, *J* = 7.5 Hz, 2H), 2.14 (d, *J* = 13.2 Hz, 1H, H23), 1.69 (m, 5H), 1.58 (m, 2H), 1.38 (bs, 8H).

Synthesis of JQ1-FepicN

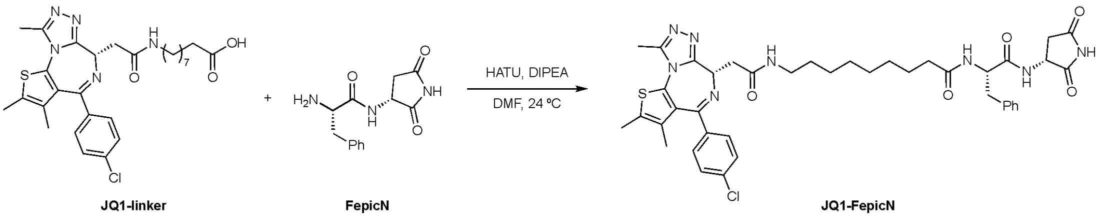

FepicN was prepared as previously reported.[7] **JQ1-linker** (20.6 mg, 37.0 µmol, 1.00 equiv) and **FepicN** (10.6 mg, 40.7 µmol, 1.10 equiv) were dissolved in dry *N,N*-dimethylformamide (0.78 mL, 0.048 M). *N,N*-Diisopropylethyl amine (32 µL, 185 µmol , 5.00 equiv) and HATU (15.5 mg, 40.7 µmol, 1.10 equiv) were added in sequence to the stirred reaction mixture. After stirring at 24 °C for 18–24 h, the reaction mixture was diluted with ethyl acetate and washed sequentially with brine, 10% aqueous citric acid, and saturated aqueous $NaHCO_3$. The organic layer was dried over Na_2SO_4, filtered, and concentrated with the aid of a rotary evaporator. The residue obtained was purified by column chromatography (ISCO, 4 g column, 0–10% MeOH/CH_2Cl_2, 40 min gradient), the title compound, **JQ1-FepicN**, was obtained as a white solid (17.0 mg, 21.3 µmol, 57% yield).

^{1}H NMR (400 MHz, dimethylsulfoxide-d_6) δ 11.23 (br s, 1H, N*H*), 8.58 (d, *J* = 7.8 Hz, 1H, N*H*), 8.15 (t, *J* = 5.6 Hz, 1H, N*H*), 8.04 (d, *J* = 8.5 Hz, 1H, N*H*), 7.48 (d, *J* = 8.9 Hz, 2H), 7.42 (d, *J* = 8.8 Hz, 2H), 7.27–7.19 (m, 4H), 7.19–7.10 (m, 1H), 4.62–4.34 (m, 3H), 3.28–3.04 (m, 4H), 3.00 (dd, *J* = 13.9, 4.8 Hz, 1H), 2.84 (dd, *J* = 17.6, 9.3 Hz, 1H), 2.71 (dd, *J* = 13.8, 10.0 Hz, 1H), 2.59 (s, 3H), 2.41 (s, 3H), 2.33 (dd, *J* = 17.6, 5.4 Hz, 1H), 2.02 (t, *J* = 7.4 Hz, 2H), 1.62 (s, 3H), 1.48–1.39 (m, 2H), 1.37–1.29 (m, 2H), 1.27–1.04 (m, 8H). ^{13}C NMR (101 MHz,

dimethylsulfoxide-d_6) δ 177.4, 176.4, 172.1, 171.7, 169.3, 163.0, 155.1, 149.8, 137.9, 136.7, 135.2, 132.3, 130.7, 130.1, 129.8, 129.6, 129.1, 128.4, 128.0, 126.2, 53.9, 53.5, 49.4, 38.5, 37.7, 37.4, 36.3, 35.2, 29.3, 28.8, 28.7, 28.4, 26.4, 25.1, 14.0, 12.7, 11.3. IR (ATR-FTIR) 3291 (br), 2926 (m), 1725 (s), 1646 (s), 1535 (s), 1419 (m), 1362 (m), 1191 (m). HRMS (ESI) (*m/z*) $[M+H]^+$ calculated for $C_{41}H_{48}ClN_8O_5S$, 799.3151; found, 799.3136.

General Procedure H: Synthesis of Fmoc-GGG-FepicQ

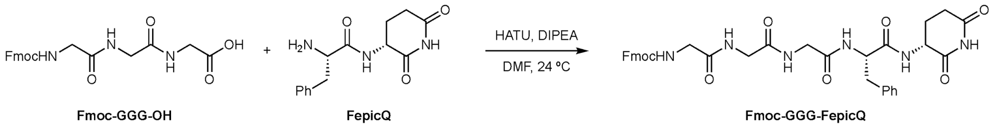

Fmoc-GGG-OH (31.5 mg, 76.6 µmol, 1.00 equiv) and **FepicQ** (21.1 mg, 76.6 µmol, 1.00 equiv) were dissolved in dry *N,N*-dimethylformamide (1.53 mL, 0.050 M). *N,N*-diisopropylethyl amine (67 µL, 383 µmol, 5.00 equiv) and HATU (30.6 mg, 80.5 µmol, 1.05 equiv) were added in sequence to the stirred reaction mixture. After stirring at 24 °C for 18 h, the reaction mixture was concentrated with the aid of a rotary evaporator and the crude material was directly purified by column chromatography (ISCO, 4 g column, 0–15% MeOH/CH_2Cl_2, 30 min gradient). The obtained oil was triturated with diethyl ether (2.0 mL x 3), ethyl acetate (2.0 mL x 3) and acetone (1.0 mL x 3) to afford **Fmoc-GGG-FepicQ** as a white solid (31.3 mg, 46.8 µmol, 61% yield).

^{1}H NMR (400 MHz, dimethylsulfoxide-d_6) δ 10.83 (s, 1H), 8.38 (d, *J* = 8.1 Hz, 1H), 8.16 (d, *J* = 8.4 Hz, 1H), 8.12 (t, *J* = 5.8 Hz, 1H), 8.08 (t, *J* = 5.7 Hz, 1H), 7.89 (d, *J* = 7.5 Hz, 2H), 7.71 (d, *J* = 7.5 Hz, 2H), 7.54 (t, *J* = 6.0 Hz, 1H), 7.42 (dd, *J* = 7.2, 7.2 Hz, 2H), 7.33 (dd, *J* = 7.2, 7.2 Hz, 2H), 7.28 – 7.16 (m, 5H), 4.62 – 4.45 (m, 2H, H14), 4.33 – 4.14 (m, 3H), 3.83 – 3.57 (m, 6H), 2.99 (dd, *J* = 13.6, 5.3 Hz, 1H), 2.80 (dd, *J* = 13.6, 9.1 Hz, 1H), 2.72 – 2.59 (m, 1H), 2.46 – 2.37 (m, 1H), 1.81-1.75 (m, 2H).^{13}C NMR (101 MHz, dimethylsulfoxide-d_6) δ 172.9, 171.9, 170.8, 169.5, 169.1, 168.4, 156.5, 143.8, 140.7, 137.6, 129.2, 128.1, 127.6, 127.1, 126.3, 125.3, 120.1, 65.7, 53.9, 49.0, 46.6, 43.5, 42.0, 41.8, 38.0, 30.6, 24.2. IR (ATR-FTIR) 3369 (br), 1698 (s), 1651 (s), 1544 (m), 1417 (m), 1359 (m), 1282 (s), 1030 (s), 703 (m), 639 (m). HRMS (ESI) (*m/z*) $[M+H]^+$ calculated for $C_{35}H_{37}N_6O_8$, 669.2667; found, 669.2668.

Synthesis of Fmoc-GGG-FepicN

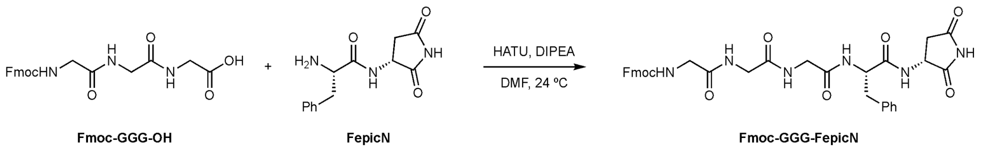

Fmoc-GGG-FepicN was prepared according to General Procedure H from **Fmoc-GGG-OH** (13.0 mg, 31.7 µmol, 1.10 equiv), **FepicN** (7.5 mg, 28.8 µmol, 1.00 equiv), *N,N*-diisopropylethyl amine (25 µL, 144 µmol , 5.00 equiv), and HATU (12.0 mg, 31.7 µmol, 1.10 equiv) in dry *N,N*-dimethylformamide (0.58 mL, 0.050 M). After purification by column chromatography (ISCO, 4 g column, 0–15% MeOH/CH_2Cl_2, 30 min gradient) as well as trituration with diethyl ether (2.0 mL x 3), EtOAc (2.0 mL x 3), and acetone (1.0 mL x 3), **Fmoc-GGG-FepicN** was obtained as a white solid (5.7 mg, 8.71 µmol, 30% yield).

^{1}H NMR (400 MHz, dimethylsulfoxide-d_6) δ 11.25 (br s, 1H, N*H*), 8.58 (d, *J* = 7.8 Hz, 1H, N*H*), 8.24–7.99 (m, 3H, N*H*), 7.89 (d, *J* = 7.5 Hz, 2H), 7.71 (d, *J* = 7.4 Hz, 2H), 7.54 (t, *J* = 6.1 Hz, 1H, N*H*), 7.41 (t, *J* = 7.3 Hz, 2H), 7.32 (t, *J* = 7.3 Hz, 2H), 7.29–7.16 (m, 5H), 4.53–4.38 (m, 2H), 4.34–4.15 (m, 3H), 3.79–3.58 (m, 6H), 3.01 (dd, *J* = 13.7, 5.1 Hz, 1H), 2.90–2.72 (m, 2H), 2.31 (dd, *J* = 17.8, 5.2 Hz, 1H). ^{13}C NMR (101 MHz, dimethylsulfoxide-d_6) δ 177.3, 176.3, 171.2, 169.5, 169.1, 168.5, 156.5, 143.8, 140.7, 137.6, 129.2, 128.1, 127.6, 127.1, 126.3,

125.3, 120.1, 65.7, 53.8, 49.4, 46.6, 43.5, 42.0, 41.8, 37.4, 36.3. IR (ATR-FTIR) 3304 (br), 1719 (s), 1656 (s), 1535 (s), 1249 (m), 1197 (m). HRMS (ESI) (*m/z*) $[M+NH_4]^+$ calculated for $C_{34}H_{38}N_7O_8$, 672.2756; found, 672.2776.

Synthesis of Thal-FITC

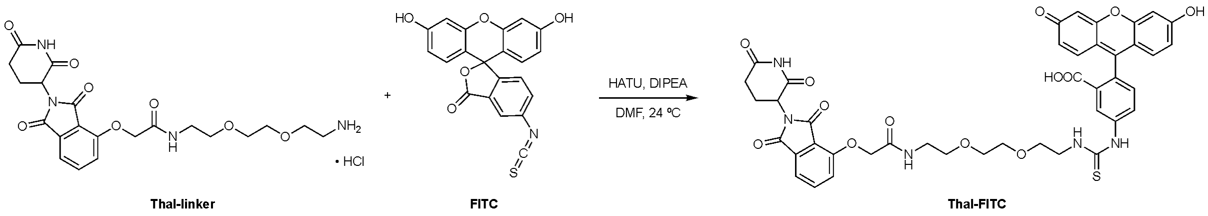

N-[2-[2-(2-Aminoethoxy)ethoxy]ethyl]-2-[[2-(2,6-dioxo-3-piperidinyl)-2,3-dihydro-1,3-dioxo-1*H*-isoindol-4-yl]oxy]acetamide hydrochloride (**Thal-linker**, 11.7 mg, 25.0 µmol, 1.25 equiv) was dissolved in *N,N*-dimethylformamide (500 µL) with *N,N*-diisopropylethyl amine (35 µL, 26 mg, 200 µmol, 10.0 equiv) then 5(6)-fluorescein isothiocyanate (**FITC**, 8.0 mg, 20.0 µmol, 1.00 equiv). The reaction mixture was briefly vortexed and allowed to stand at room temperature for 10 min. The product was directly purified via reverse phase flash chromatography to obtain the titled compound as a yellow powder (11.6 mg, 13.4 µmol, 67%).

^{1}H NMR (400 MHz, dimethylsulfoxide-d_6) δ 11.13 (s, 1H), 10.38–9.83 (m, 3H), 8.27 (s, 1H), 8.12 (s, 1H), 8.03 (t, J = 5.4 Hz, 1H), 7.84–7.77 (m, 1H), 7.73 (d, J = 7.8 Hz, 1H), 7.49 (d, J = 7.2 Hz, 1H), 7.39 (d, J = 8.5 Hz, 1H), 7.17 (d, J = 8.3 Hz, 1H), 6.67 (d, J = 2.0 Hz, 2H), 6.62–6.54 (m, 4H), 5.11 (dd, J = 12.9, 5.4 Hz, 1H), 4.79 (s, 2H), 3.67 (s, 2H), 3.63–3.53 (m, 6H), 3.48 (t, J = 5.6 Hz, 2H), 3.16 (s, 1H), 2.96–2.82 (m, 1H), 2.65–2.51 (m, 2H), 2.10–2.00 (m, 2H). ^{13}C NMR (101 MHz, dimethylsulfoxide -d_6) δ 180.53, 172.80, 169.90, 168.53, 166.95, 166.74, 165.47, 159.51, 154.98, 151.88, 141.34, 136.95, 133.05, 129.06, 126.56, 124.08, 120.33, 116.76, 116.31, 116.07, 112.60, 109.72, 102.23, 69.62, 68.86, 68.45, 67.49, 48.81, 48.61, 43.69, 38.40, 30.96, 22.02. MS (ESI$^{+/-}$) m/z $(M+H)^+$ 852.37, m/z $(M-H)^-$ 850.42, [calculated $C_{42}H_{37}N_5O_{13}S$: 851.21].

Synthesis of HCl•H_2N-GGG-AcN

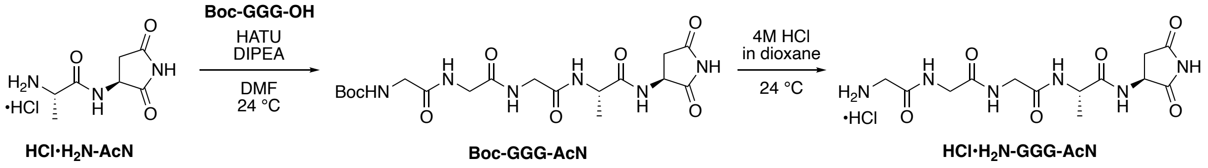

To a solution of **Boc-GGG-OH** (9.5 mg, 33.0 µmol, 1.00 equiv) and **HCl•H$_2$N-AcN**[7] (8 mg, 36.0 µmol, 1.09 equiv) in anhydrous *N,N*-dimethylformamide (2.0 mL), HATU (14 mg, 36.0 µmol, 1.09 equiv) and *N,N*-diisopropylethyl amine (29 µL, 165 µmol, 5.00 equiv) were added at 24 °C. After stirring at 24 °C for 18 h, the reaction mixture was quenched by adding acetic acid (10 µL) and directly purified via semi-preparative HPLC to yield **Boc-GGG-AcN** as a white solid (10 mg, 21.9 µmol, 66%).

^{1}H NMR (400 MHz, DMSO) δ 11.22 (s, 1H), 8.42 (d, *J* = 7.8 Hz, 1H), 8.08 (q, *J* = 6.6 Hz, 3H), 6.98 (t, *J* = 5.8 Hz, 1H), 4.46 (ddd, *J* = 9.3, 7.8, 5.5 Hz, 1H), 4.25 (p, *J* = 7.2 Hz, 1H), 3.81–3.70 (m, 4H), 3.58 (d, *J* = 6.0 Hz, 2H), 2.92–2.81 (m, 1H), 2.43 (dd, *J* = 17.5, 5.5 Hz, 1H), 2.36–2.31 (m, 1H), 1.38 (s, 10H), 1.21 (d, *J* = 7.2 Hz, 4H).

To **Boc-GGG-AcN** (10 mg, 21.9 µmol), 4M HCl in dioxane (2.0 mL, 8.0 mmol) was added. After stirring at 24 °C for 2.5 h, the reaction mixture was concentrated with the aid of a rotary

evaporator and triturated with MeOH and diethyl ether three times, dried under high vacuum, yielding **HCl·NH$_2$-GGG-AcN** as a white solid (4.5 mg, 11.5 µmol, 52%).

^{1}H NMR (400 MHz, methanol-d_4) δ 4.54 (dd, *J* = 9.4, 5.6 Hz, 1H), 4.40 (q, *J* = 7.2 Hz, 1H), 4.04–3.90 (m, 3H), 3.86 (d, *J* = 16.7 Hz, 1H), 3.79 (d, *J* = 2.1 Hz, 2H), 2.99 (dd, *J* = 17.9, 9.3 Hz, 1H), 2.68 (dd, *J* = 17.9, 5.6 Hz, 1H), 1.39 (d, *J* = 7.2 Hz, 3H). ^{13}C NMR (101 MHz, methanol-d_4) δ 179.13, 178.06, 175.26, 172.07, 171.33, 168.41, 51.48, 50.18, 49.71, 49.64, 49.42, 49.21, 49.00, 48.79, 48.57, 48.36, 43.70, 43.57, 41.59, 36.97, 17.65. HRMS (ESI) (m/z) [M+H]$^+$ calculated for $C_{13}H_{21}N_6O_6$, 357.1517; found, 357.1524.

Synthesis of HCl•H$_2$N-GGG-AepicN

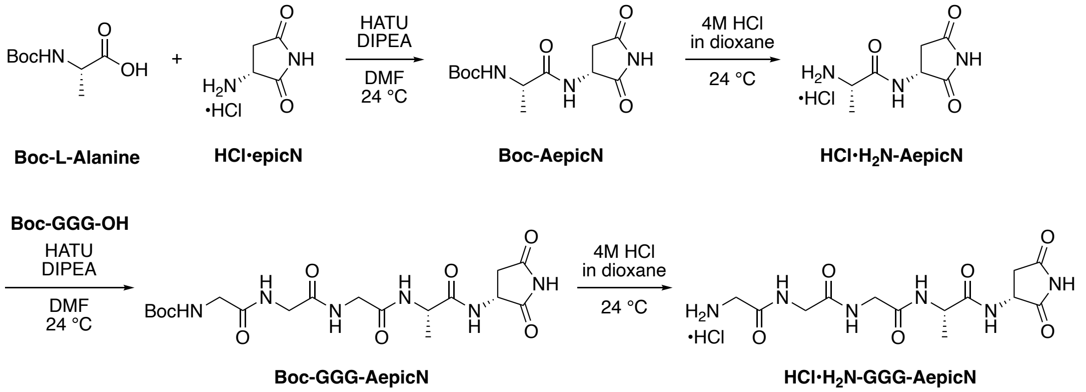

To a solution of **Boc-L-Alanine** (89 mg, 470 µmol, 1.00 equiv) and **HCl•epicN** (78 mg, 518 µmol, 1.10 equiv) in anhydrous *N,N*-dimethylformamide (2.0 mL), HATU (198 mg, 518 µmol, 1.10 equiv) and *N,N*-diisopropylethyl amine (452 µL, 2.6 mmol, 5.53 equiv) were added at 24 °C. After stirring at 24 °C for 15 h, the reaction mixture was concentrated with the aid of a rotary evaporator and the crude material was directly purified by column chromatography (10% MeOH/CH_2Cl_2) to yield **Boc-AepicN** as a white solid. To **Boc-AepicN**, 4M HCl in dioxane (2.0 mL, 8.0 mmol) was added. After stirring at 24 °C for 2.5 h, the reaction mixture was concentrated with the aid of a rotary evaporator and triturated with MeOH and diethyl ether three times, dried under high vacuum, yielding **HCl•H$_2$N-AepicN** as a white solid (45 mg, 203 µmol, 43%).
^{1}H NMR (400 MHz, dimethylsulfoxide-d_6) δ 11.35 (s, 1H), 9.02 (d, J = 7.5 Hz, 1H), 8.11 (s, 3H), 4.53 (ddd, J = 9.3, 7.5, 5.5 Hz, 1H), 4.09 (s, 1H), 3.86 (d, J = 7.3 Hz, 1H), 3.17 (s, 3H), 2.93 (dd, J = 17.6, 9.3 Hz, 1H), 2.58–2.46 (m, 1H), 1.35 (d, J = 7.0 Hz, 3H).

To a solution of **Boc-GGG-OH** (24 mg, 82.2 µmol, 1.00 equiv) and **HCl•H$_2$N-AepicN** (20 mg, 90.0 µmol, 1.09 equiv) in anhydrous *N,N*-dimethylformamide, HATU (34 mg, 90.0 µmol, 1.09 equiv) and *N,N*-diisopropylethyl amine (72 µL, 401 µmol, 4.88 equiv) were added at 24 °C. After stirring at 24 °C for 18 h, the reaction mixture was quenched by adding acetic aci d(24 µL) and purified via semi-preparative HPLC to yield **Boc-GGG-AepicN** as a white solid (26 mg, 57.0 µmol, 69%).
^{1}H NMR (400 MHz, dimethylsulfoxide-d_6) δ 11.22 (s, 1H), 8.45 (d, *J* = 7.8 Hz, 1H), 8.07 (d, *J* = 7.1 Hz, 3H), 6.98 (t, *J* = 5.9 Hz, 1H), 4.43 (ddd, *J* = 9.3, 7.7, 5.5 Hz, 1H), 4.22 (p, *J* = 7.2 Hz, 1H), 3.73 (t, *J* = 5.0 Hz, 4H), 3.57 (d, *J* = 6.0 Hz, 2H), 3.17 (d, *J* = 5.2 Hz, 1H), 2.91–2.80 (m, 1H), 2.42 (dd, *J* = 17.5, 5.5 Hz, 1H), 1.38 (s, 8H), 1.21 (d, *J* = 7.2 Hz, 3H).

To **Boc-GGG-Aepic**N (26 mg, 57.0 µmol), 4M HCl in dioxane (2.0 mL, 8.0 mmol) was added. After stirring at 24 °C for 2.5 h, the reaction mixture was concentrated with the aid of a rotary evaporator and triturated with MeOH and diethyl ether three times, dried under high vacuum, yielding **HCl• H$_2$N-GGG-AepicN** as a white solid (20 mg, 56.1 µmol, 98%).

^{1}H NMR (400 MHz, methanol-d_4) δ 4.66–4.56 (m, 1H), 4.36 (q, *J* = 7.2 Hz, 1H), 4.01–3.91 (m, 3H), 3.85 (d, *J* = 16.7 Hz, 1H), 3.81 (s, 2H), 3.00 (dd, *J* = 17.9, 9.4 Hz, 1H), 2.67 (dd, *J* = 17.9, 5.7 Hz, 1H), 1.40 (d, *J* = 7.3 Hz, 3H). ^{13}C NMR (101 MHz, methanol-d_4) δ 179.10, 178.17, 175.35, 171.99, 171.46, 168.44, 51.33, 50.33, 49.71, 49.64, 49.43, 49.21, 49.00, 48.79, 48.58, 48.36, 43.78, 43.68, 43.52, 41.68, 37.24, 36.95, 17.64. HRMS (ESI) (m/z) $[M+H]^+$ calculated for $C_{13}H_{21}N_6O_6$, 357.1517; found, 357.1531.

REFERENCES

1. E. R. Watson, S. J. Novick, M. E. Matyskiela, P. P. Chamberlain, A. H. de la Peña, J.-Y. Zhu, E. Tran, P. R. Griffin, I. E. Wertz and G. C. Lander, *Science*, 2022, **378**, 549-553.
2. N. C. Payne, A. S. Kalyakina, K. Singh, M. A. Tye and R. Mazitschek, *Nat Chem Biol*, 2021, **17**, 1168-1177.
3. T. V. Nguyen, J. E. Lee, M. J. Sweredoski, S. J. Yang, S. J. Jeon, J. S. Harrison, J. H. Yim, S. G. Lee, H. Handa, B. Kuhlman, J. S. Jeong, J. M. Reitsma, C. S. Park, S. Hess and R. J. Deshaies, *Mol Cell*, 2016, **61**, 809-820.
4. S. M. Park, D. K. Miyamoto, G. Y. Q. Han, M. Chan, N. M. Curnutt, N. L. Tran, A. Velleca, J. H. Kim, A. Schurer, K. Chang, W. Xu, M. G. Kharas and C. M. Woo, *Cancer Cell*, 2023, **41**, 726-739 e711.
5. S. Ichikawa, N. C. Payne, W. Xu, C.-F. Chang, S. Frome, H. A. Flaxman, R. Mazitschek and C. M. Woo, *Cell Chem Bio.* 2024, **31**, 1162-1175.e10.
6. R. P. Nowak, S. L. DeAngelo, D. Buckley, Z. He, K. A. Donovan, J. An, N. Safaee, M. P. Jedrychowski, C. M. Ponthier, M. Ishoey, T. Zhang, J. D. Mancias, N. S. Gray, J. E. Bradner and E. S. Fischer, *Nat Chem Biol*, 2018, **14**, 706-714.
7. S. Ichikawa, H. A. Flaxman, W. Xu, N. Vallavoju, H. C. Lloyd, B. Wang, D. Shen, M. R. Pratt and C. M. Woo, *Nature*, 2022, **610**, 775-782.
8. D. K. Miyamoto, H. A. Flaxman, H. Y. Wu, J. Gao and C. M. Woo, *ACS Chem Biol*, 2019, **14**, 2527-2532.
9. M. Sonnett, E. Yeung and M. Wuhr, *Anal Chem*, 2018, **90**, 5032-5039.
10. Z. Lin, Y. Amako, F. Kabir, H. A. Flaxman, B. Budnik and C. M. Woo, *J Am Chem Soc*, 2022, **144**, 606-614.
11. H. J. Merker, W. Heger, K. Sames, H. Stürje and D. Neubert, *Arch Toxicol*, 1988, **61**, 165-179.
12. X. D. Zhang, *Genomics*, 2007, **89**, 552-561.
13. W. E. Johnson, C. Li and A. Rabinovic, *Biostatistics*, 2007, **8**, 118-127.
14. W. Huber, A. von Heydebreck, H. Sultmann, A. Poustka and M. Vingron, *Bioinformatics (Oxford, England)*, 2002, **18 Suppl 1**, S96-104.
15. Z. Li, P. Hao, L. Li, C. Y. Tan, X. Cheng, G. Y. Chen, S. K. Sze, H. M. Shen and S. Q. Yao, *Angew Chem Int Ed Engl*, 2013, **52**, 8551-8556.
16. J. Gao, A. Mfuh, Y. Amako and C. M. Woo, *J Am Chem Soc*, 2018, **140**, 4259-4268.
17. H.-W. Man, G. W. Muller, A. L. Ruchelman, E. M. Khalil, R. S.-C. Chen, W. Zhang, Arylmethoxy isoindoline derivatives and compositions comprising and methods of using the same. US2011/0196150A1, 2011.
18. Z. I. Szabo, M. Foroughbakhshfasaei, R. Gal, P. Horvath, B. Komjati, B. Noszal and G. Toth, *J Sep Sci*, 2018, **41**, 1414-1423.

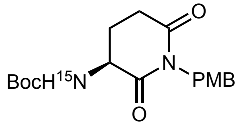

9

^{1}H NMR (500 MHz, $CDCl_3$)

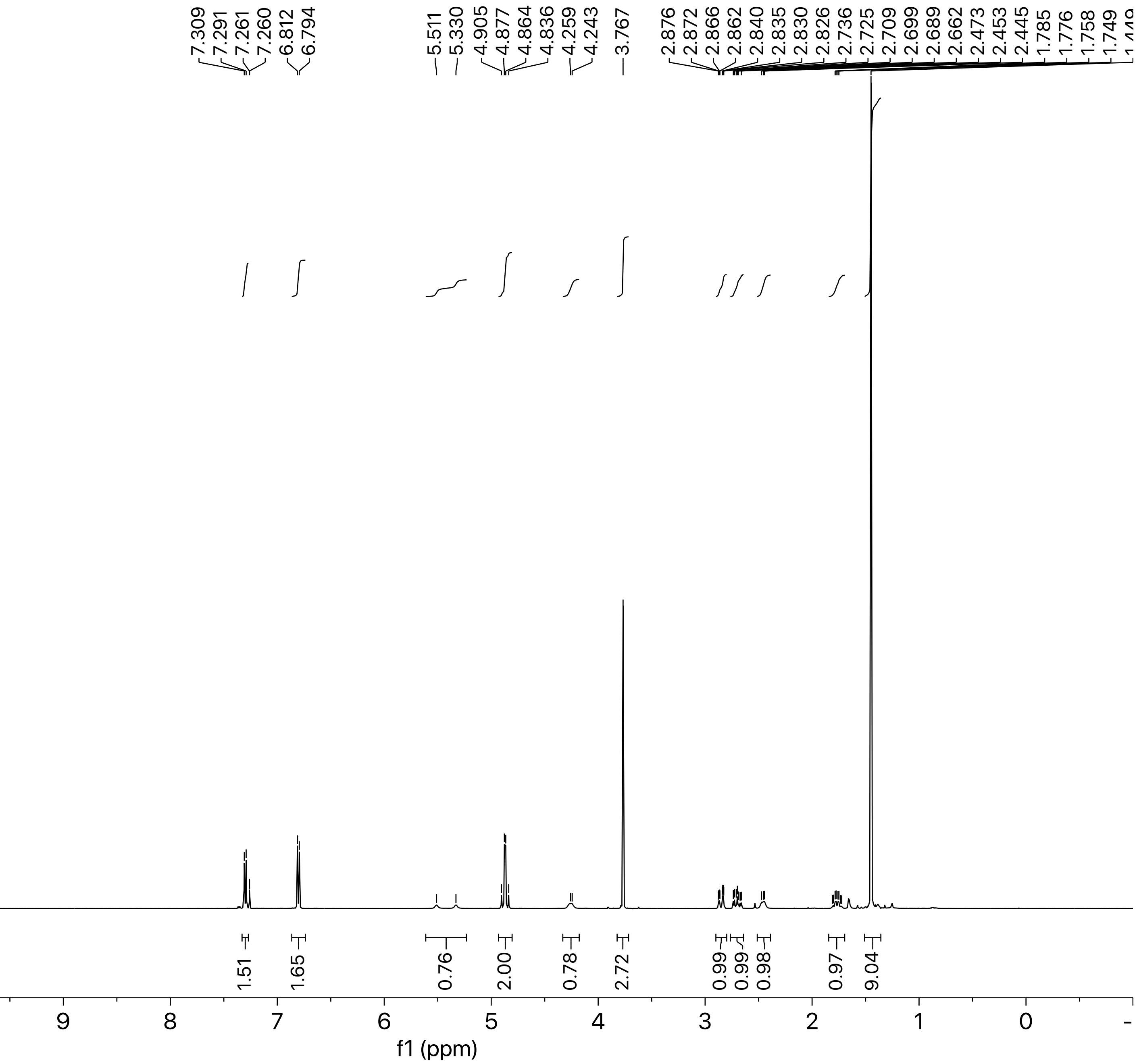

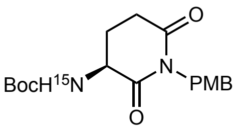

9

^{13}C NMR (125 MHz, $CDCl_3$)

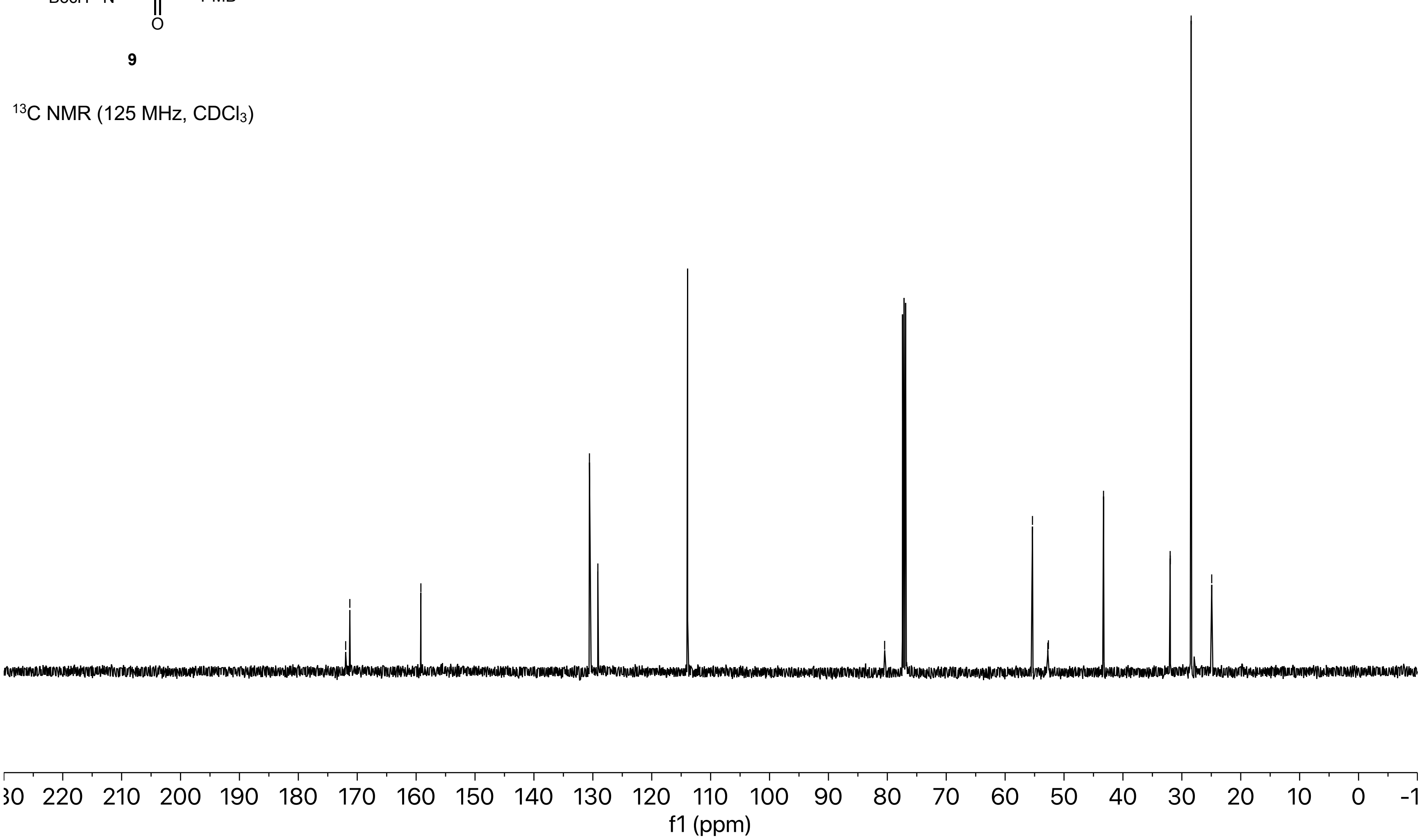

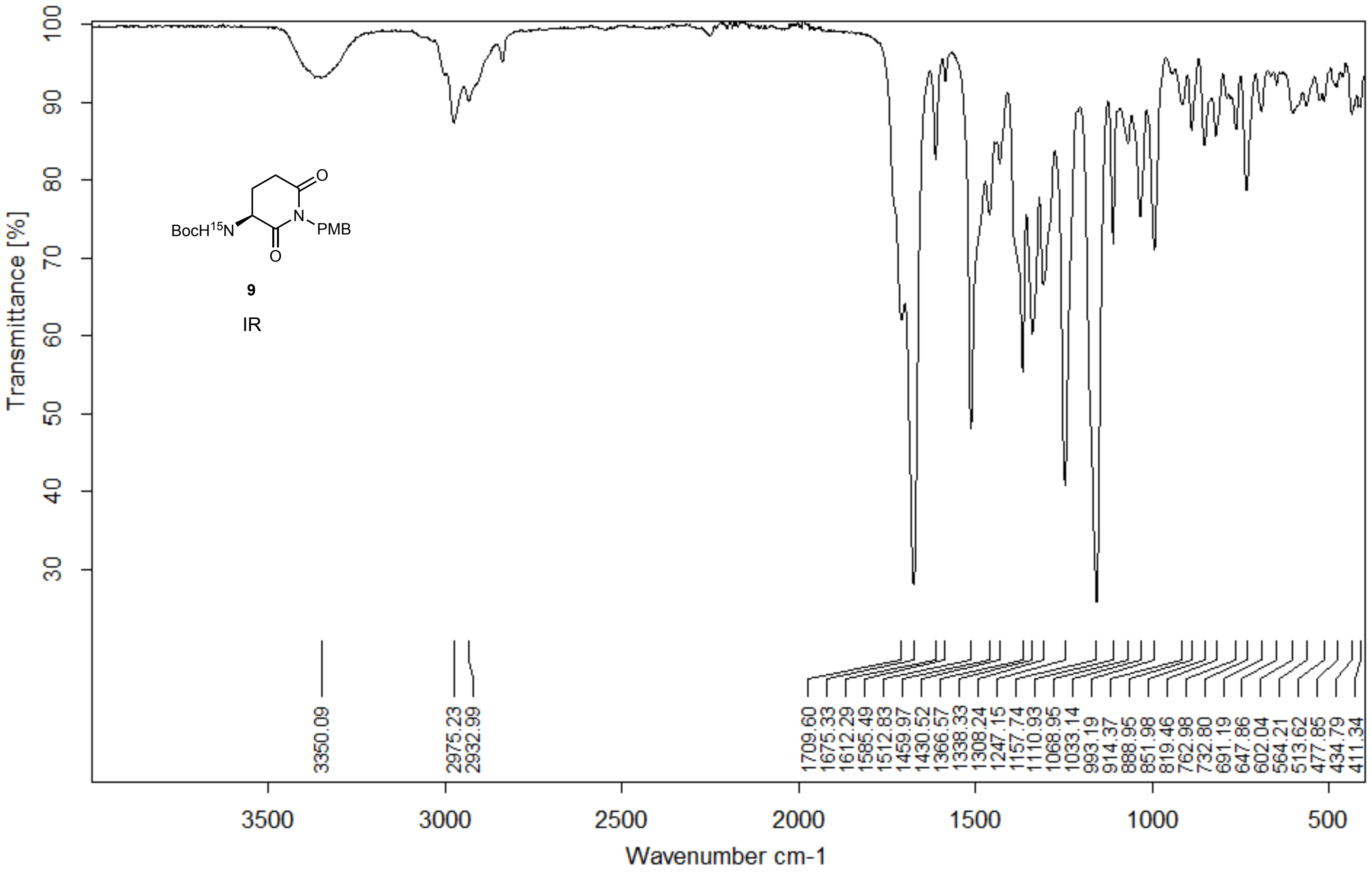

Z:\Alpha FTIR Data\Woo\Yuka\YA139.0	YA139	Diamond ATR	08/12/2018

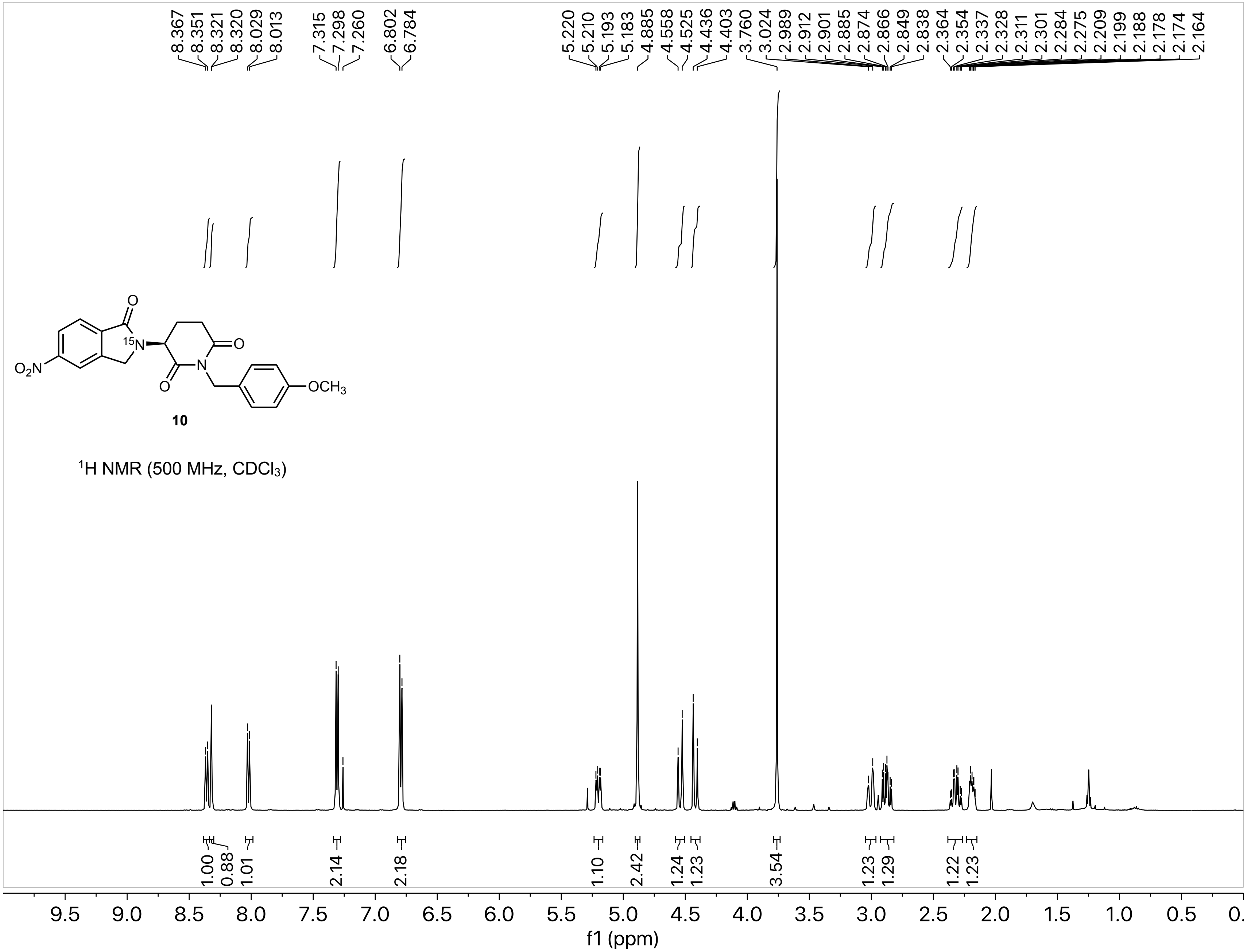

8.367
8.351
8.321
8.320
8.029
8.013
7.315
7.298
7.260
6.802
6.784
5.220
5.210
5.193
5.183
4.885
4.558
4.525
4.436
4.403
3.760
3.024
2.989
2.912
2.901
2.885
2.874
2.866
2.849
2.838
2.364
2.354
2.337
2.328
2.311
2.301
2.284
2.275
2.209
2.199
2.188
2.178
2.174
2.164
O
15N
O
O2N
O
N
OCH3
10
1H NMR (500 MHz, CDCl3)
1.00
0.88
1.01
2.14
2.18
1.10
2.42
1.24
1.23
3.54
1.23
1.29
1.22
1.23
9.5
9.0
8.5
8.0
7.5
7.0
6.5
6.0
5.5
5.0
4.5
4.0
3.5
3.0
2.5
2.0
1.5
1.0
0.5
f1 (ppm)

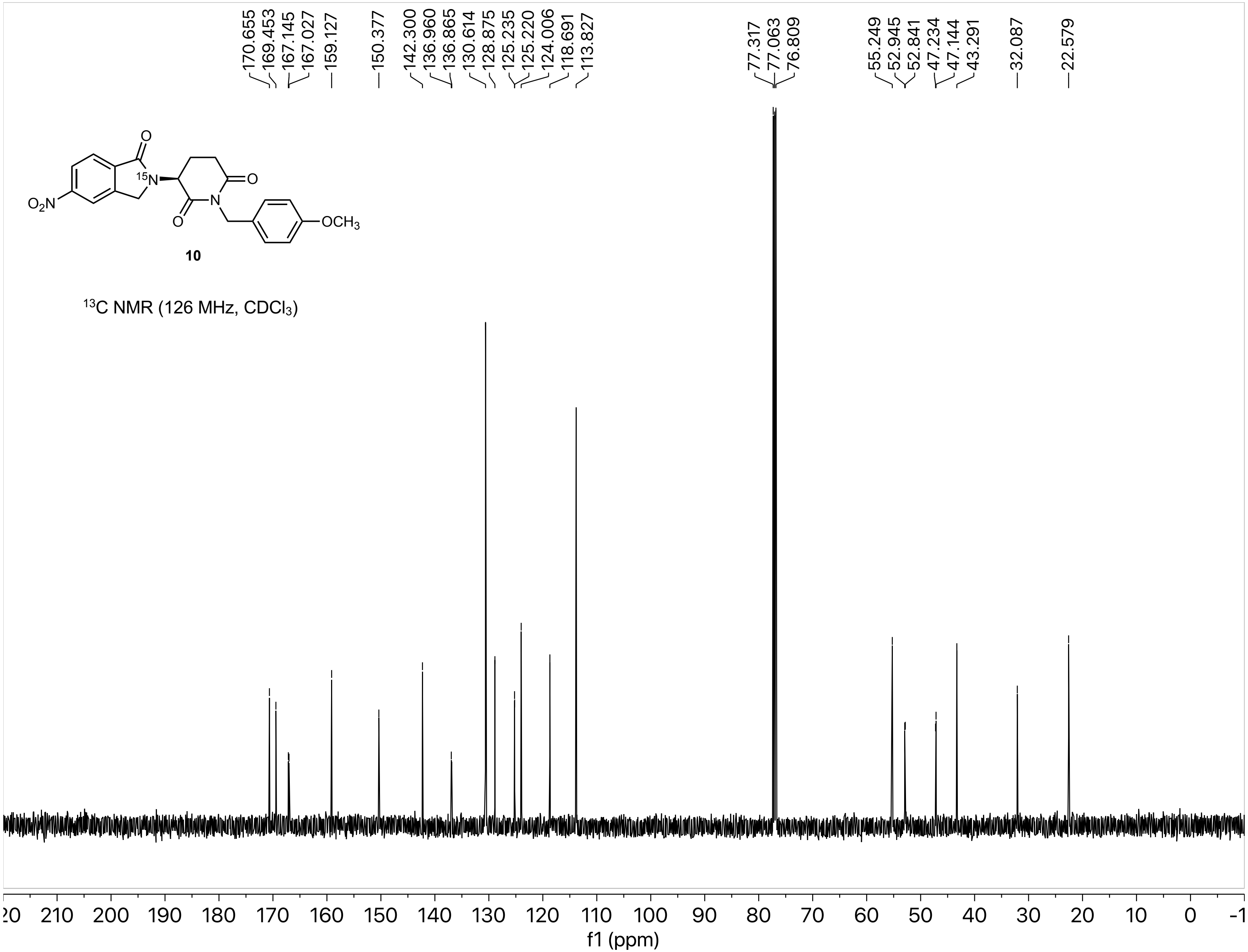
170.655
169.453
167.145
167.027
159.127
150.377
142.300
136.960
136.865
130.614
128.875
125.235
125.220
124.006
118.691
113.827
77.317
77.063
76.809
55.249
52.945
52.841
47.234
47.144
43.291
32.087
22.579
O
15N
O
O2N
O
N
OCH3
10
13C NMR (126 MHz, CDCl3)
f1 (ppm)

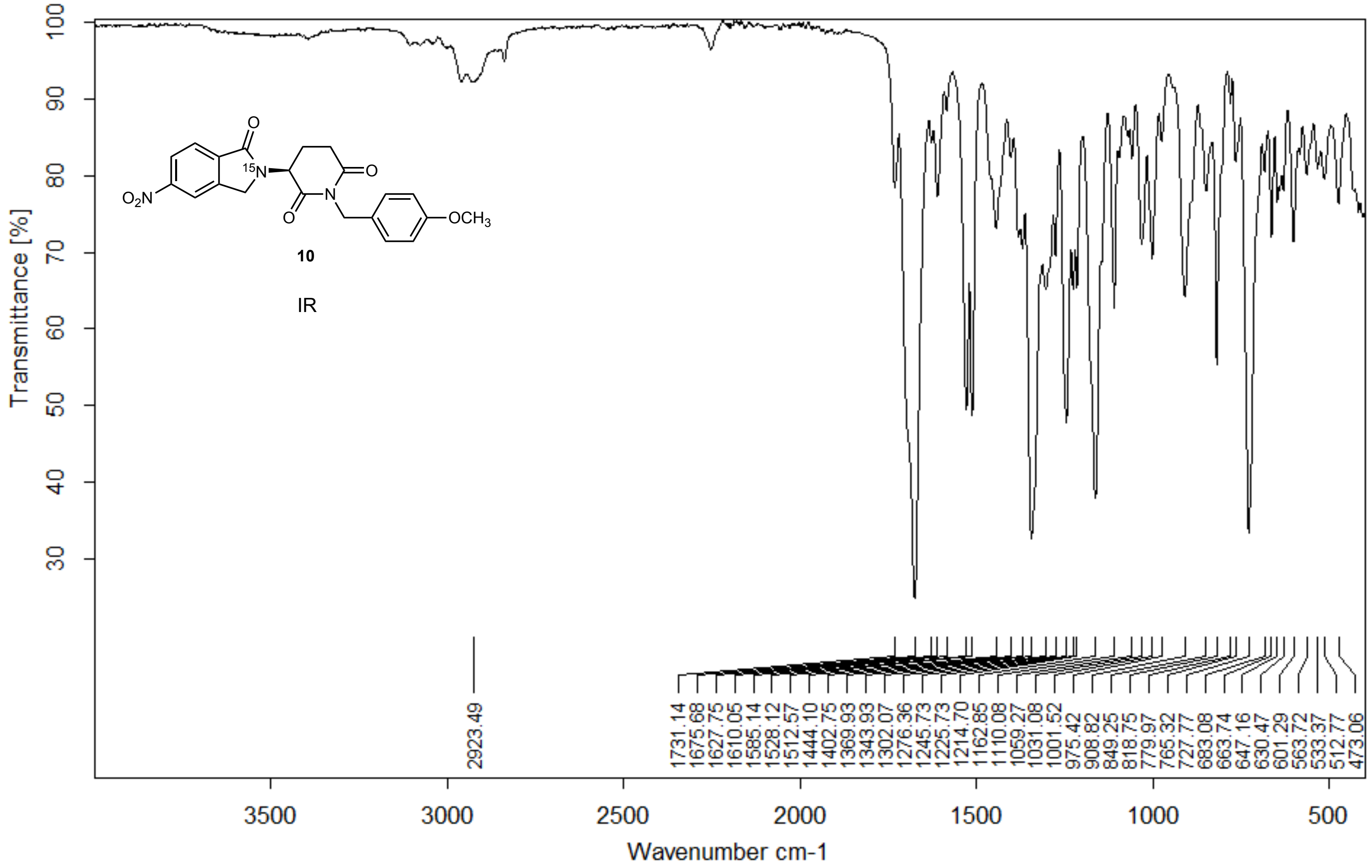

Z:\ALPHA FTIR DATA\Woo\Yuka\YA367.0	YA367	Diamond ATR	05/07/2018

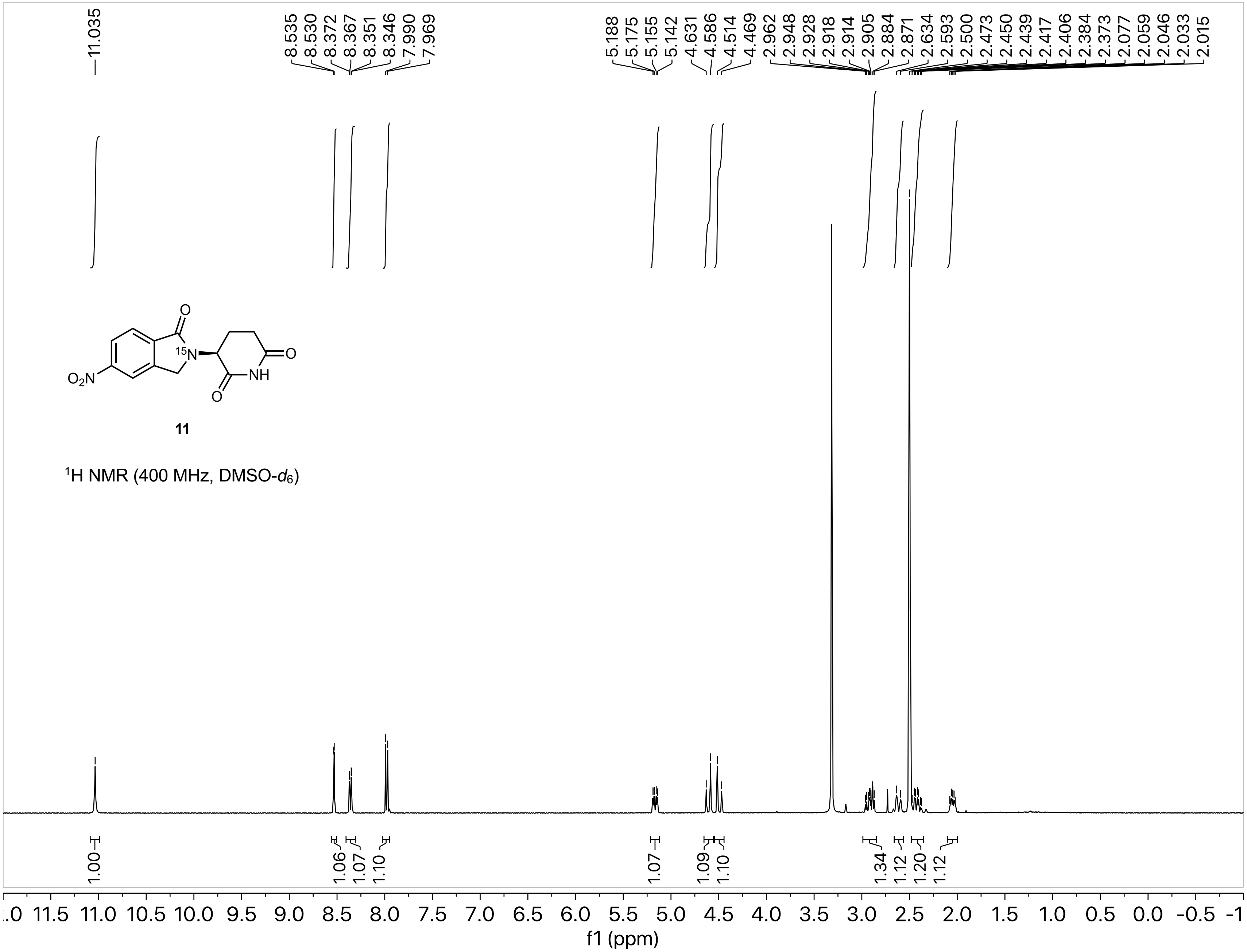
11.035
8.535 8.530 8.372 8.367 8.351 8.346 7.990 7.969
5.188 5.175 5.155 5.142 4.631 4.586 4.514 4.469 2.962 2.948 2.928 2.918 2.914 2.905 2.884 2.871 2.634 2.593 2.500 2.473 2.450 2.439 2.417 2.406 2.384 2.373 2.077 2.059 2.046 2.033 2.015
O
15N
O
O2N
NH
O
11
1H NMR (400 MHz, DMSO-d6)
1.00
1.06
1.07
1.10
1.07
1.09
1.10
1.34
1.12
1.20
1.12
.0 11.5 11.0 10.5 10.0 9.5 9.0 8.5 8.0 7.5 7.0 6.5 6.0 5.5 5.0 4.5 4.0 3.5 3.0 2.5 2.0 1.5 1.0 0.5 0.0 -0.5 -1
f1 (ppm)

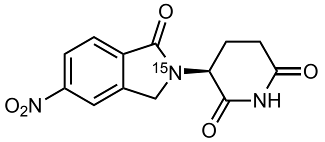

11

^{13}C NMR (101 MHz, DMSO-d_6)

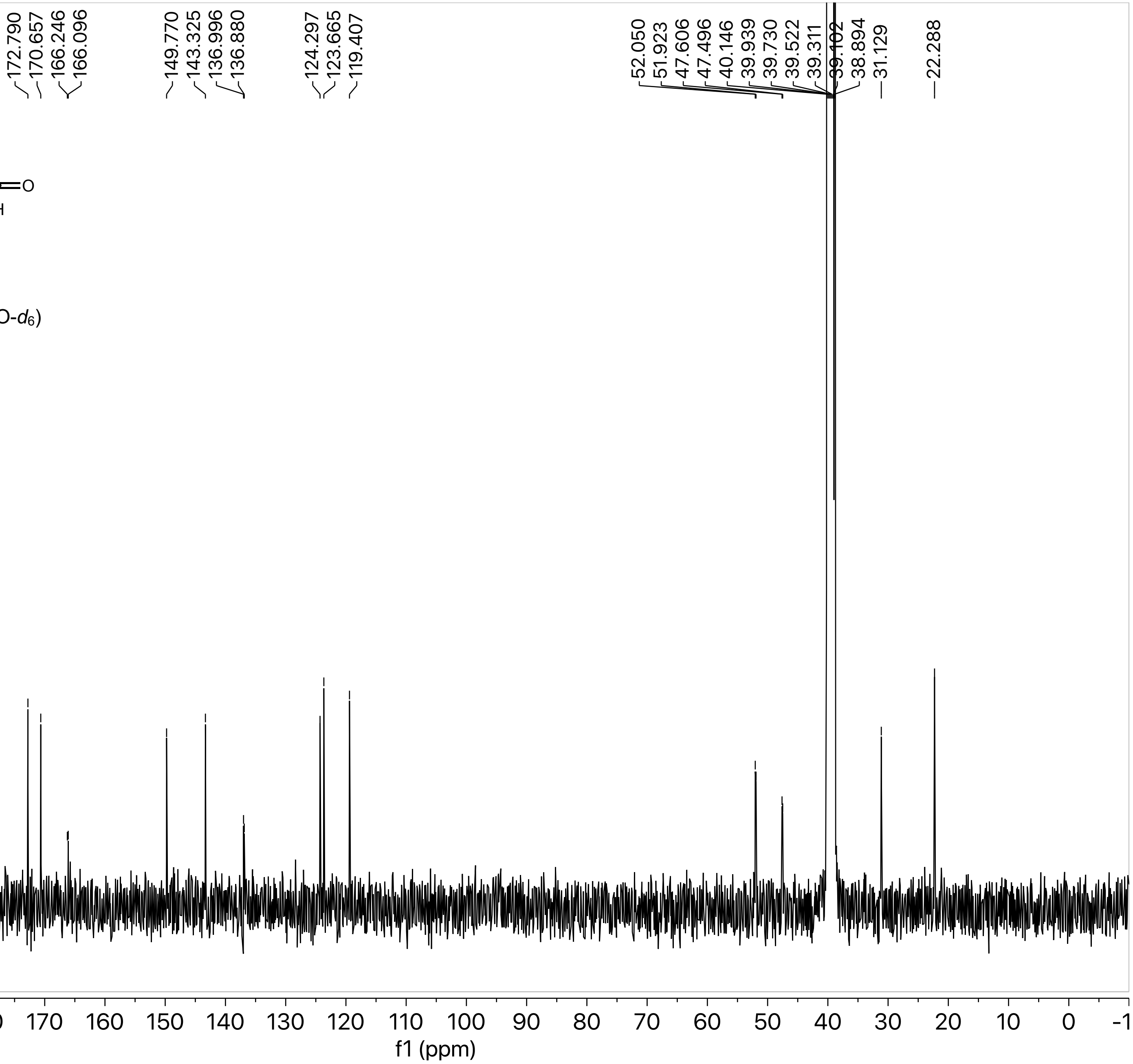

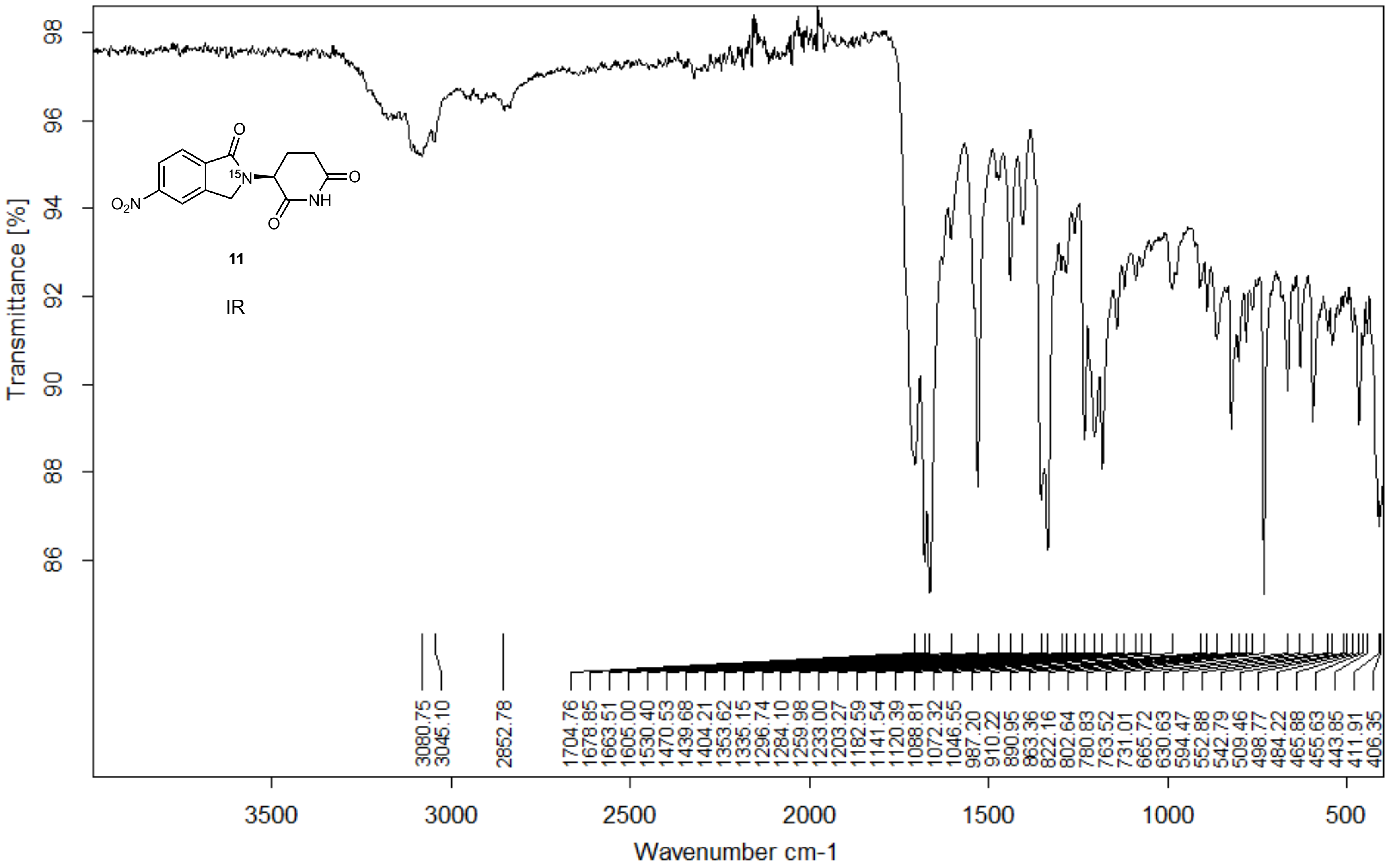

Z:\Alpha FTIR Data\Woo\Yuka\.0	Diamond ATR	05/12/2018

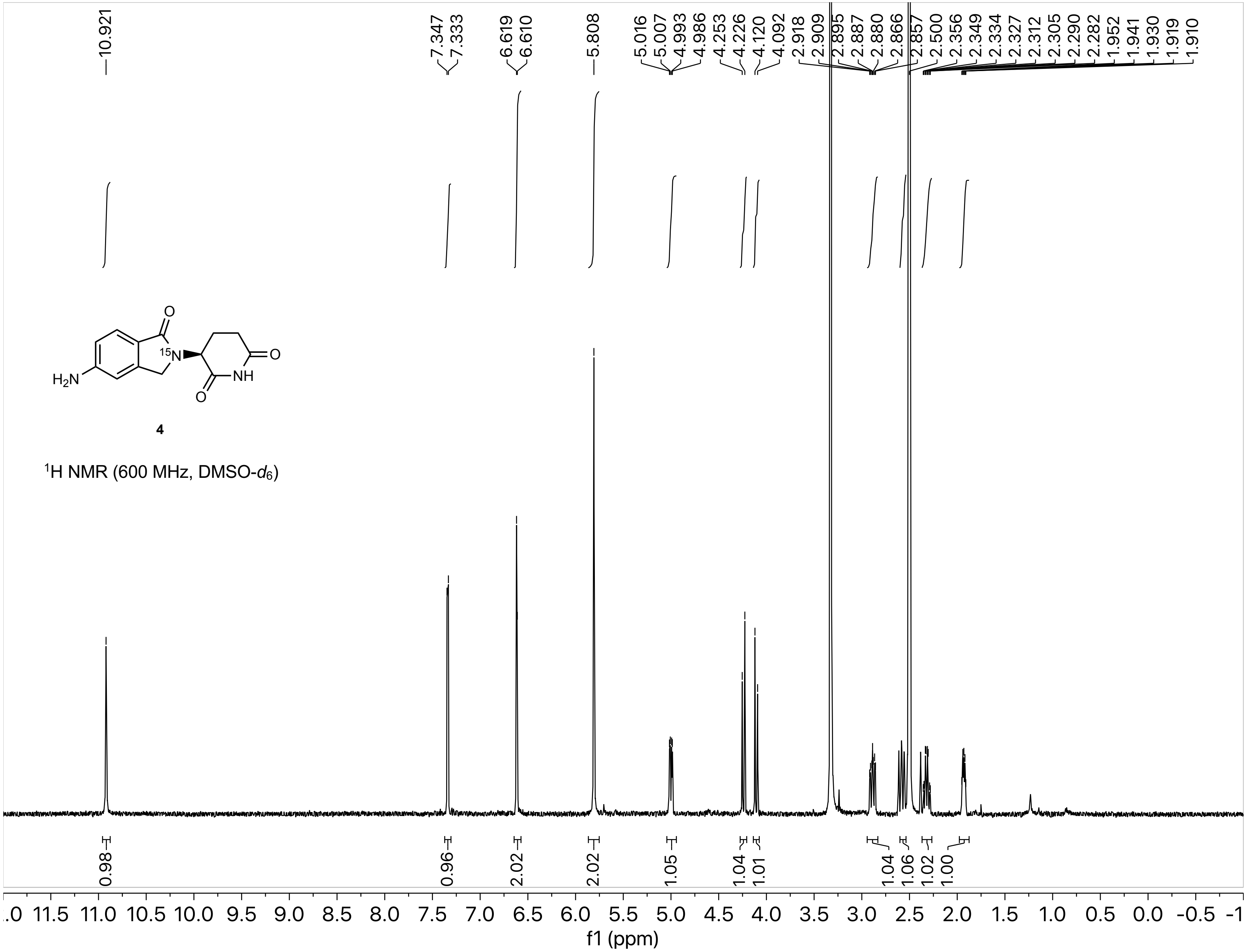
10.921
7.347
7.333
6.619
6.610
5.808
5.016
5.007
4.993
4.986
4.253
4.226
4.120
4.092
2.918
2.909
2.895
2.887
2.880
2.866
2.857
2.500
2.356
2.349
2.334
2.327
2.312
2.305
2.290
2.282
1.952
1.941
1.930
1.919
1.910
O
^{15}N
O
NH
H_2N
O
4
^{1}H NMR (600 MHz, DMSO-d_6)
0.98
0.96
2.02
2.02
1.05
1.04
1.01
1.04
1.06
1.02
1.00
11.5 11.0 10.5 10.0 9.5 9.0 8.5 8.0 7.5 7.0 6.5 6.0 5.5 5.0 4.5 4.0 3.5 3.0 2.5 2.0 1.5 1.0 0.5 0.0 -0.5 -1
f1 (ppm)

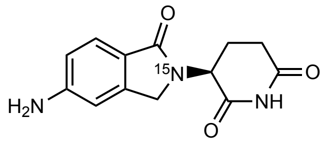

4

^{13}C NMR (101 MHz, DMSO-d_6)

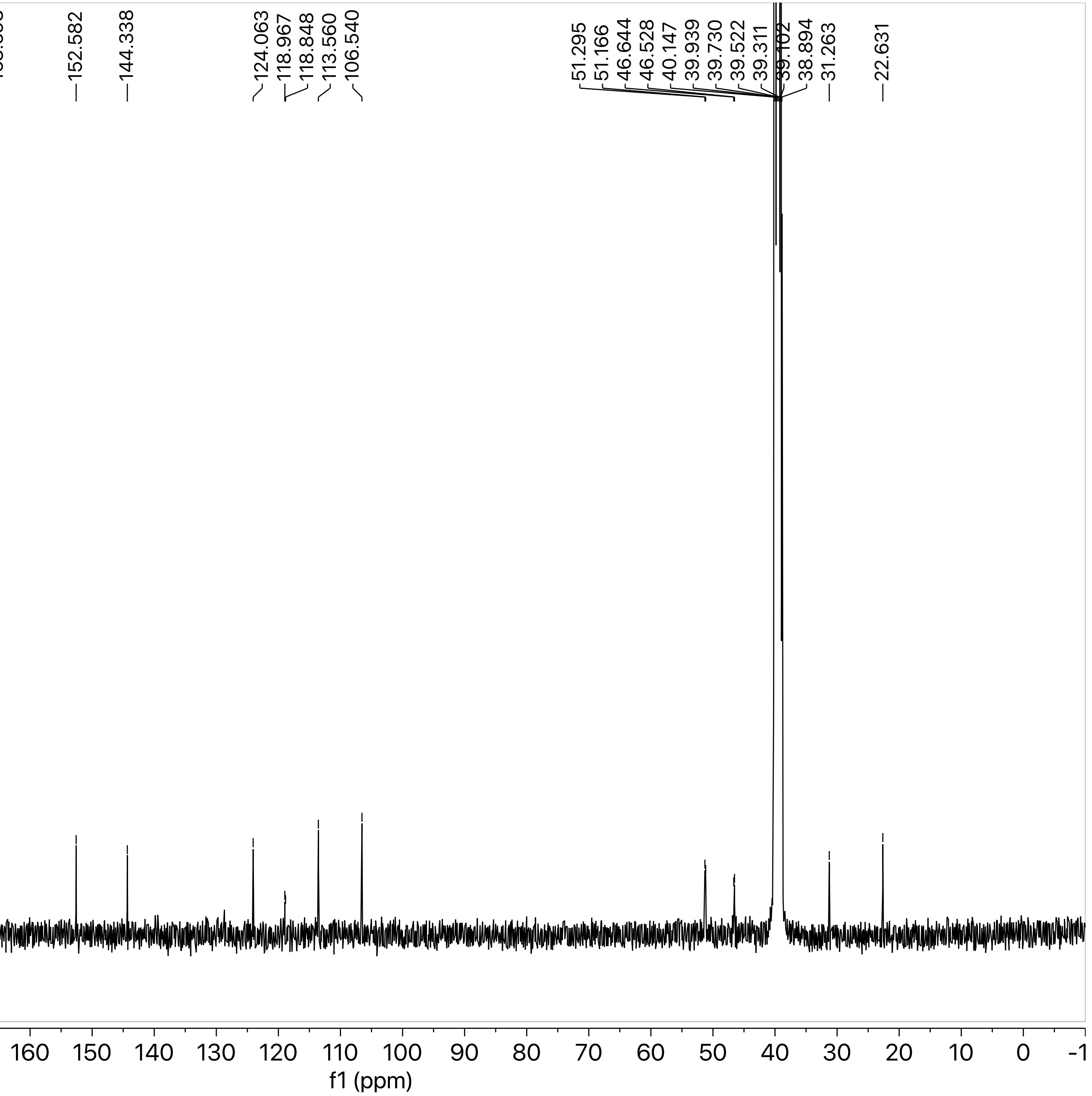

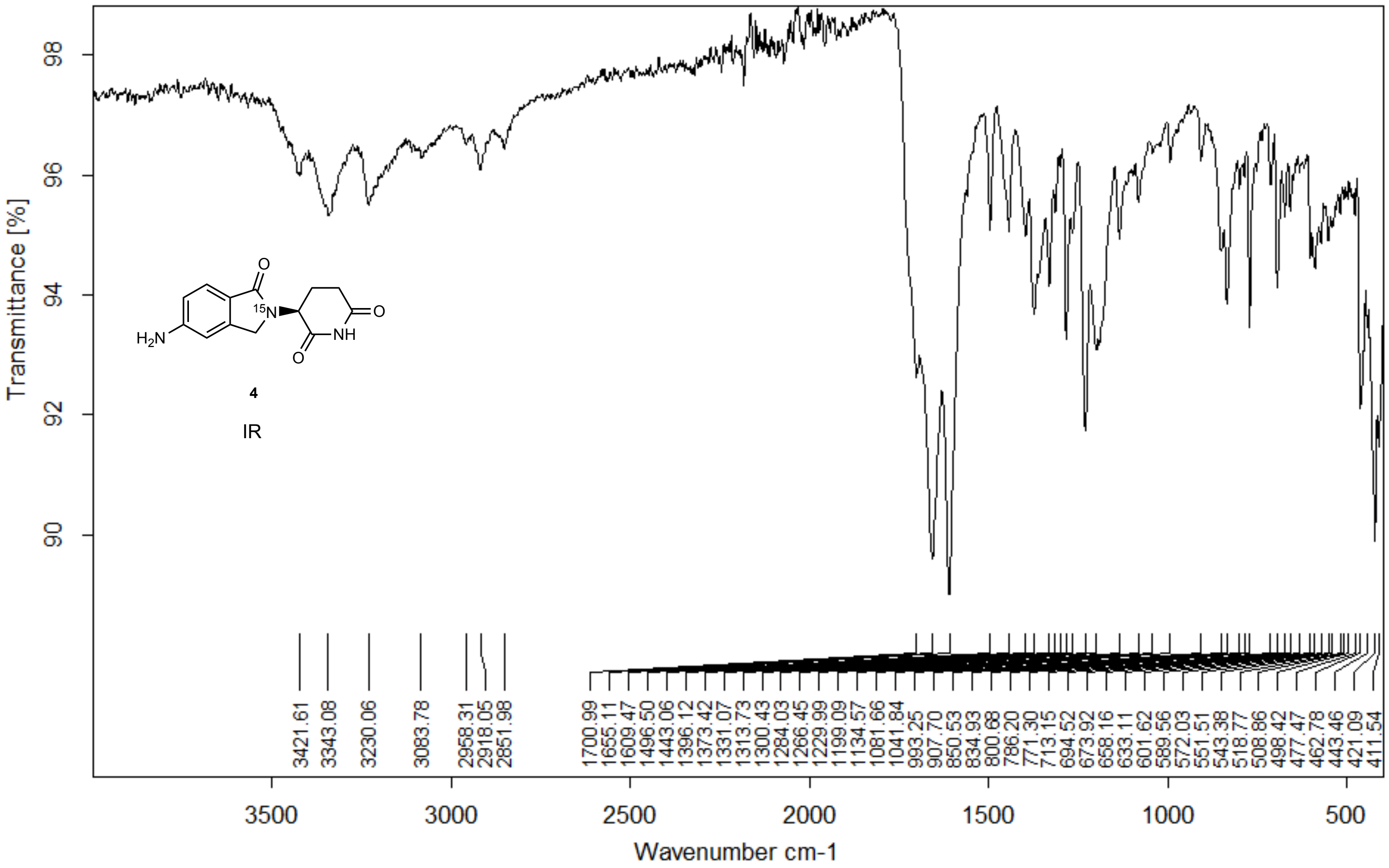

Z:\Alpha FTIR Data\Woo\Yuka\.3	Diamond ATR	05/12/2018

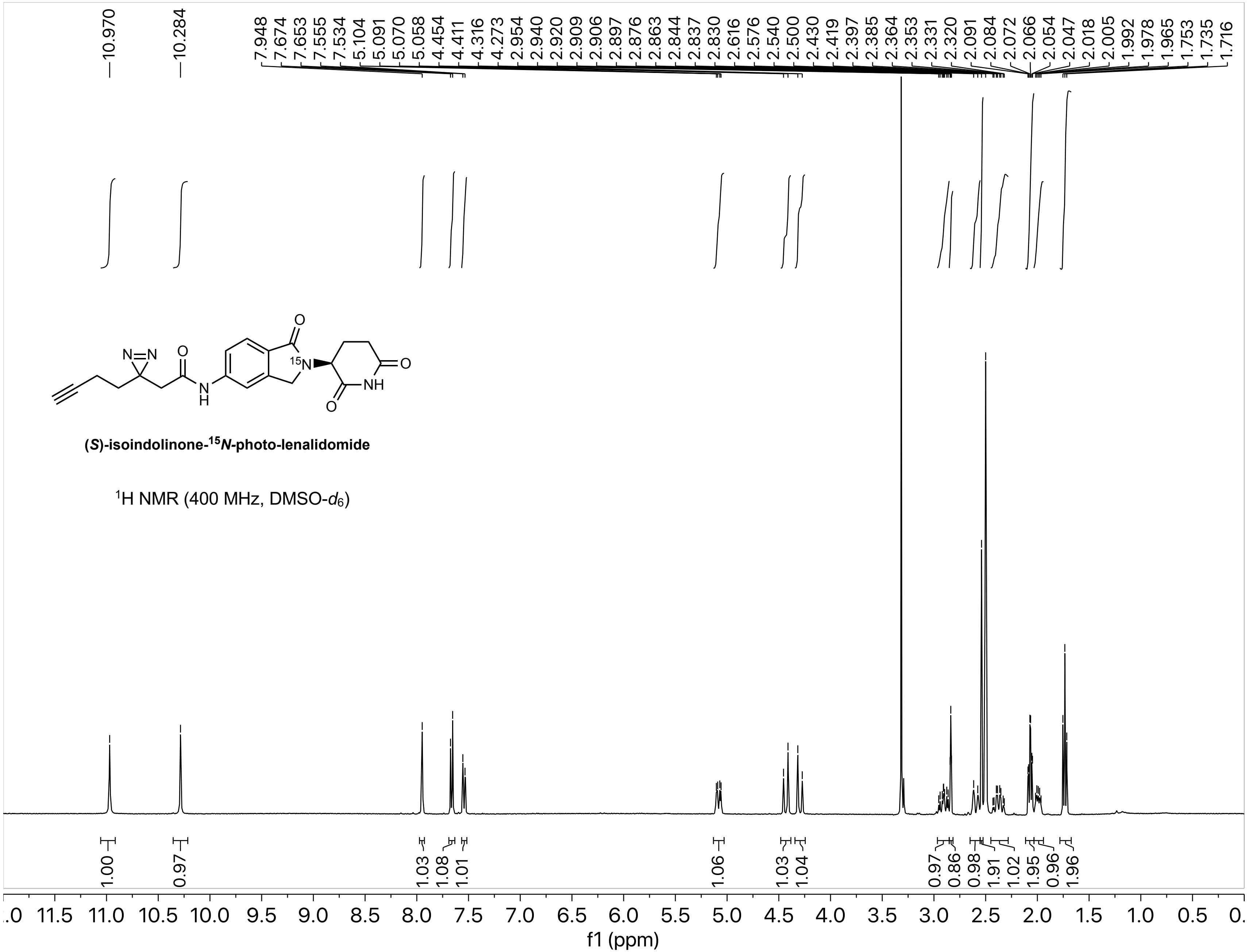

(*S*)-isoindolinone-^{15}*N*-photo-lenalidomide

^{1}H NMR (400 MHz, DMSO-d_6)

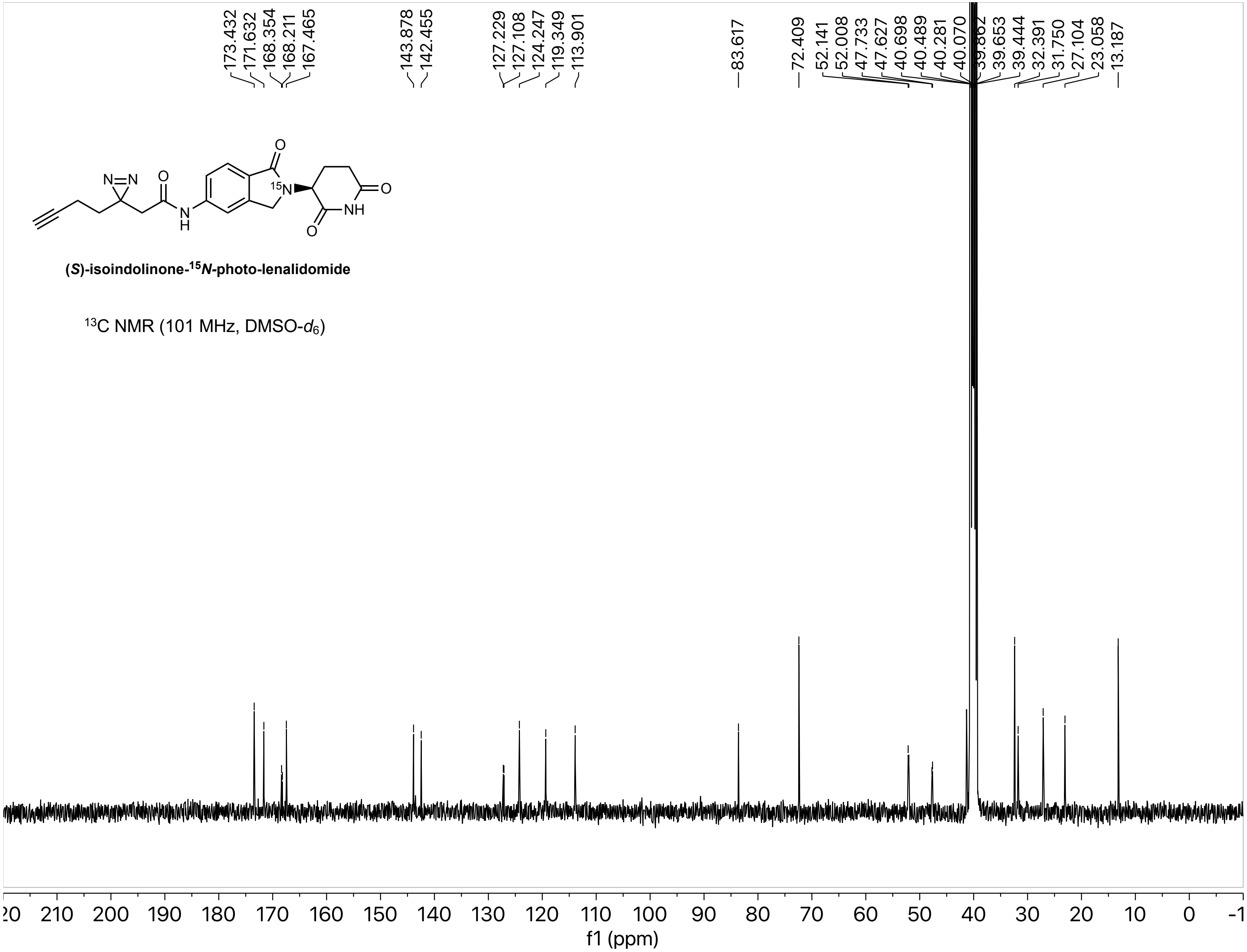

173.432
171.632
168.354
168.211
167.465
143.878
142.455
127.229
127.108
124.247
119.349
113.901
83.617
72.409
52.141
52.008
47.733
47.627
40.698
40.489
40.281
40.070
39.862
39.653
39.444
32.391
31.750
27.104
23.058
13.187
N=N
O
O
15N
O
N
H
NH
O
(S)-isoindolinone-15N-photo-lenalidomide
13C NMR (101 MHz, DMSO-d6)
20 210 200 190 180 170 160 150 140 130 120 110 100 90 80 70 60 50 40 30 20 10 0 -1
f1 (ppm)

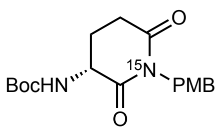

12

^{1}H NMR (500 MHz, $CDCl_3$)

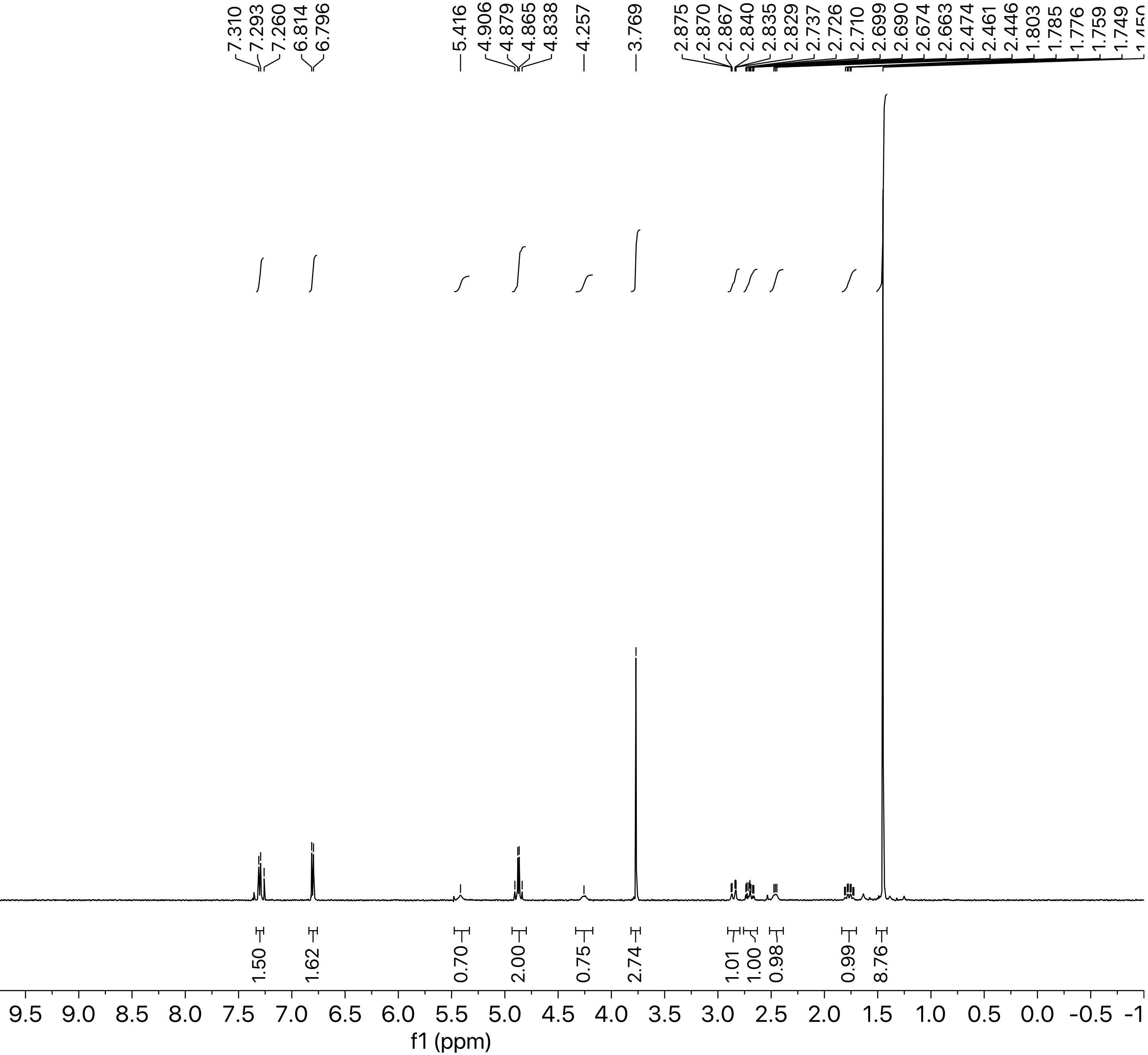

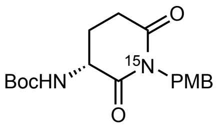

12

^{13}C NMR (126 MHz, $CDCl_3$)

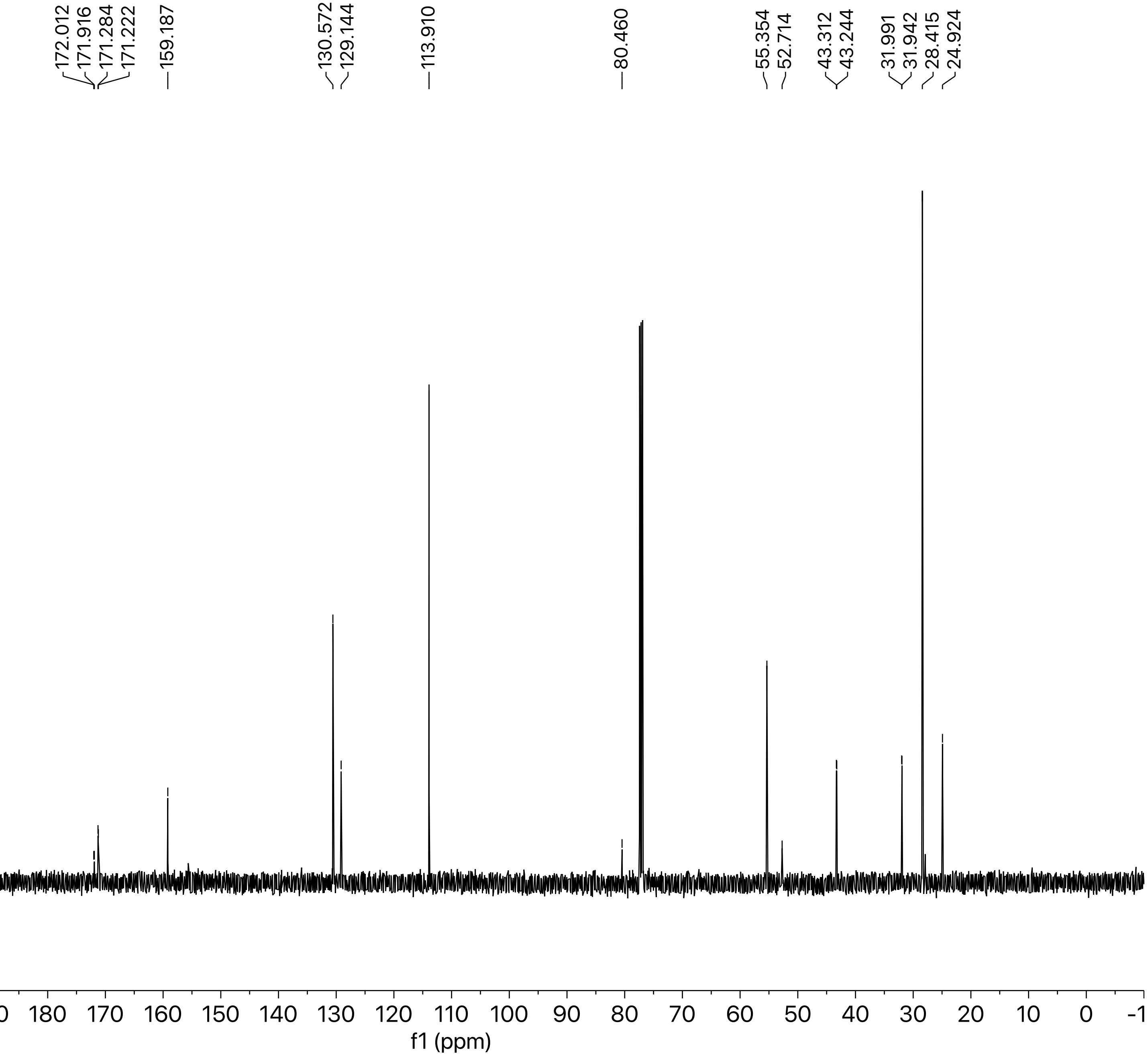

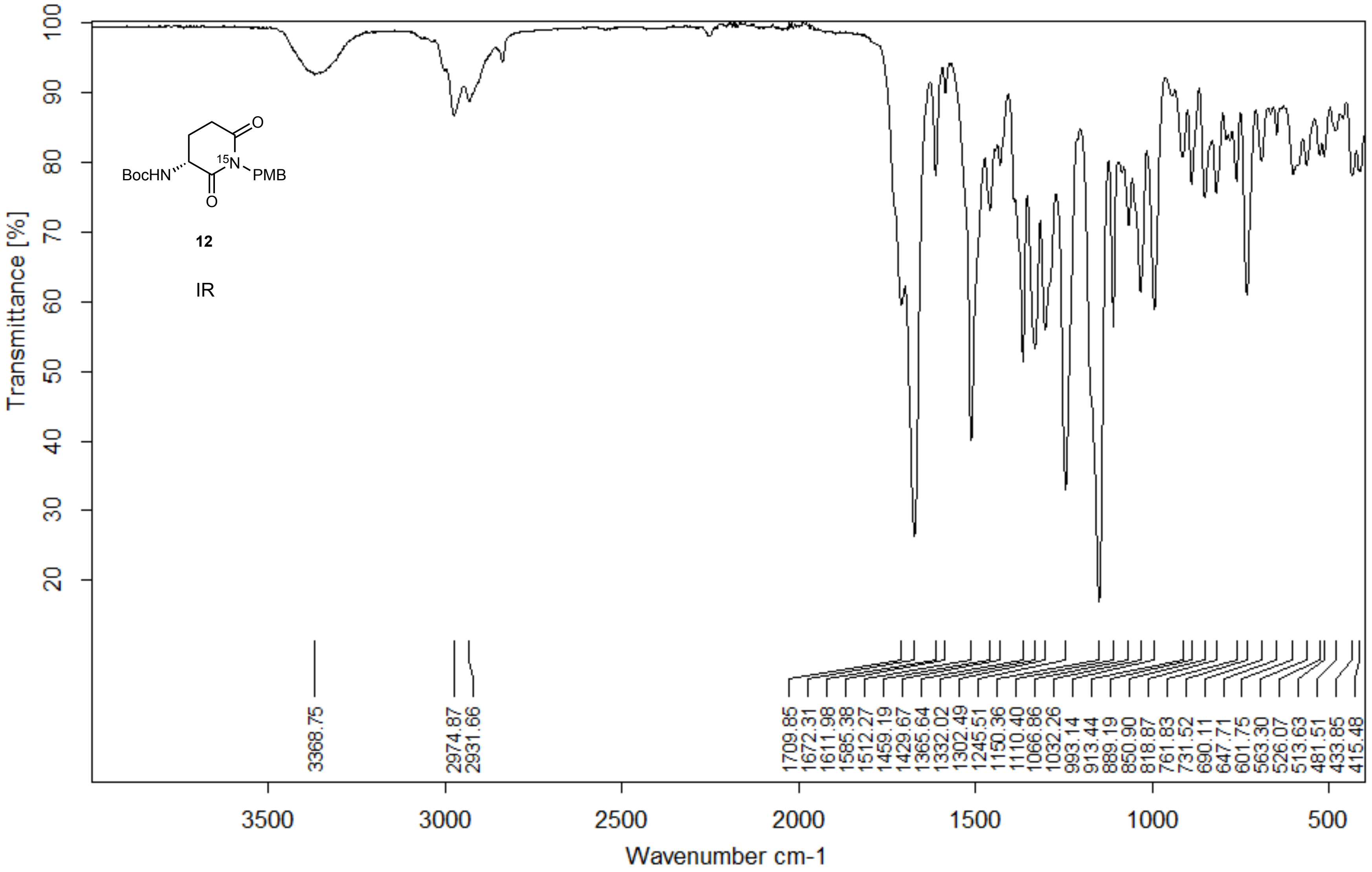

Z:\Alpha FTIR Data\Woo\Yuka\YA138.0 YA138 Diamond ATR | 08/12/2018

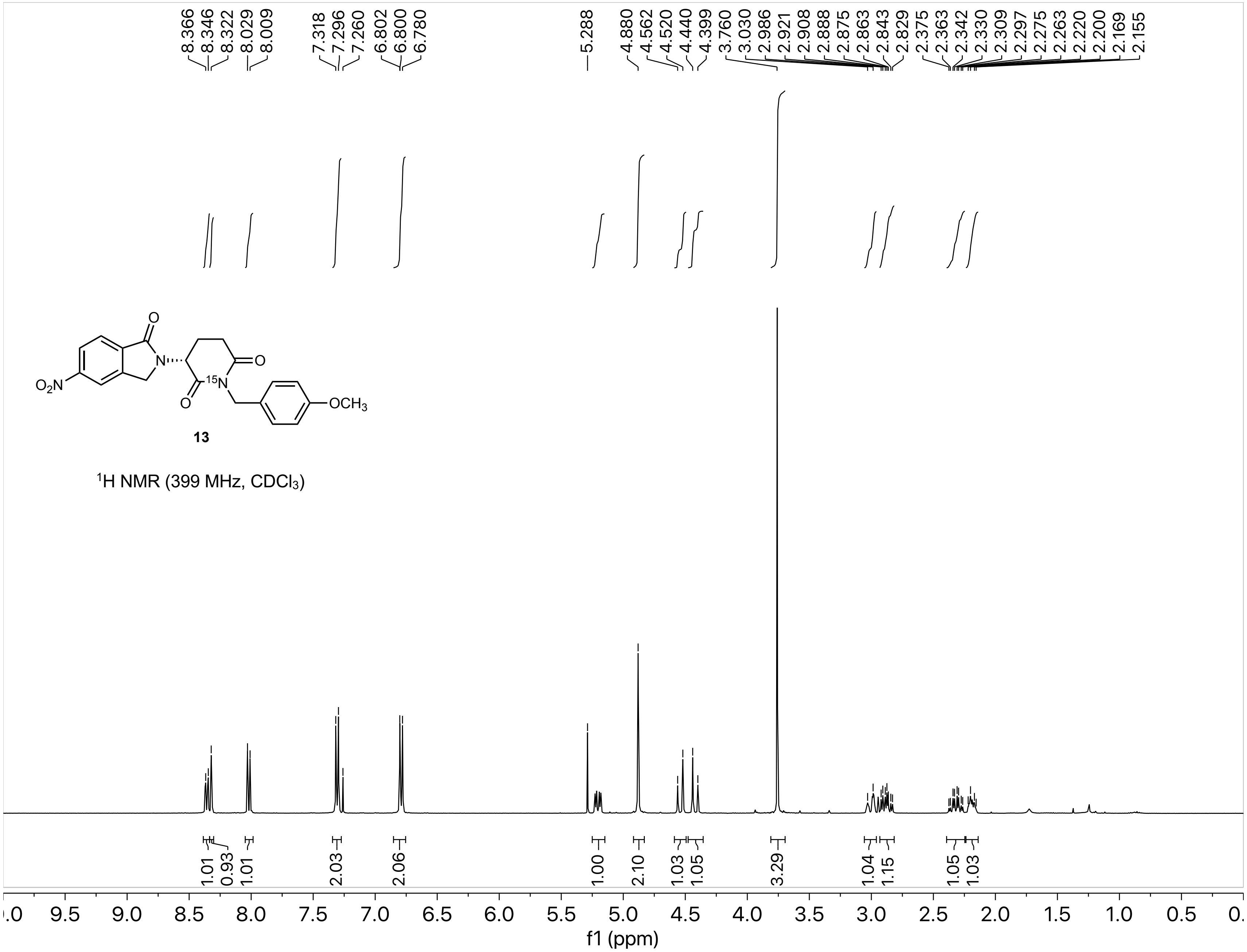
13
^{1}H NMR (399 MHz, CDCl$_3$)
f1 (ppm)

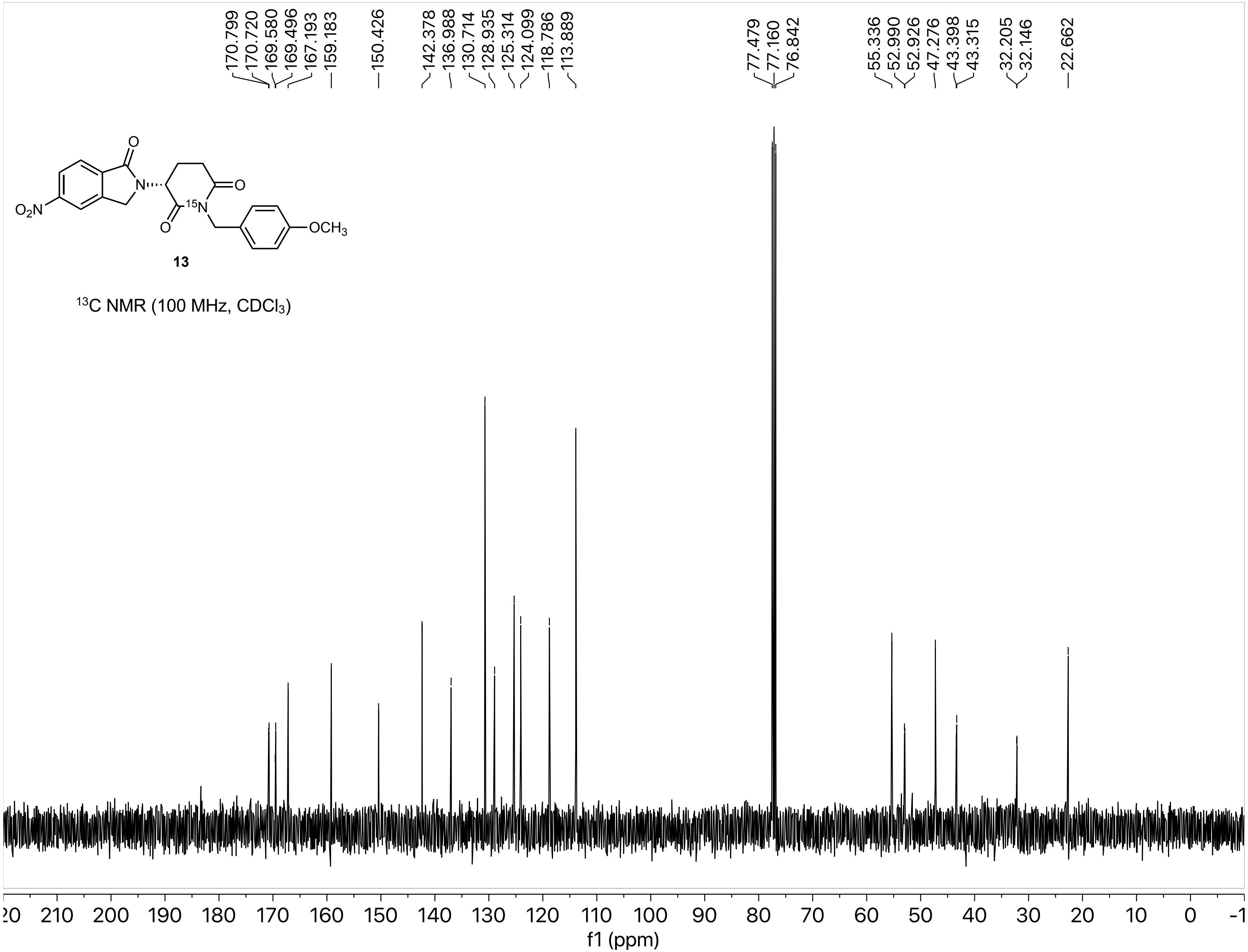

170.799
170.720
169.580
169.496
167.193
159.183
150.426
142.378
136.988
130.714
128.935
125.314
124.099
118.786
113.889
77.479
77.160
76.842
55.336
52.990
52.926
47.276
43.398
43.315
32.205
32.146
22.662
O
N
15N
O
O
O2N
OCH3
13
^{13}C NMR (100 MHz, $CDCl_3$)
20 210 200 190 180 170 160 150 140 130 120 110 100 90 80 70 60 50 40 30 20 10 0 -1
f1 (ppm)

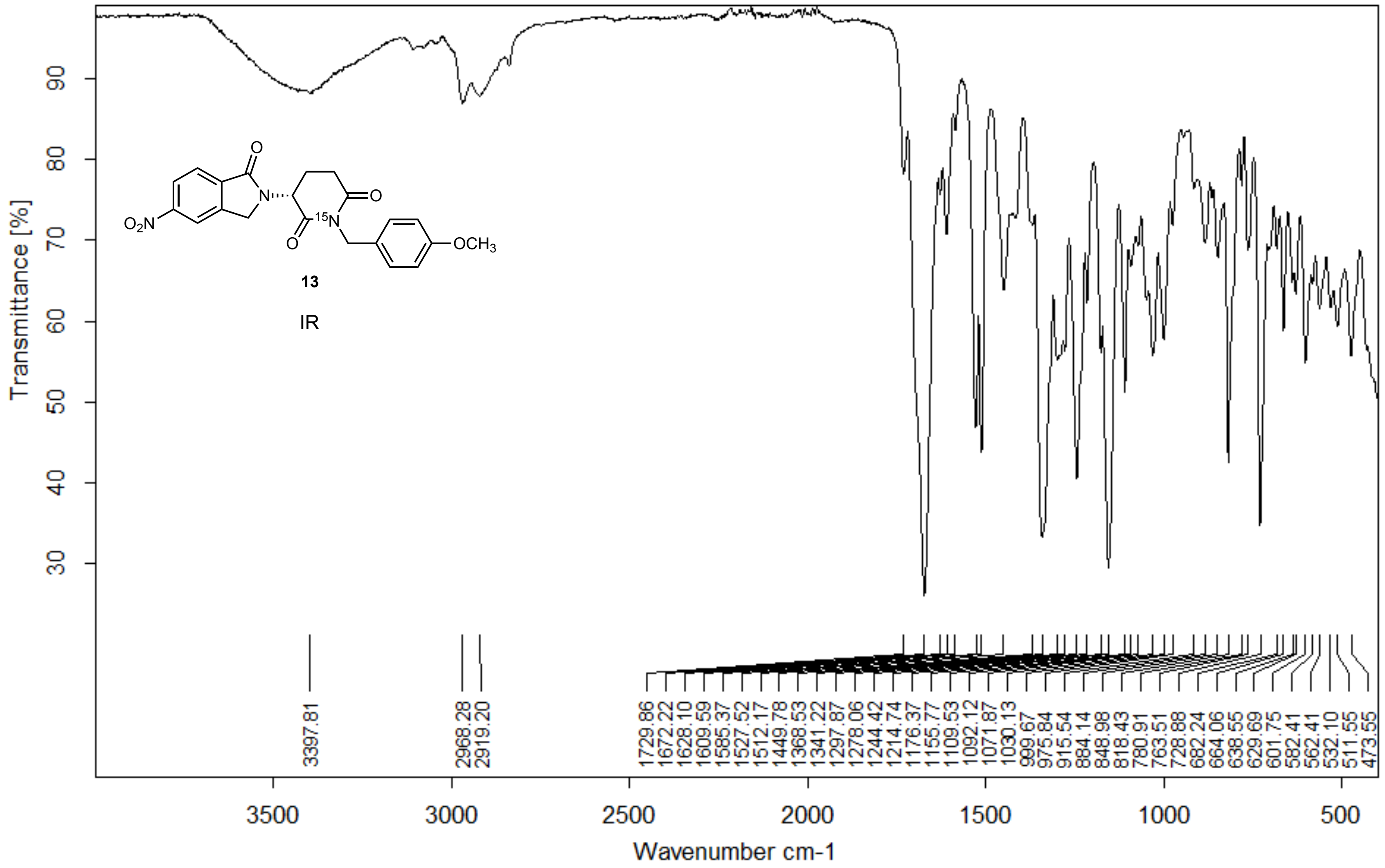

Z:\ALPHA FTIR DATA\Woo\Yuka\YA366.0	YA366	Diamond ATR	03/07/2018

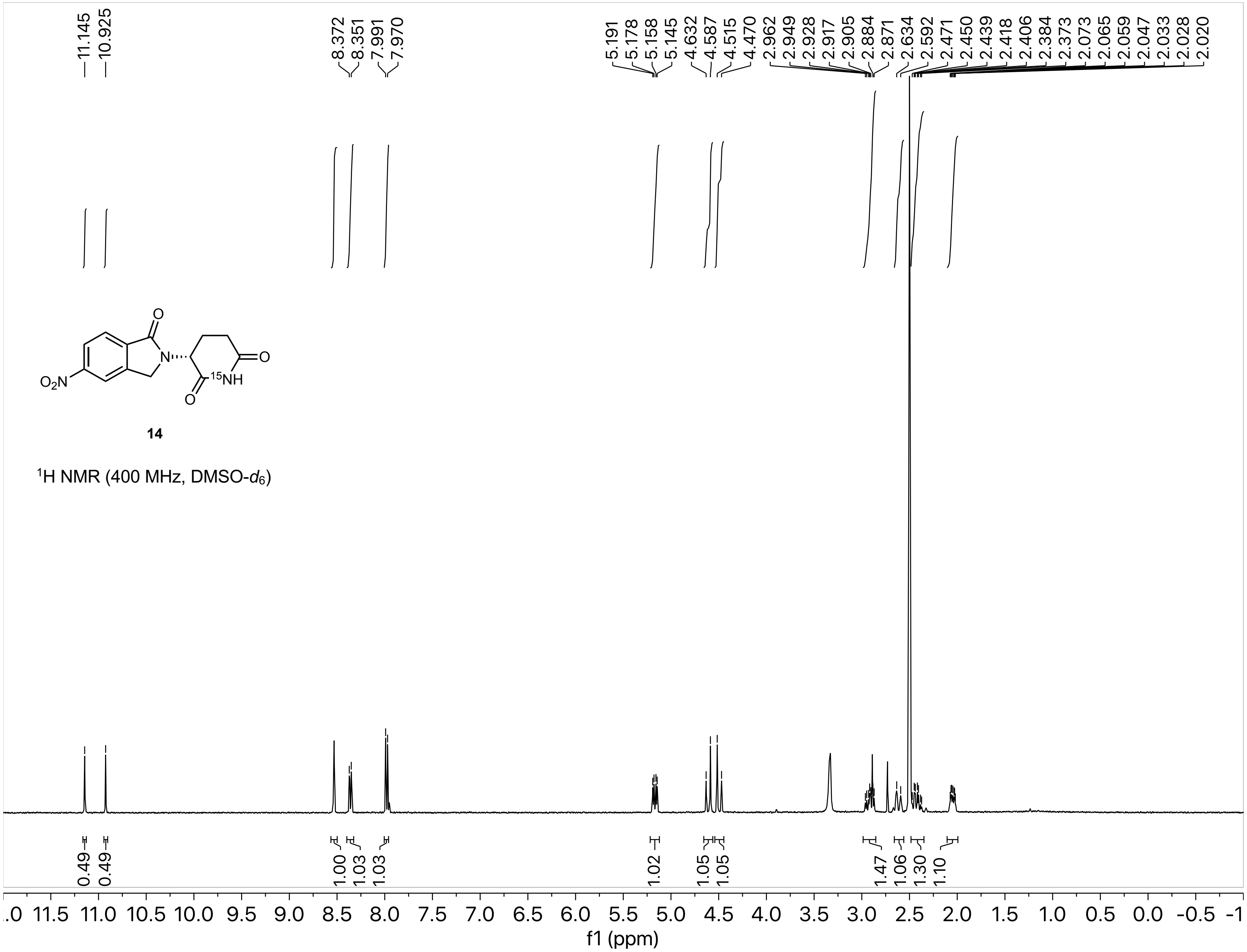
11.145
10.925
8.372
8.351
7.991
7.970
5.191
5.178
5.158
5.145
4.632
4.587
4.515
4.470
2.962
2.949
2.928
2.917
2.905
2.884
2.871
2.634
2.592
2.471
2.450
2.439
2.418
2.406
2.384
2.373
2.073
2.065
2.059
2.047
2.033
2.028
2.020
O
N
O
^{15}NH
O
O_2N
O
14
^{1}H NMR (400 MHz, DMSO-d_6)
0.49
0.49
1.00
1.03
1.03
1.02
1.05
1.05
1.47
1.06
1.30
1.10
.0 11.5 11.0 10.5 10.0 9.5 9.0 8.5 8.0 7.5 7.0 6.5 6.0 5.5 5.0 4.5 4.0 3.5 3.0 2.5 2.0 1.5 1.0 0.5 0.0 -0.5 -1
f1 (ppm)

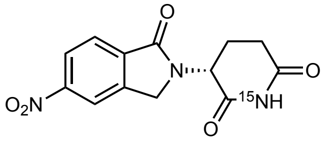

14

^{13}C NMR (101 MHz, DMSO-d_6)

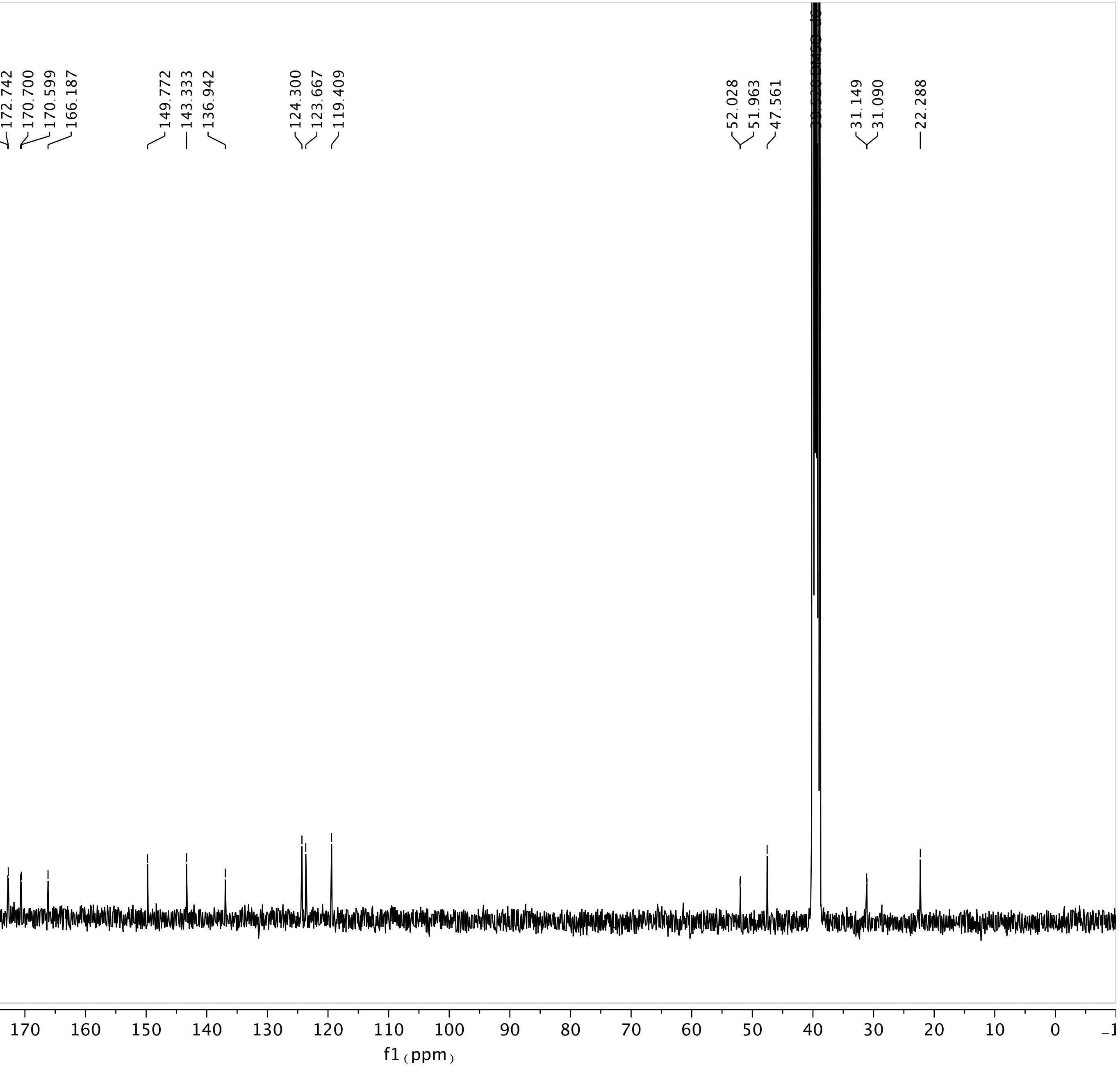

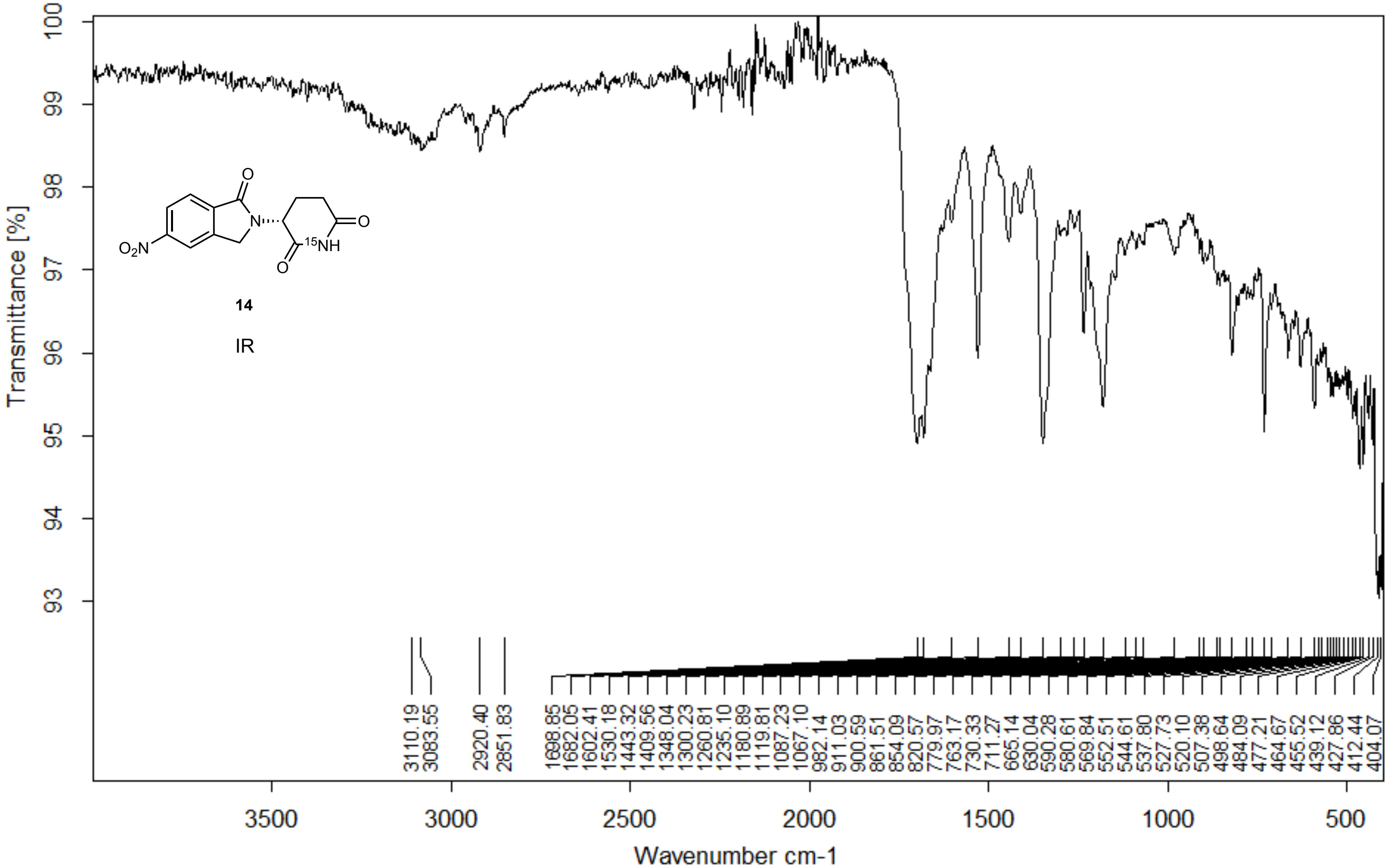

Z:\Alpha FTIR Data\Woo\Yuka\azido teste.0	azido teste	Diamond ATR	05/12/2018

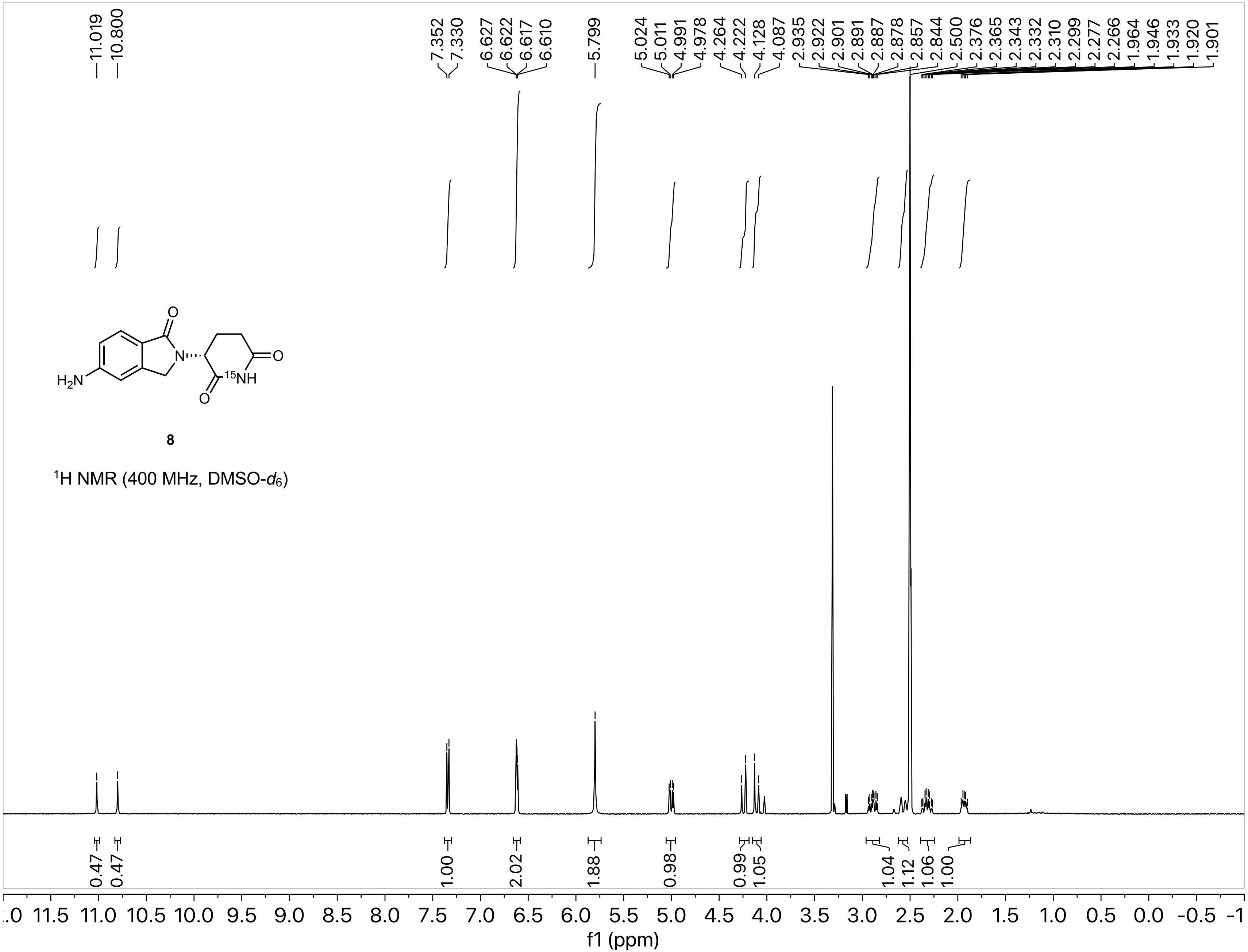

8
1H NMR (400 MHz, DMSO-d6)
11.019
10.800
7.352
7.330
6.627
6.622
6.617
6.610
5.799
5.024
5.011
4.991
4.978
4.264
4.222
4.128
4.087
2.935
2.922
2.901
2.891
2.887
2.878
2.857
2.844
2.500
2.376
2.365
2.343
2.332
2.310
2.299
2.277
2.266
1.964
1.946
1.933
1.920
1.901
0.47
0.47
1.00
2.02
1.88
0.98
0.99
1.05
1.04
1.12
1.06
1.00
f1 (ppm)

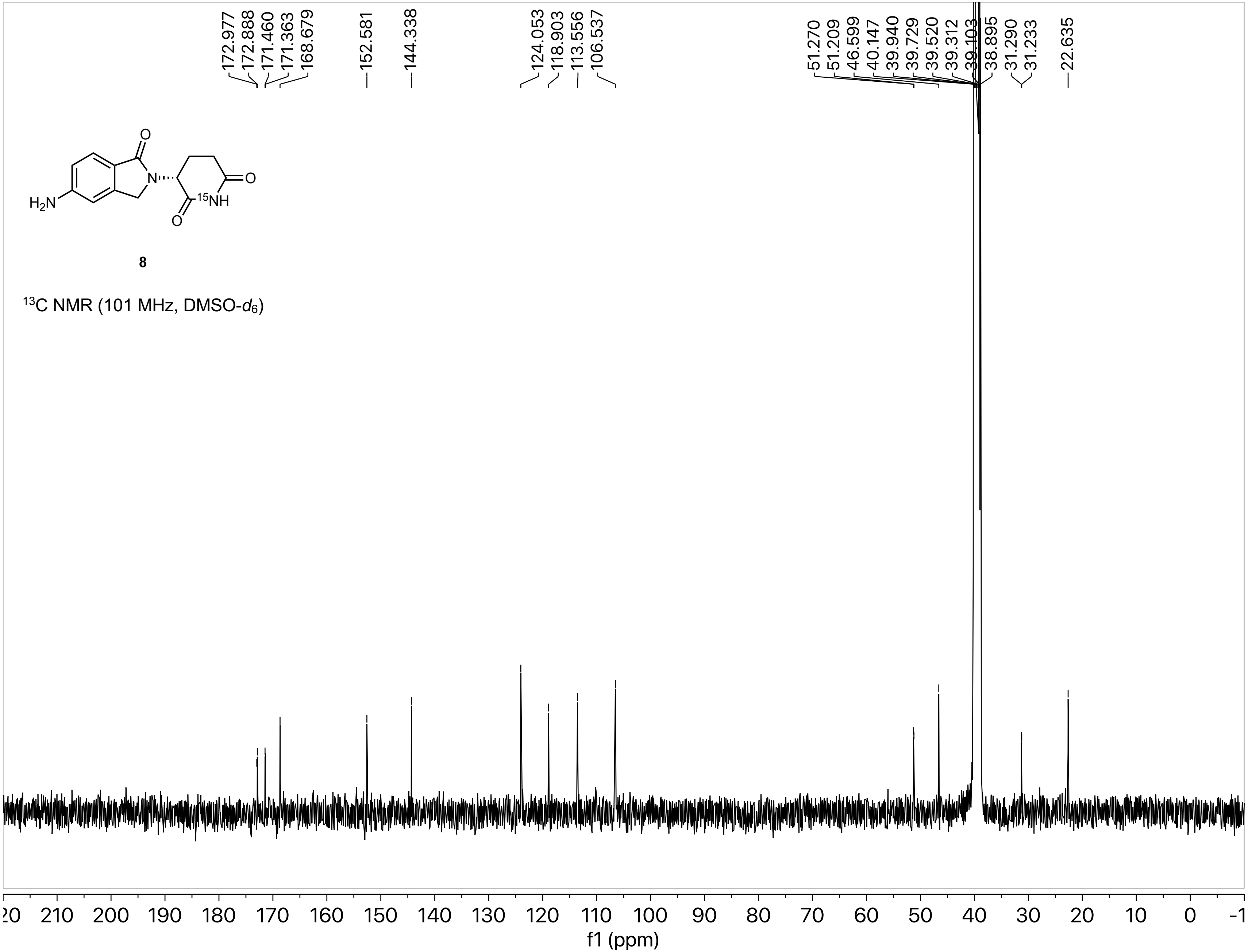

8

^{13}C NMR (101 MHz, DMSO-d_6)

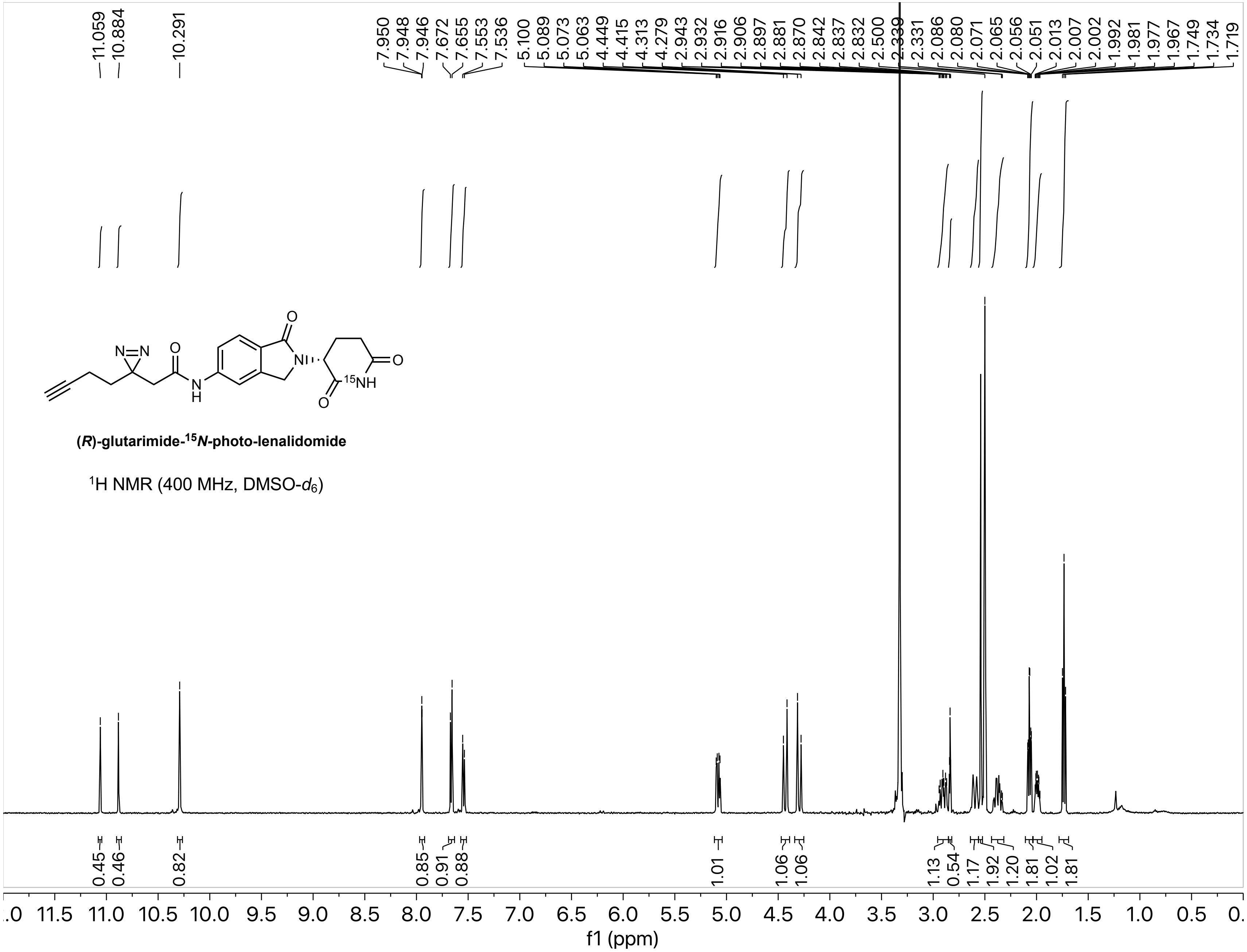

11.059
10.884
10.291
7.950
7.948
7.946
7.672
7.655
7.553
7.536
5.100
5.089
5.073
5.063
4.449
4.415
4.313
4.279
2.943
2.932
2.916
2.906
2.897
2.881
2.870
2.842
2.837
2.832
2.500
2.339
2.331
2.086
2.080
2.071
2.065
2.056
2.051
2.013
2.007
2.002
1.992
1.981
1.977
1.967
1.749
1.734
1.719
N=N
O
N
H
N
O
O
O
15NH
(*R*)-glutarimide-15*N*-photo-lenalidomide
1H NMR (400 MHz, DMSO-*d*6)
0.45
0.46
0.82
0.85
0.91
0.88
1.01
1.06
1.06
1.13
0.54
1.17
1.92
1.20
1.81
1.02
1.81
.0 11.5 11.0 10.5 10.0 9.5 9.0 8.5 8.0 7.5 7.0 6.5 6.0 5.5 5.0 4.5 4.0 3.5 3.0 2.5 2.0 1.5 1.0 0.5 0.
f1 (ppm)

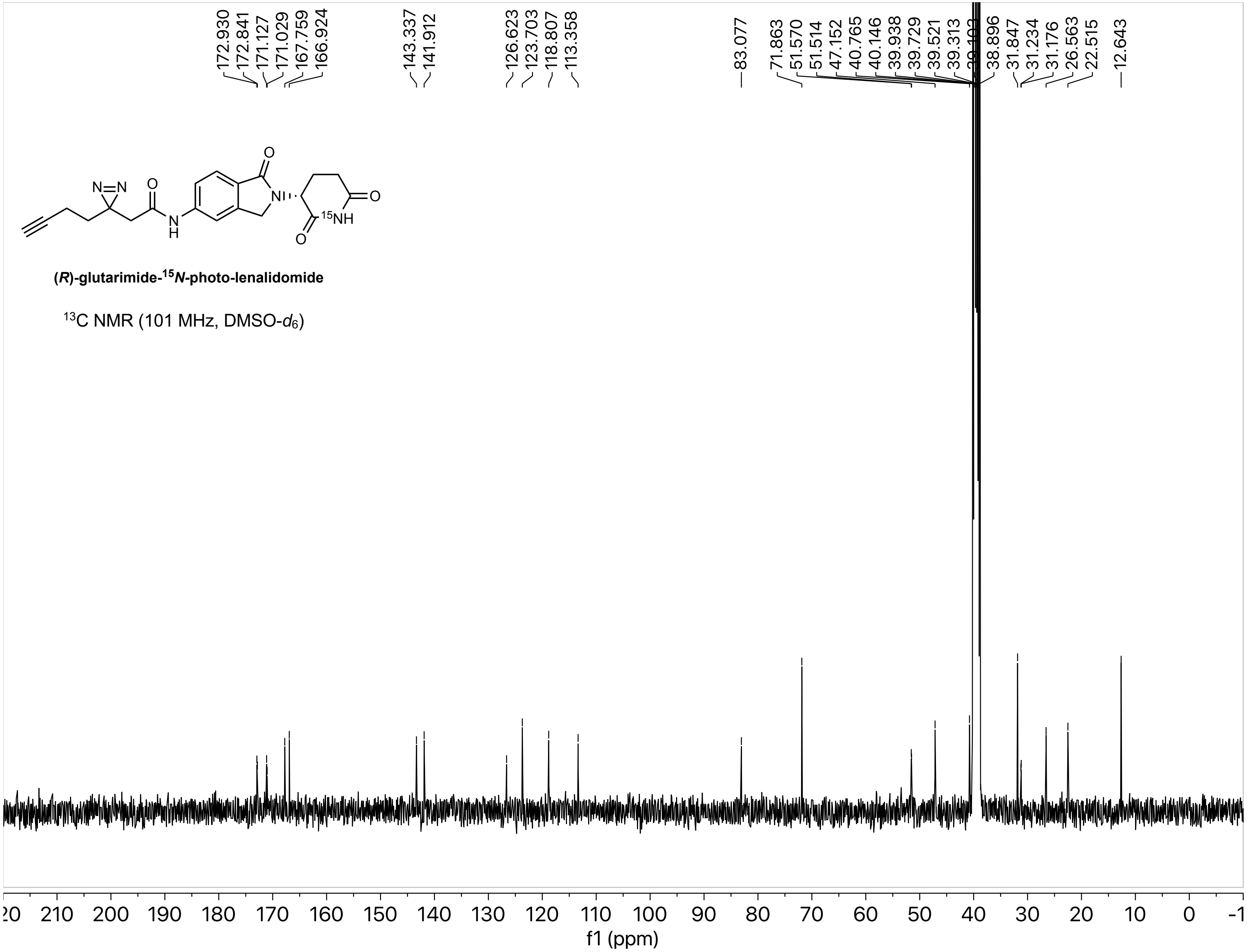
(R)-glutarimide-15N-photo-lenalidomide
13C NMR (101 MHz, DMSO-d6)
172.930
172.841
171.127
171.029
167.759
166.924
143.337
141.912
126.623
123.703
118.807
113.358
83.077
71.863
51.570
51.514
47.152
40.765
40.146
39.938
39.729
39.521
39.313
38.896
31.847
31.234
31.176
26.563
22.515
12.643
f1 (ppm)

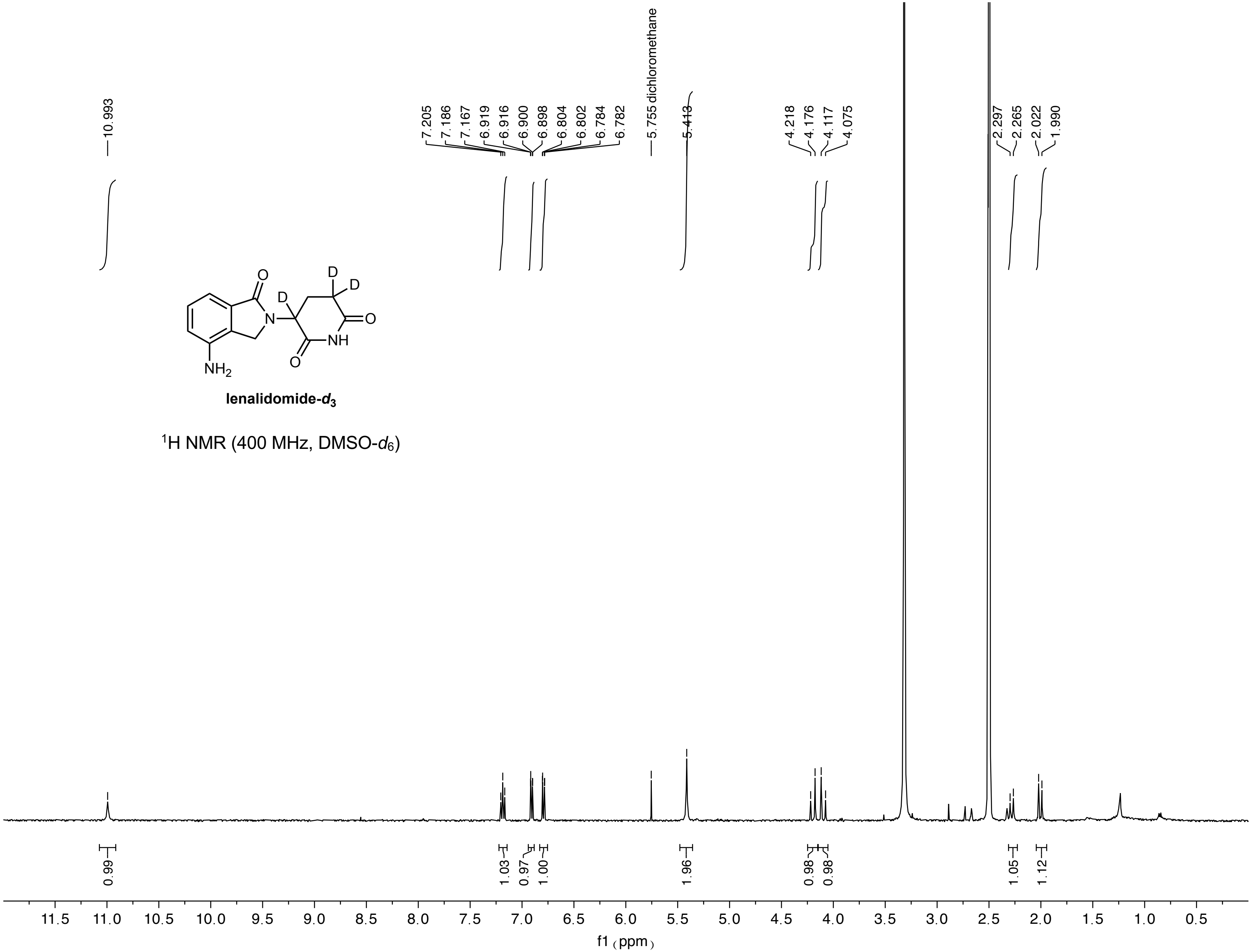
10.993
7.205
7.186
7.167
6.919
6.916
6.900
6.898
6.804
6.802
6.784
6.782
5.755 dichloromethane
5.413
4.218
4.176
4.117
4.075
2.297
2.265
2.022
1.990
D
D
D
O
O
O
N
NH
NH_2
lenalidomide-*d*$_3$
^{1}H NMR (400 MHz, DMSO-d_6)
0.99
1.03
0.97
1.00
1.96
0.98
0.98
1.05
1.12
11.5
11.0
10.5
10.0
9.5
9.0
8.5
8.0
7.5
7.0
6.5
6.0
5.5
5.0
4.5
4.0
3.5
3.0
2.5
2.0
1.5
1.0
0.5
f1 (ppm)

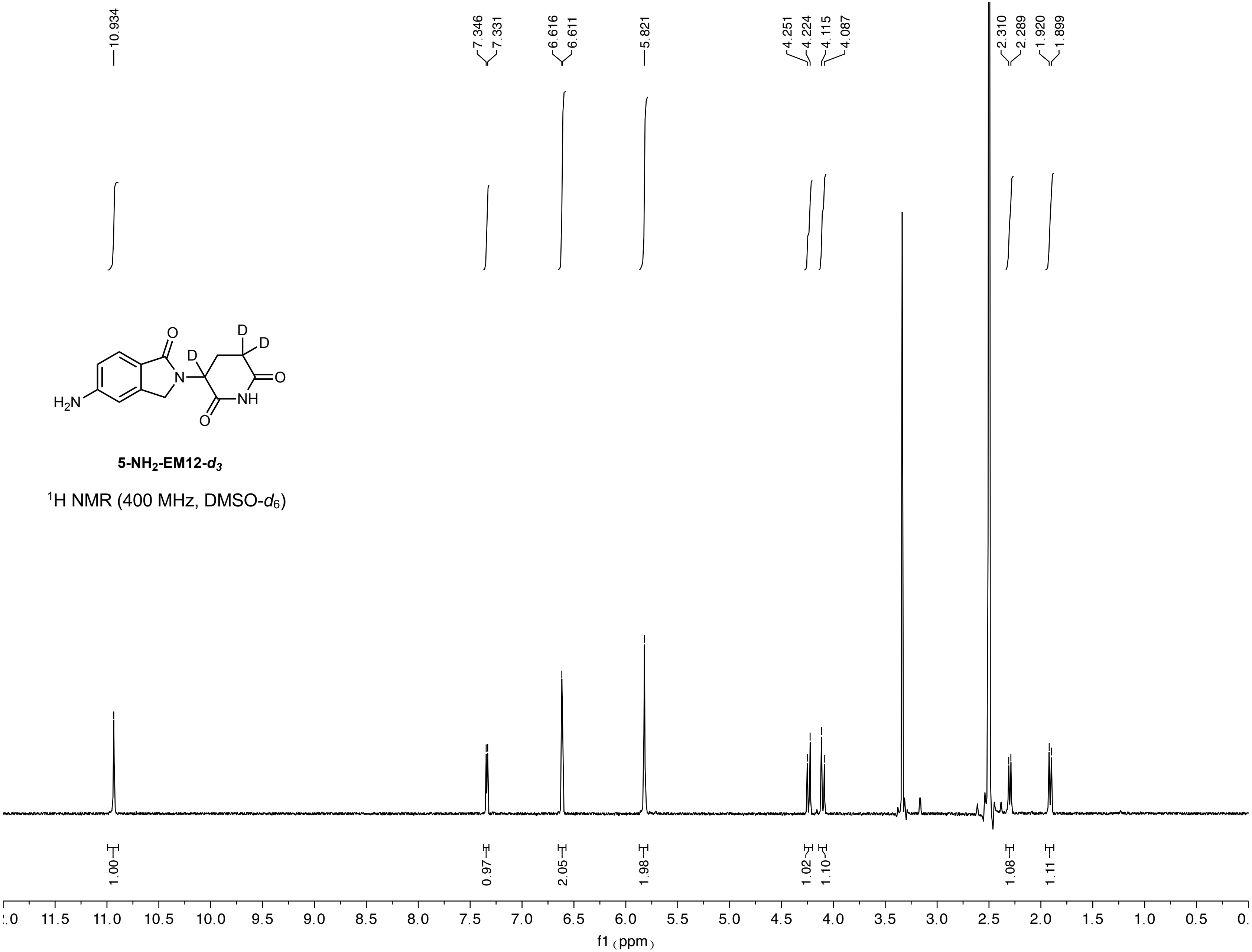

5-NH$_2$-EM12-*d*$_3$

^{1}H NMR (400 MHz, DMSO-d_6)

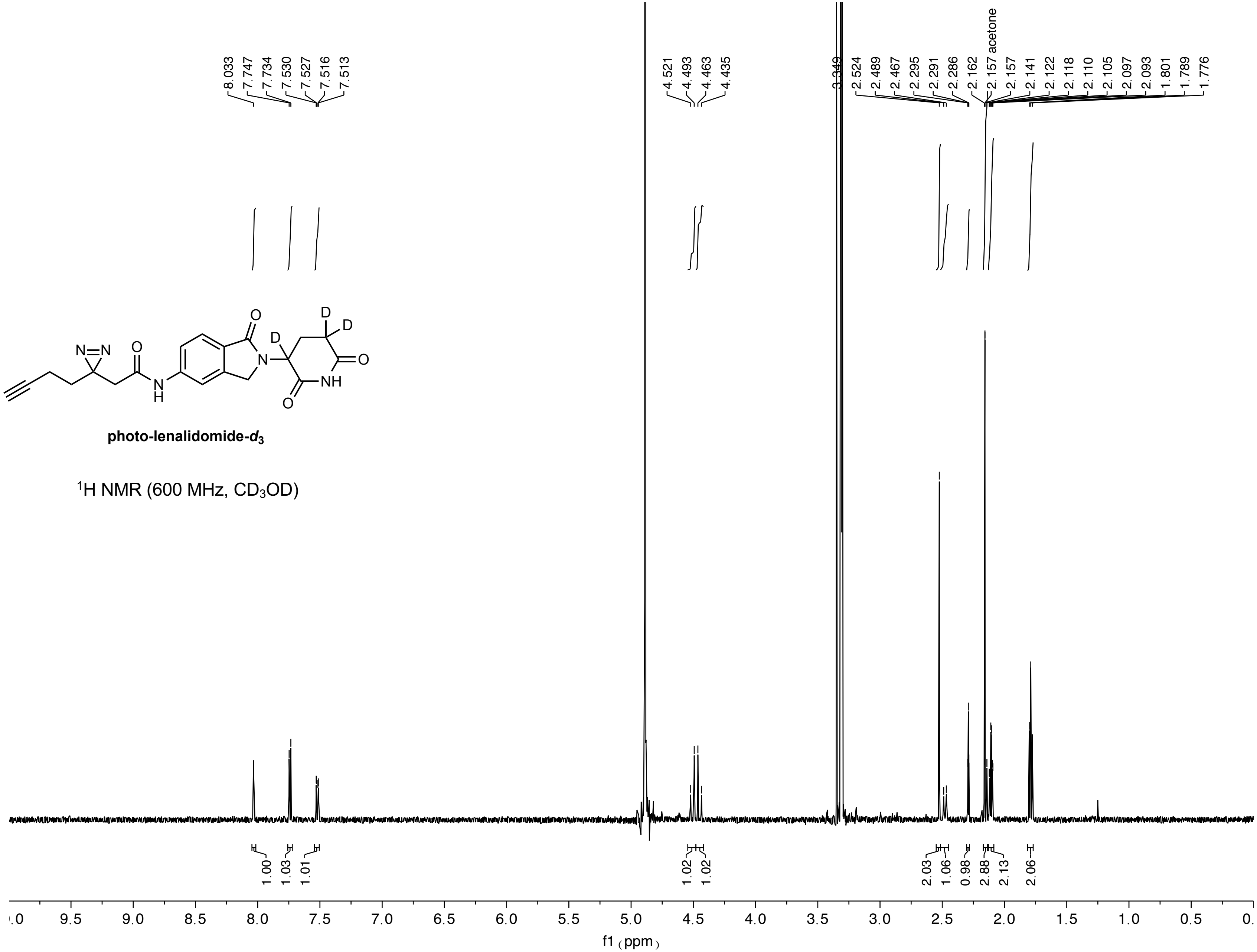

photo-lenalidomide-*d*$_3$

^{1}H NMR (600 MHz, CD_3OD)

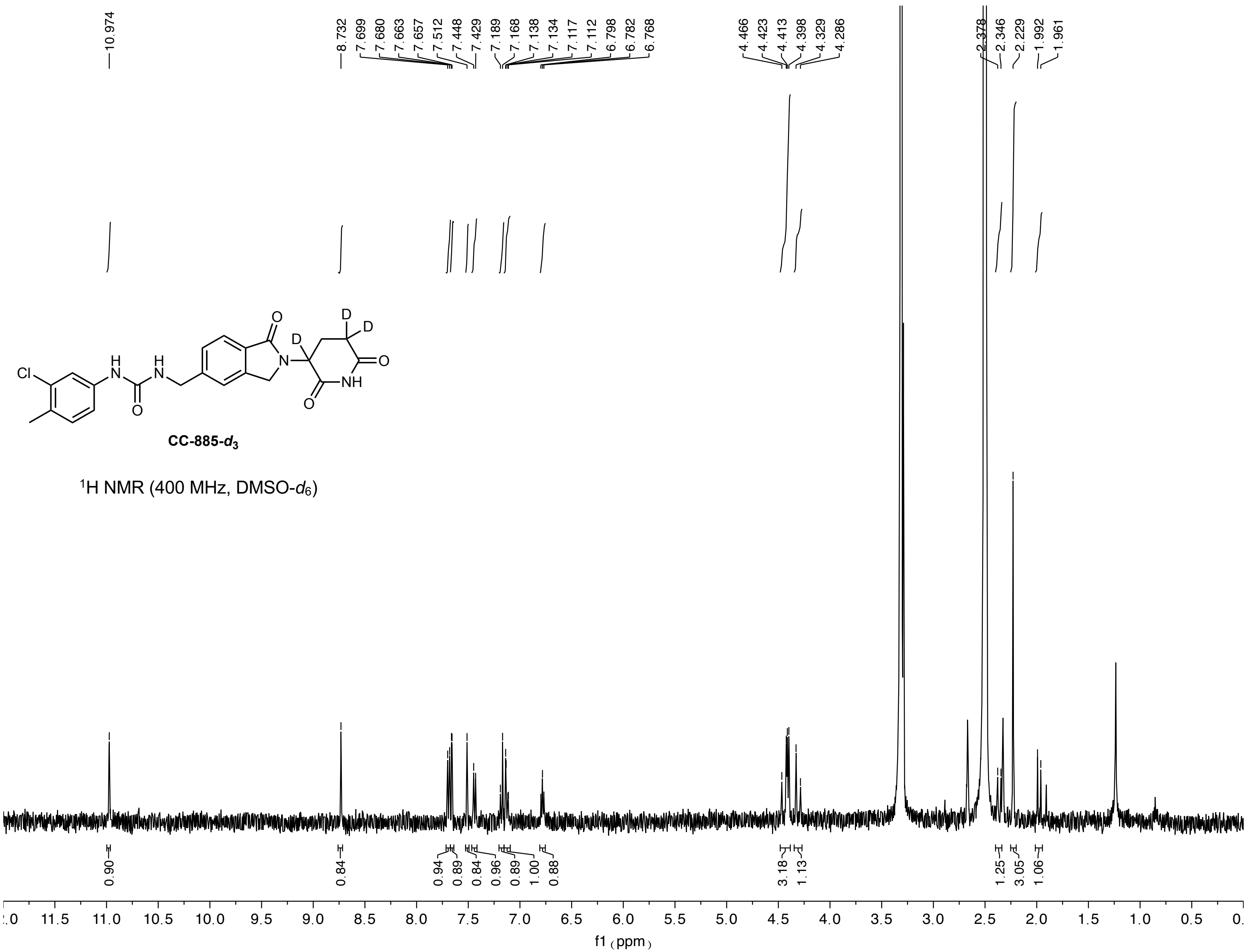

10.974
8.732
7.699
7.680
7.663
7.657
7.512
7.448
7.429
7.189
7.168
7.138
7.134
7.117
7.112
6.798
6.782
6.768
4.466
4.423
4.413
4.398
4.329
4.286
2.378
2.346
2.229
1.992
1.961
CC-885-d_3
^{1}H NMR (400 MHz, DMSO-d_6)
0.90
0.84
0.94
0.89
0.84
0.96
0.89
1.00
0.88
3.18
1.13
1.25
3.05
1.06
f1 (ppm)

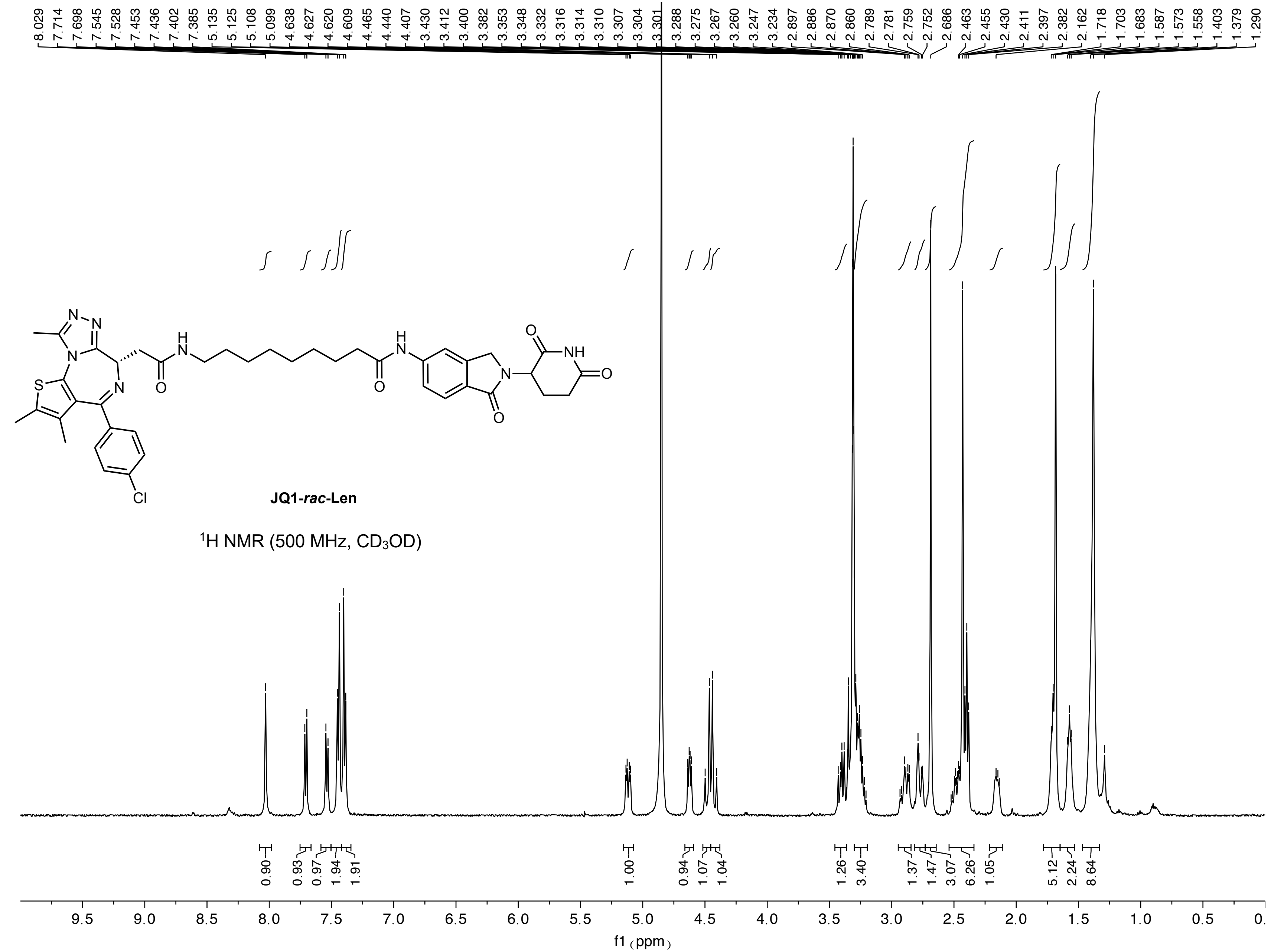
JQ1-*rac*-Len
^{1}H NMR (500 MHz, CD_3OD)
f1 (ppm)

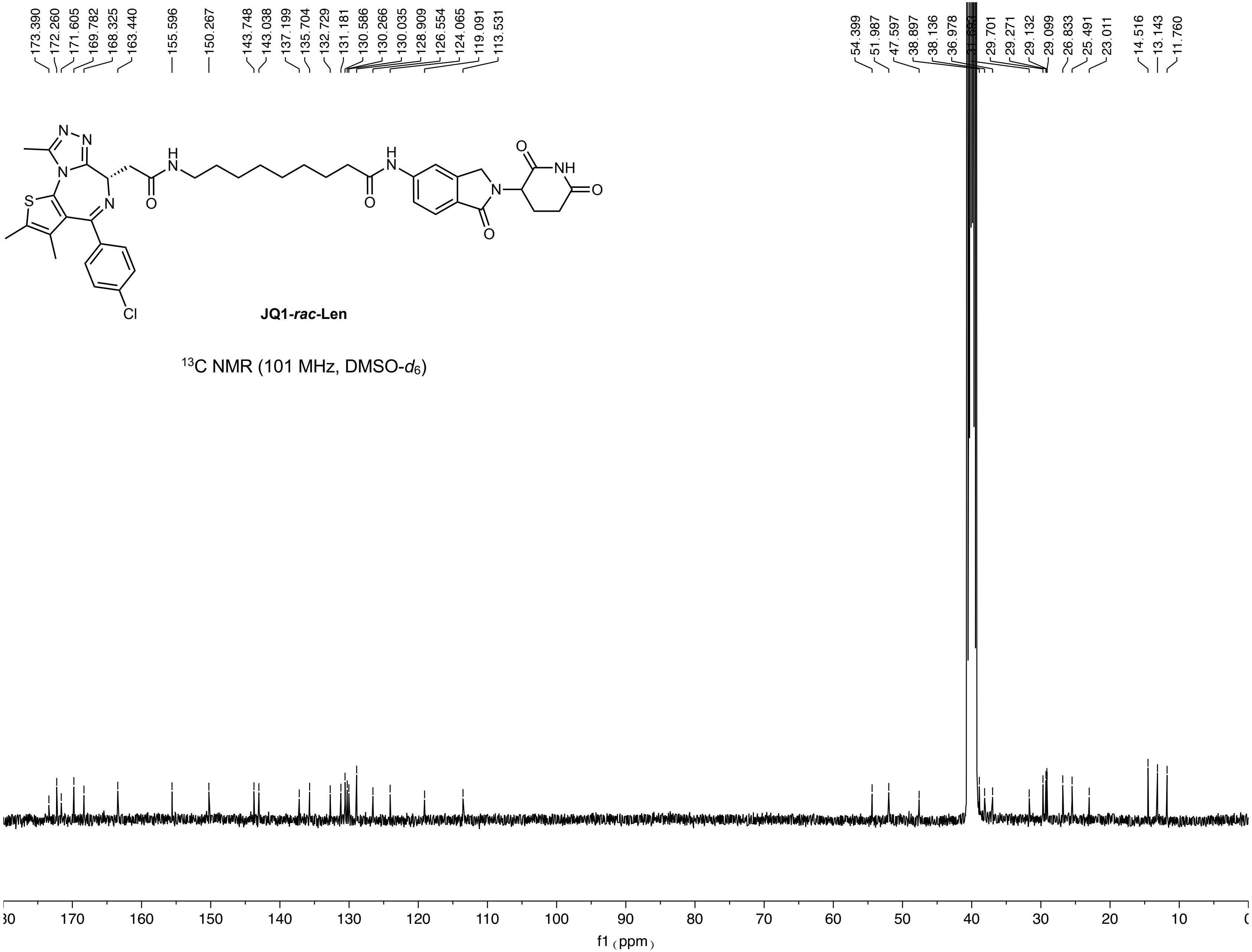

JQ1-*rac*-Len

^{13}C NMR (101 MHz, DMSO-d_6)

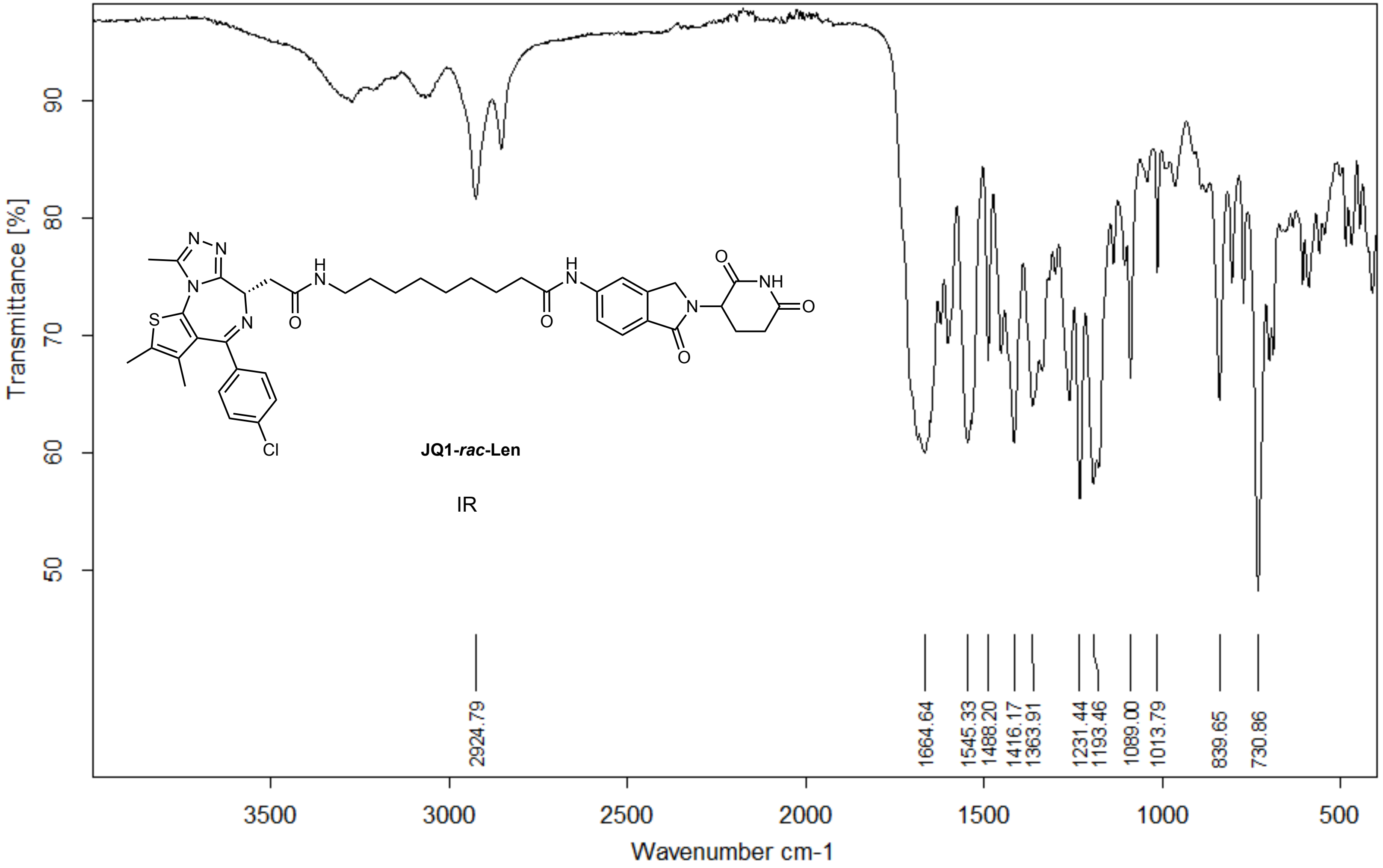

Z:\Woo\Yuka\YA499.0 YA499 Diamond ATR 12/2/2020

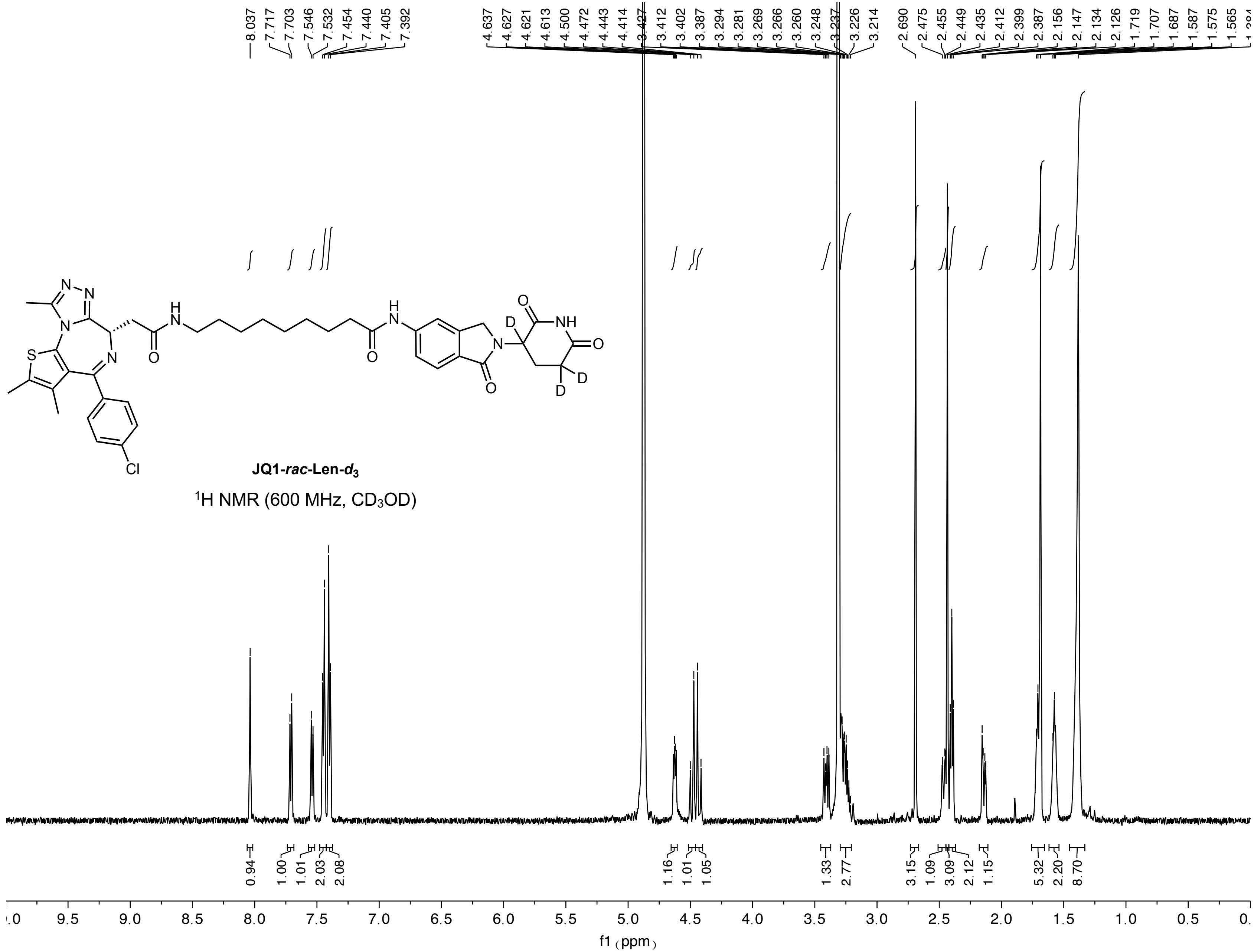

JQ1-*rac*-Len-*d*$_3$

^{1}H NMR (600 MHz, CD_3OD)

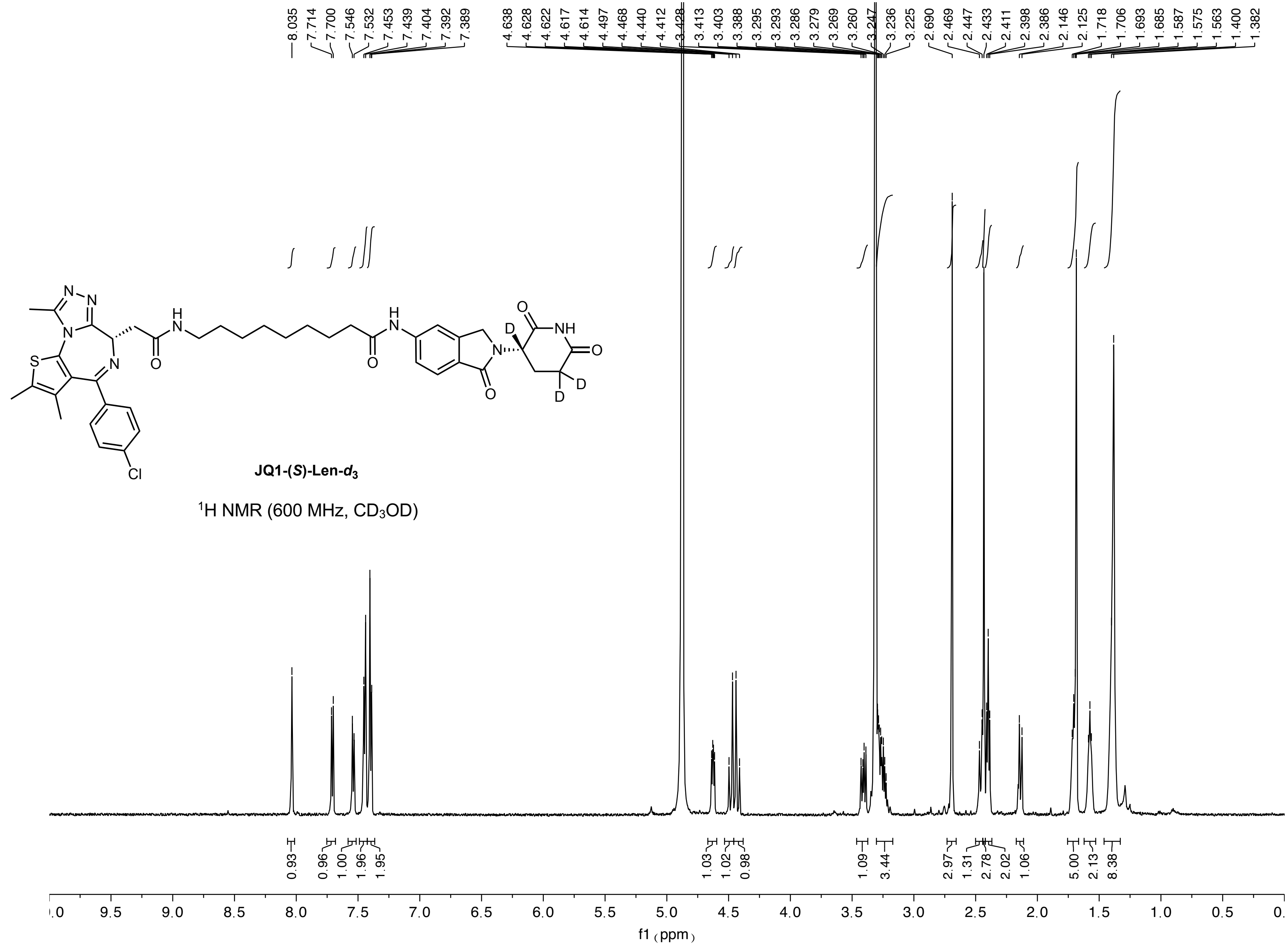

JQ1-(*S*)-Len-d_3

^{1}H NMR (600 MHz, CD_3OD)

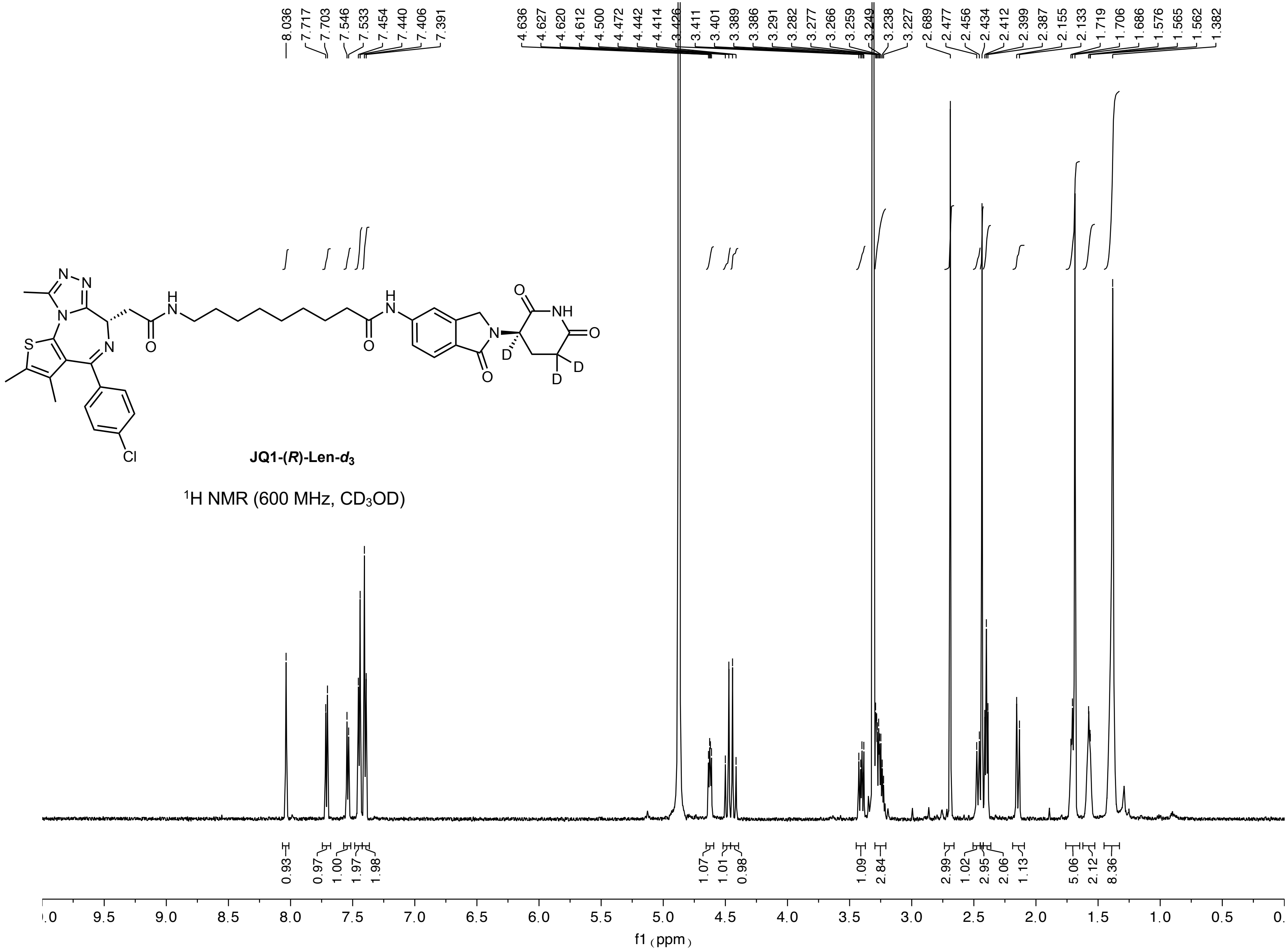

JQ1-(*R*)-Len-*d*$_3$

^{1}H NMR (600 MHz, CD_3OD)

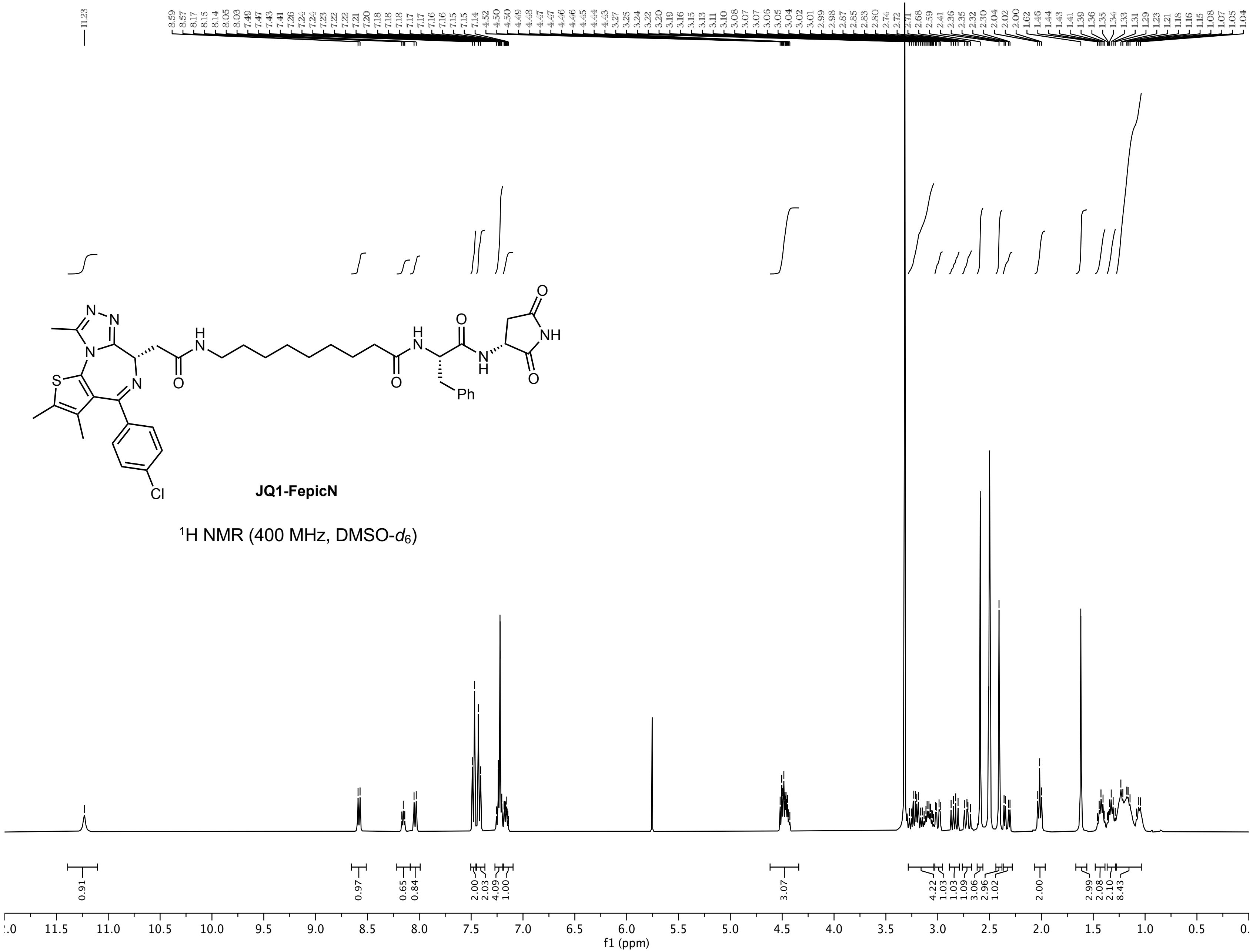
JQ1-FepicN
1H NMR (400 MHz, DMSO-d6)
f1 (ppm)

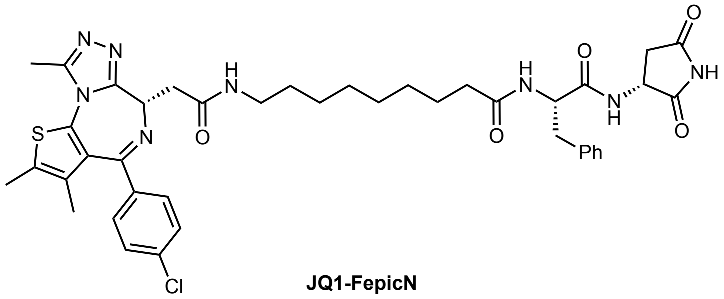

JQ1-FepicN

^{13}C NMR (1201 MHz, DMSO-d_6)

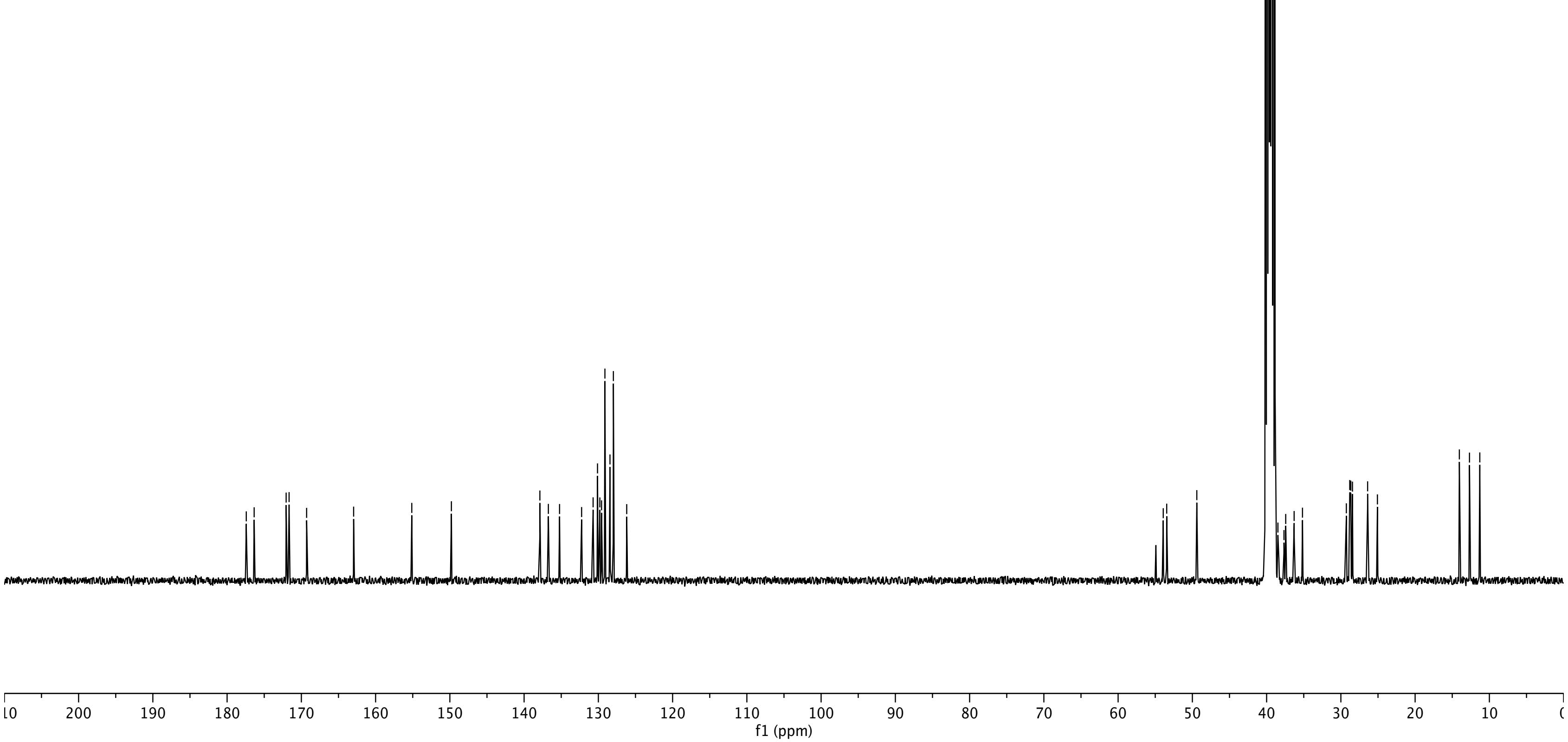

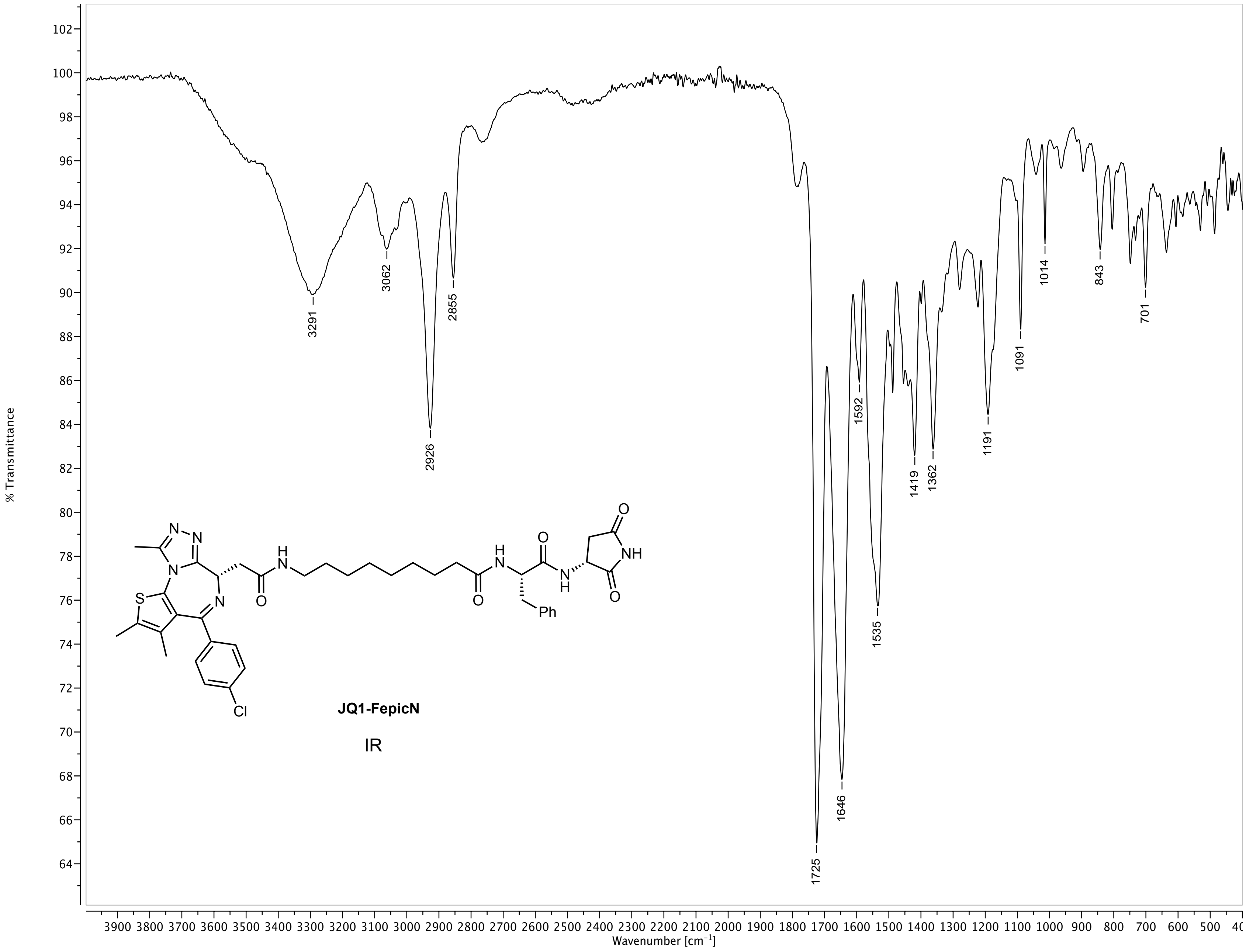
% Transmittance
Wavenumber [cm⁻¹]
3291
3062
2926
2855
1725
1646
1592
1535
1419
1362
1191
1091
1014
843
701
JQ1-FepicN
IR

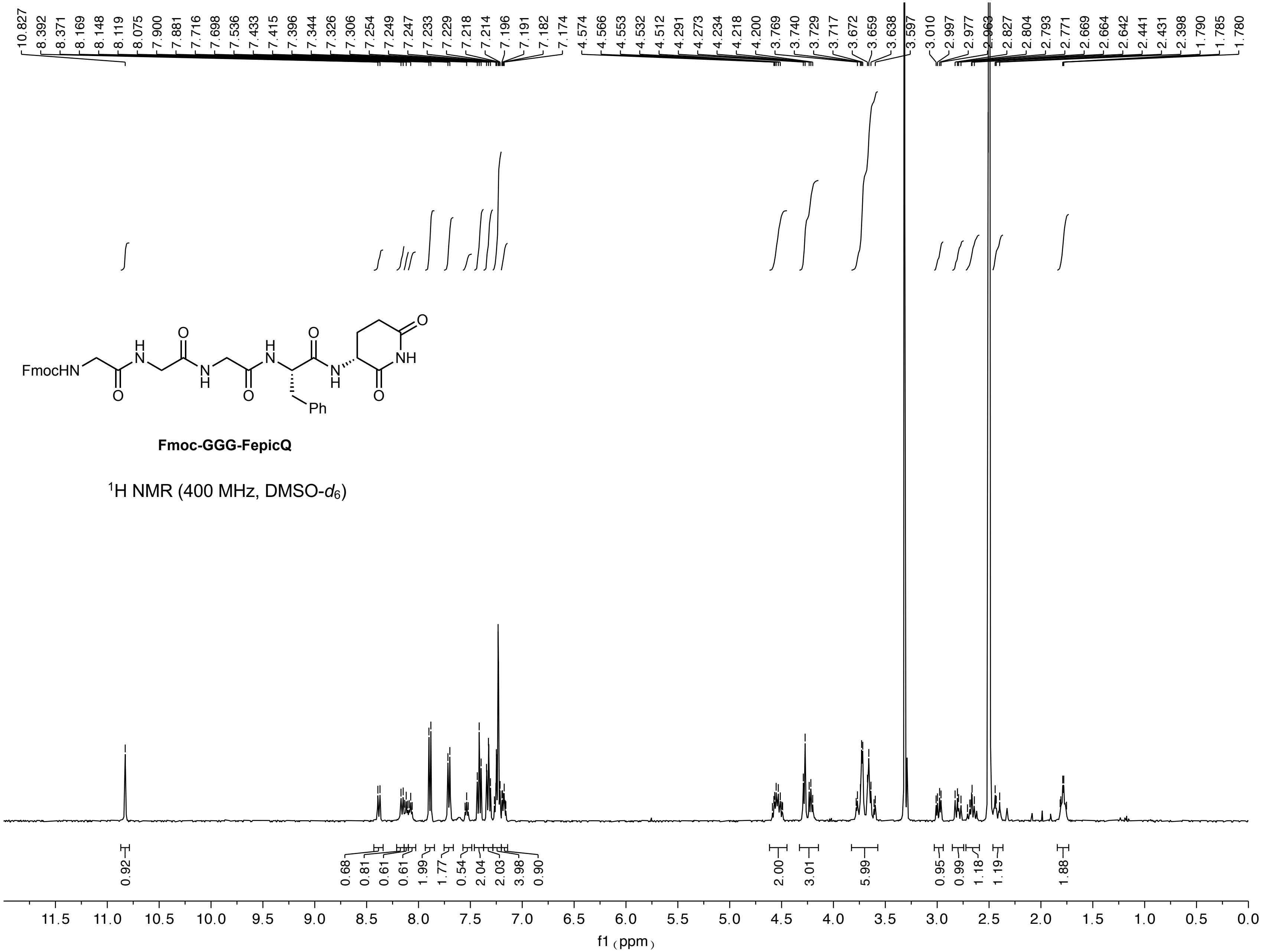

Fmoc-GGG-FepicQ

^{1}H NMR (400 MHz, DMSO-d_6)

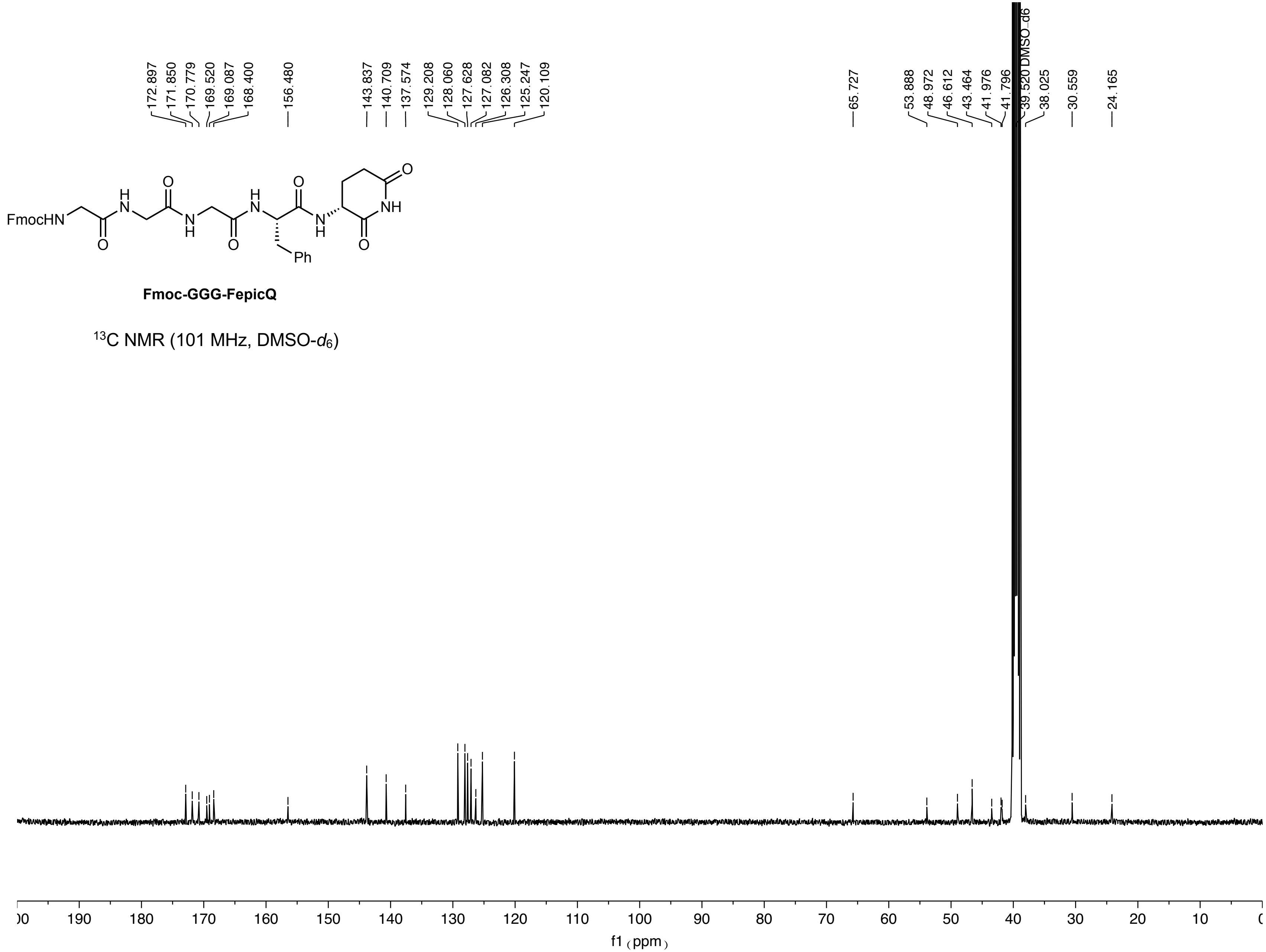

Fmoc-GGG-FepicQ

^{13}C NMR (101 MHz, DMSO-d_6)

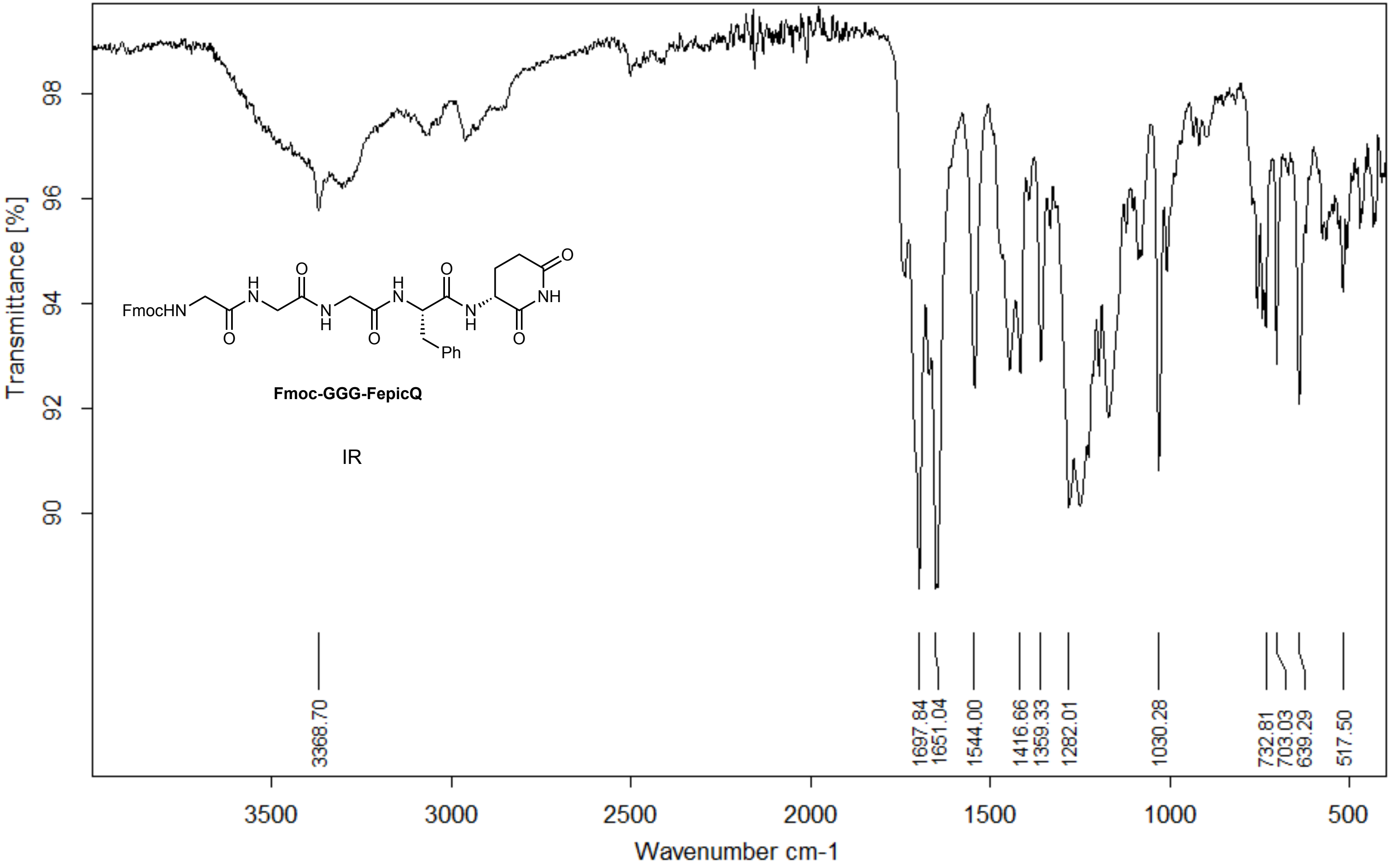

Z:\Woo\Yuka\YA580.0 YA580 Diamond ATR 2/22/2022

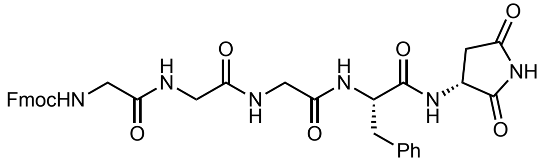

Fmoc-GGG-FepicN

^{1}H NMR (400 MHz, DMSO-d_6)

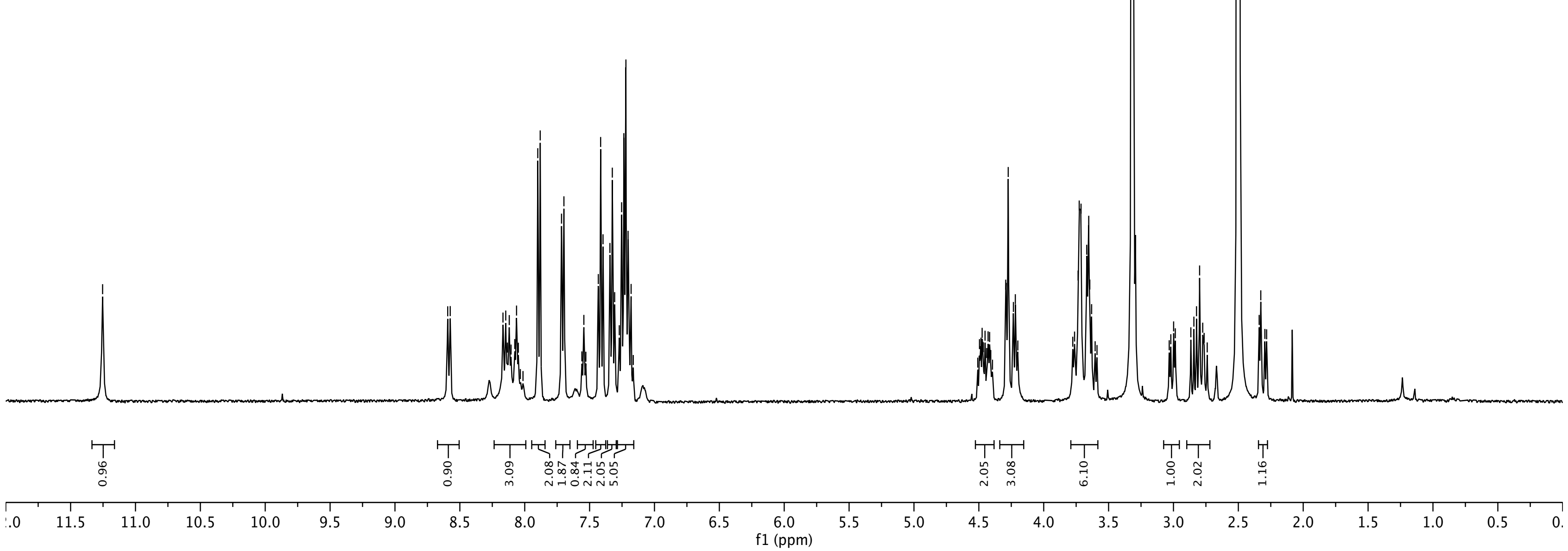

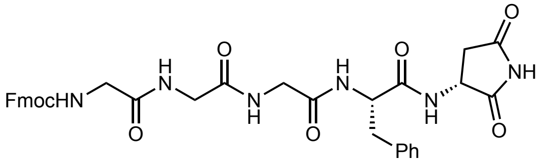

Fmoc-GGG-FepicN

^{13}C NMR (101 MHz, DMSO-d_6)

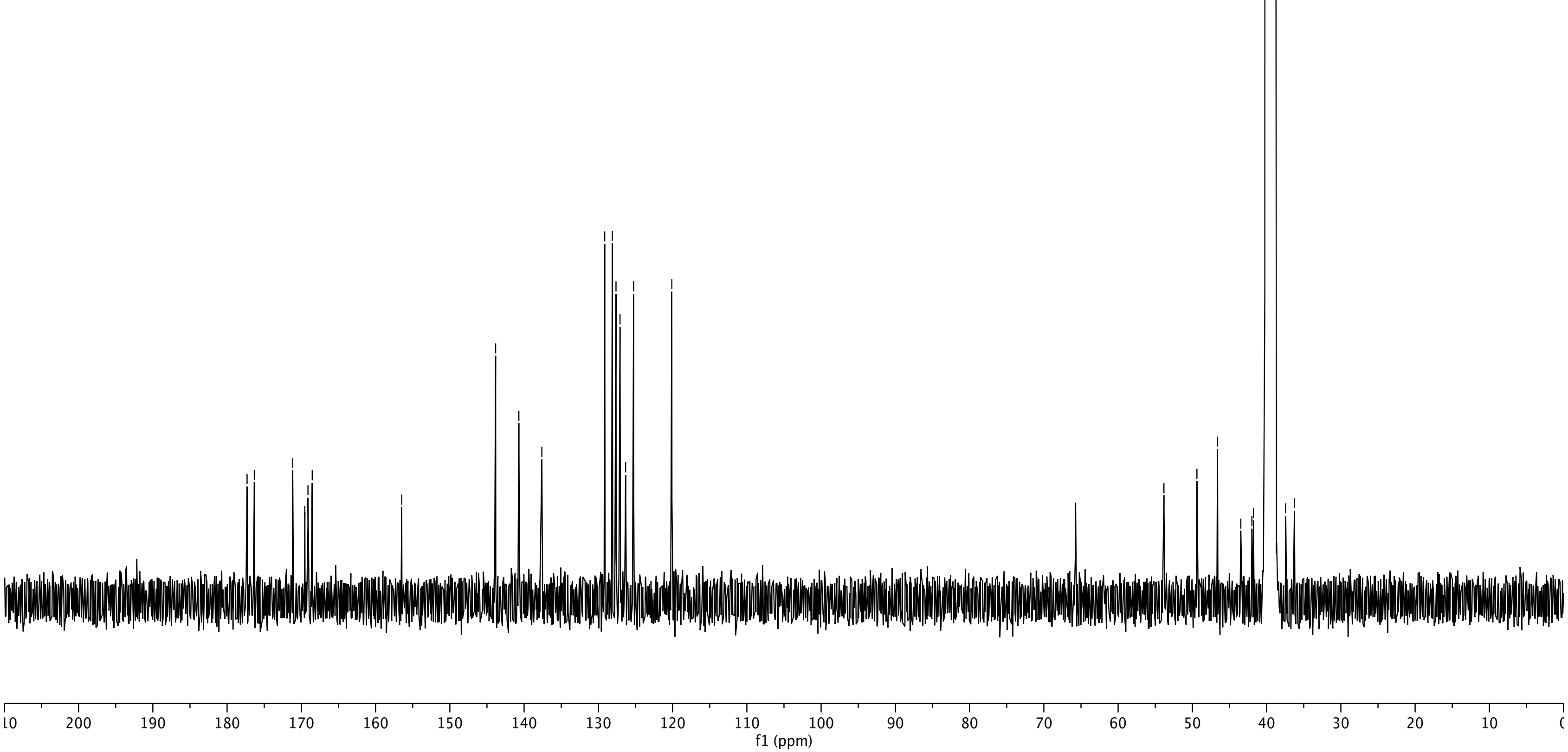

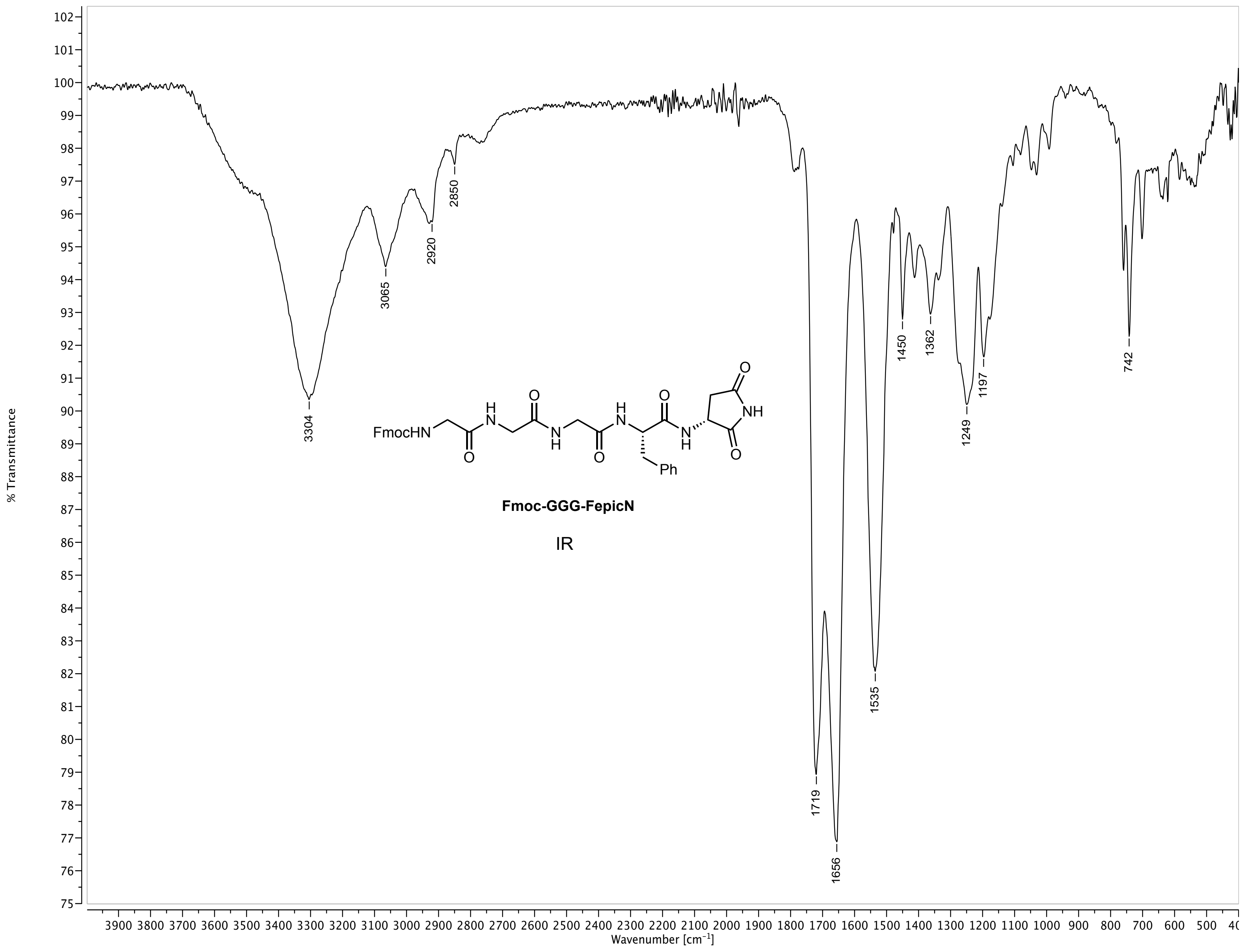

% Transmittance
Wavenumber [cm^{-1}]
3304
3065
2920
2850
1719
1656
1535
1450
1362
1249
1197
742
FmocHN
NH
Ph
Fmoc-GGG-FepicN
IR

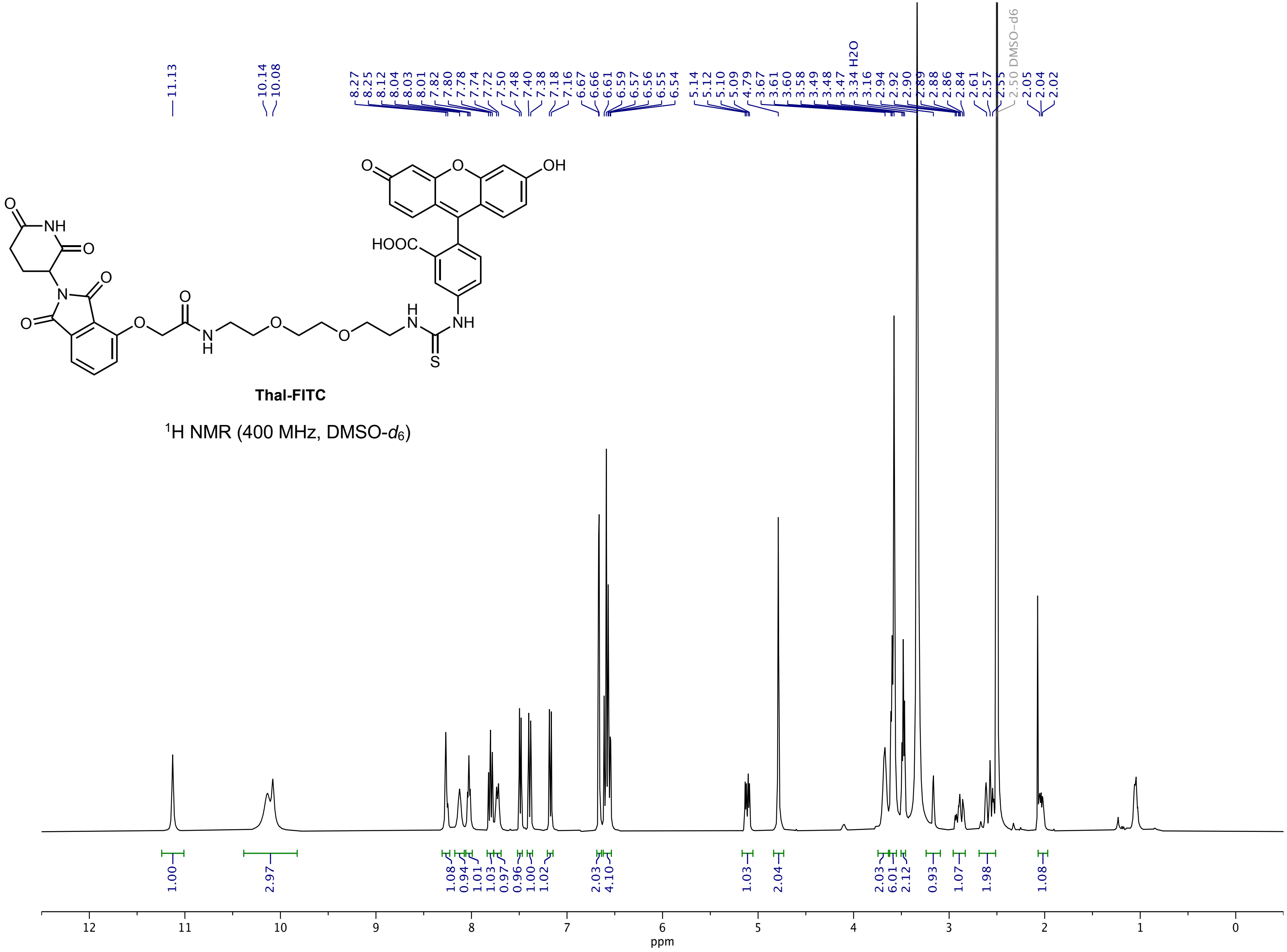

Thal-FITC

^{1}H NMR (400 MHz, DMSO-d_6)

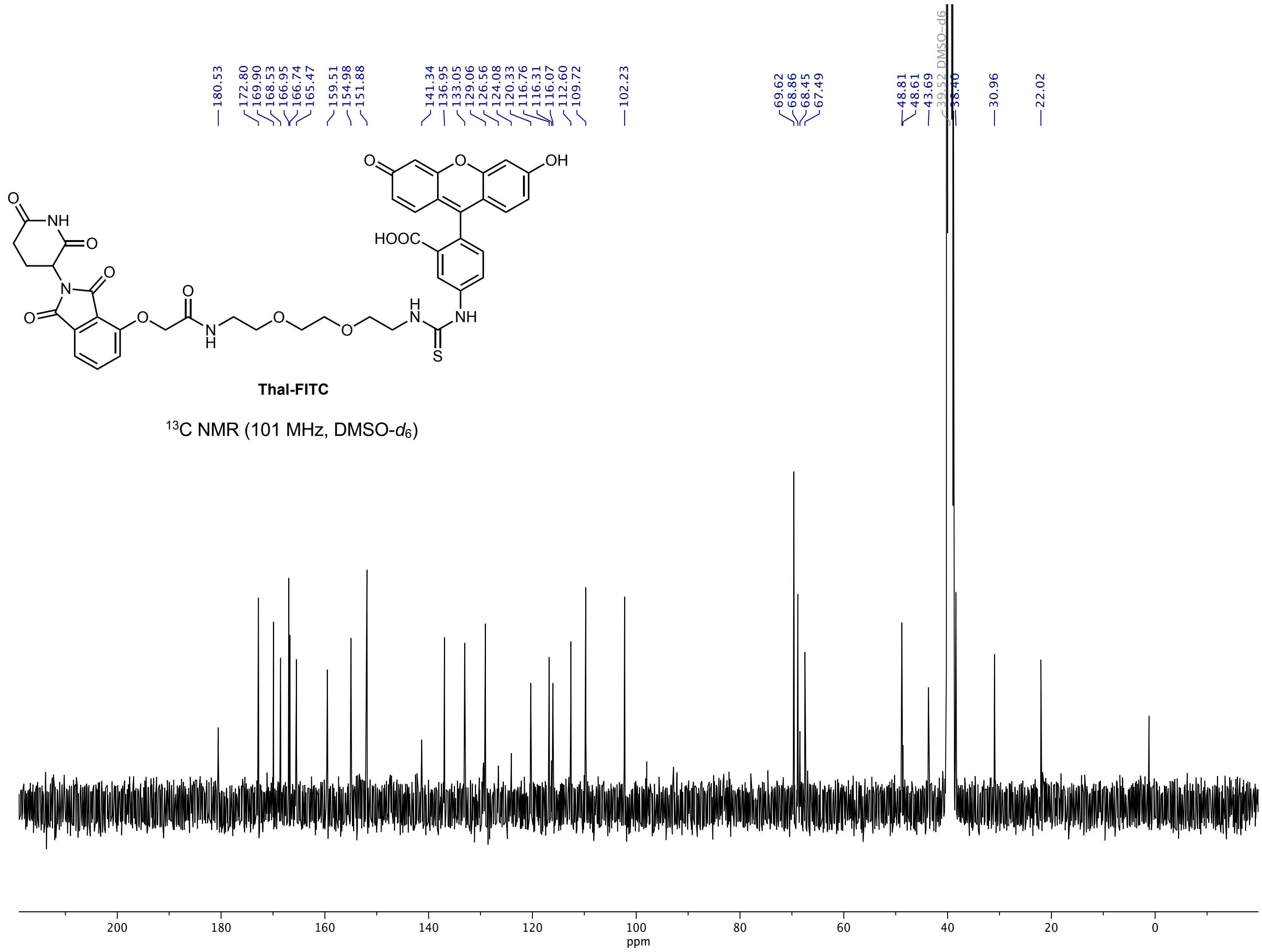

Thal-FITC

^{13}C NMR (101 MHz, DMSO-d_6)

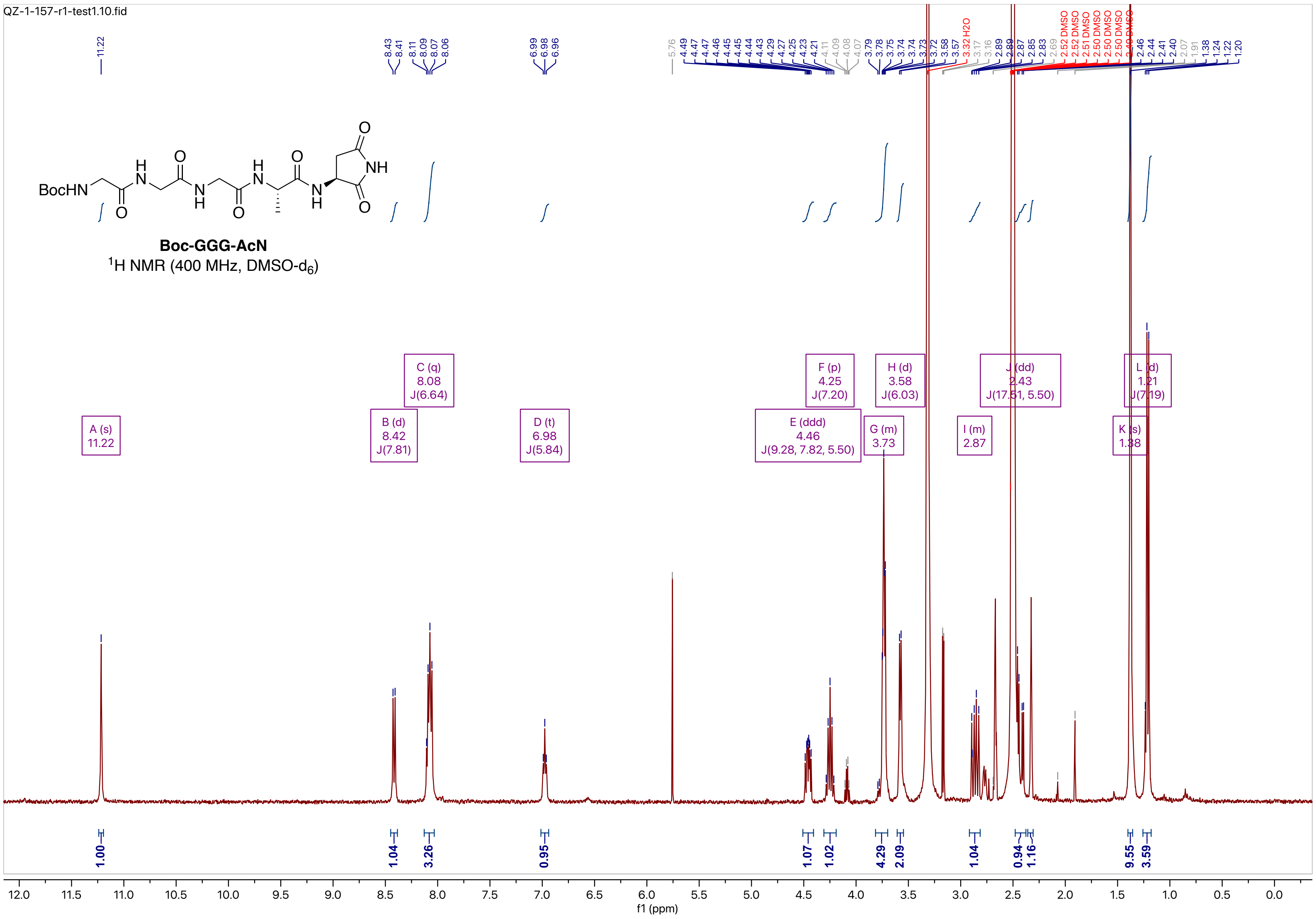
QZ-1-157-r1-test1.10.fid
BocHN
NH
Boc-GGG-AcN
^{1}H NMR (400 MHz, DMSO-d$_6$)
A (s) 11.22
B (d) 8.42 J(7.81)
C (q) 8.08 J(6.64)
D (t) 6.98 J(5.84)
E (ddd) 4.46 J(9.28, 7.82, 5.50)
F (p) 4.25 J(7.20)
G (m) 3.73
H (d) 3.58 J(6.03)
I (m) 2.87
J (dd) 2.43 J(17.51, 5.50)
K (s) 1.38
L (d) 1.21 J(7.19)
f1 (ppm)

QZ-1-160-r1-test2.10.fid

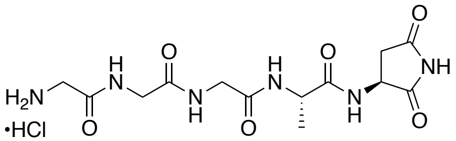

HCl·H_2N-GGG-AcN
^{1}H NMR (400 MHz, CD_3OD)

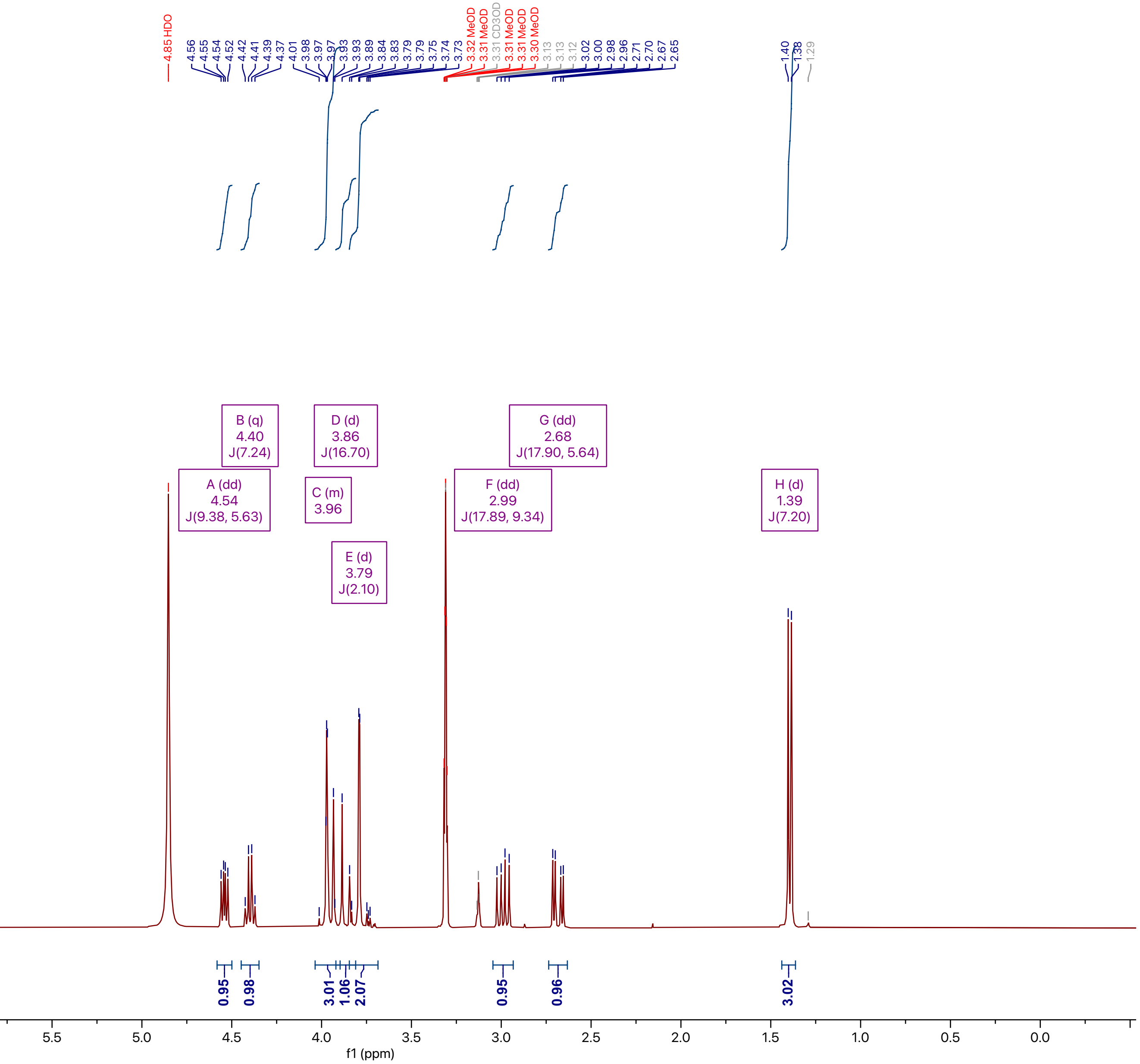

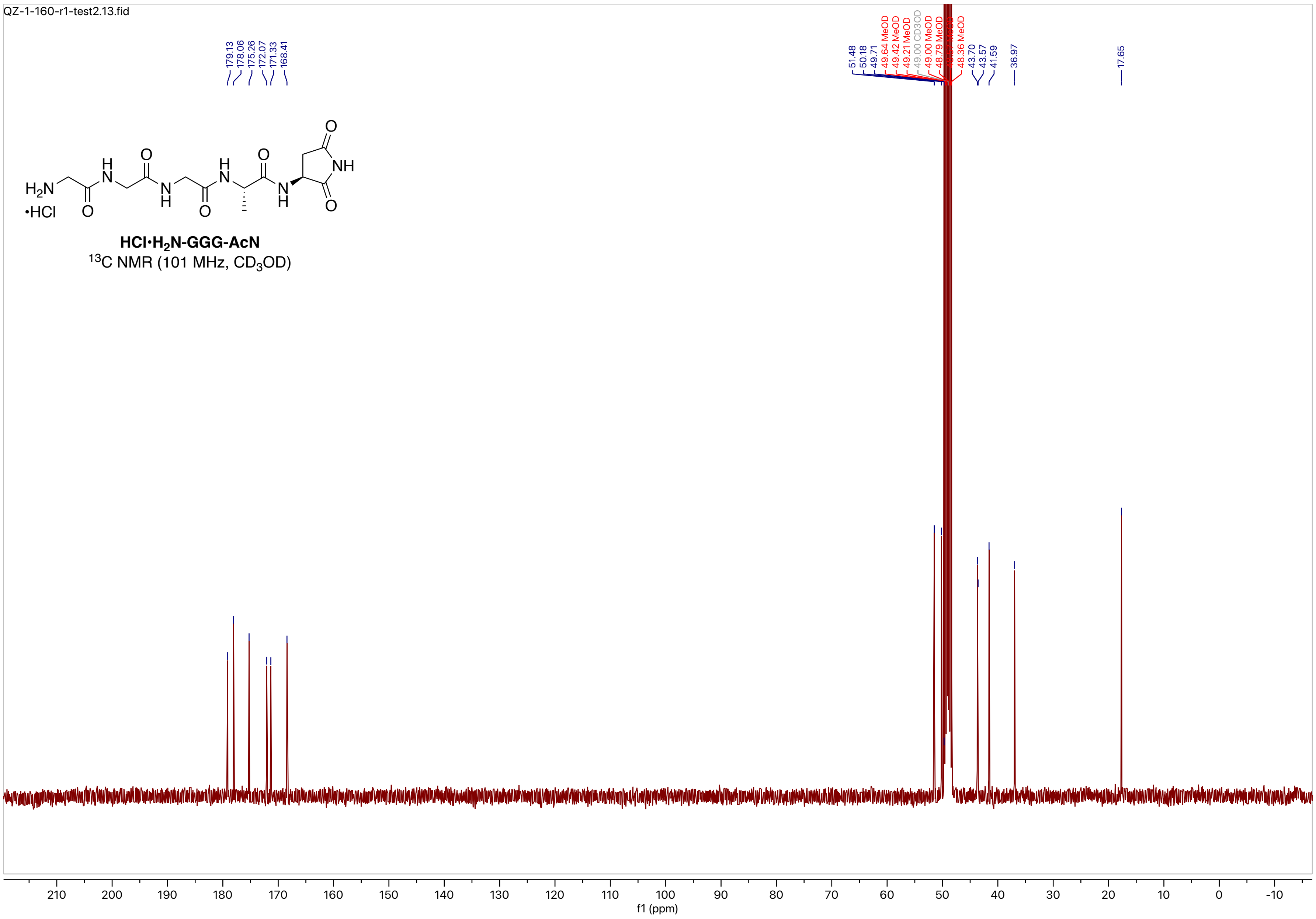
QZ-1-160-r1-test2.13.fid
179.13
178.06
175.26
172.07
171.33
168.41
51.48
50.18
49.71
49.64 MeOD
49.42 MeOD
49.21 MeOD
49.00 CD3OD
49.00 MeOD
48.79 MeOD
48.36 MeOD
43.70
43.57
41.59
36.97
17.65
H2N
•HCl
NH
HCl·H2N-GGG-AcN
13C NMR (101 MHz, CD3OD)
210 200 190 180 170 160 150 140 130 120 110 100 90 80 70 60 50 40 30 20 10 0 -10
f1 (ppm)

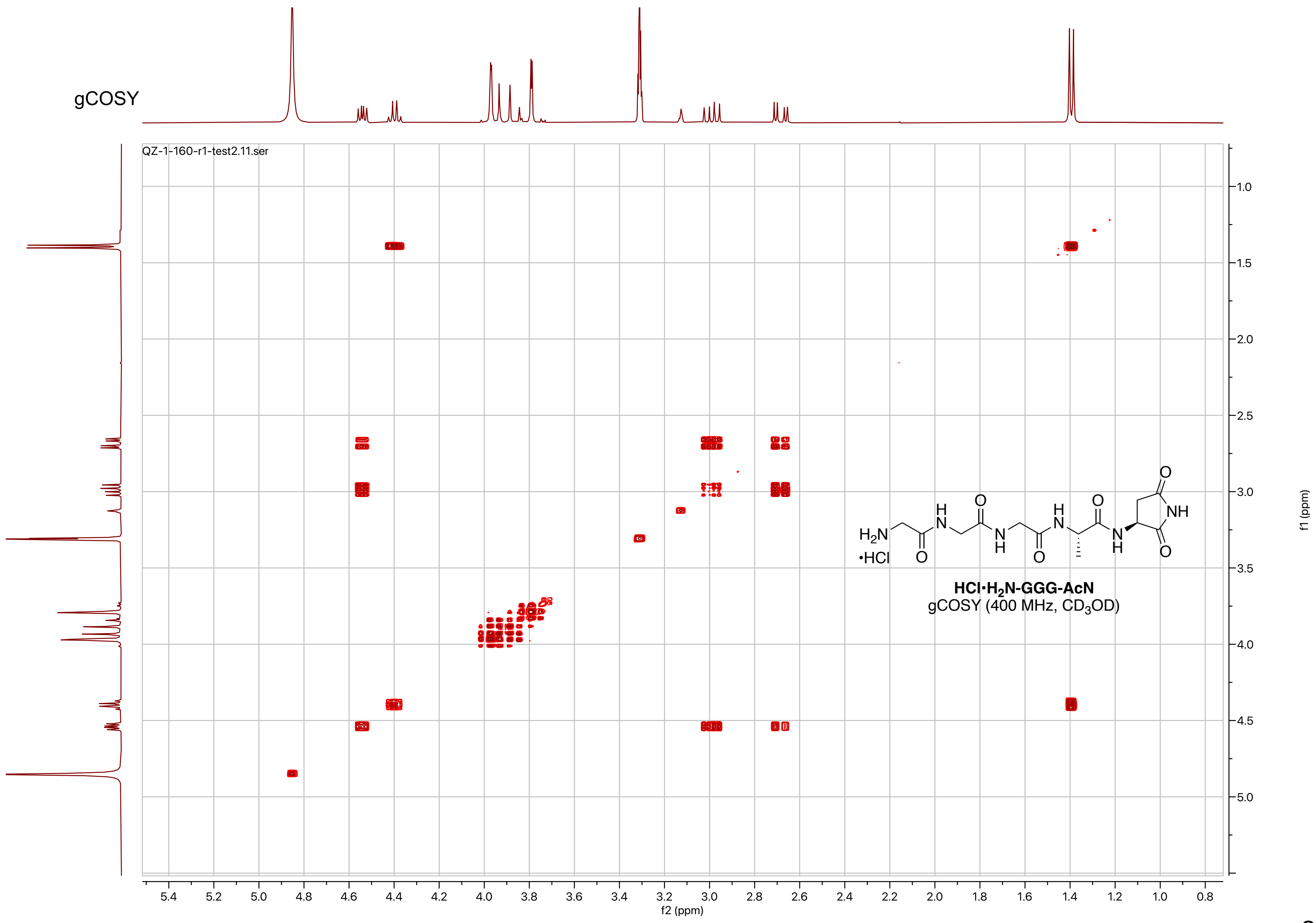
gCOSY
QZ-1-160-r1-test2.11.ser
H2N
•HCl
NH
HCl•H2N-GGG-AcN
gCOSY (400 MHz, CD3OD)
f2 (ppm)
f1 (ppm)

NOESY

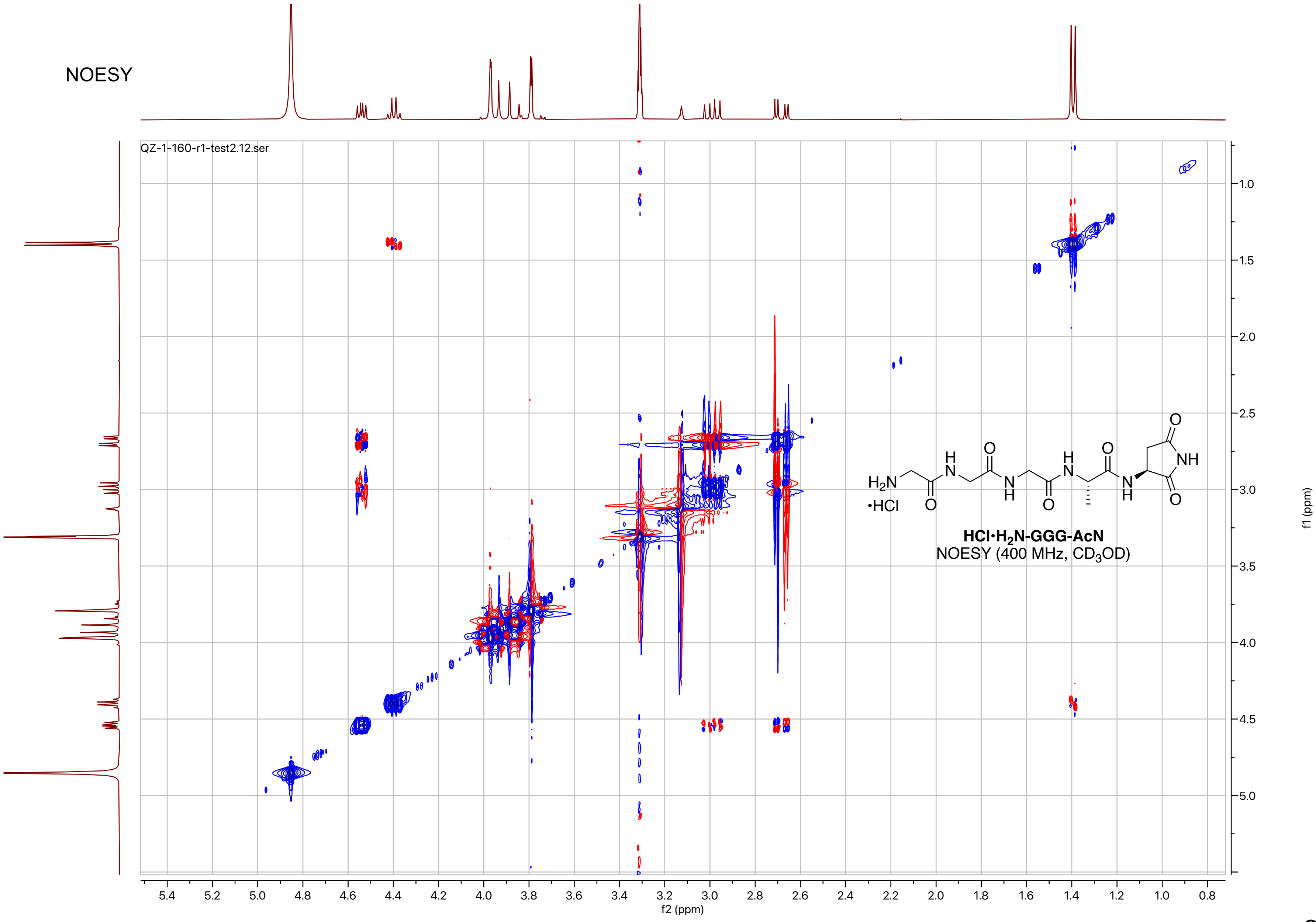

QZ-1-160-r1-test2.12.ser
f2 (ppm)
f1 (ppm)
H2N
•HCl
NH
HCl·H2N-GGG-AcN
NOESY (400 MHz, CD3OD)

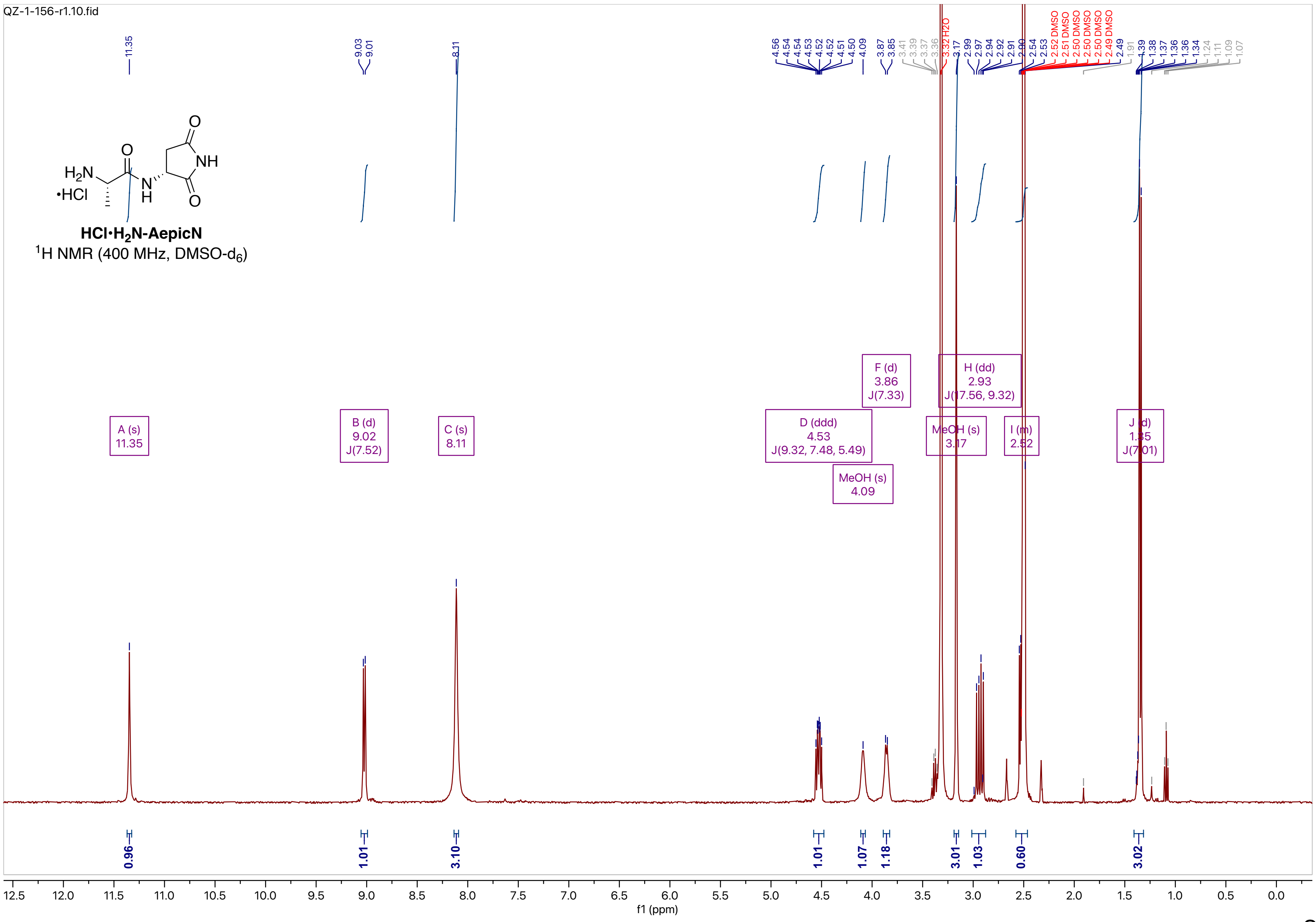
QZ-1-156-r1.10.fid
HCl·H2N-AepicN
1H NMR (400 MHz, DMSO-d6)
A (s) 11.35
B (d) 9.02 J(7.52)
C (s) 8.11
D (ddd) 4.53 J(9.32, 7.48, 5.49)
MeOH (s) 4.09
F (d) 3.86 J(7.33)
MeOH (s) 3.17
H (dd) 2.93 J(17.56, 9.32)
I (m) 2.52
J (d) 1.35 J(7.01)
f1 (ppm)

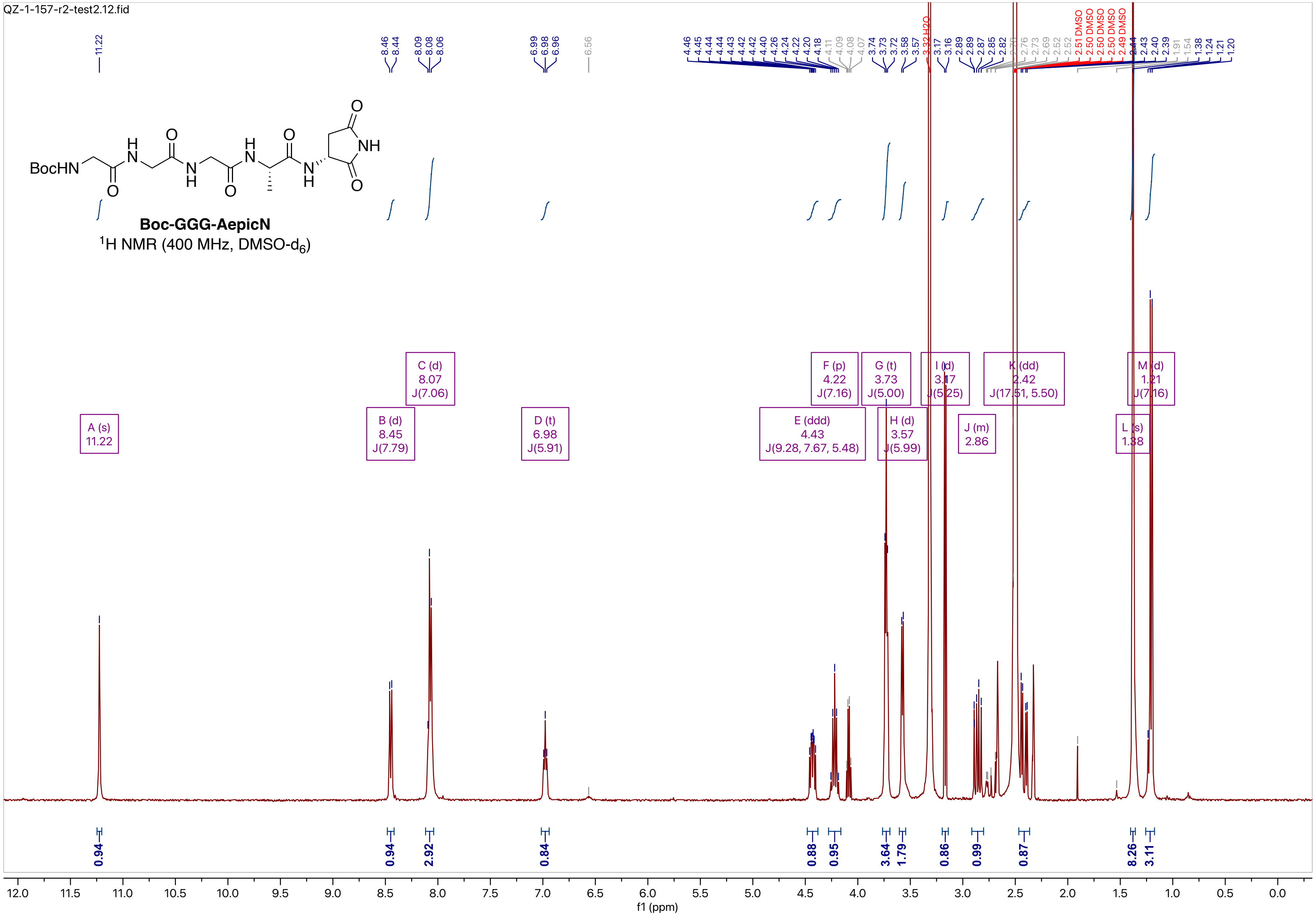
QZ-1-157-r2-test2.12.fid
BocHN
Boc-GGG-AepicN
1H NMR (400 MHz, DMSO-d6)
A (s) 11.22
B (d) 8.45 J(7.79)
C (d) 8.07 J(7.06)
D (t) 6.98 J(5.91)
E (ddd) 4.43 J(9.28, 7.67, 5.48)
F (p) 4.22 J(7.16)
G (t) 3.73 J(5.00)
H (d) 3.57 J(5.99)
I (d) 3.17 J(5.25)
J (m) 2.86
K (dd) 2.42 J(17.51, 5.50)
L (s) 1.38
M (d) 1.21 J(7.16)
f1 (ppm)

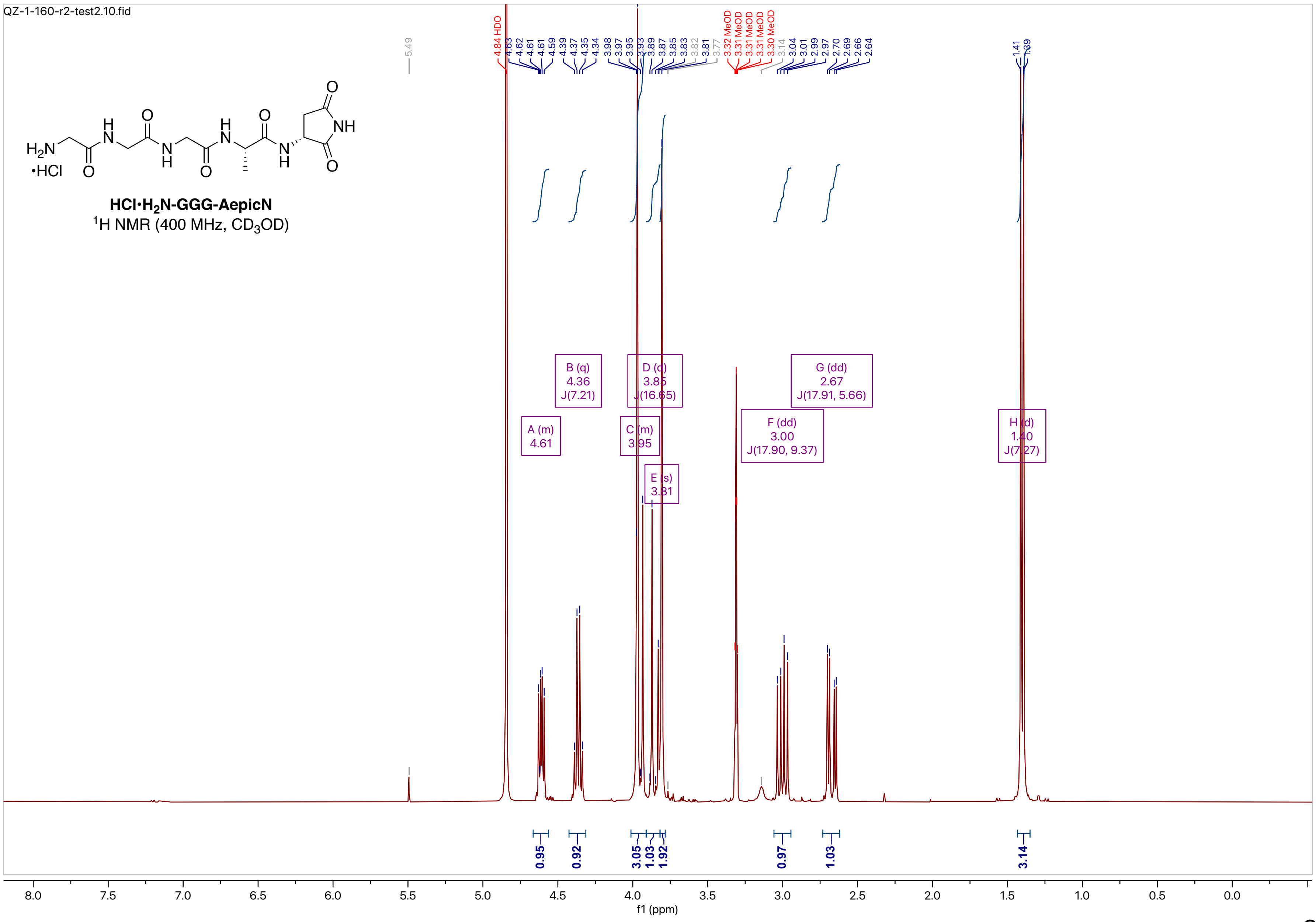
QZ-1-160-r2-test2.10.fid
HCl•H2N-GGG-AepicN
1H NMR (400 MHz, CD3OD)
A (m) 4.61
B (q) 4.36 J(7.21)
C (m) 3.95
D (q) 3.85 J(16.65)
E (s) 3.81
F (dd) 3.00 J(17.90, 9.37)
G (dd) 2.67 J(17.91, 5.66)
H (d) 1.40 J(7.27)
4.84 HDO
3.32 MeOD
3.31 MeOD
3.31 MeOD
3.31 MeOD
3.30 MeOD
0.95
0.92
3.05
1.03
1.92
0.97
1.03
3.14
f1 (ppm)

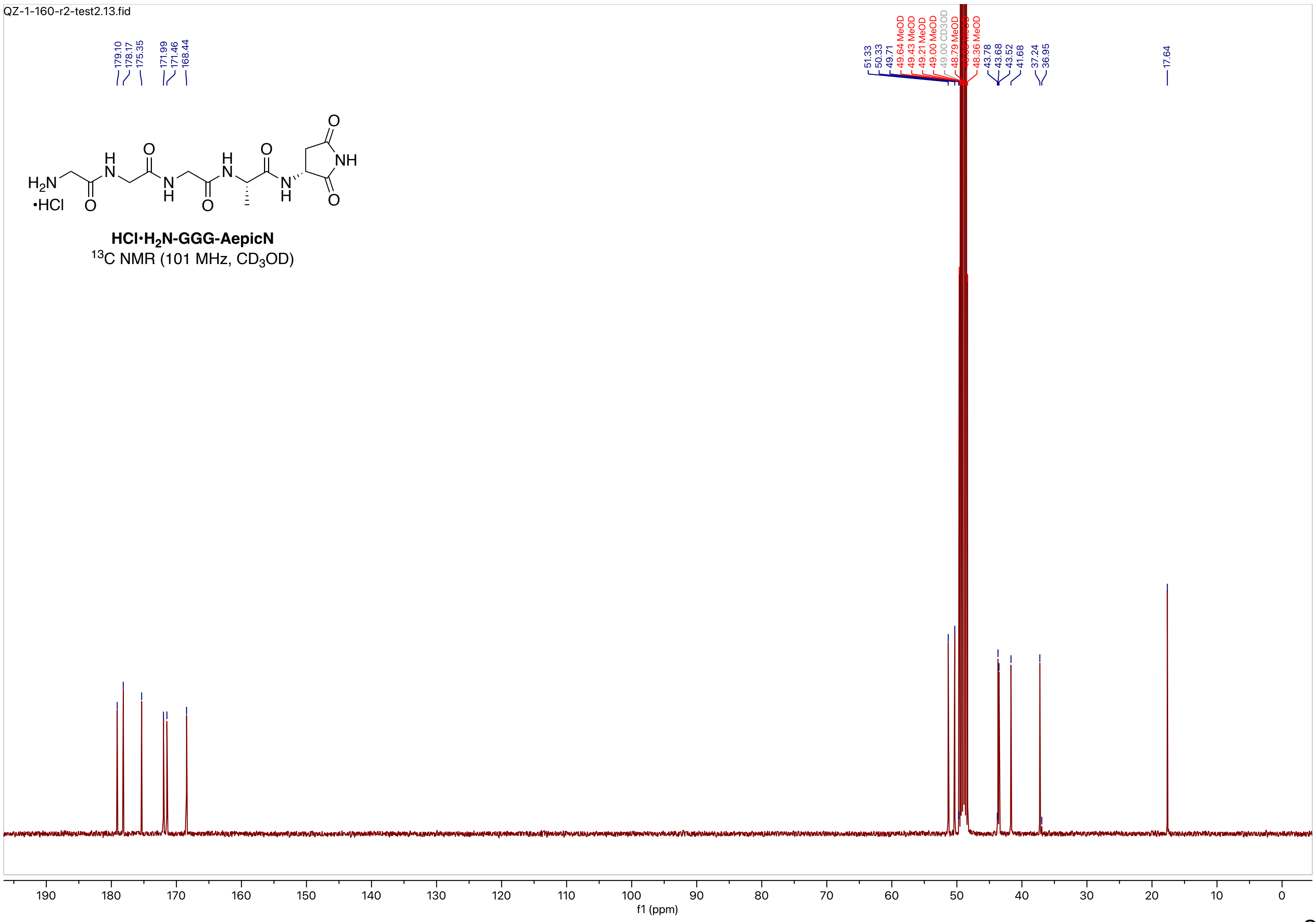
QZ-1-160-r2-test2.13.fid
179.10
178.17
175.35
171.99
171.46
168.44
51.33
50.33
49.71
49.64 MeOD
49.43 MeOD
49.21 MeOD
49.00 MeOD
49.00 CD3OD
48.79 MeOD
48.36 MeOD
43.78
43.68
43.52
41.68
37.24
36.95
17.64
H2N
•HCl
HCl•H2N-GGG-AepicN
13C NMR (101 MHz, CD3OD)
190
180
170
160
150
140
130
120
110
100
90
80
70
60
50
40
30
20
10
0
f1 (ppm)

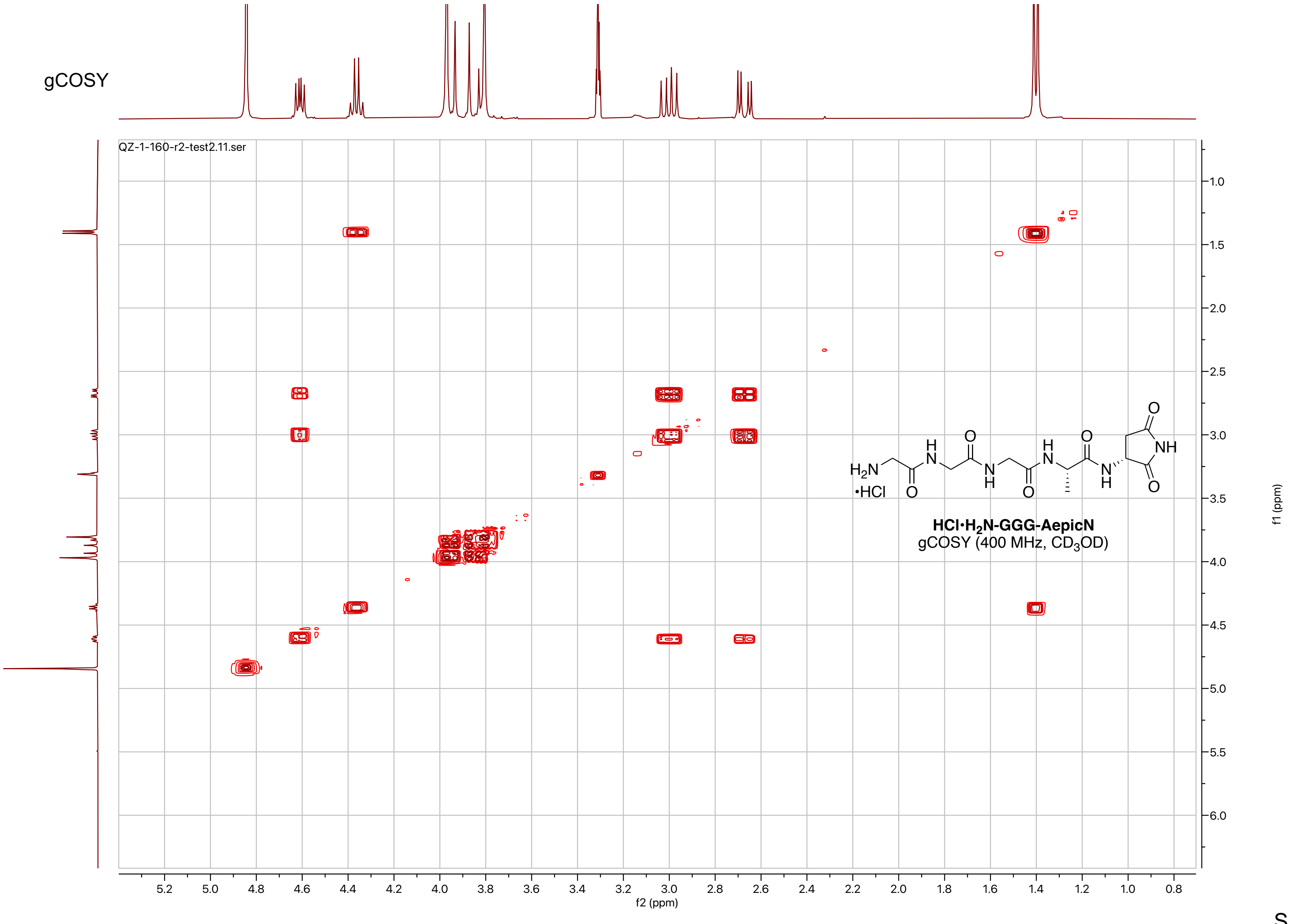
gCOSY
QZ-1-160-r2-test2.11.ser
H2N
•HCl
HCl•H2N-GGG-AepicN
gCOSY (400 MHz, CD3OD)
f2 (ppm)
f1 (ppm)

NOESY

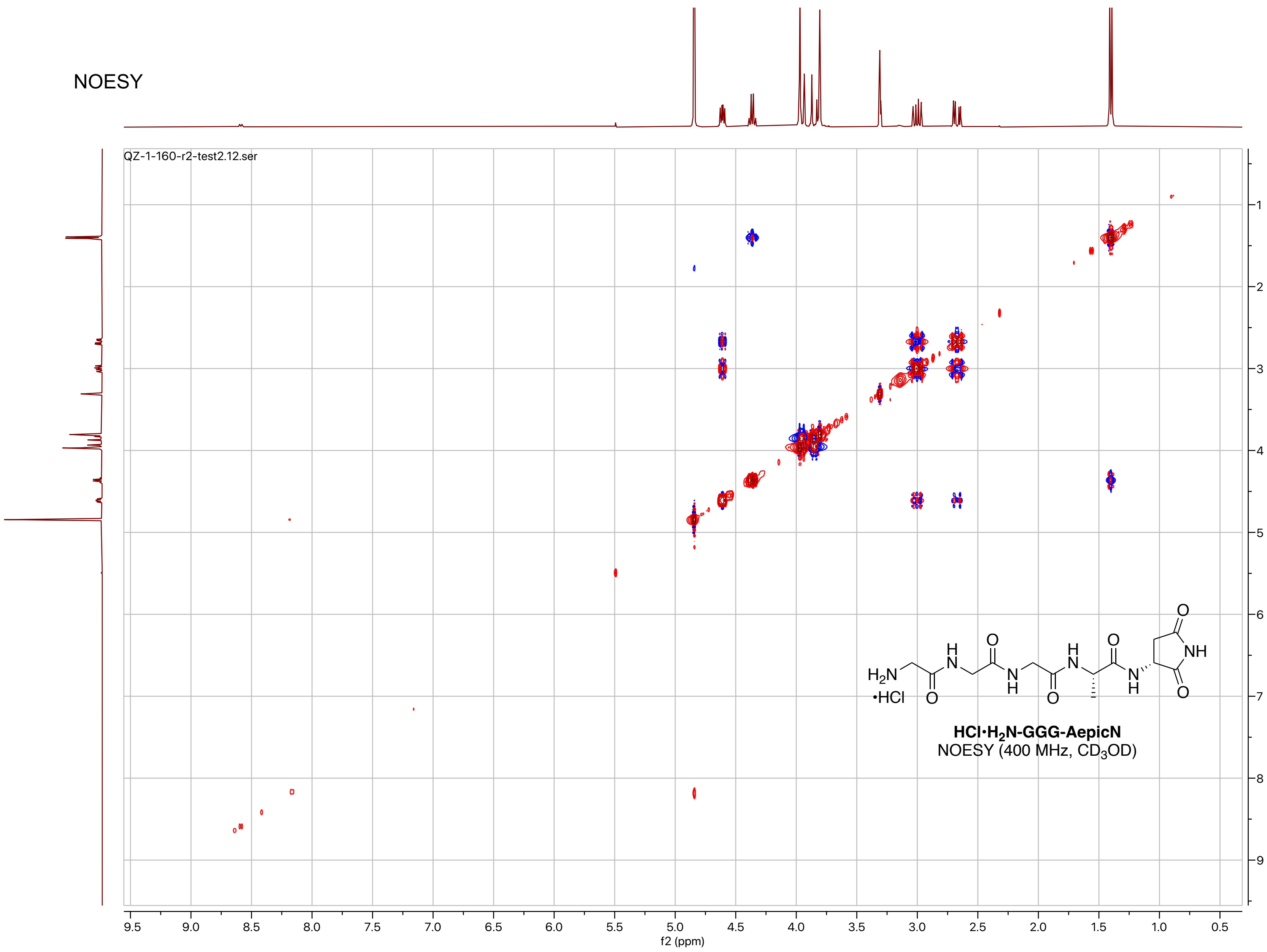
QZ-1-160-r2-test2.12.ser
f2 (ppm)
HCl•H2N-GGG-AepicN
NOESY (400 MHz, CD3OD)
